# Nickel-catalyzed allylic defluorinative alkylation of trifluoromethyl alkenes with reductive decarboxylation of redox-active esters†
†Electronic supplementary information (ESI) available: Experimental protocols and spectral data. See DOI: 10.1039/c8sc04335c


**DOI:** 10.1039/c8sc04335c

**Published:** 2018-11-07

**Authors:** Xi Lu, Xiao-Xu Wang, Tian-Jun Gong, Jing-Jing Pi, Shi-Jiang He, Yao Fu

**Affiliations:** a Hefei National Laboratory for Physical Sciences at the Microscale , CAS Key Laboratory of Urban Pollutant Conversion , Anhui Province Key Laboratory of Biomass Clean Energy , iChEM , University of Science and Technology of China , Hefei 230026 , China . Email: luxi@mail.ustc.edu.cn ; Email: fuyao@ustc.edu.cn

## Abstract

Herein, we report a nickel-catalyzed allylic defluorinative alkylation of trifluoromethyl alkenes through reductive decarboxylation of redox-active esters. The present reaction enables the preparation of functionalized *gem*-difluoroalkenes with the formation of sterically hindered C(sp^3^)–C(sp^3^) bonds under very mild reaction conditions, while tolerating many sensitive functional groups and requiring minimal substrate protection. Therefore, this method provides an efficient and convenient approach for late-stage modification of biologically interesting molecules.

## Abstract 2

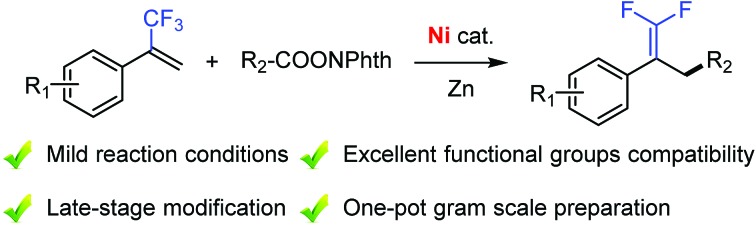
Synthesis of functionalized *gem*-difluoroalkenes was achieved through nickel-catalyzed allylic defluorinative alkylation of trifluoromethyl alkenes with reductive decarboxylation of redox-active esters.

## Introduction

Efficient strategies for introducing fluorine-containing fragments into organic compounds exert positive influences on biochemical sciences, because these fluorochemicals have superior bioactivity and physicochemical characteristics compared to their non-fluorinated counterparts.^1^ Among them, *gem*-difluoroalkenes are a class of structurally superior fluorine-containing compounds, and they have attracted substantial interest in agrochemistry and medicinal chemistry. For instance, the *gem*-difluoroethylene moiety is widely used as an ideal carbonyl group bioisostere in drug design.^2^ In addition, the *gem*-difluoroethylene moiety can be easily transformed into other fluorine-containing structures such as monofluoroalkenyl, difluoromethylenyl, and trifluoromethyl groups.^3^ To date, various strategies have been developed for the preparation of *gem*-difluoroalkenes, including the conventional ones, such as direct difluoroolefination of carbonyl or diazo groups.^4^ More recently, defluorinative functionalization of trifluoromethyl alkenes has been applied to the synthesis of *gem*-difluoroalkenes.^5^ For example, Hayashi reported a rhodium-catalyzed cross coupling of 1-(trifluoromethyl)alkenes with arylboroxines to access 1,1-difluoroalkenes with C(sp^2^)–C(sp^3^) bond construction.^6^ Molander realized an example of defluorinative alkylation of trifluoromethyl alkenes using radical precursors (potassium organotrifluoroborates, alkylbis(catecholato)silicates and trimethylsilylamines) under photocatalysis conditions.^7^ Despite the great successes achieved, general methods to obtain *gem*-difluoroalkenes with readily available reagents under mild conditions are still required.

**Fig. 1:**
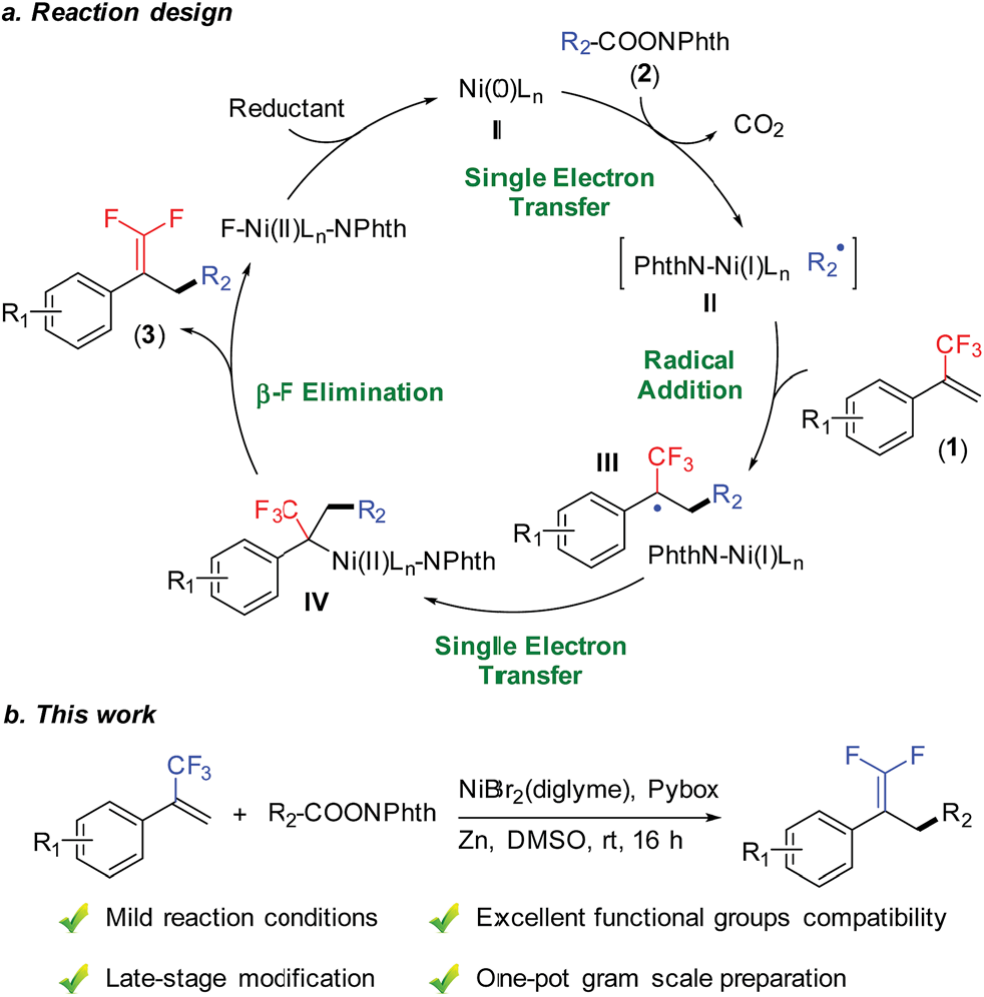
Nickel-catalyzed allylic defluorinative alkylation. NPhth = phthalimide.

Reductive cross-coupling reactions represent a versatile tool for accurate construction of C–C bonds from cheap, abundant, and stable electrophiles as compared with methods using the corresponding organometallic reagents.^8^ Recently, reductive decarboxylative coupling has been exploited for generating alkyl radicals from alkanoic acids, complementing the use of alkyl halides beneficially.^9^ As part of our ongoing interest in alkene functionalization reactions9b,^10^ and fluorinated olefin synthesis,^11^ we set out to take advantage of allylic defluorination and reductive decarboxylation for the radical alkylation of trifluoromethyl alkenes (Fig. 1a).^7^,^12^–^14^ Herein, we report nickel-catalyzed defluorinative reductive cross-coupling of trifluoromethyl alkenes with redox-active esters to access functionalized *gem*-difluoroalkenes (Fig. 1b). This reaction enabled C(sp^3^)–C(sp^3^) bond formation through trifluoromethyl C–F bond cleavage and a decarboxylation process under mild reaction conditions. In terms of practicality and usability, this reaction shows a high degree of tolerance to many sensitive functional groups and requires minimal substrate protection. Therefore it can be a useful method for the synthesis of fluorinated compounds.

## Results and discussion

We commenced the study with the synthesis of *gem*-difluoroalkene **3aa** through defluorinative reductive cross-coupling between **1a** and **2a**. Systematic screening of all the reaction parameters was carried out for optimizing the reaction performance (Table 1). A nickel-bipyridine-reductant system was tested first, affording the desired product in low yields (entries 1 and 2). Use of tridentate N-ligands (entries 3 and 4) led to dramatic improvements: Pybox (**L4**) increased the yield to 79% (entry 4). The selection of the nickel source was critical to this reaction: compared to NiBr_2_(diglyme), only NiCl_2_(Py)_4_ provided 75% yield, while other nickel catalysts were much less effective (entries 5–10). A number of solvents were also examined: ether solvents (entries 11–13), acetonitrile (entry 14), NMP (entry 15), and DMF (entry 16) were inferior. Fortunately, the use of DMSO (entry 17) provided a nearly quantitative 95% GC yield with 92% isolated yield. Because of the formation of observable by-products (see the ESI† for more details), Zn exhibited better efficiency over other reductants such as Mn, silane, or a diboron reagent (entries 18–20).

**Table 1:** Optimization of the reaction conditions^*a*^

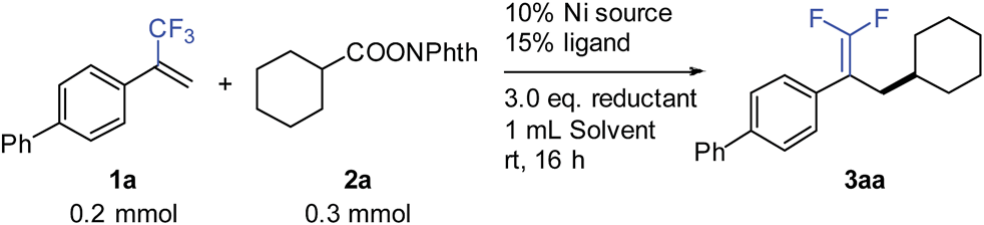					
Entry	Nickel source	Ligand	Reductant	Solvent	Yield^*a*^/%
1	NiBr_2_(diglyme)	**L1**	Zn	DMAc	23
2	NiBr_2_(diglyme)	**L2**	Zn	DMAc	32
3	NiBr_2_(diglyme)	**L3**	Zn	DMAc	47
4	NiBr_2_(diglyme)	**L4**	Zn	DMAc	79
5	NiCl_2_	**L4**	Zn	DMAc	33
6	Ni(NO_3_)_2_	**L4**	Zn	DMAc	<5
7	Ni(acac)_2_	**L4**	Zn	DMAc	26
8	NiCl_2_(Py)_4_	**L4**	Zn	DMAc	75
9	NiCl_2_(PPh_3_)_2_	**L4**	Zn	DMAc	23
10	NiCl_2_(PCy_3_)_2_	**L4**	Zn	DMAc	17
11	NiBr_2_(diglyme)	**L4**	Zn	Dioxane	<5
12	NiBr_2_(diglyme)	**L4**	Zn	DME	22
13	NiBr_2_(diglyme)	**L4**	Zn	THF	43
14	NiBr_2_(diglyme)	**L4**	Zn	MeCN	<5
15	NiBr_2_(diglyme)	**L4**	Zn	NMP	54
16	NiBr_2_(diglyme)	**L4**	Zn	DMF	60
***17***	***NiBr***_***2***_ ***(diglyme)***	***L4***	***Zn***	***DMSO***	***95 (92*** ^*b*^ ***)***
18	NiBr_2_(diglyme)	**L4**	Mn	DMSO	64
19	NiBr_2_(diglyme)	**L4**	DEMS/Na_2_CO_3_	DMSO	18
20	NiBr_2_(diglyme)	**L4**	(BPin)_2_/K_3_PO_4_	DMSO	22
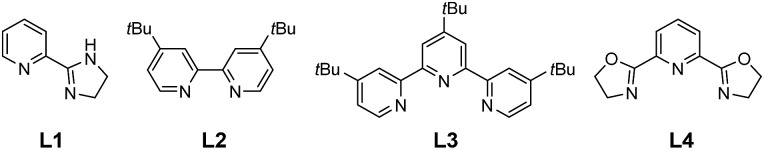					

^*a*^GC yield. Triphenylmethane was used as an internal standard.

^*b*^Isolated yield. rt = room temperature. Diglyme = 2-methoxyethyl ether. acac = Acetylacetone. Py = pyridine. Cy = cyclohexyl. DMAc = *N*,*N*-dimethylacetamide. DME = 1,2-dimethoxyethane. THF = tetrahydrofuran. NMP = 1-methyl-2-pyrrolidinone. DMF = *N*,*N*-dimethylformamide. DMSO = dimethyl sulfoxide. DEMS = diethoxymethylsilane. (BPin)_2_ = bis(pinacolato)diboron.

With the optimal reaction conditions in hand, we sought to examine the generality of this defluorinative reductive cross-coupling by exploring a wide range of redox-active esters (Table 2). These substrates were successfully transformed into the corresponding products, obtaining in all cases good to excellent isolated yields (63–97%). This reaction could be applied to secondary (**3aa**), tertiary (**3ab**), and primary (**3ac**) aliphatic redox-active esters. Both cyclic and acyclic esters were good substrates in this transformation. With respect to cyclic esters, modification of the ring size posed no problem. For example, cyclobutyl (**3ad**), tetrahydrofuran (**3ae**), and adamantyl (**3af**) substrates provided the respective products with nearly quantitative yields. Under mild reaction conditions, this reaction exhibited good compatibility with many synthetically useful functional groups such as carbamate (**3ag**), ketone (**3ah**), and sulfonamide (**3bi**). In addition, heterocycles including furan (**3bj**) and pyridine (**3ak**) were well tolerated. The compatibility to aryl chloride (**3al**) and aryl bromide (**3am**) provided opportunities for convenient transformations at the retained carbon–halogen bonds through the use of other cross-coupling reactions. Moreover, this reaction could even be conducted in the presence of an acidic phenolic hydroxyl group (**3an**).^15^ It is worth mentioning that a number of products (*e.g.*, **3ae**, **3al**, **3ao**, **3ap**, **3aq** and **3ar**) were difficult and even chemically infeasible to prepare through the use of a cross-coupling with the corresponding alkyl halides.^16^ A one-pot synthesis at the gram scale further demonstrated the simplicity and usability of this new method. A representative substrate (**3al**) was conveniently prepared without any prior preparation and isolation of the corresponding redox-active esters (see the ESI† for more details).

**Table 2:** Substrate scope of redox-active esters^*a*^

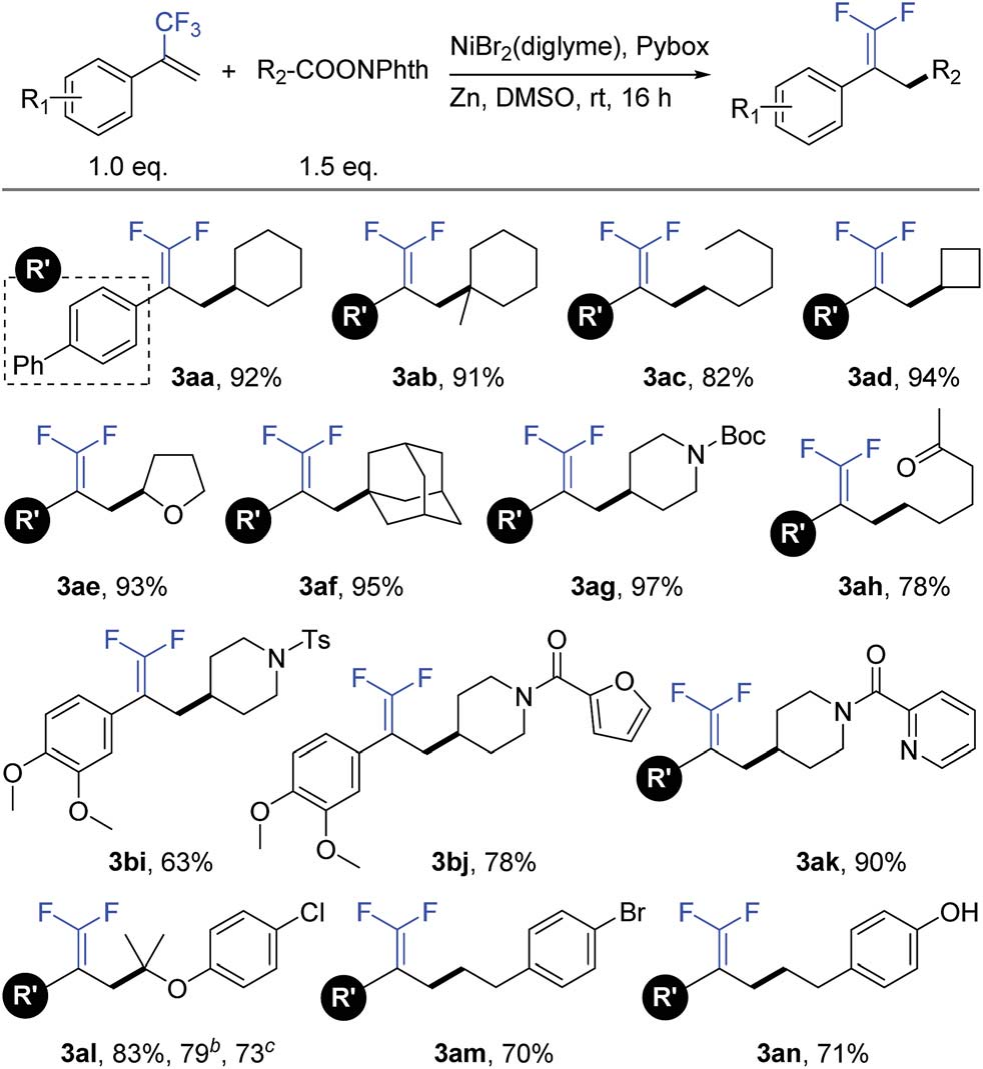

^*a*^Isolated yield for 0.2 mmol scale reaction. Reaction conditions are the same as those for Table 1, entry 17.

^*b*^Isolated yield for 0.2 mmol scale one-pot reaction.

^*c*^Isolated yield for 5.0 mmol scale one-pot reaction. Ratio of desired product/addition by-product >50 : 1 unless otherwise noted. Boc = *tert*-butoxycarbonyl. Ts = tosyl.

We further examined the applicability of this reaction by evaluating the substrate scope of trifluoromethyl alkenes. As shown in Table 3, trifluoromethyl alkenes with different functional groups could be converted to the desired products successfully. For instance, benzyl ether (**3ca**), naphthalene (**3db**), and diphenyl ether (**3eb**) were well tolerated. Heterocycles such as dioxolane (**3fb**), dibenzofuran (**3gb**), morpholine (**3hb**), and piperazine (**3ib**) could be used in this transformation. Moreover, some base-sensitive groups such as acetyl (**3ja**), cyano (**3ka**), and ethoxycarbonyl (**3la**) also survived during the defluorinative reductive cross-coupling process. The tolerance of aryl tosylate (**3mb**) and intramolecular terminal alkene (**3nb**) afforded further functionalization possibilities. Finally, more active groups that have been difficult substrates in transition-metal-catalyzed cross-coupling reactions, such as sulfoether (**3ob**), unprotected phenolic hydroxyl (**3pb**), and primary amine (**3qb**), were compatible with this defluorinative reductive cross-coupling.^17^

**Table 3:** Substrate scope of trifluoromethyl alkenes^*a*^

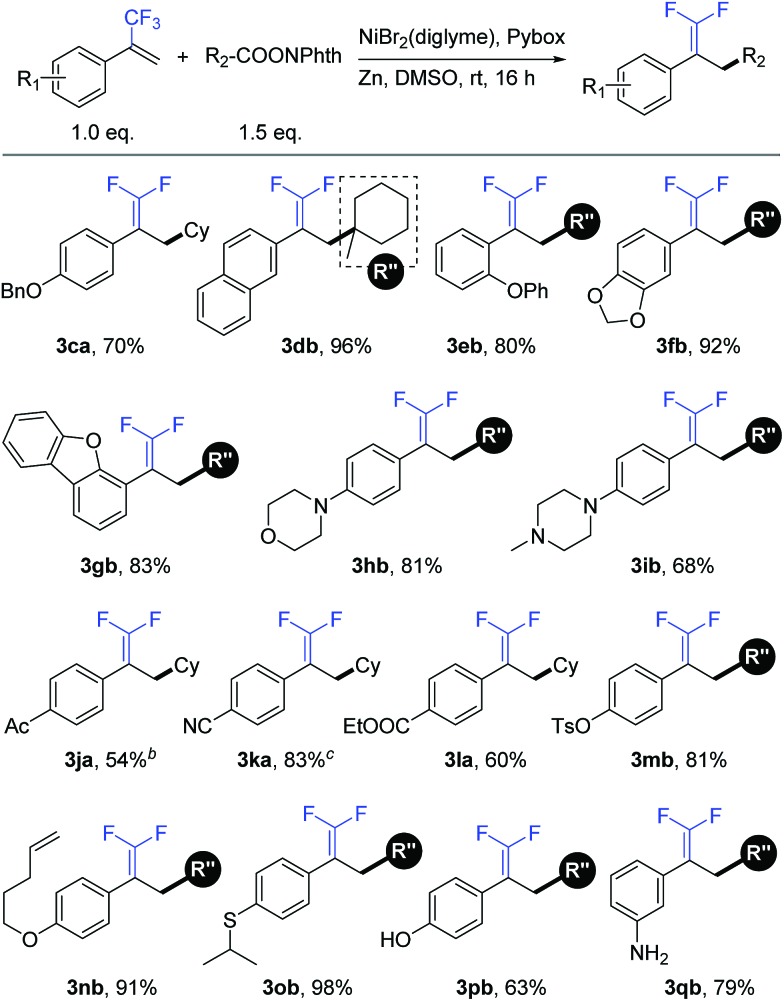

^*a*^Isolated yield for 0.2 mmol scale reaction. Reaction conditions are the same as those for Table 1, entry 17. Ratio of desired product/addition by-product >50 : 1 unless otherwise noted.

^*b*^Ratio of desired product/addition by-product = 14 : 1.

^*c*^Ratio of desired product/addition by-product = 35 : 1. Bn = benzyl. Ac = acetyl.

To further demonstrate the high compatibility of this reaction with diverse functional groups, we exploited its application as an easy-to-use tool for the modification of natural products and drug molecules (Table 4). As an illustration, lithocholic acid derivative **2o** smoothly reacted with **1a** to afford the desired product **3ao** with 74% isolated yield. Another example is of dehydrocholic acid ester **2p** containing three base-sensitive ketone groups, which performed well during this modification process. In the modification of more complex gibberellic acid ester **2q**, the desired product **3aq** was obtained in 22% yield despite the presence of an ester group, internal and terminal alkenes, and unprotected secondary and tertiary alcohol groups in the reactant. Modification of a niflumic acid derivative **1r** produced the corresponding product **3ra** while tolerating the ester group, pyridine ring, and secondary amine. Indometacin derivative **1s** could react with **2a** to provide product **3sa** in 68% yield, without affecting either the indole ring or aryl chloride. Finally, fructose derivative **1t** was also a good substrate and afforded product **3ta** with a satisfactory 75% isolated yield. Therefore, this defluorinative reductive cross-coupling presents an attractive opportunity for late-stage protecting-group-free modification of biologically interesting molecules.

**Table 4:** Modification of natural products and drug molecules^*a*^

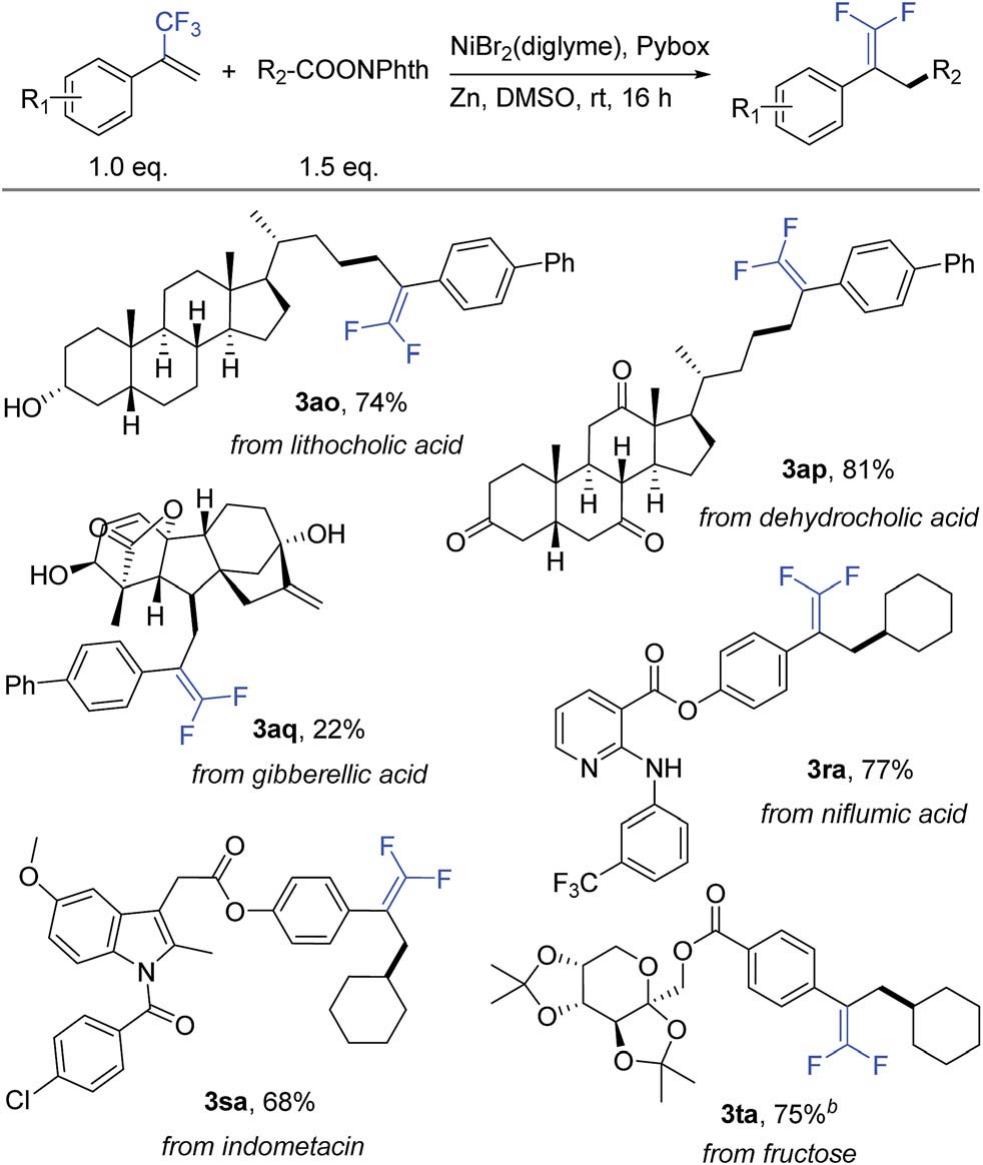

^*a*^Isolated yield for 0.2 mmol scale reaction. Reaction conditions are the same as those for Table 1, entry 17. Ratio of desired product/addition by-product >50 : 1 unless otherwise noted.

^*b*^Ratio of desired product/addition by-product = 7 : 1.

Similar to our previous studies,9b,10a,11a we herein show that this allylic defluorinative alkylation reaction could be applied to alkyl halides (Table 5), which perhaps less surprising is also practical. Several sensitive functional groups were examined, such as thiophene (**5ba**), cyano (**5bb**), aldehyde (**5bc**), and phenolic hydroxyl (**5bd**), and good to excellent yields were obtained in all cases.

**Table 5:** Expansion to alkyl halides^*a*^

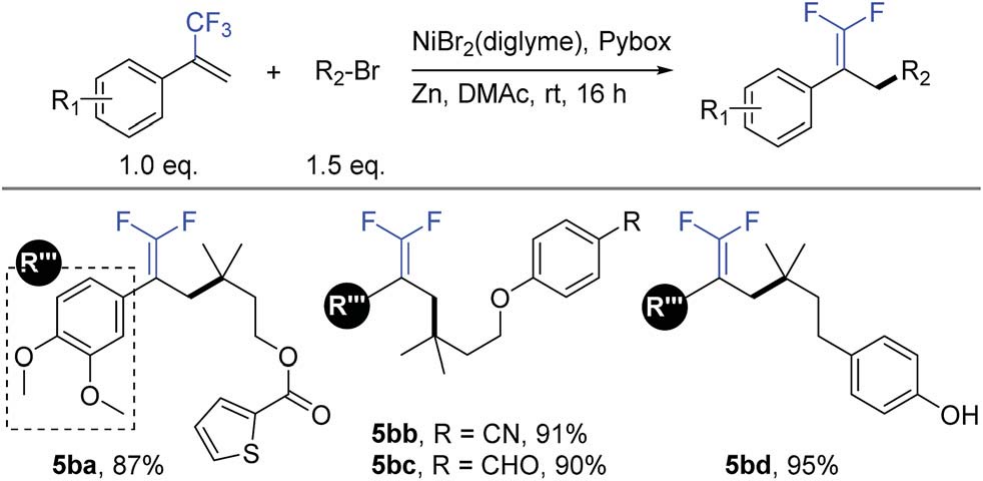

^*a*^Isolated yield for 0.2 mmol scale reaction. Reaction conditions: trifluoromethyl alkenes (1.0 eq.), alkyl halides (1.5 eq.), NiBr_2_(diglyme) (10%), Pybox (15%), Zn (3.0 eq.), DMAc (0.2 M), rt, 16 h. Ratio of desired product/addition by-product >50 : 1 unless otherwise noted.

**Scheme 1:**
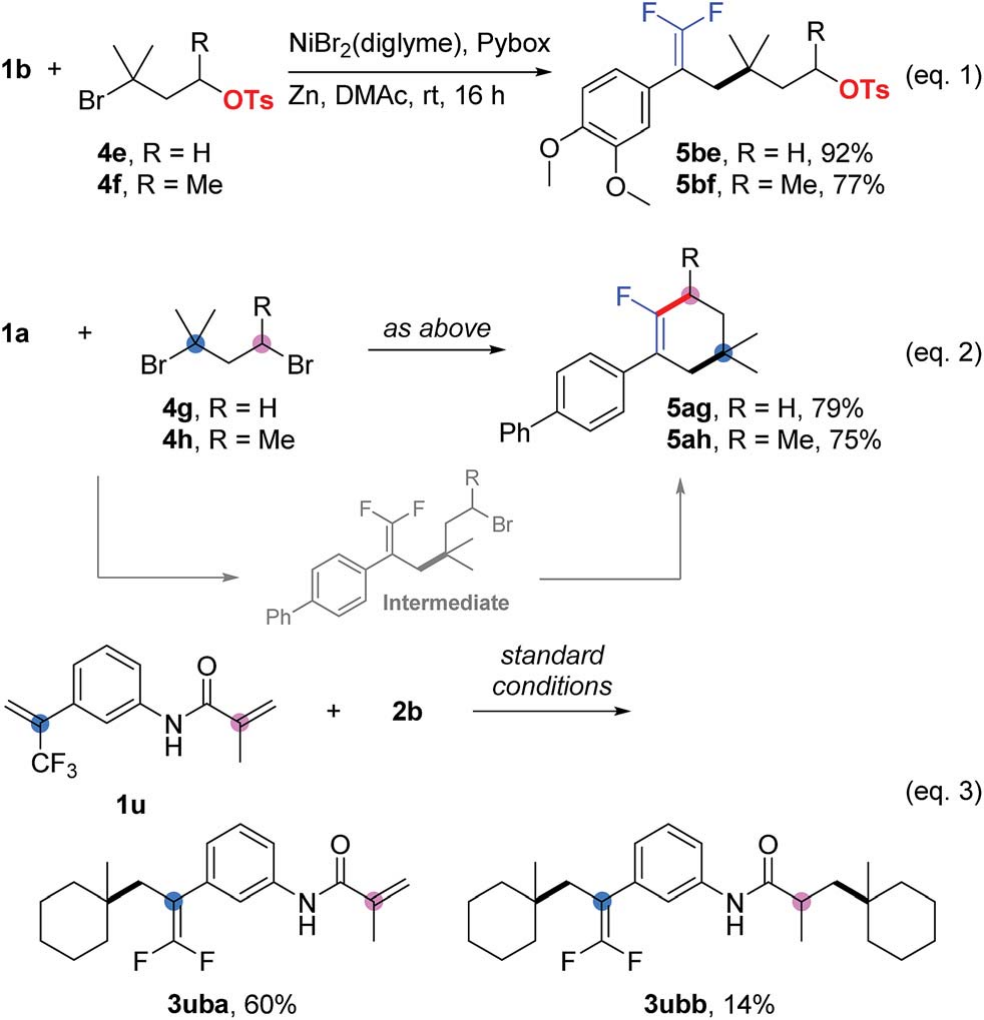
Competition experiments. Isolated yield for 0.2 mmol scale reaction. Reaction conditions for eqn (1) and eqn (2) are the same as those for Table 5. Reaction conditions for eqn (3) are the same as those for Table 1, entry 17. Ratio of desired product/addition by-product >50 : 1 unless otherwise noted.

In competition experiments, tertiary alkyl electrophiles exhibited better reactivity than both primary and secondary ones. For instance, we obtained **5be** and **5bf** as the sole products (Scheme 1, eqn (1)), in which carbon–carbon bonds were formed at the tertiary alkyl bromide sites, while the primary and secondary alkyl sulfonates survived. Interesting results were obtained for the substrates (**5ag** and **5ah**) containing tertiary and primary or secondary alkyl bromides (Scheme 1, eqn (2)). Cyclization products (as the sole product for **5ag** and the main product for **5ah**) were generated firstly through allylic defluorinative alkylation of the tertiary alkyl bromide and then intramolecular cyclization at the primary or secondary sites.^18^ Finally, using a trifluoromethyl alkene containing an acrylamide (**1u**) provided a mixture of mono-alkylation (**3uba**, defluorinative alkylation) and di-alkylation (**3ubb**, defluorinative alkylation and Giese addition) products (Scheme 1, eqn (3)).^19^

**Scheme 2:**
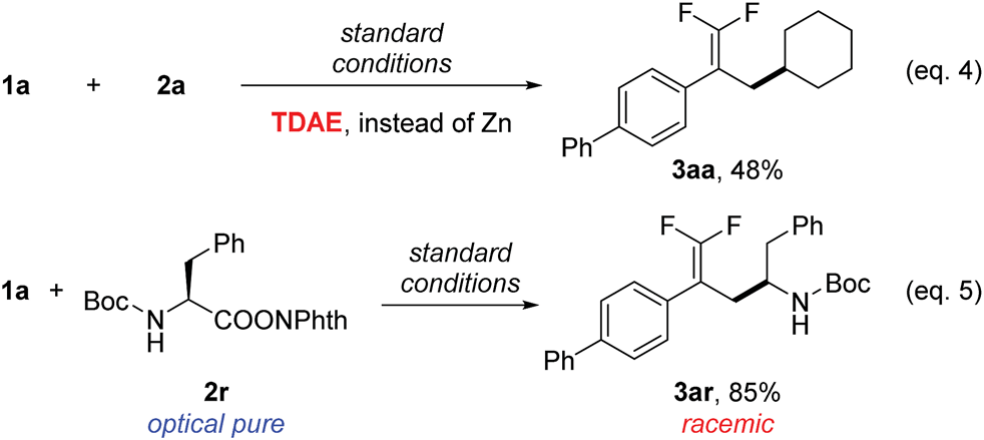
Mechanistic probes. Isolated yield for 0.2 mmol scale reaction. Reaction conditions are the same as those for Table 1, entry 17. Ratio of desired product/addition by-product >50 : 1 unless otherwise noted. TDAE = 1,1,2,2-tetrakis(dimethylamino)ethylene.

To examine the reaction mechanism, the nonmetallic reducing agent TDAE was used to replace Zn(0), which provided an appreciable amount of product and revealed that the activation of redox-active esters might proceed through a single-electron-transfer (SET) process rather than *in situ* formation of organozinc reagents (Scheme 2, eqn (4)).^20^ An optically pure redox-active ester (**1r**) was used to study the stereochemistry, which led to a racemic product (**3ar**) in 85% isolated yield (Scheme 2, eqn (5)). Collectively, the above results supported a radical-type reaction mechanism for this defluorinative reductive cross-coupling.^21^

## Conclusions

We developed a nickel-catalyzed defluorinative reductive cross-coupling of trifluoromethyl alkenes with redox-active esters. This reaction enables convenient and efficient preparation of *gem*-difluoroalkenes through C(sp^3^)–F bond cleavage and C(sp^3^)–C(sp^3^) bond formation. Under mild reaction conditions, many sensitive functional groups were tolerated, therefore providing a robust approach for late-stage protecting-group-free modification of natural products or drug molecules. A one-pot synthesis at the gram scale further demonstrated the usability and applicability of this new method. Preliminary mechanistic studies suggested a nickel-catalyzed radical-type process. Our next challenge is the extension of the reaction to an asymmetric version.

## Conflicts of interest

The authors declare no competing interests.

## Supplementary Material

Supplementary information
