# Supplementary material for: Nickel-catalyzed allylic defluorinative alkylation of trifluoromethyl alkenes with reductive decarboxylation of redox-active esters
Source: Chem Sci. 2018 Nov 7;10(3):809–14. doi: 10.1039/c8sc04335c (PMC6345349; doi:10.1039/c8sc04335c)
Supplement: Supplementary file 1 [file SC-010-C8SC04335C-s001.pdf]

Electronic Supplementary Material (ESI) for Chemical Science.
This journal is © The Royal Society of Chemistry 2018

Supplementary Information

Nickel-Catalyzed Allylic Defluorinative Alkylation of Trifluoromethyl Alkenes with Reductive Decarboxylation of Redox-Active Esters

Xi Lu,*,† Xiao-Xu Wang,† Tian-Jun Gong, Jing-Jing Pi, Shi-Jiang He, Yao Fu*

† These authors contributed equally.

Hefei National Laboratory for Physical Sciences at the Microscale, CAS Key Laboratory of Urban Pollutant Conversion, Anhui Province Key Laboratory of Biomass Clean Energy, *i*ChEM, University of Science and Technology of China, Hefei 230026, China

Table of contents

1. General Information S3

1.1. Materials S3

1.2. Analytical Methods S3

2. Preparation of Substrates S4

2.1. Preparation of Trifluoromethyl Alkenes S4

2.2. Preparation of NHPI Esters S37

2.3. Preparation of Alkyl Bromides S39

3. General Procedure for Table 1 .... S41

4. General Procedure for the Defluorinative Reductive Cross-Coupling S43

5. Examples Described in Table 2 .... S44

6. One-Pot Synthesis at Gram Scale S80

7. Examples Described in Table 3 .... S81

8. Examples Described in Table 4 .... S121

9. General Procedure for Examples Described in Table 5 .... S139

10. Examples Described in Table 5 .... S140

11. General Procedure for Examples Described in Scheme 1 S151

12. Examples Described in Scheme 1 .... S152

13. Examples Described in Scheme 2 .... S168

14. References S171

1. General Information

1.1. Materials

The following chemicals were purchased and used as received: nickel(II) bromide 2-methoxyethyl ether complex (CAS: 312696-09-6, Aldrich, 459674-5G); zinc, 98+%, dust (CAS: 7440-66-6, Acros Organics, 198340010); dimethyl sulfoxide (CAS: 67-68-5, Adamas-beta, 75927M); 1,3-dibromo-3-methylbutane (CAS: 24443-15-0, Alfa Aesar, B20468.06).

2,6-Bis(4,5-dihydrooxazol-2-yl)pyridine (Pybox) was synthesized according to the reported literature.[1]

1.2. Analytical Methods

^{1}H NMR, ^{13}C NMR and ^{19}F NMR spectra were recorded on a Bruker 400 MHz spectrometer at 295 K in $CDCl_3$ unless otherwise noted. Data for ^{1}H NMR were reported as follows: chemical shift (δ ppm), multiplicity, coupling constant (Hz), and integration. Data for ^{13}C NMR were reported as follows: chemical shift (δ ppm), multiplicity, and coupling constant (Hz). Data for ^{19}F NMR were reported as follows: chemical shift (δ ppm), multiplicity, coupling constant (Hz). Chemical shifts were reported using the residual solvent $CHCl_3$ as the internal reference for ^{1}H NMR (δ = 7.260 ppm) and $CDCl_3$ peak as the internal reference for ^{13}C NMR (δ = 77.160 ppm). Gas chromatographic (GC) analysis was acquired on a Shimadzu GC-2010 plus Series GC system equipped with a flame-ionization detector. Organic solutions were concentrated under reduced pressure on Buchi rotary evaporator. Column chromatographic purification of products was accomplished using forced-flow chromatography on Silica Gel (300-400 mesh).

2. Preparation of Substrates

2.1. Preparation of Trifluoromethyl Alkenes

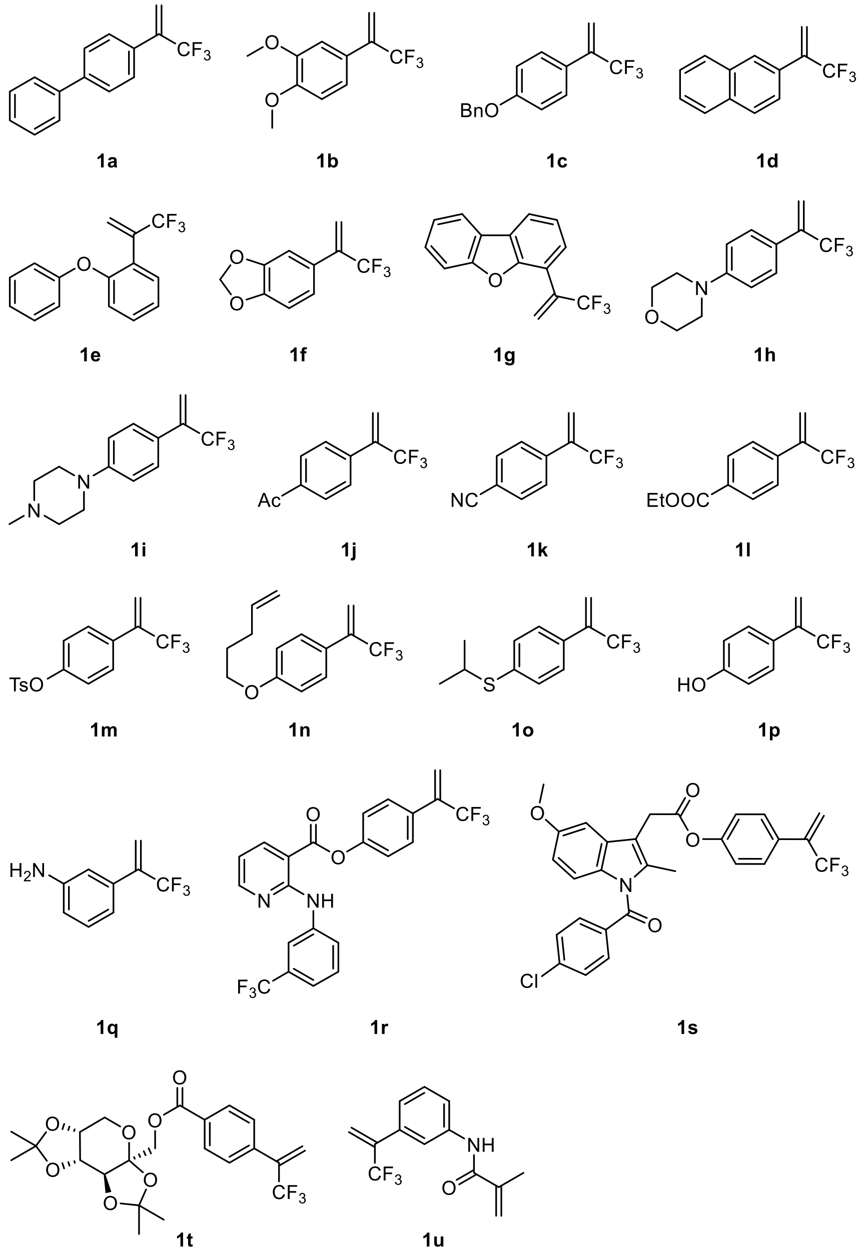

List of trifluoromethyl alkenes.

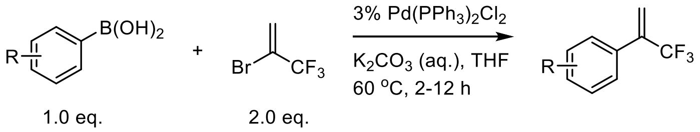

Preparation of trifluoromethyl alkenes using arylboronic acids.

According to the reported literature, trifluoromethyl alkenes (**1a-1l**, **1o**, **1q**, **1u**) were conveniently synthesized in gram scale under slightly modified reaction conditions.[2] In a Schlenk tube equipped with stir bar, arylboronic acids (1.0 equiv., 10 mmol) and $Pd(PPh_3)_2Cl_2$ (3 mol%, 0.3 mmol, 210.6 mg) were added. The vessel was evacuated and filled with argon (three cycles), then aqueous K_2CO_3 (2.0 M, 20 mL) and THF (30 mL) were added. After the addition of 2-bromo-3,3,3-trifluoropropene (2.0 equiv., 20 mmol, 2.1 mL), the solution was stirred at 60 °C for 2-12 hours (TLC tracking detection). The mixture was purified by column chromatography to afford the corresponding trifluoromethyl alkenes. **1a**,[3] **1b**,[4] **1c**,[4] **1d**,[3] **1f**,[4] were known compounds, and the data match the reported.

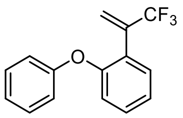

1-phenoxy-2-(3,3,3-trifluoroprop-1-en-2-yl)benzene (**1e**)

<u>^{1}H NMR (400 MHz, Chloroform-*d*)</u> δ 7.39 – 7.28 (m, 4H), 7.16 – 7.05 (m, 2H), 6.98 – 6.88 (m, 3H), 6.10 – 6.03 (m, 1H), 5.71 (s, 1H).

<u>^{13}C NMR (101 MHz, Chloroform-*d*)</u> δ 157.37, 155.04, 135.16 (q, J = 31.6 Hz), 131.09, 130.41, 129.87, 126.02, 124.01 (q, J = 5.2 Hz), 123.44, 123.38, 123.15 (q, J = 274.9 Hz), 119.44, 118.71.

<u>^{19}F NMR (376 MHz, Chloroform-*d*)</u> δ -65.61.

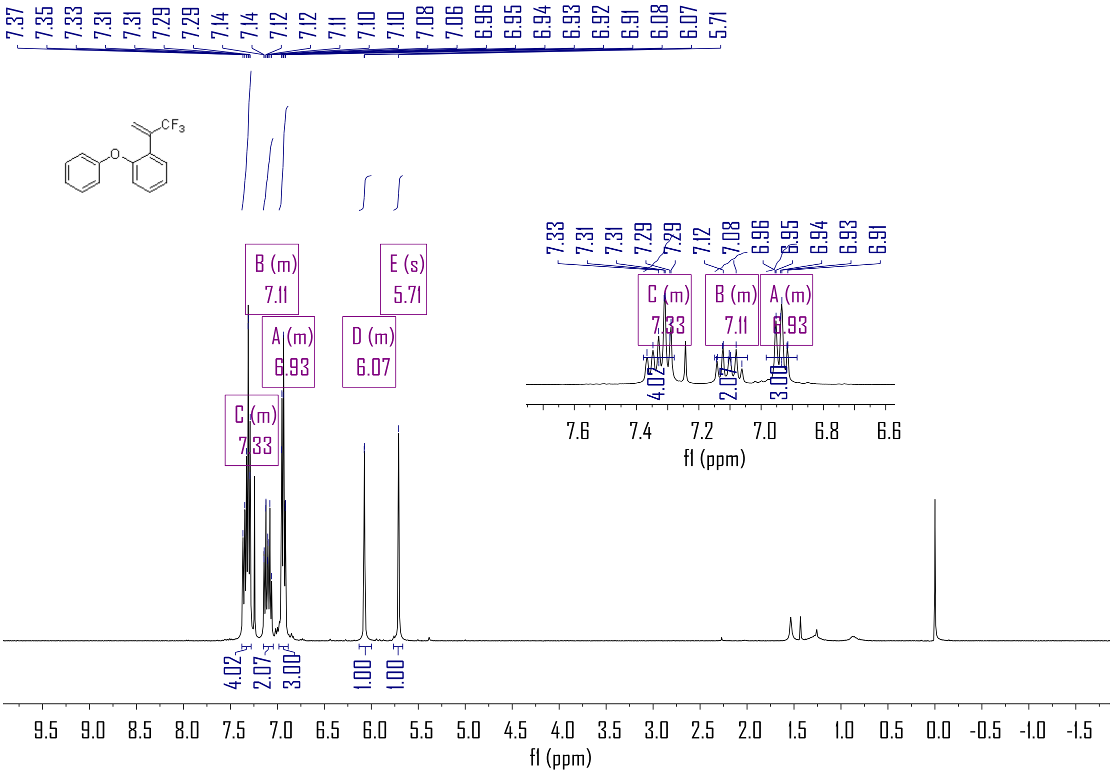

^{1}H NMR spectra for **1e**.

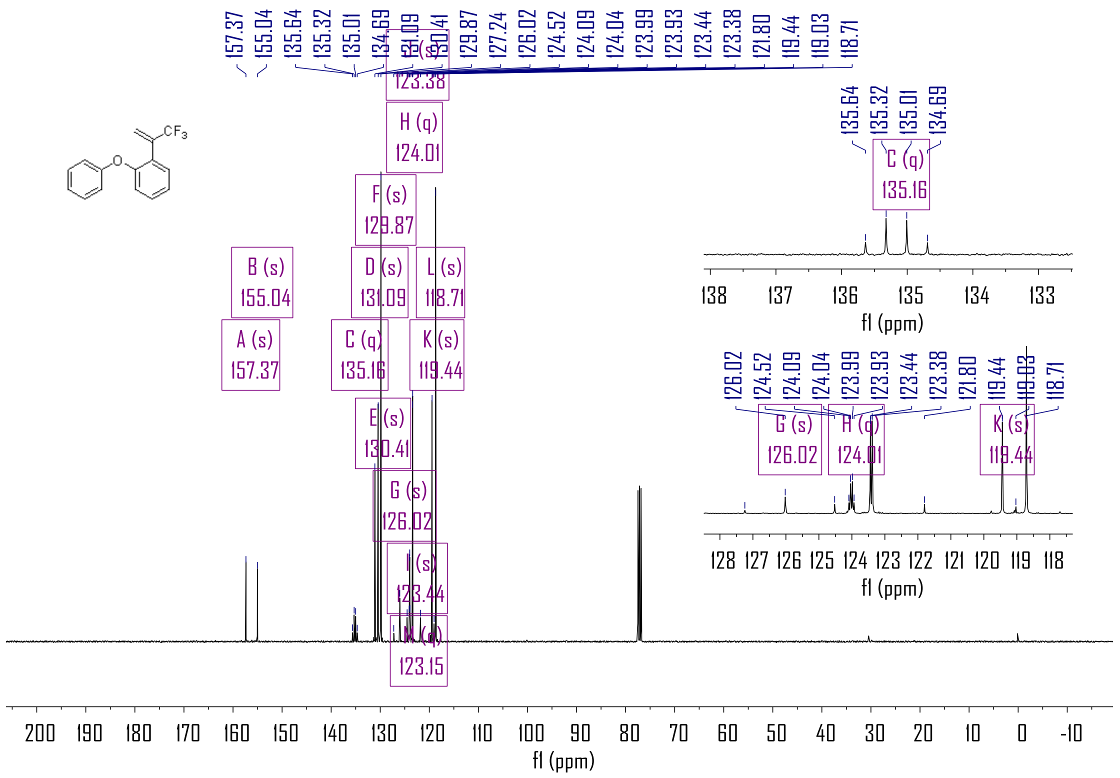

^{13}C NMR spectra for **1e**.

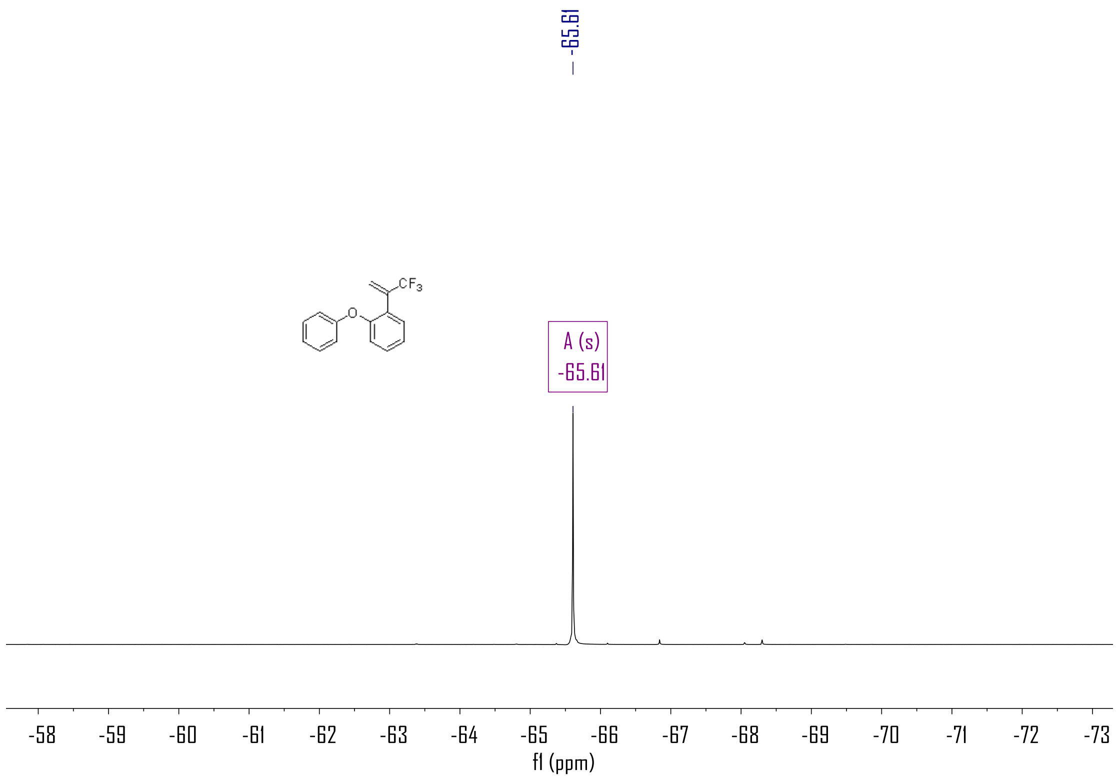

^{19}F NMR spectra for **1e**.

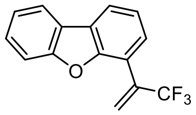

4-(3,3,3-trifluoroprop-1-en-2-yl)dibenzo[*b*,*d*]furan (**1g**)

^{1}H NMR (400 MHz, Chloroform-*d*) δ 8.02 – 7.93 (m, 2H), 7.63 – 7.59 (m, 1H), 7.56 – 7.46 (m, 2H), 7.41 – 7.34 (m, 2H), 6.41 – 6.35 (m, 2H).

^{13}C NMR (101 MHz, Chloroform-*d*) δ 156.12, 153.90, 133.48 (q, *J* = 30.9 Hz), 127.65, 126.69 (q, *J* = 1.5 Hz), 125.17, 124.27 (q, *J* = 5.7 Hz), 123.96, 123.28 (q, *J* = 274.1 Hz), 123.19, 122.84, 121.35, 120.83, 118.16, 111.93.

^{19}F NMR (376 MHz, Chloroform-*d*) δ -64.79 (dd, *J* = 2.2 Hz).

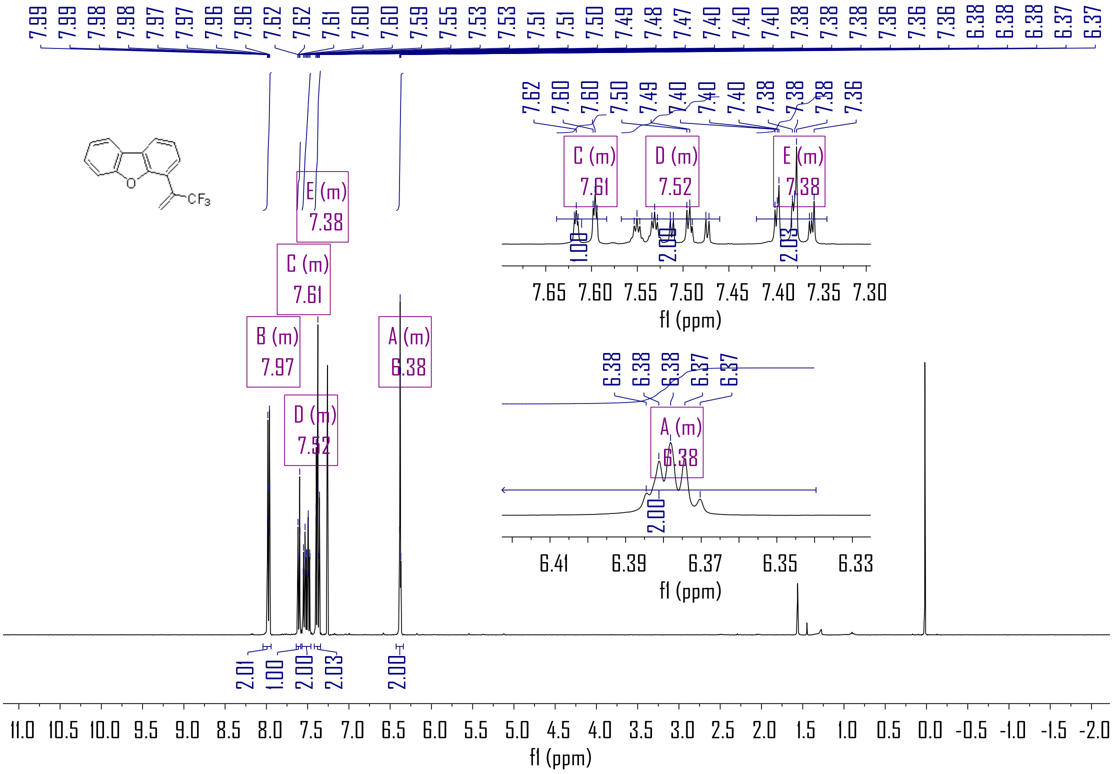

^{1}H NMR spectra for **1g**.

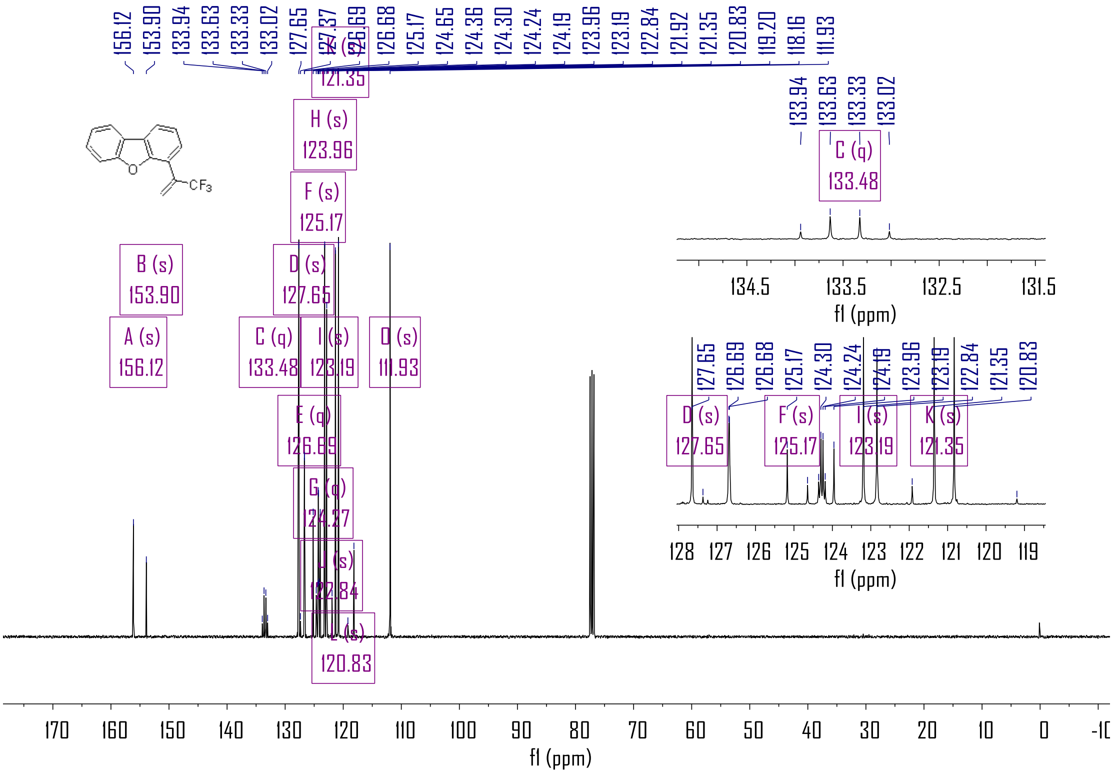

^{13}C NMR spectra for **1g**.

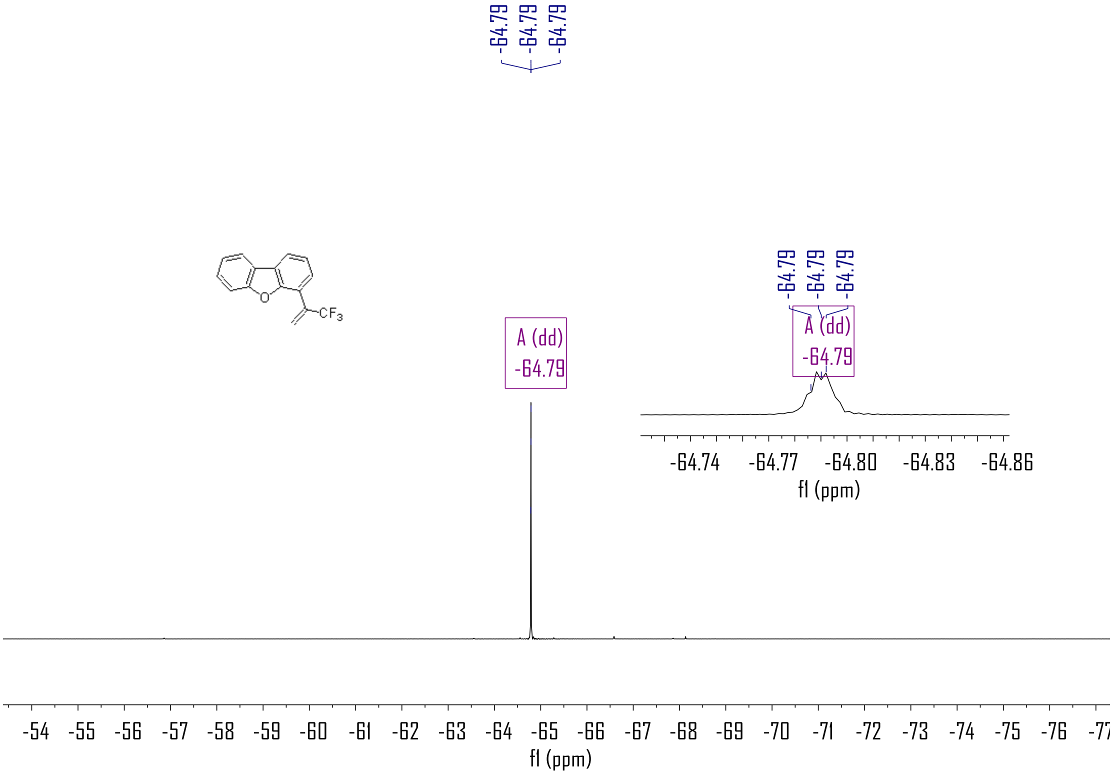

^{19}F NMR spectra for **1g**.

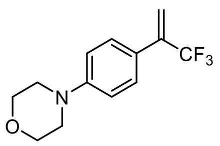

4-(4-(3,3,3-trifluoroprop-1-en-2-yl)phenyl)morpholine (**1h**)

^{1}H NMR (400 MHz, Chloroform-*d*) δ 7.45 – 7.35 (m, 2H), 6.96 – 6.84 (m, 2H), 5.83 (q, *J* = 1.3 Hz, 1H), 5.69 (q, *J* = 1.7 Hz, 1H), 4.01 – 3.78 (m, 4H), 3.47 – 3.03 (m, 4H).

^{13}C NMR (101 MHz, Chloroform-*d*) δ 151.51, 138.36 (q, *J* = 29.6 Hz), 128.31, 124.77, 123.68 (q, *J* = 274.2 Hz), 118.15 (q, *J* = 5.8 Hz), 115.12, 66.87, 48.78.

^{19}F NMR (376 MHz, Chloroform-*d*) δ -64.66.

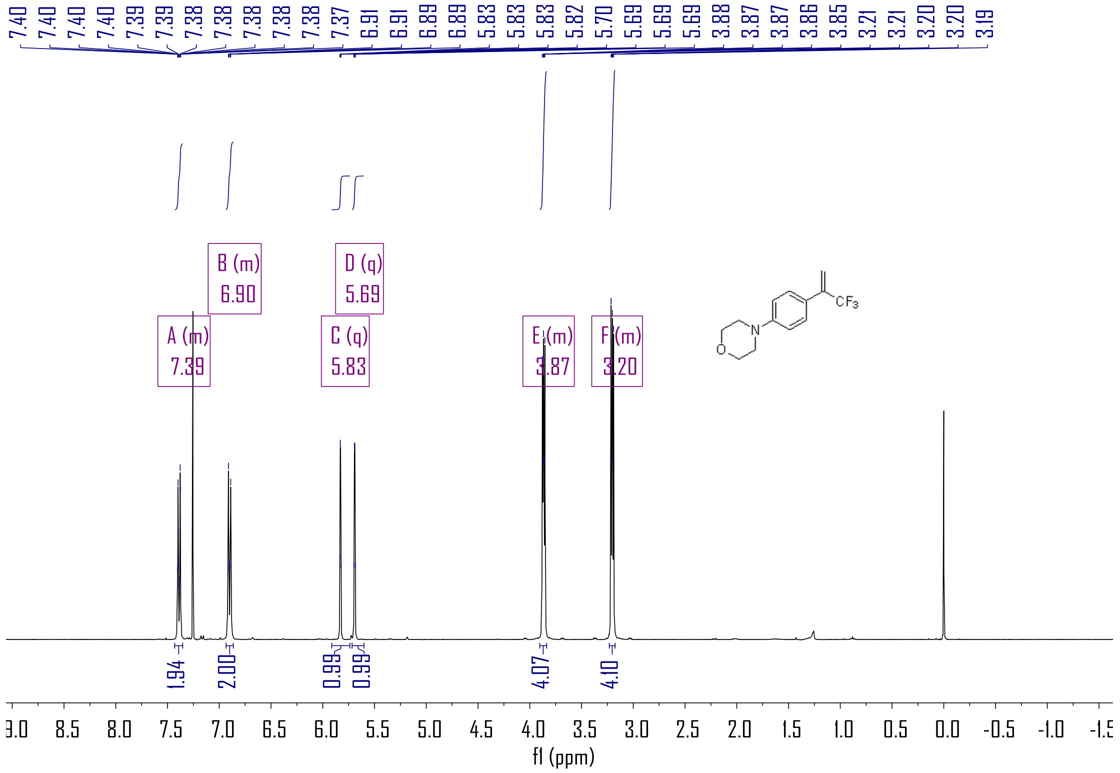

^{1}H NMR spectra for **1h**.

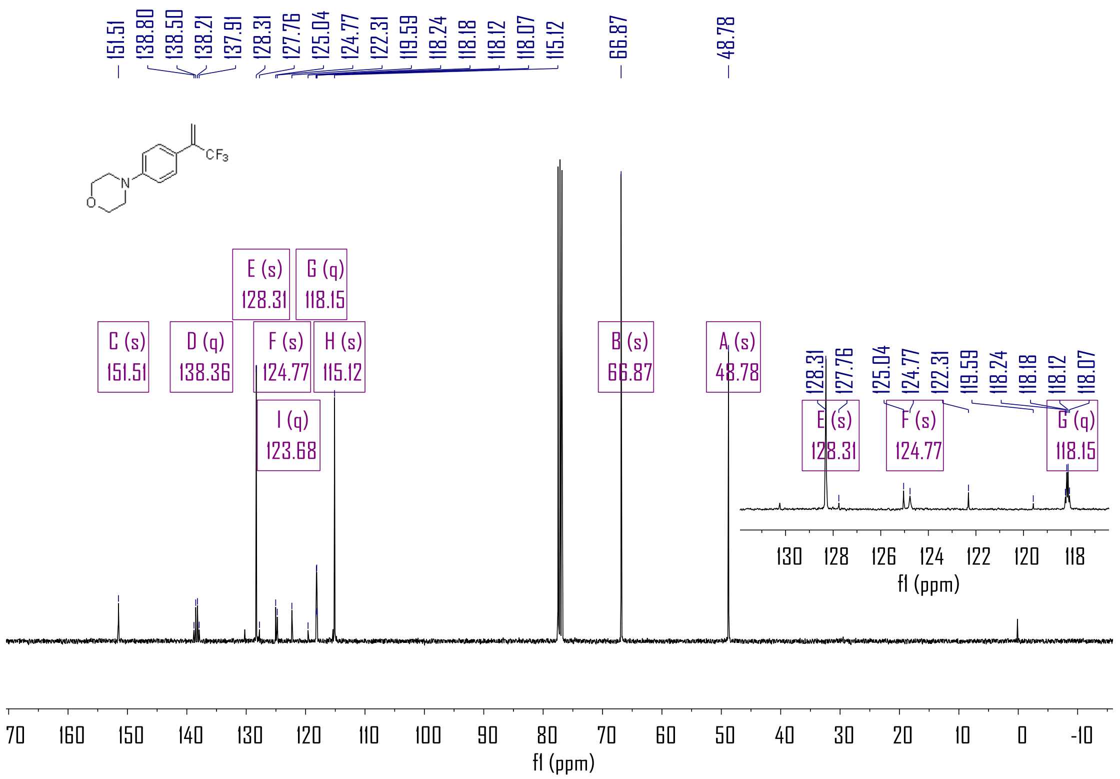

^{13}C NMR spectra for **1h**.

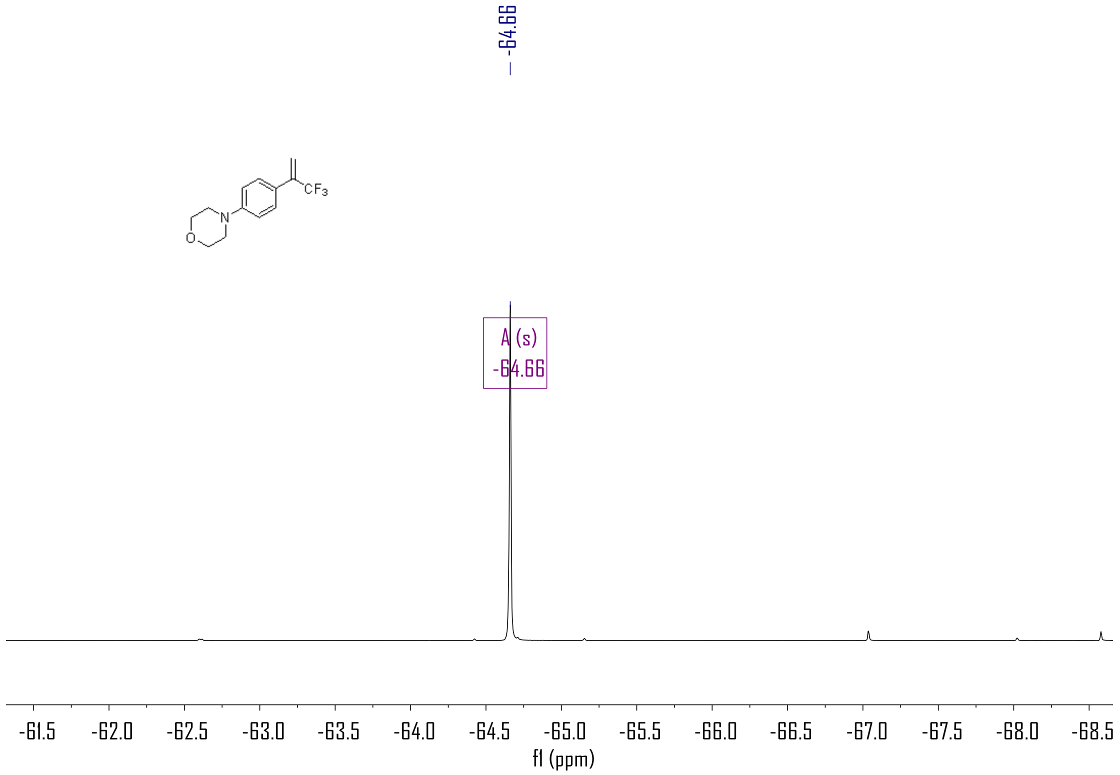

^{19}F NMR spectra for **1h**.

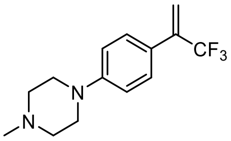

1-methyl-4-(4-(3,3,3-trifluoroprop-1-en-2-yl)phenyl)piperazine (**1i**)

^{1}H NMR (400 MHz, Chloroform-*d*) δ 7.37 (d, *J* = 8.4 Hz, 2H), 6.94 – 6.86 (m, 2H), 5.82 (q, *J* = 1.3 Hz, 1H), 5.68 (q, *J* = 1.8 Hz, 1H), 3.33 – 3.26 (m, 4H), 2.68 – 2.59 (m, 4H), 2.40 (s, 3H).

^{13}C NMR (101 MHz, Chloroform-*d*) δ 151.52, 138.39 (q, *J* = 29.6 Hz), 128.21 (q, *J* = 1.3 Hz), 124.19, 123.70 (q, *J* = 274.1 Hz), 117.86 (q, *J* = 5.9 Hz), 115.30, 55.05, 48.40, 46.23.

^{19}F NMR (376 MHz, Chloroform-*d*) δ -64.65.

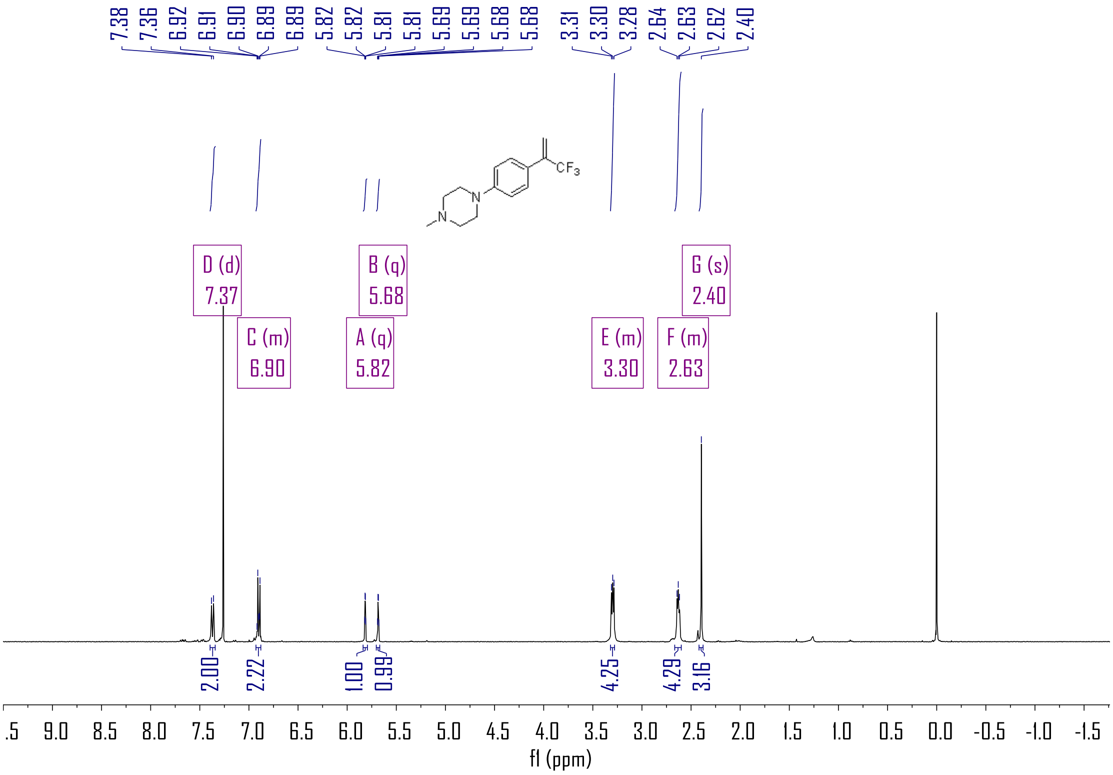

^{1}H NMR spectra for **1i**.

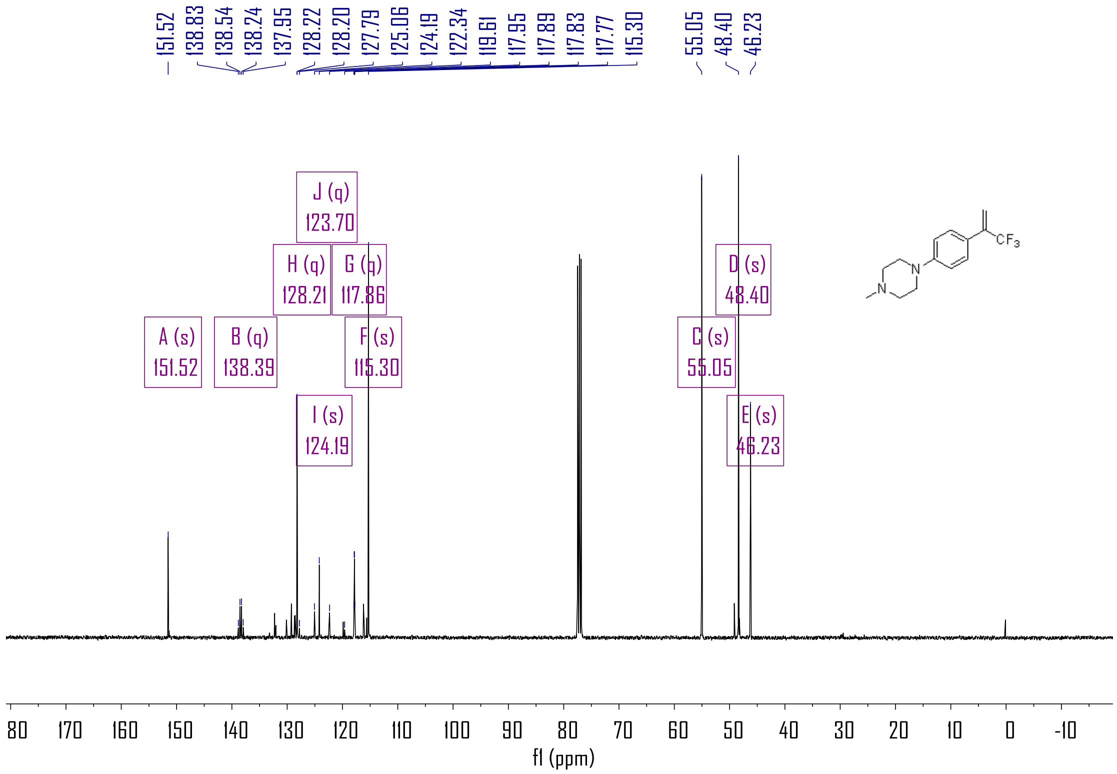

^{13}C NMR spectra for **1i**.

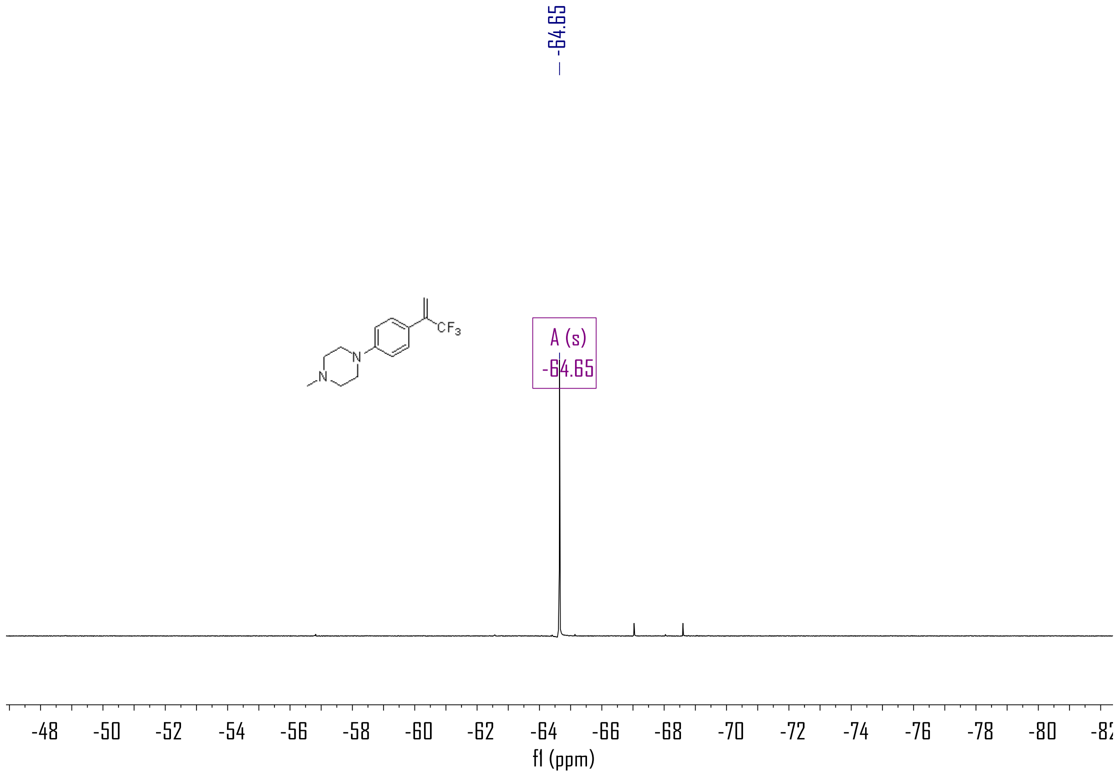

^{19}F NMR spectra for **1i**.

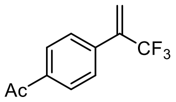

1-(4-(3,3,3-trifluoroprop-1-en-2-yl)phenyl)ethan-1-one (**1j**)

<u>^{1}H NMR (400 MHz, Chloroform-*d*)</u> δ 8.00 – 7.92 (m, 2H), 7.61 – 7.49 (m, 2H), 6.05 (q, *J* = 1.4 Hz, 1H), 5.87 (q, *J* = 1.6 Hz, 1H), 2.61 (s, 3H).

<u>^{13}C NMR (101 MHz, Chloroform-*d*)</u> δ 197.51, 138.28 (q, *J* = 30.5 Hz), 138.05, 137.34, 128.66, 127.68 (q, *J* = 1.1 Hz), 123.15 (q, *J* = 274.0 Hz), 122.07 (q, *J* = 5.7 Hz), 26.74.

<u>^{19}F NMR (376 MHz, Chloroform-d)</u> δ -64.64.

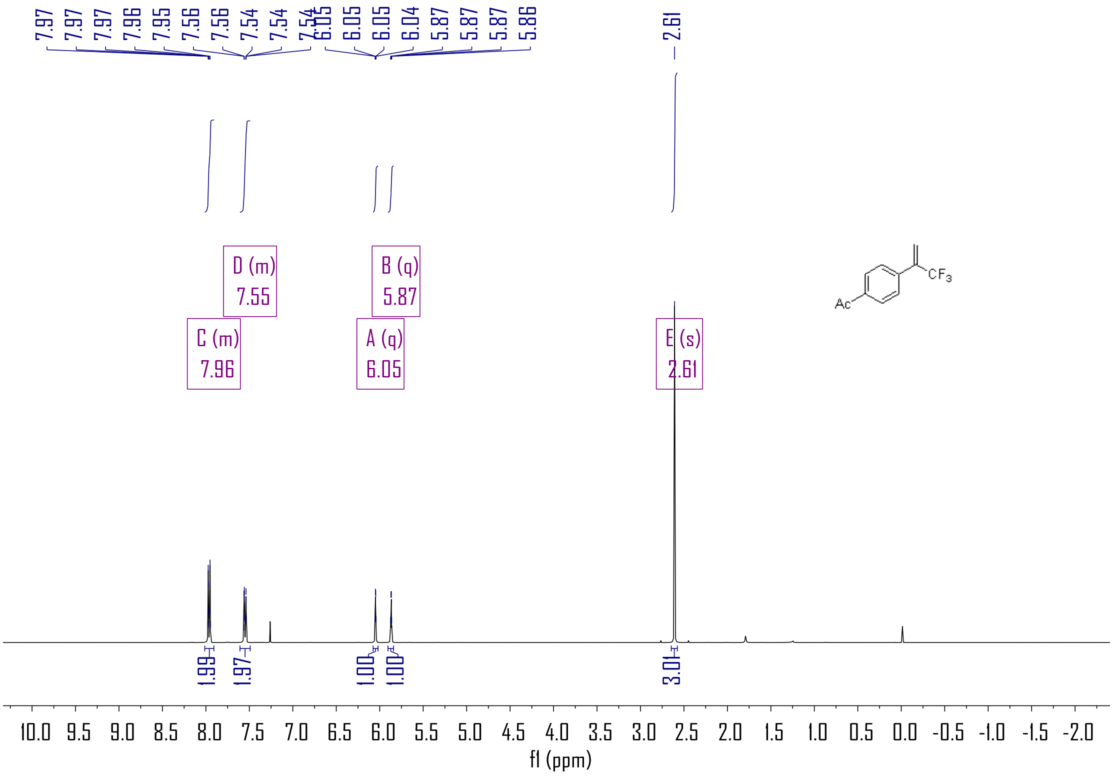

^{1}H NMR spectra for **1j**.

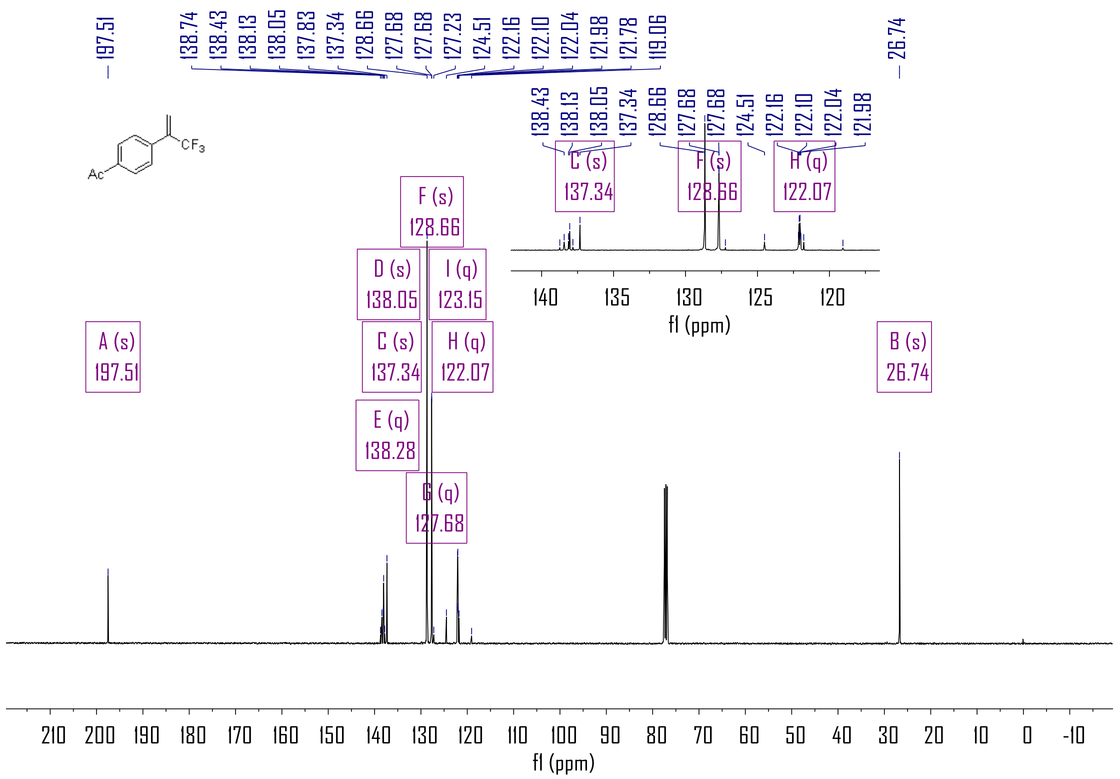

^{13}C NMR spectra for **1j**.

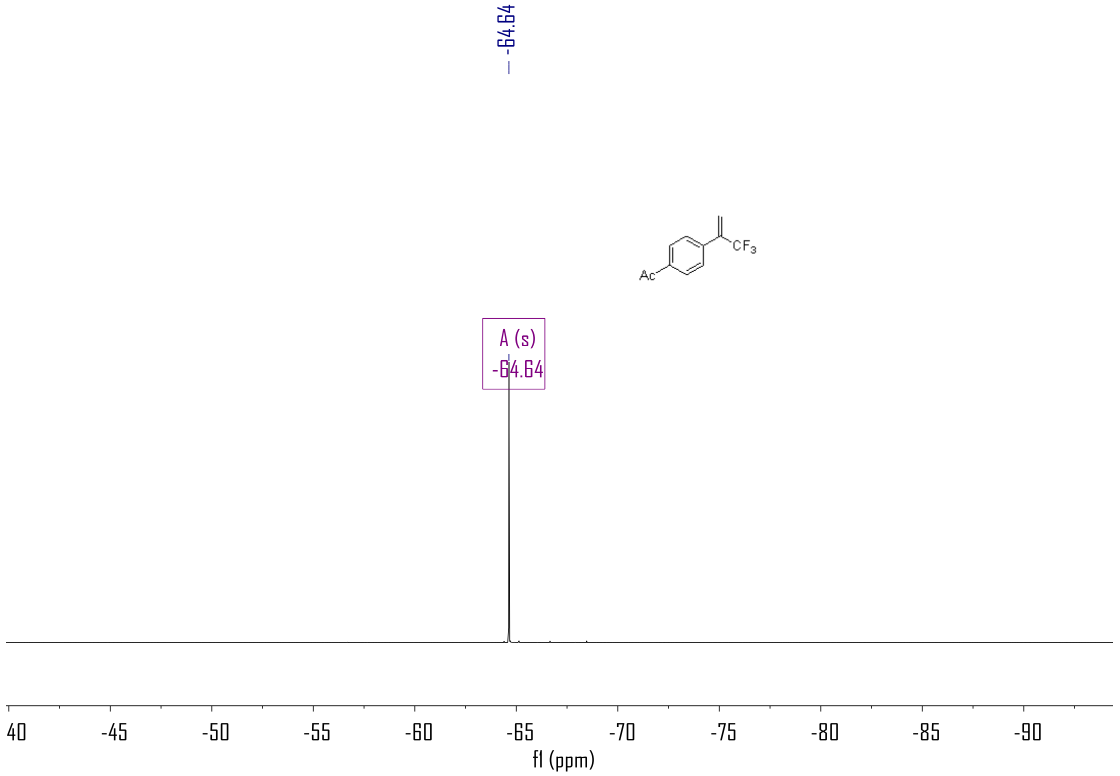

^{19}F NMR spectra for **1j**.

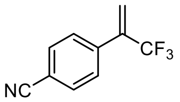

4-(3,3,3-trifluoroprop-1-en-2-yl)benzonitrile (**1k**)

^{1}H NMR (400 MHz, Chloroform-*d*) δ 7.73 – 7.68 (m, 2H), 7.58 (d, J = 8.2 Hz, 2H), 6.11 (q, J = 1.5 Hz, 1H), 5.90 (q, J = 1.7 Hz, 1H).

^{13}C NMR (101 MHz, Chloroform-*d*) δ 137.98, 137.75 (q, J = 30.8 Hz), 132.48, 128.18 (q, J = 1.1 Hz), 122.95 (q, J = 5.7 Hz), 122.90 (q, J = 273.9 Hz), 118.34, 113.00.

^{19}F NMR (376 MHz, Chloroform-*d*) δ -64.68.

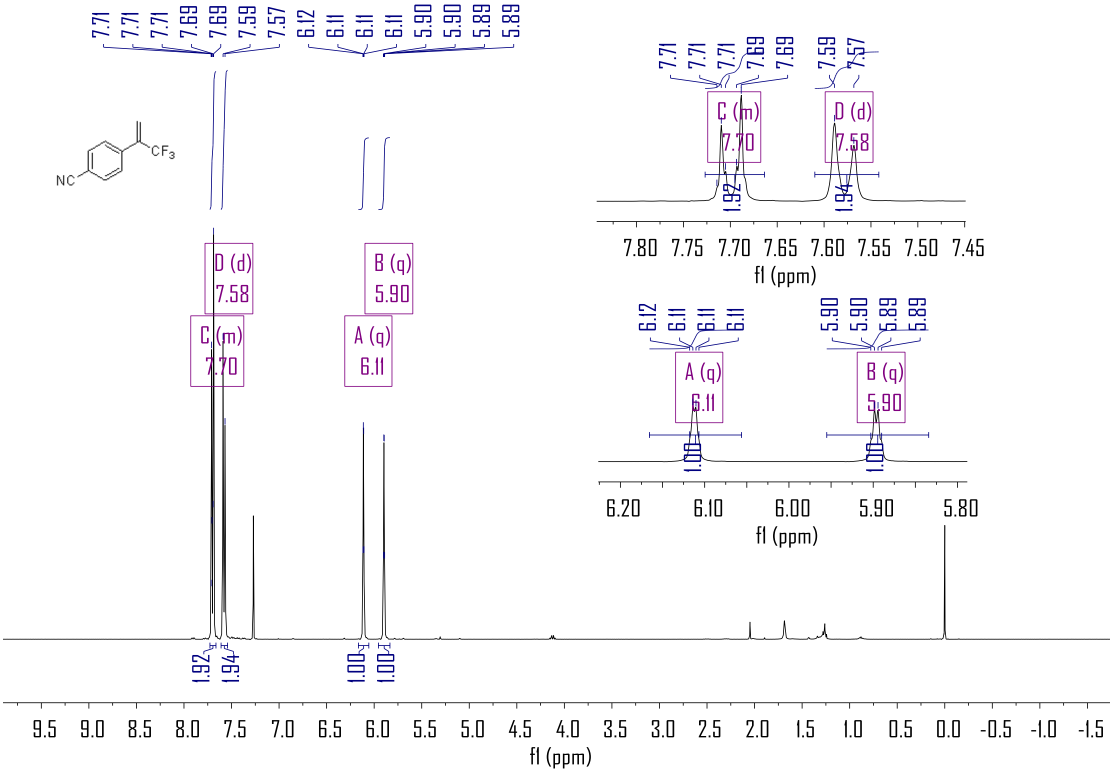

^{1}H NMR spectra for **1k**.

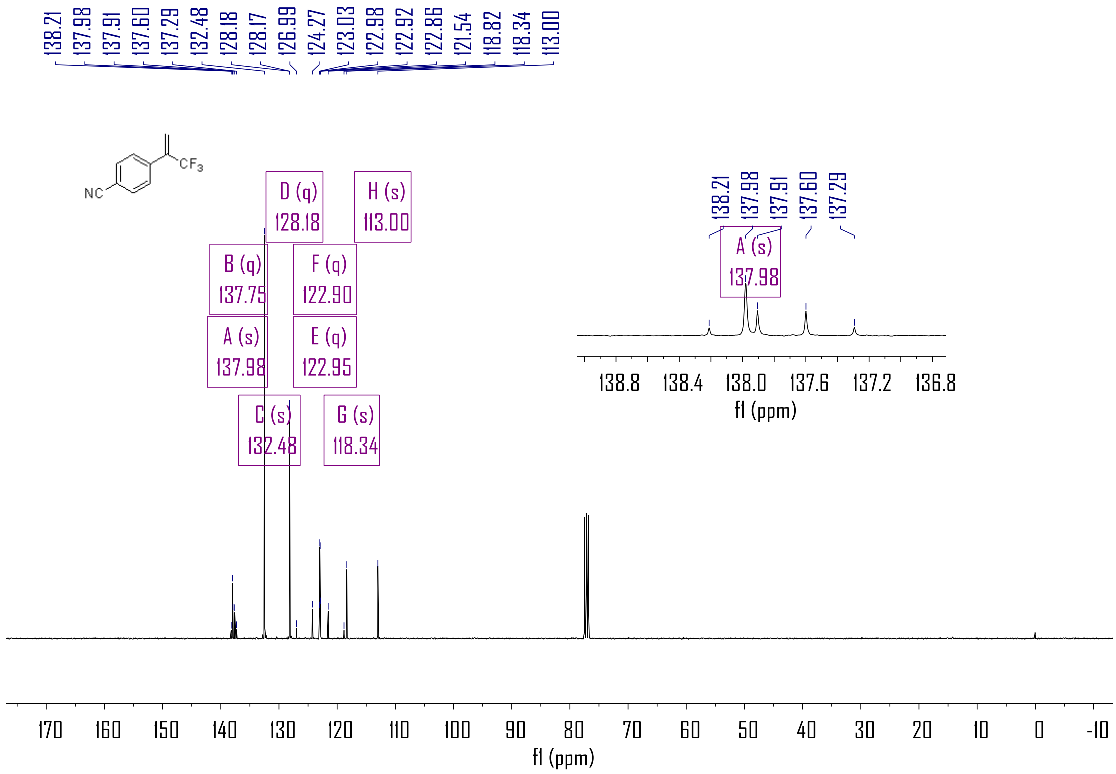

^{13}C NMR spectra for **1k**.

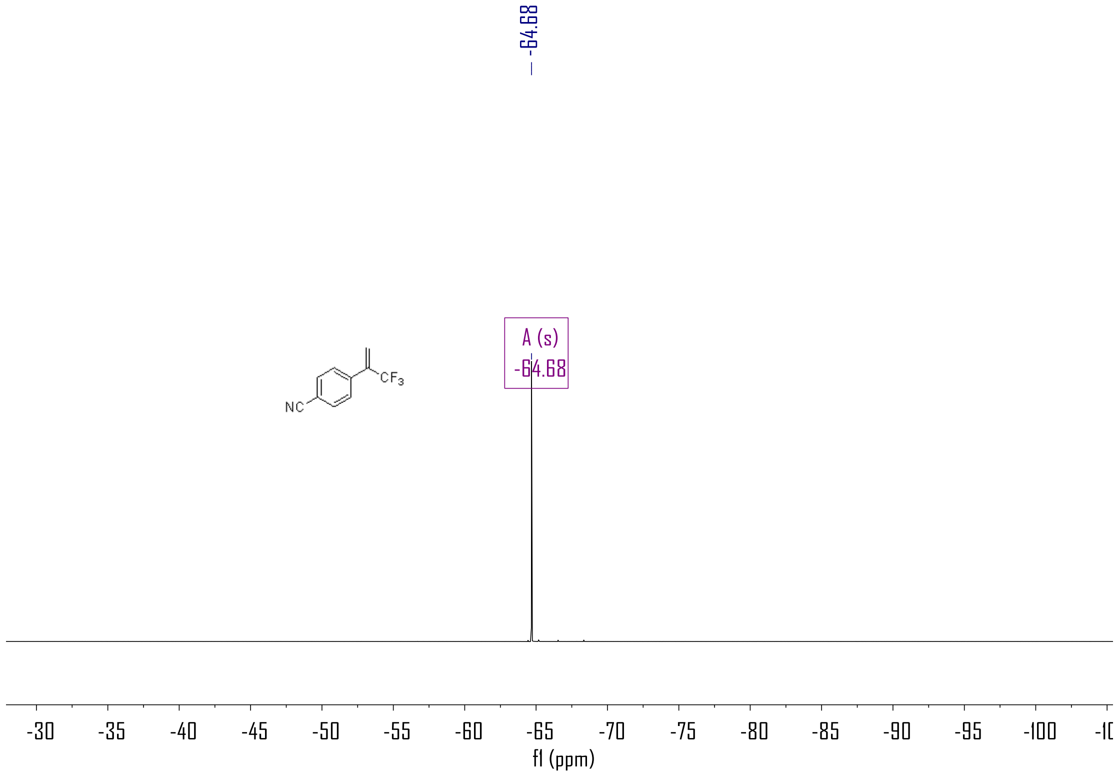

^{19}F NMR spectra for **1k**.

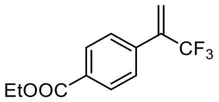

ethyl 4-(3,3,3-trifluoroprop-1-en-2-yl)benzoate (**1l**)

^{1}H NMR (400 MHz, Chloroform-*d*) δ 8.06 (d, *J* = 8.5 Hz, 2H), 7.53 (d, *J* = 8.2 Hz, 2H), 6.05 (q, *J* = 1.4 Hz, 1H), 5.86 (q, *J* = 1.7 Hz, 1H), 4.39 (q, *J* = 7.1 Hz, 2H), 1.40 (t, *J* = 7.1 Hz, 3H).

^{13}C NMR (101 MHz, Chloroform-*d*) δ 166.10, 138.45 (q, *J* = 30.4 Hz), 137.86, 131.06, 129.89, 127.43, 123.27 (q, *J* = 272.3 Hz), 121.90 (q, *J* = 5.7 Hz), 61.28, 14.40.

^{19}F NMR (376 MHz, Chloroform-*d*) δ -64.68.

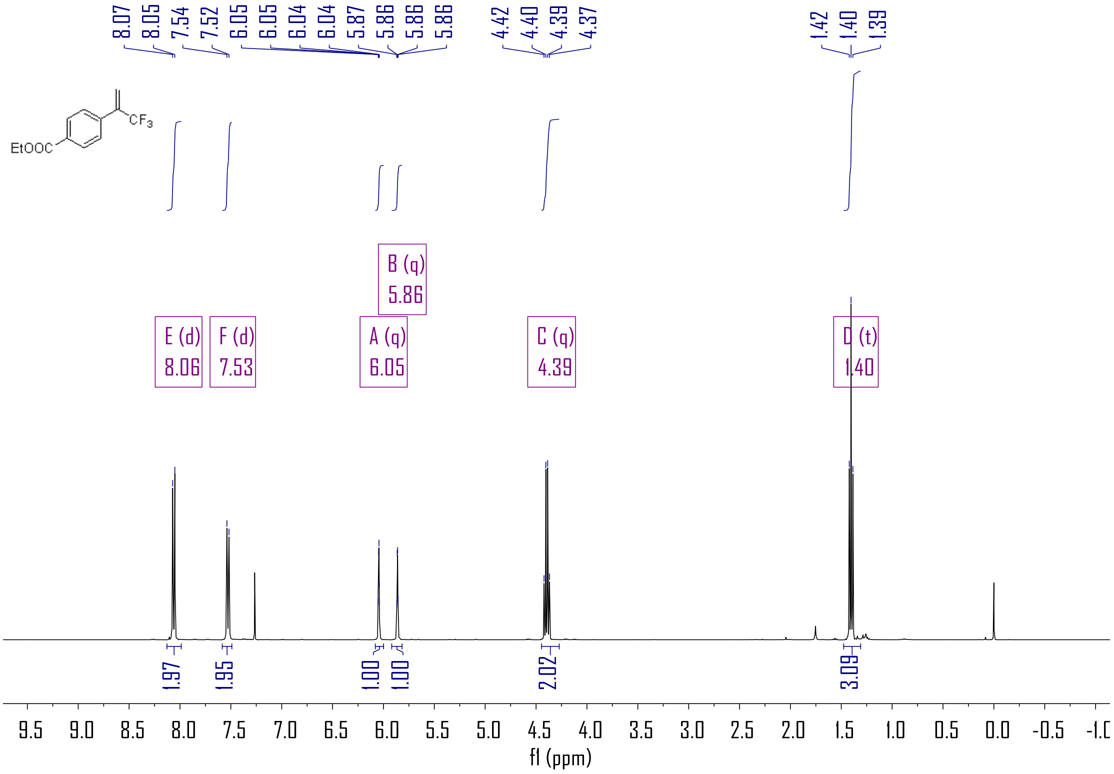

^{1}H NMR spectra for **1l**.

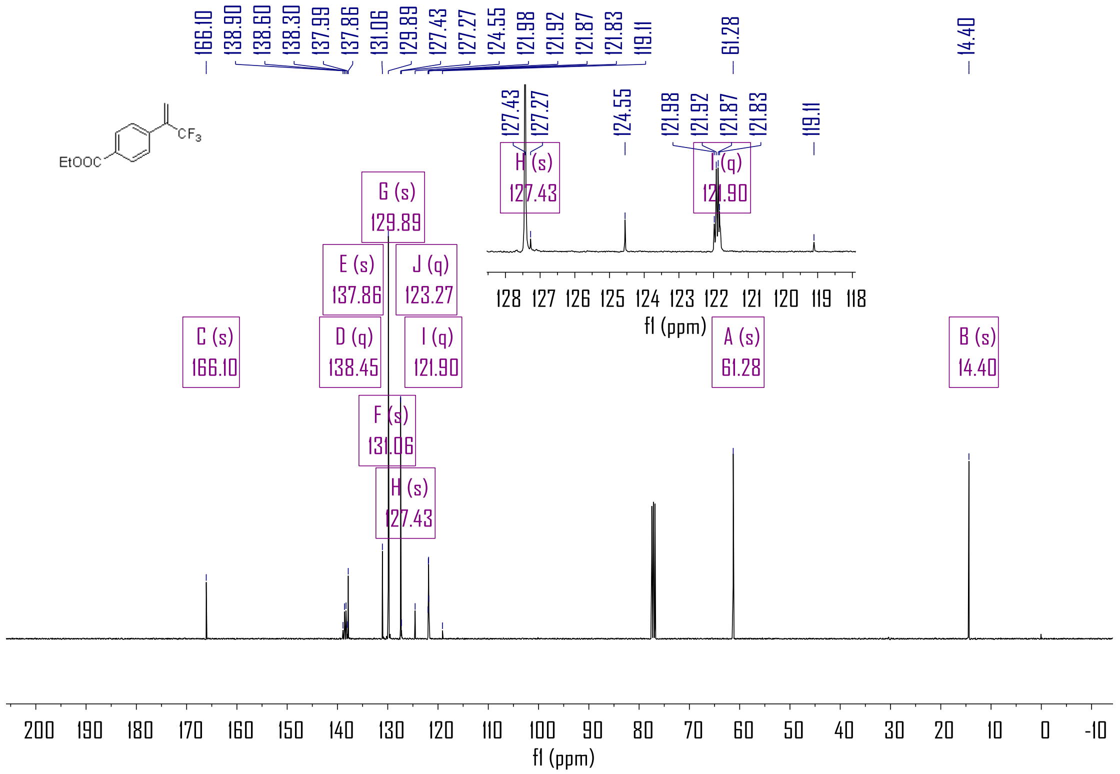

^{13}C NMR spectra for **1l**.

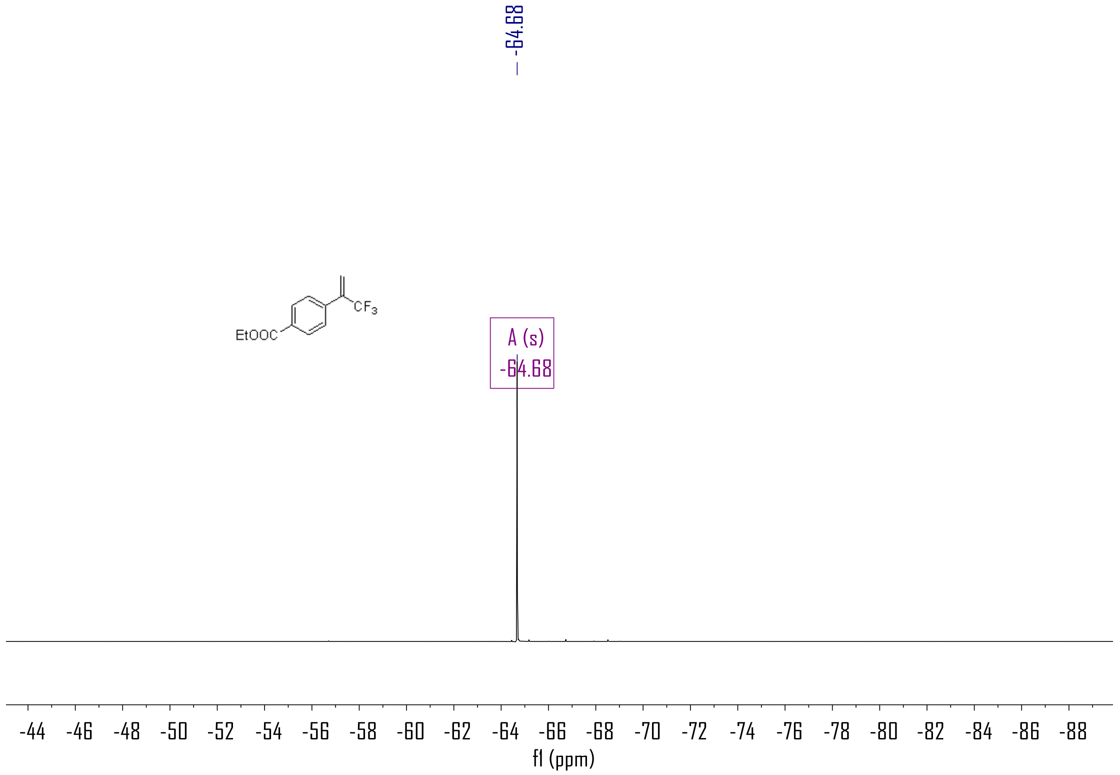

^{19}F NMR spectra for **1l**.

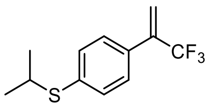

isopropyl(4-(3,3,3-trifluoroprop-1-en-2-yl)phenyl)sulfane (**1o**)

^{1}H NMR (400 MHz, Chloroform-*d*) δ 7.44 – 7.31 (m, 4H), 5.93 (q, *J* = 1.4 Hz, 1H), 5.77 (q, *J* = 1.7 Hz, 1H), 3.44 (p, *J* = 6.7 Hz, 1H), 1.32 (d, *J* = 6.7 Hz, 6H).

^{13}C NMR (101 MHz, Chloroform-*d*) δ 138.47 (q, *J* = 30.1 Hz), 137.54, 131.53, 130.89, 127.73 (q, *J* = 1.0 Hz), 123.42 (q, *J* = 274.0 Hz), 120.19 (q, *J* = 5.8 Hz), 37.84, 23.19.

^{19}F NMR (376 MHz, Chloroform-*d*) δ -64.67.

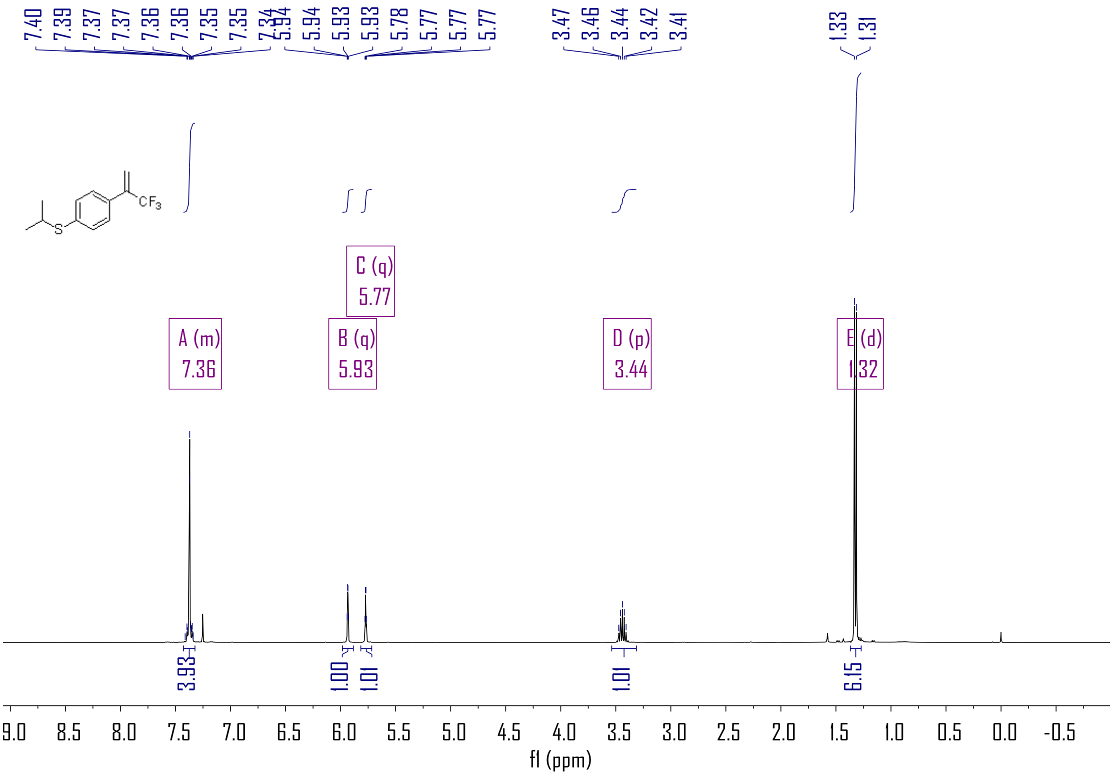

^{1}H NMR spectra for **1o**.

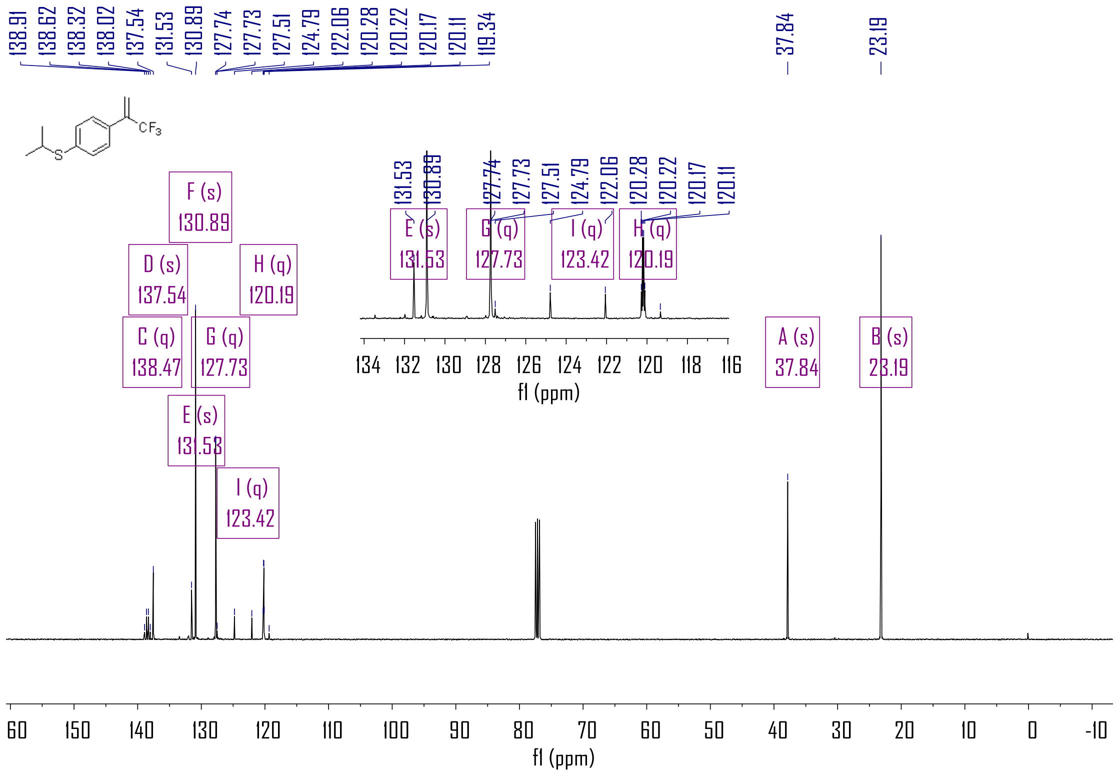

^{13}C NMR spectra for **1o**.

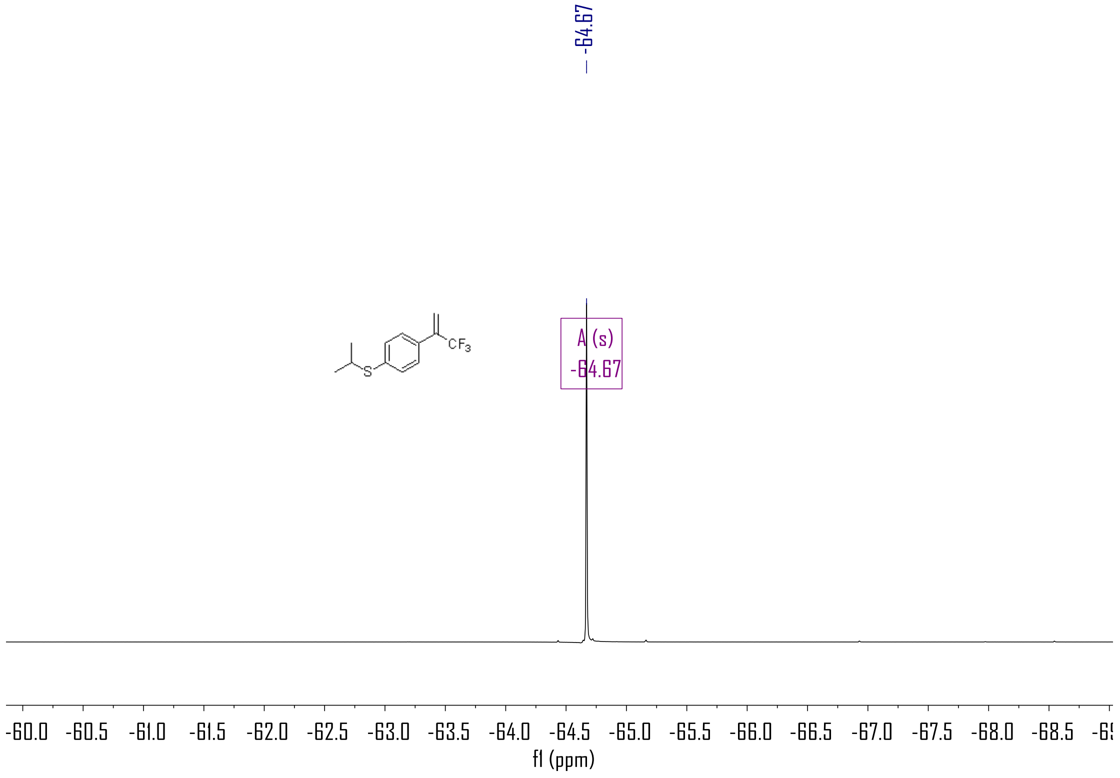

^{19}F NMR spectra for **1o**.

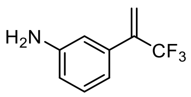

3-(3,3,3-trifluoroprop-1-en-2-yl)aniline (**1q**)

^{1}H NMR (400 MHz, Chloroform-*d*) δ 7.16 (t, *J* = 7.9 Hz, 1H), 6.84 (d, *J* = 7.7 Hz, 1H), 6.76 (s, 1H), 6.69 (dd, *J* = 8.0, 1.7 Hz, 1H), 5.90 (q, *J* = 1.4 Hz, 1H), 5.73 (q, *J* = 1.7 Hz, 1H), 3.53 (s, 2H).

^{13}C NMR (101 MHz, Chloroform-*d*) δ 146.53, 139.12 (q, *J* = 29.8 Hz), 134.82, 129.61, 123.48 (q, *J* = 274.0 Hz), 120.32 (q, *J* = 5.8 Hz), 117.89, 115.79, 114.05.

^{19}F NMR (376 MHz, Chloroform-*d*) δ -64.64.

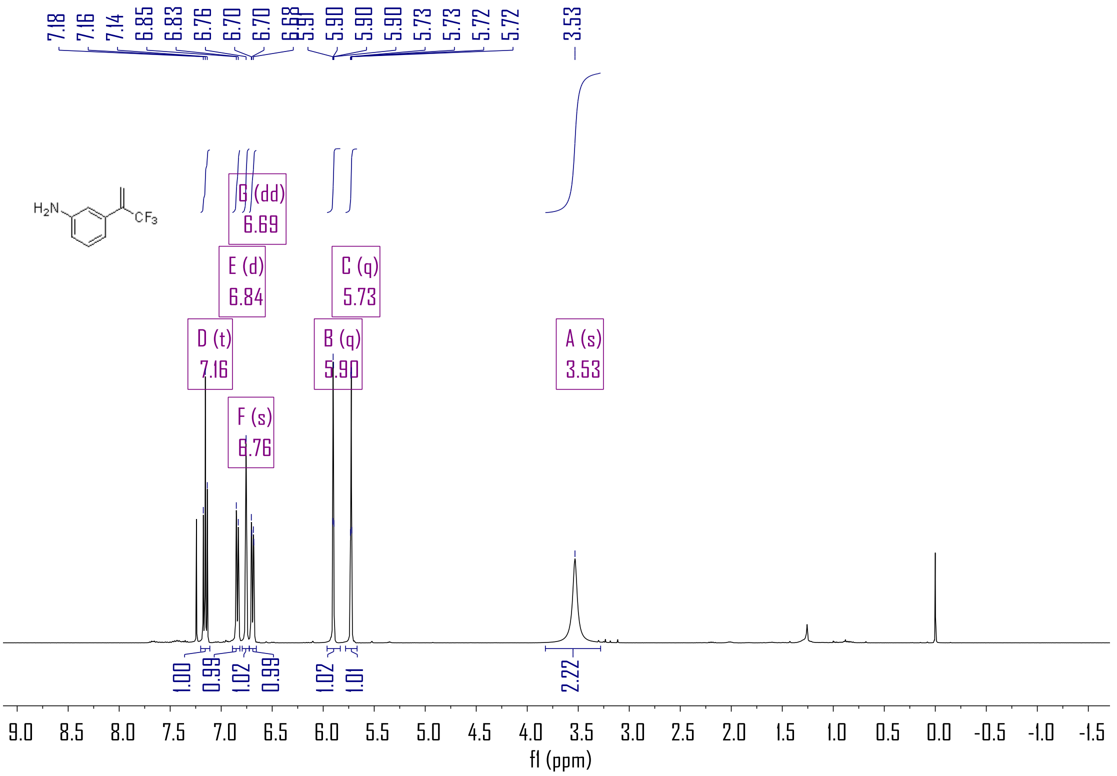

^{1}H NMR spectra for **1q**.

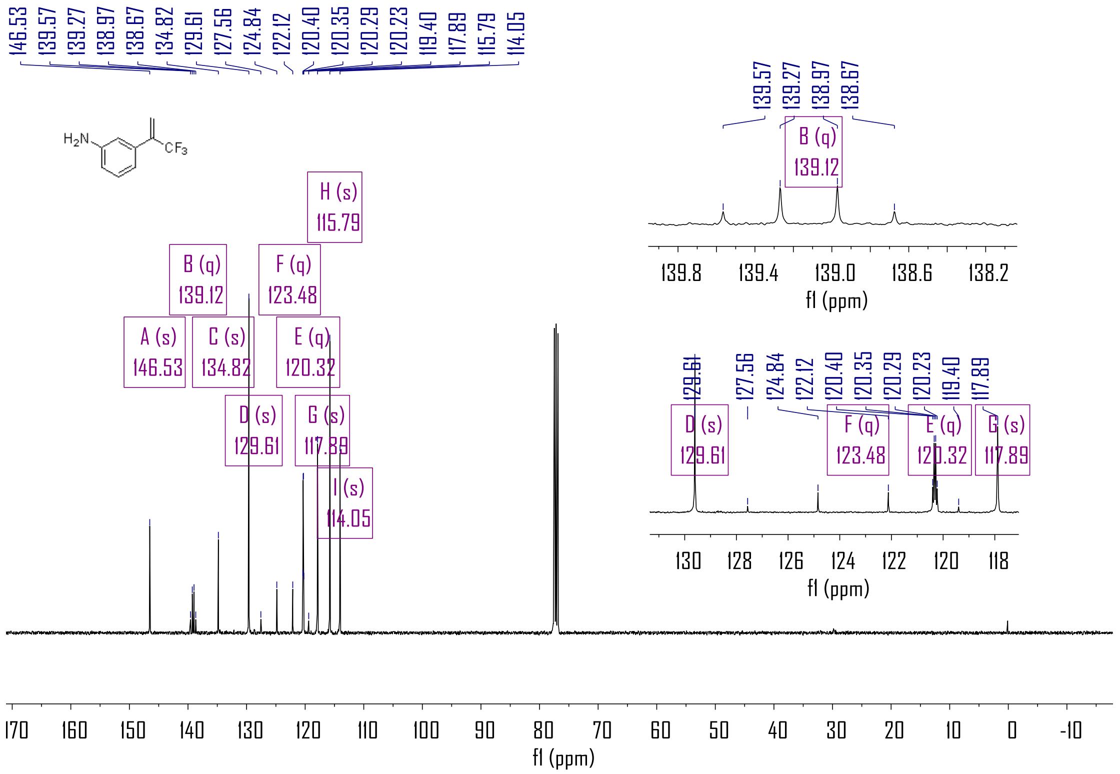

^{13}C NMR spectra for **1q**.

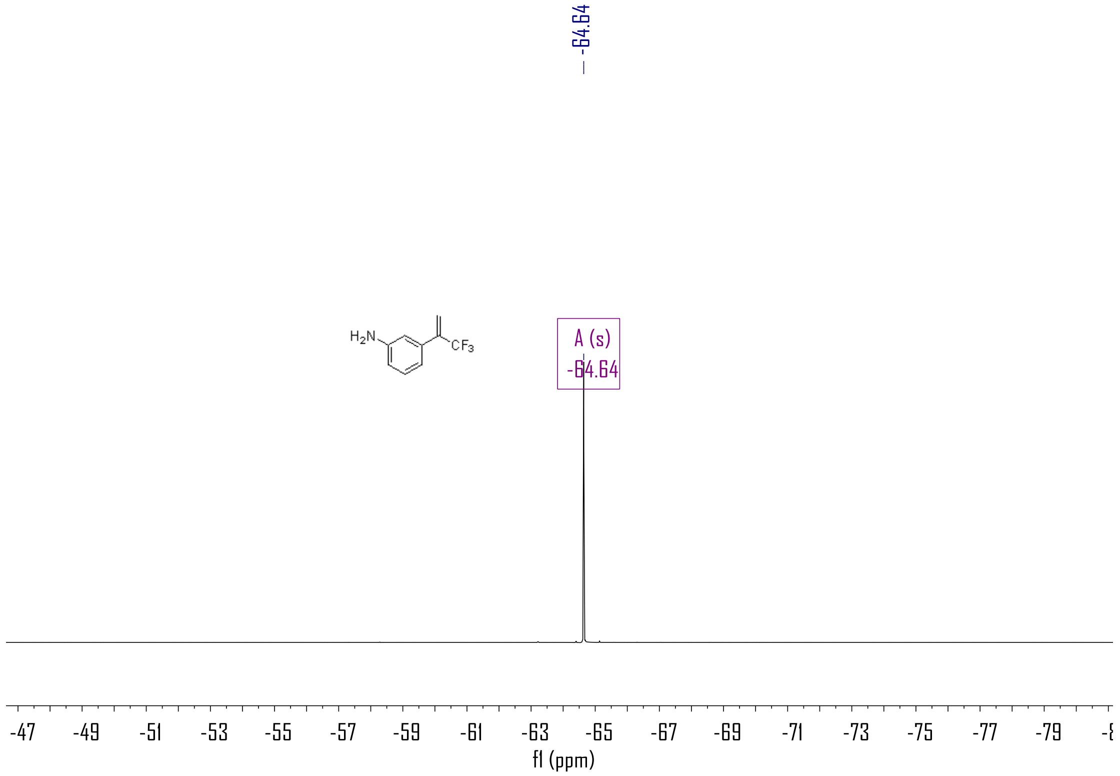

^{19}F NMR spectra for **1q**.

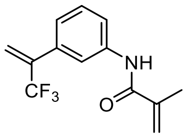

N-(3-(3,3,3-trifluoroprop-1-en-2-yl)phenyl)methacrylamide (**1u**)

^{1}H NMR (400 MHz, Chloroform-*d*) δ 7.67 (s, 1H), 7.62 (d, J = 8.0 Hz, 2H), 7.35 (t, J = 7.9 Hz, 1H), 7.21 (d, J = 7.8 Hz, 1H), 5.97 (s, 1H), 5.83 – 5.78 (m, 2H), 5.48 (s, 1H), 2.06 (s, 3H).

^{13}C NMR (101 MHz, Chloroform-*d*) δ 166.85, 140.87, 138.59 (q, J = 30.2 Hz), 138.16, 134.56, 129.38, 123.52, 123.35 (q, J = 274.0 Hz), 121.13 (q, J = 5.8 Hz), 120.73, 120.25, 119.15, 18.86.

^{19}F NMR (376 MHz, Chloroform-*d*) δ -64.71.

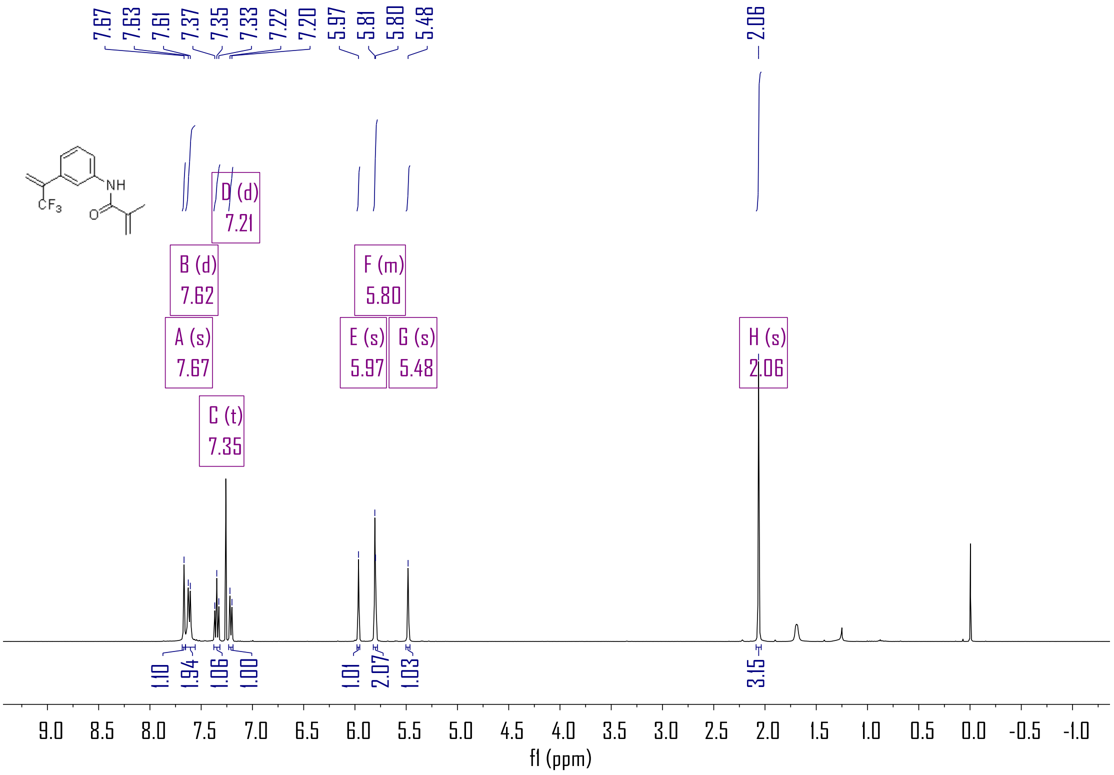

^{1}H NMR spectra for **1u**.

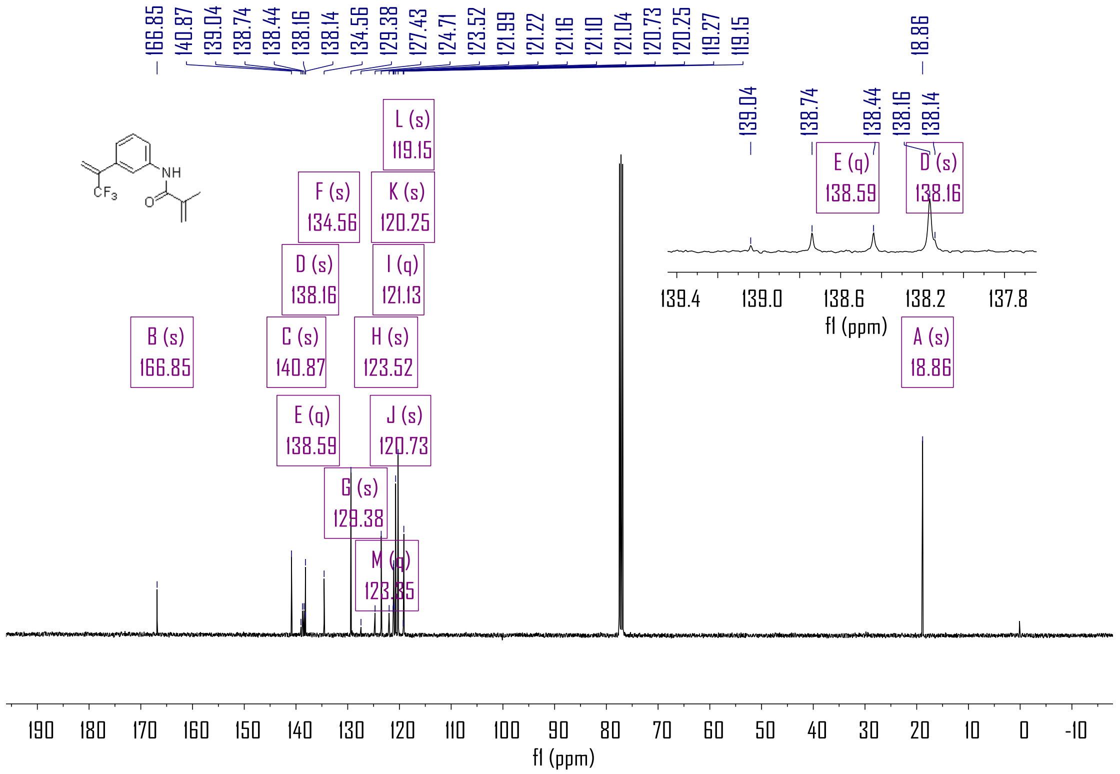

^{13}C NMR spectra for **1u**.

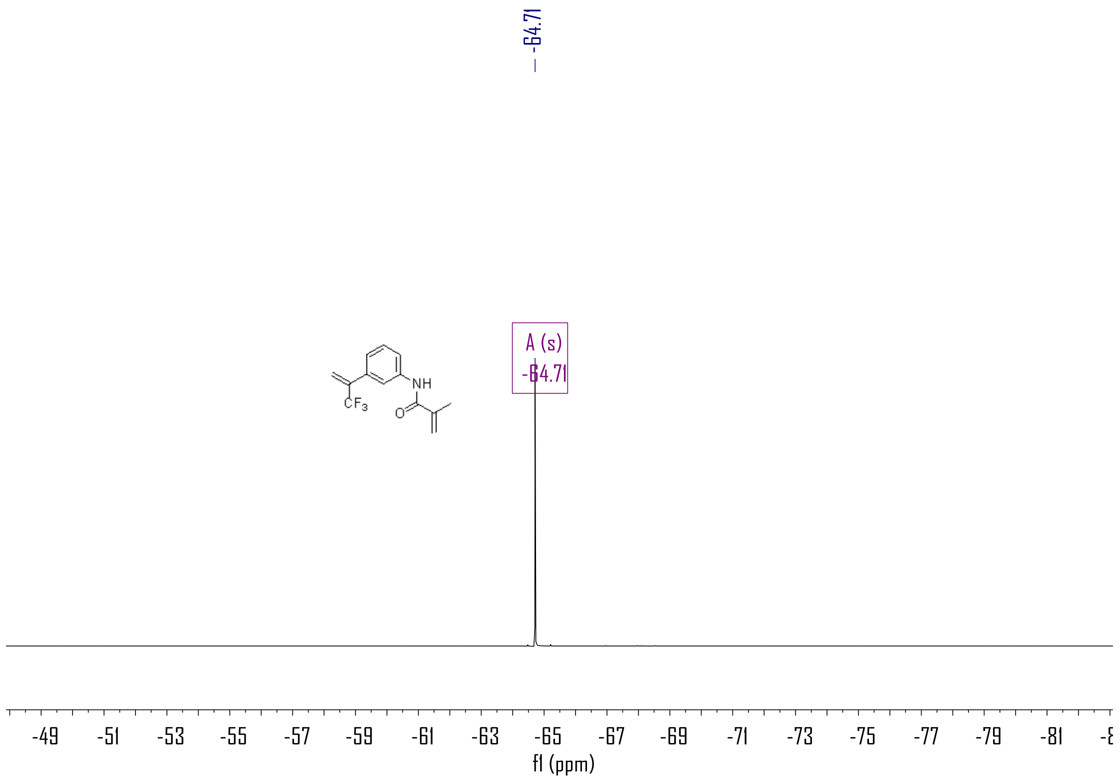

^{19}F NMR spectra for **1u**.

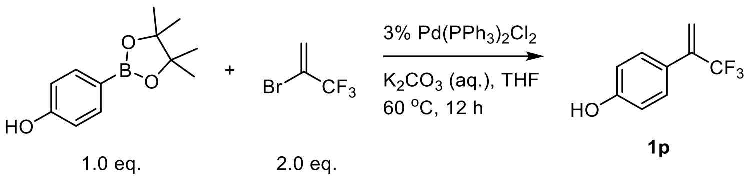

Preparation of trifluoromethyl alkenes **1p**.

According to the reported literature, trifluoromethyl alkenes **1p** was conveniently synthesized in 50 mmol scale under slightly modified reaction conditions.[2] In a Schlenk tube equipped with stir bar, 4-(4,4,5,5-tetramethyl-1,3,2-dioxaborolan-2-yl)phenol (1.0 equiv., 50 mmol, 11.0 g) and $Pd(PPh_3)_2Cl_2$ (3 mol%, 1.5 mmol, 1.05 g) were added. The vessel was evacuated and filled with argon (three cycles), then aqueous K_2CO_3 (2.0 M, 100 mL) and THF (150 mL) were added. After the addition of 2-bromo-3,3,3-trifluoropropene (2.0 equiv., 100 mmol, 10.5 mL), the solution was stirred at 60 °C for 12 hours (TLC tracking detection). The mixture was purified by column chromatography to afford the corresponding trifluoromethyl alkenes.

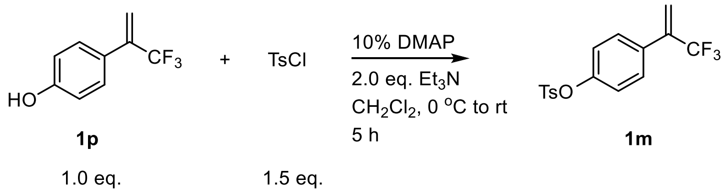

Preparation of trifluoromethyl alkenes **1m**.

According to the reported literature, trifluoromethyl alkenes **1m** was conveniently synthesized.[5] To a solution of **1p** (1.0 equiv., 10 mmol, 1.88 g), 4-dimethylaminopyridine (DMAP) (10 mol%, 1 mmol, 122 mg) and Et_3N (2.0 equiv., 20 mmol, 2.8 mL) in CH_2Cl_2 (50 mL), TsCl (1.5 equiv., 15 mmol, 2.86 g) dissolved in CH_2Cl_2 (20 mL) was added at 0 °C over 10 minutes. Then solution was stirred at room temperature for 5 hours (TLC tracking detection). The mixture was purified by column chromatography to afford trifluoromethyl alkenes **1m**.

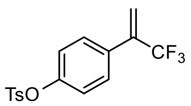

4-(3,3,3-trifluoroprop-1-en-2-yl)phenyl 4-methylbenzenesulfonate (**1m**)

^{1}H NMR (400 MHz, Chloroform-*d*) δ 7.73 (d, *J* = 8.4 Hz, 2H), 7.38 (d, *J* = 8.6 Hz, 2H), 7.33 (d, *J* = 8.2 Hz, 2H), 7.01 (d, *J* = 8.8 Hz, 2H), 5.97 (q, *J* = 1.4 Hz, 1H), 5.75 (q, *J* = 1.7 Hz, 1H), 2.46 (s, 3H).

^{13}C NMR (101 MHz, Chloroform-*d*) δ 150.12, 145.72, 137.87 (q, *J* = 30.4 Hz), 132.57, 132.40, 129.98, 128.88 (q, *J* = 1.1 Hz), 128.60, 123.17 (q, *J* = 273.9 Hz), 122.67, 121.36 (q, *J* = 5.7 Hz), 21.84.

^{19}F NMR (376 MHz, Chloroform-*d*) δ -64.88.

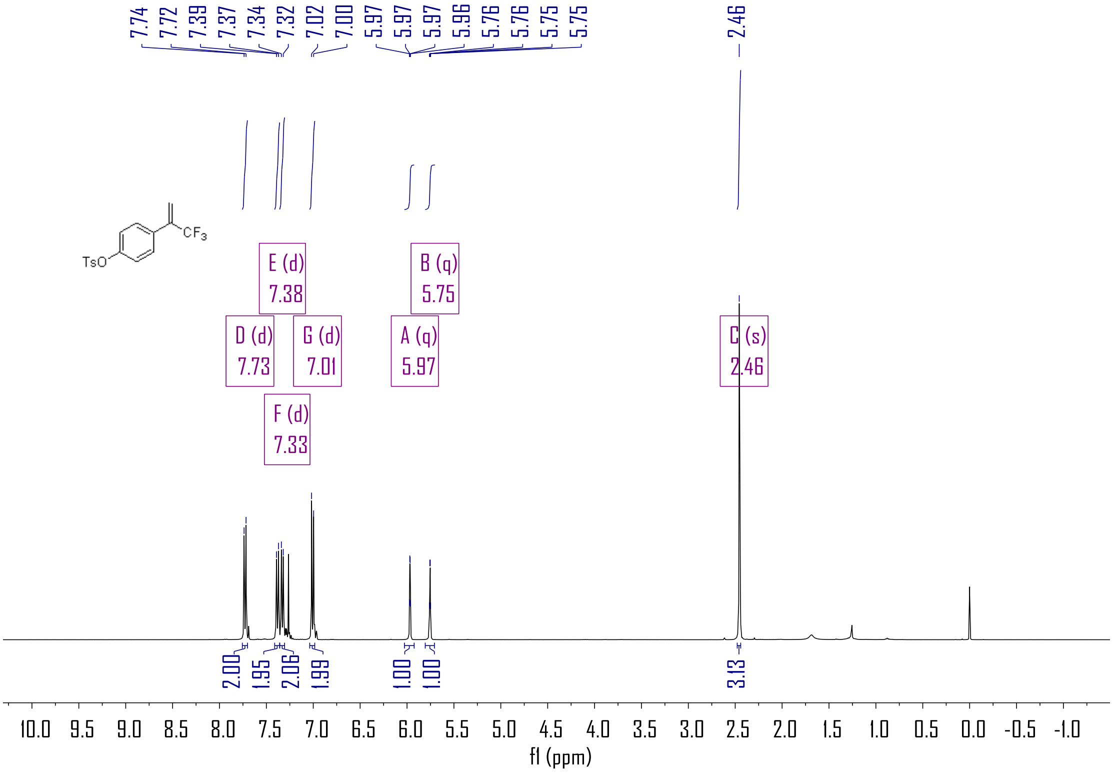

^{1}H NMR spectra for **1m**.

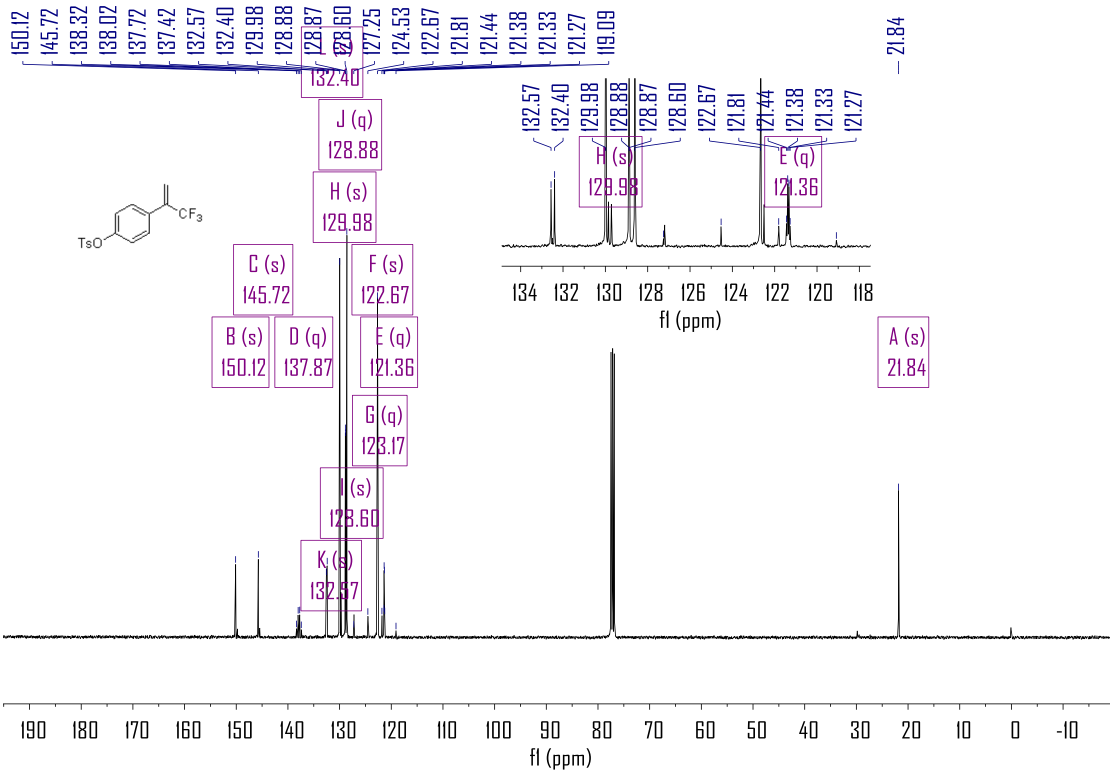

^{13}C NMR spectra for **1m**.

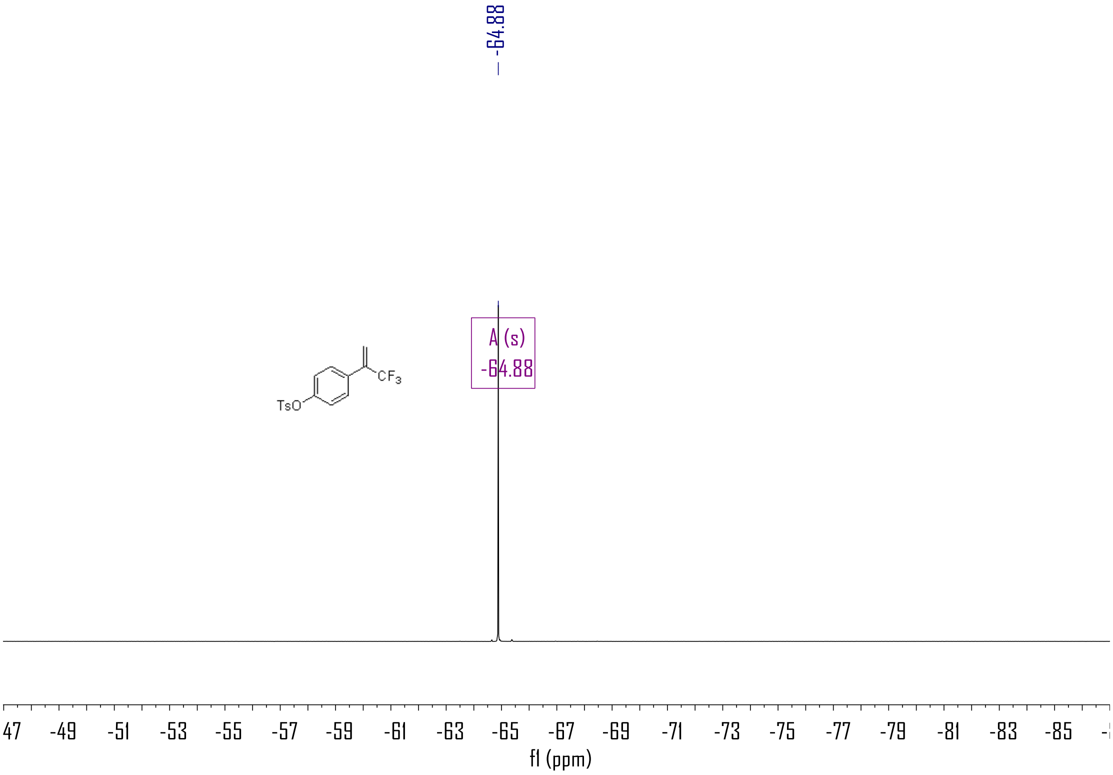

^{19}F NMR spectra for **1m**.

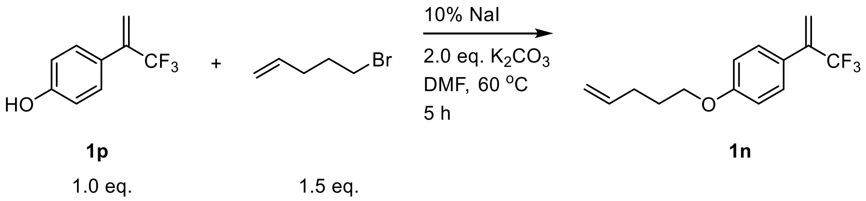

Preparation of trifluoromethyl alkenes **1n**.

According to the reported literature, trifluoromethyl alkenes **1n** was conveniently synthesized.[6] **1p** (1.0 equiv., 10 mmol, 1.88 g), K_2CO_3 (2.0 equiv., 20 mmol, 2.76 g) and NaI (10 mol%, 1 mmol, 150 mg) in DMF (50 mL) was stirred at 60 °C for 30 minutes. Then 5-bromopent-1-ene (1.5 equiv., 15 mmol, 1.8 mL) was added and stirred at 60 °C for 4.5 hours (TLC tracking detection). The mixture was purified by column chromatography to afford trifluoromethyl alkenes **1n**.

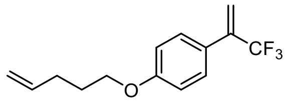

1-(pent-4-en-1-yloxy)-4-(3,3,3-trifluoroprop-1-en-2-yl)benzene (**1n**)

^{1}H NMR (400 MHz, Chloroform-*d*) δ 7.38 (d, *J* = 8.5 Hz, 2H), 6.89 (d, *J* = 8.8 Hz, 2H), 5.94 – 5.77 (m, 2H), 5.69 (q, *J* = 1.8 Hz, 1H), 5.07 (dq, *J* = 17.1, 1.7 Hz, 1H), 5.01 (dq, *J* = 10.1, 1.4 Hz, 1H), 3.98 (t, *J* = 6.4 Hz, 2H), 2.24 (tdd, *J* = 7.9, 6.1, 1.5 Hz, 2H), 1.97 – 1.80 (m, 2H).

^{13}C NMR (101 MHz, Chloroform-*d*) δ 159.77, 138.49 (q, *J* = 29.8 Hz), 137.83, 128.71, 125.98, 123.62 (q, *J* = 274.1 Hz), 118.84 (q, *J* = 5.8 Hz), 115.42, 114.62, 67.34, 30.22, 28.49.

^{19}F NMR (376 MHz, Chloroform-*d*) δ -64.80.

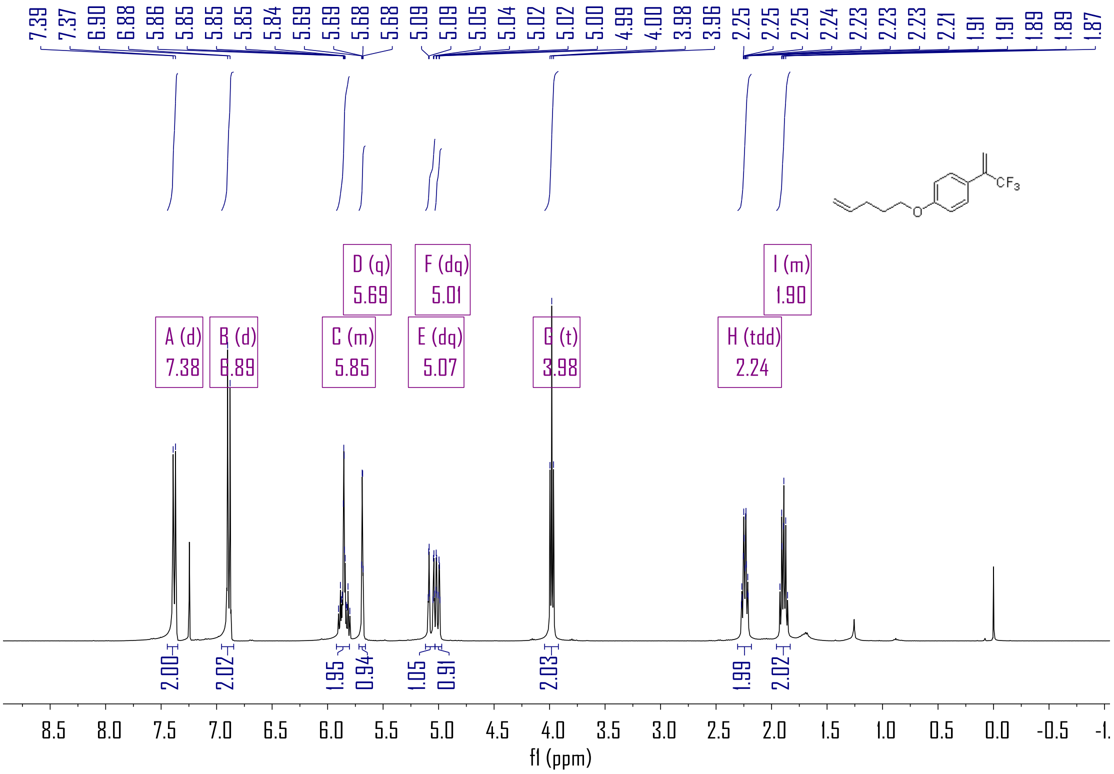

^{1}H NMR spectra for **1n**.

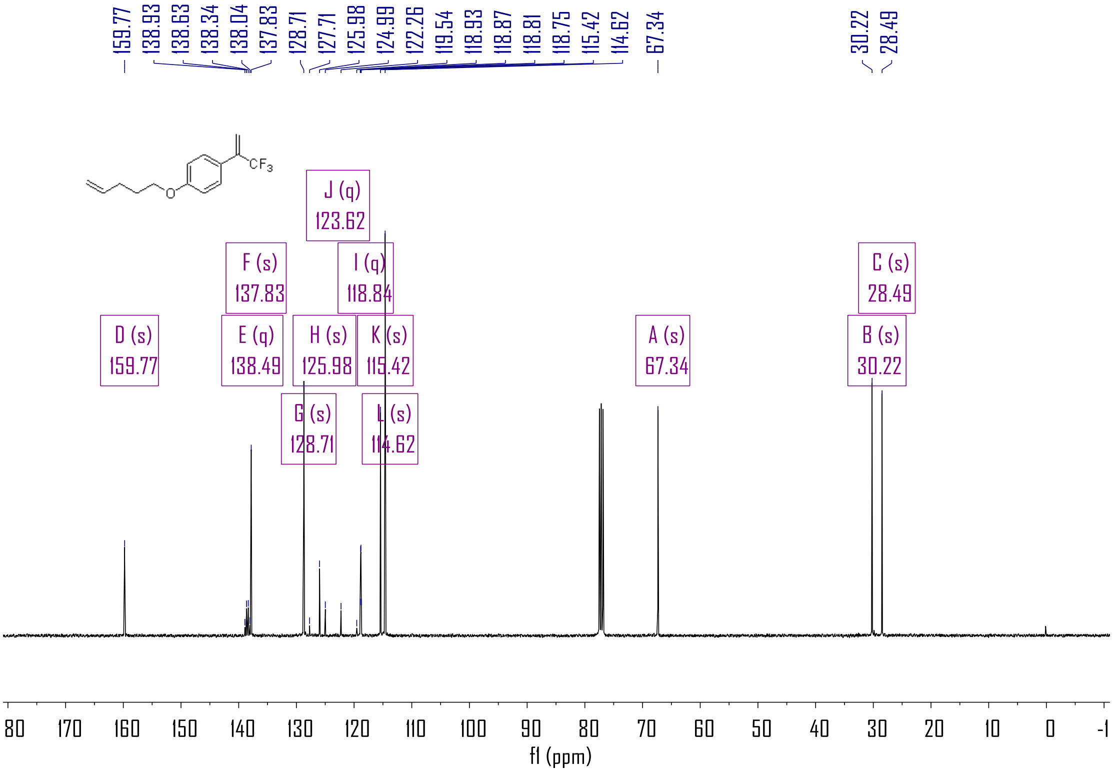

^{13}C NMR spectra for **1n**.

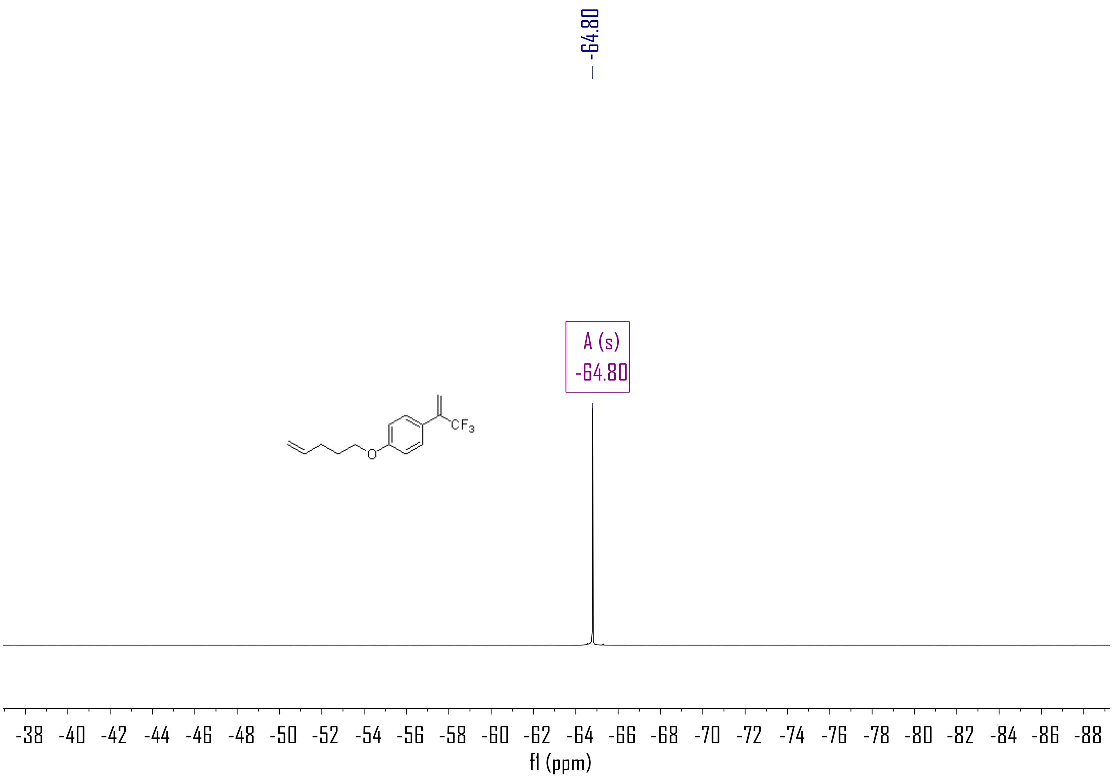

^{19}F NMR spectra for **1n**.

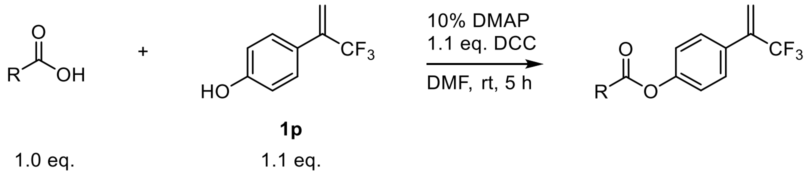

Preparation of trifluoromethyl alkenes **1r** & **1s**.

According to the reported literature, **1r** & **1s** were conveniently synthesized in gram scale.[7] To a solution of carboxylic acid (1.0 equiv., 10 mmol), 4-dimethylaminopyridine (DMAP) (10 mol%, 1 mmol, 122 mg) and **1p** (1.1 equiv., 11 mmol, 2.07 g) in DMF (25 mL), *N*,*N'*-dicyclohexylcarbodiimide (DCC) (1.1 equiv., 11 mmol, 2.27 g) was added. The reaction mixture was stirred at room temperature for 5 hours (TLC tracking detection). The mixture was purified by column chromatography to afford the corresponding trifluoromethyl alkenes **1r** & **1s**.

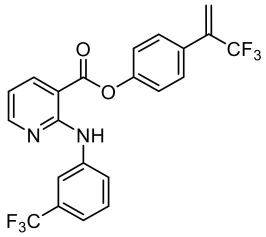

4-(3,3,3-trifluoroprop-1-en-2-yl)phenyl 2-((3-(trifluoromethyl)phenyl)amino)nicotinate (**1r**)

^{1}H NMR (400 MHz, Chloroform-*d*) δ 10.23 (s, 1H), 8.49 (d, *J* = 6.3 Hz, 2H), 8.15 – 8.03 (m, 1H), 7.92 – 7.79 (m, 1H), 7.56 (d, *J* = 8.4 Hz, 2H), 7.41 (t, *J* = 8.0 Hz, 1H), 7.31 – 7.26 (m, 1H), 7.26 – 7.20 (m, 2H), 6.96 – 6.83 (m, 1H), 6.00 (q, *J* = 1.4 Hz, 1H), 5.80 (q, *J* = 1.7 Hz, 1H).

^{13}C NMR (101 MHz, Chloroform-*d*) δ 166.37, 156.28, 154.23, 150.95, 140.94, 140.14, 138.14 (q, *J* = 30.4 Hz), 131.97, 131.29 (q, *J* = 32.1 Hz), 129.39, 129.00, 124.25 (q, *J* = 272.4 Hz), 123.67, 123.32 (q, *J* = 273.9 Hz), 122.07, 121.16 (q, *J* = 5.7 Hz), 119.46 (q, *J* = 3.8 Hz), 117.37 (q, *J* = 4.0 Hz), 114.40, 106.37.

^{19}F NMR (376 MHz, Chloroform-*d*) δ -62.61, -64.85.

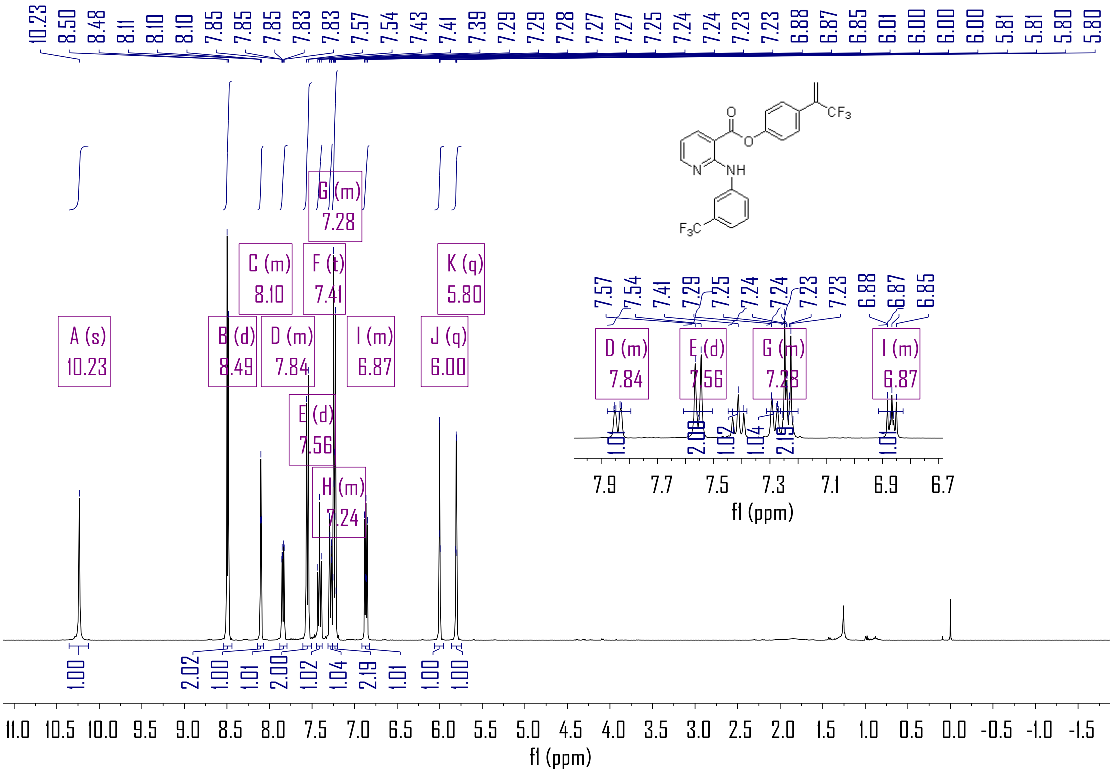

^{1}H NMR spectra for **1r**.

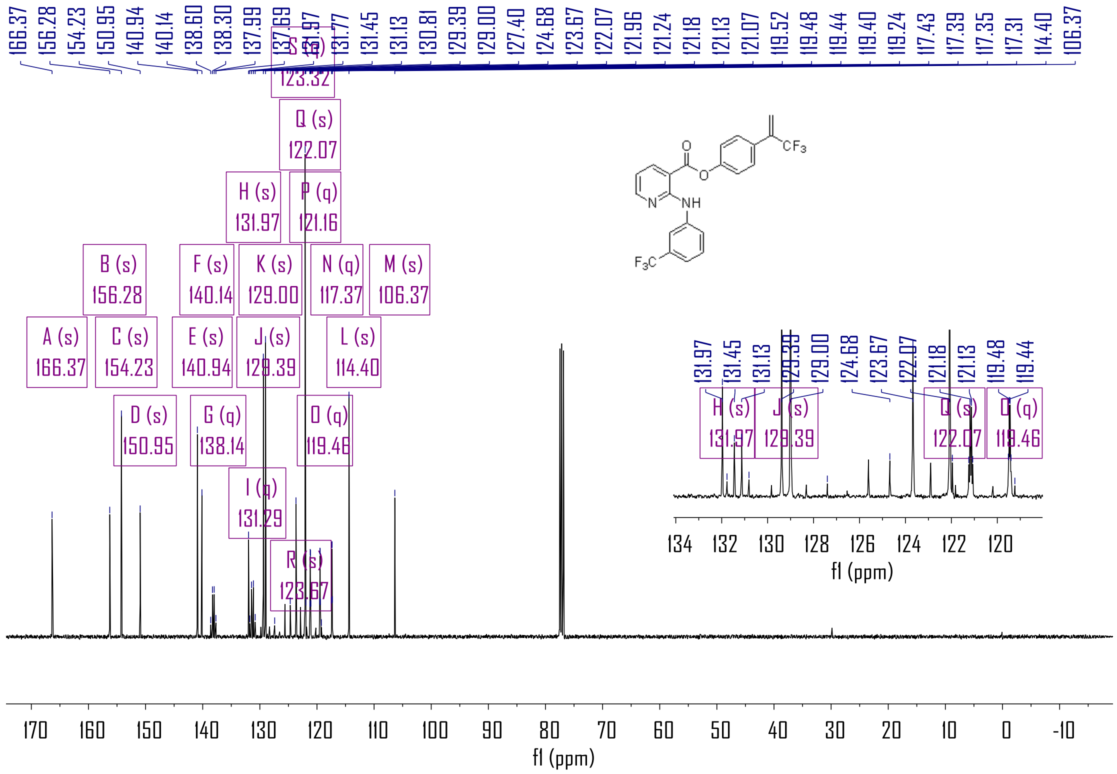

^{13}C NMR spectra for **1r**.

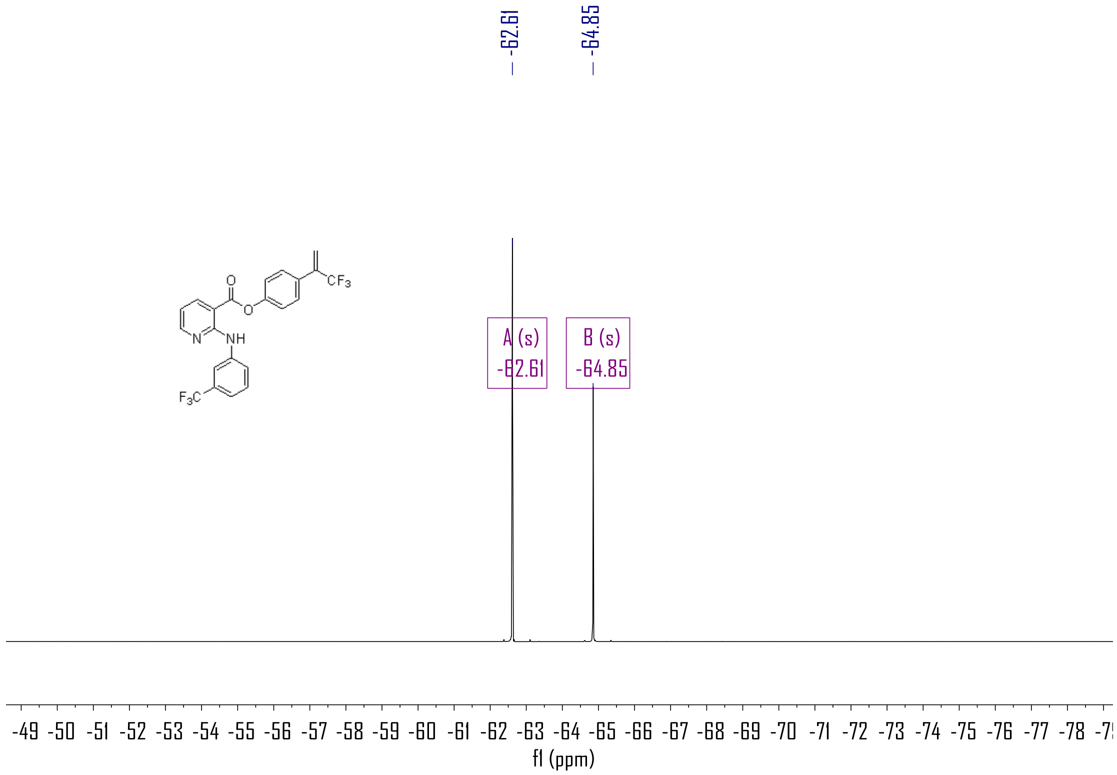

^{19}F NMR spectra for **1r**.

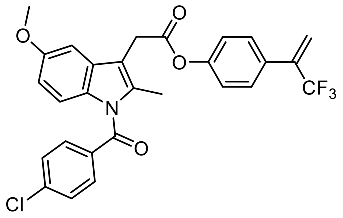

4-(3,3,3-trifluoroprop-1-en-2-yl)phenyl 2-(1-(4-chlorobenzoyl)-5-methoxy-2-methyl-1*H*-indol-3-yl)acetate (**1s**)

^{1}H NMR (400 MHz, Chloroform-*d*) δ 7.69 (d, *J* = 8.5 Hz, 2H), 7.55 – 7.42 (m, 4H), 7.16 – 7.05 (m, 3H), 6.92 (d, *J* = 9.0 Hz, 1H), 6.72 (dd, *J* = 9.0, 2.5 Hz, 1H), 5.96 (q, *J* = 1.4 Hz, 1H), 5.75 (q, *J* = 1.7 Hz, 1H), 3.93 (s, 2H), 3.85 (s, 3H), 2.47 (s, 3H).

^{13}C NMR (101 MHz, Chloroform-*d*) δ 169.18, 168.37, 156.23, 151.27, 139.44, 138.08 (q, *J* = 30.3 Hz), 136.36, 133.87, 131.41, 131.28, 130.93, 130.53, 129.23, 128.68, 123.27 (q, *J* = 274.0 Hz), 121.69, 120.87 (q, *J* = 5.8 Hz), 115.14, 111.88, 101.29, 55.79, 30.61, 13.51.

^{19}F NMR (376 MHz, Chloroform-*d*) δ -64.84.

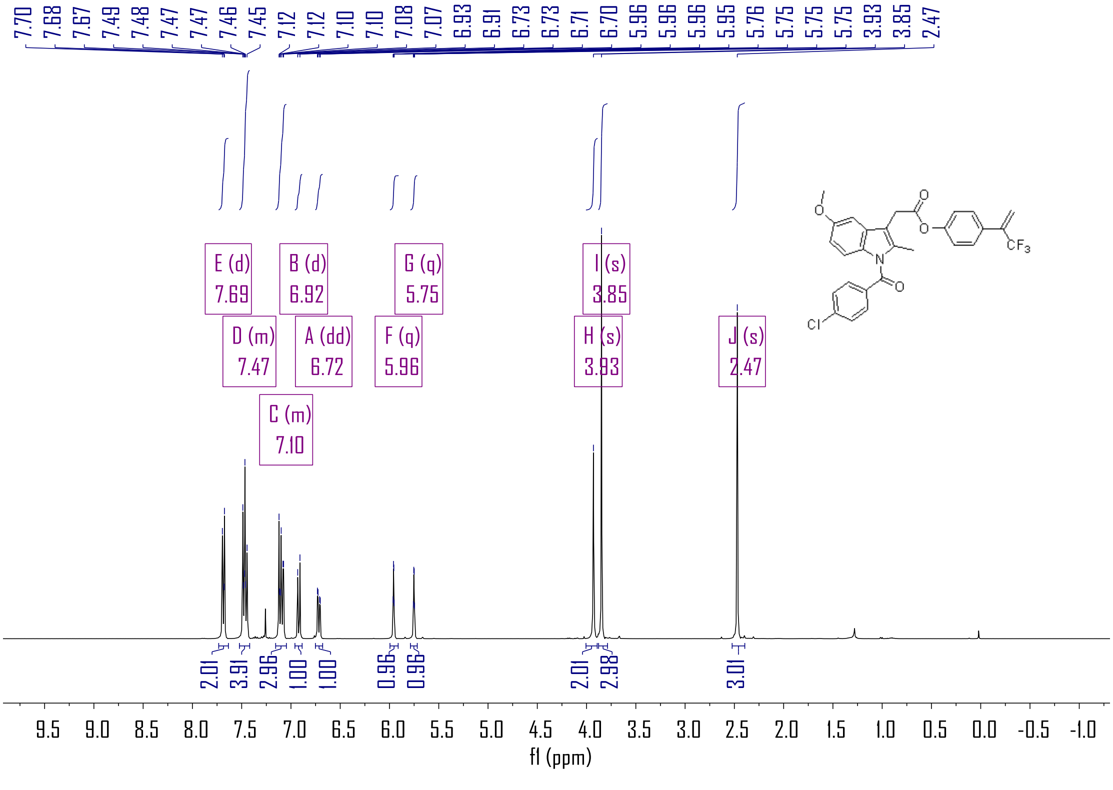

^{1}H NMR spectra for **1s**.

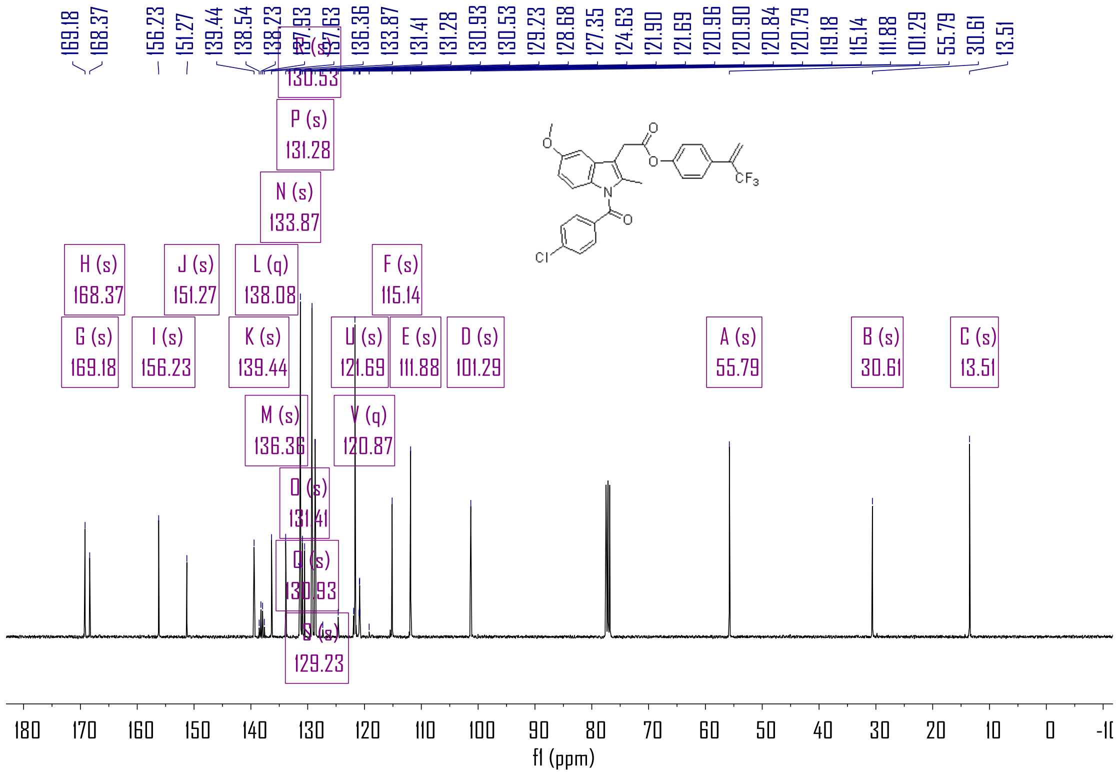

^{13}C NMR spectra for **1s**.

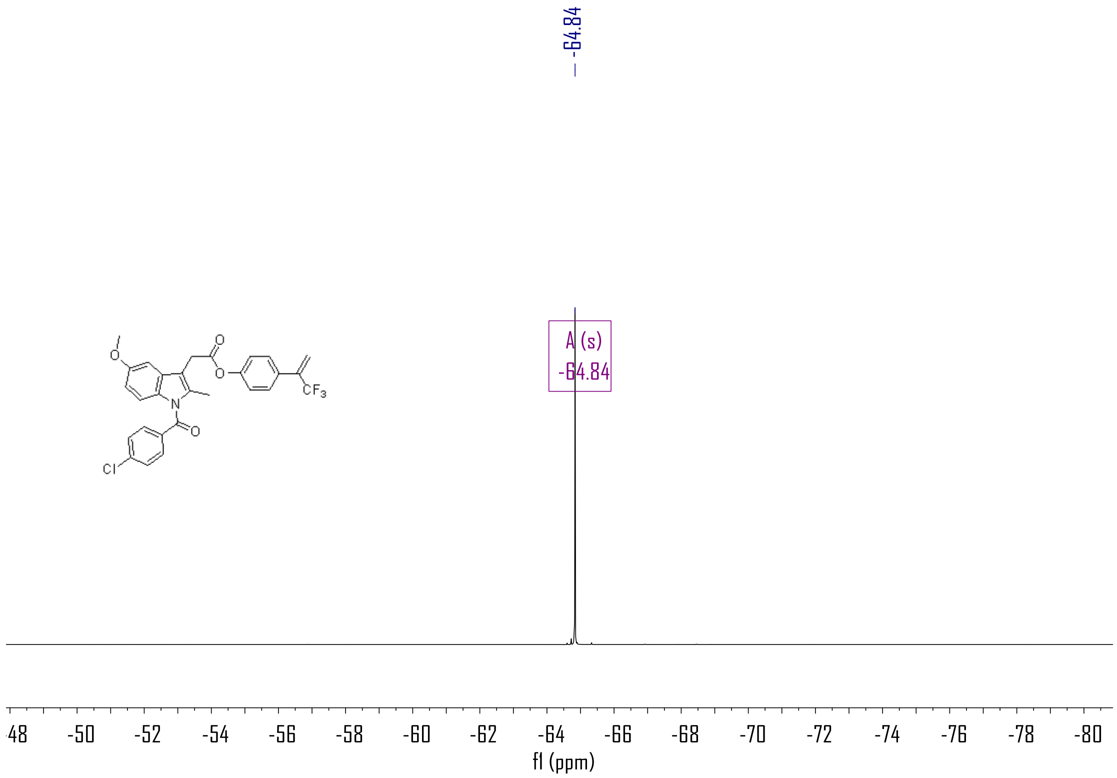

^{19}F NMR spectra for **1s**.

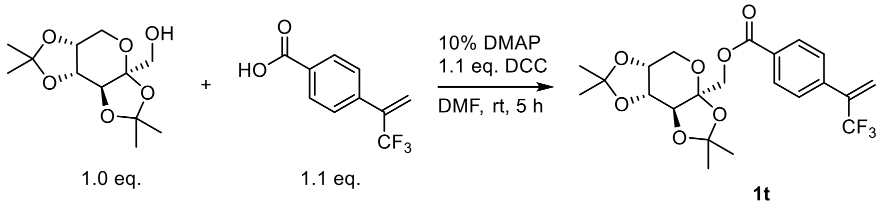

Preparation of trifluoromethyl alkenes **1t**.

According to the reported literature, **1t** was conveniently synthesized in gram scale.[7] To a solution of ((3a*S*,5a*R*,8a*R*,8b*S*)-2,2,7,7-tetramethyltetrahydro-3a*H*-bis([1,3]dioxolo)[4,5-*b*:4',5'-*d*]pyran-3a-yl)methanol (1.0 equiv., 10 mmol, 2.60 g), 4-dimethylaminopyridine (DMAP) (10 mol%, 1 mmol, 122 mg) and 4-(3,3,3-trifluoroprop-1-en-2-yl)benzoic acid (1.1 equiv., 11 mmol, 2.38 g) in DMF (25 mL), *N*,*N*'-dicyclohexylcarbodiimide (DCC) (1.1 equiv., 11 mmol, 2.27 g) was added. The reaction mixture was stirred at room temperature for 5 hours (TLC tracking detection). The mixture was purified by column chromatography to afford the corresponding trifluoromethyl alkenes **1t**.

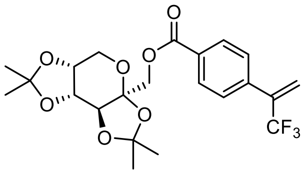

((3a*S*,5a*R*,8a*R*,8b*S*)-2,2,7,7-tetramethyltetrahydro-3a*H*-bis([1,3]dioxolo)[4,5-*b*:4',5'-*d*]pyran-3a-yl)methyl 4-(3,3,3-trifluoroprop-1-en-2-yl)benzoate (**1t**)

<u>^{1}H NMR (400 MHz, Chloroform-*d*)</u> δ 8.08 (d, *J* = 8.6 Hz, 2H), 7.53 (d, *J* = 8.2 Hz, 2H), 6.05 (q, *J* = 1.4 Hz, 1H), 5.87 (q, *J* = 1.7 Hz, 1H), 4.68 (d, *J* = 11.7 Hz, 1H), 4.64 (dd, *J* = 7.9, 2.6 Hz, 1H), 4.45 (d, *J* = 2.6 Hz, 1H), 4.34 (d, *J* = 11.8 Hz, 1H), 4.29 – 4.23 (m, 1H), 3.96 (dd, *J* = 13.0, 1.9 Hz, 1H), 3.80 (dd, *J* = 13.0, 0.8 Hz, 1H), 1.55 (s, 3H), 1.46 (s, 3H), 1.37 (s, 3H), 1.34 (s, 3H).

<u>^{13}C NMR (101 MHz, Chloroform-*d*)</u> δ 165.48, 138.34 (q, *J* = 30.5 Hz), 138.15, 130.45, 130.13, 127.51, 123.15 (q, *J* = 274.0 Hz), 122.11 (q, *J* = 5.7 Hz), 109.32, 109.00, 101.74, 70.91, 70.70, 70.22, 65.63, 61.50, 26.65, 26.00, 25.67, 24.15.

<u>^{19}F NMR (376 MHz, Chloroform-*d*)</u> δ -64.62.

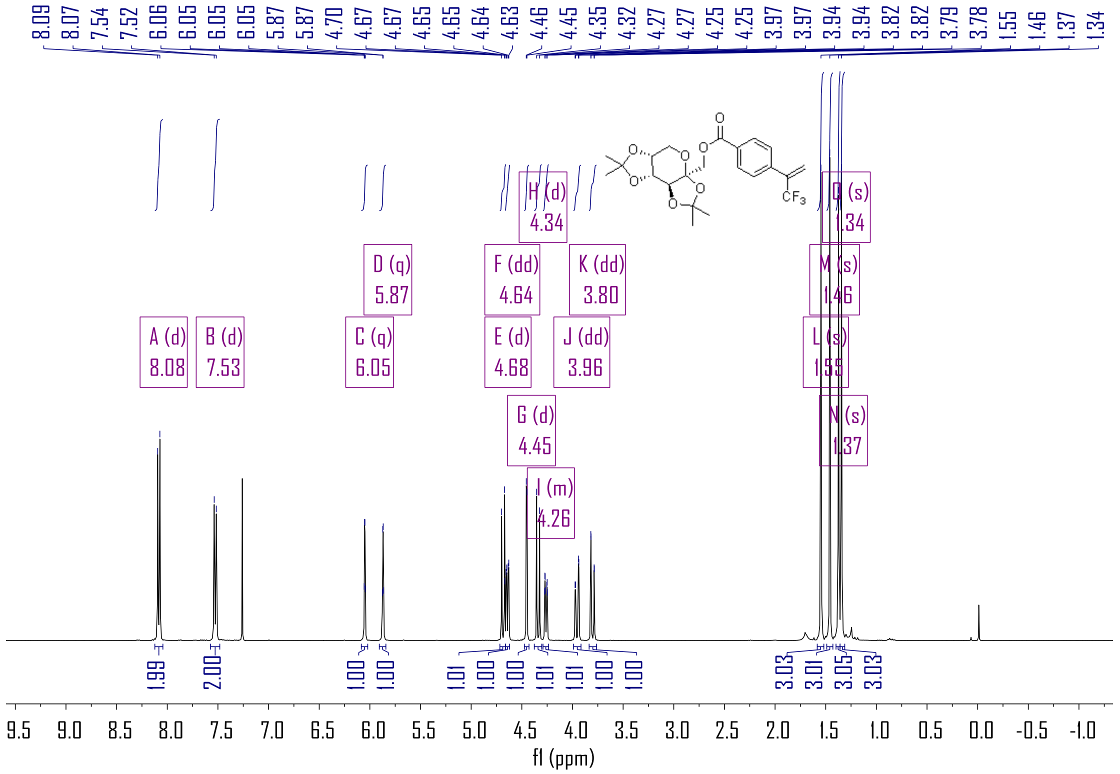

^{1}H NMR spectra for **1t**.

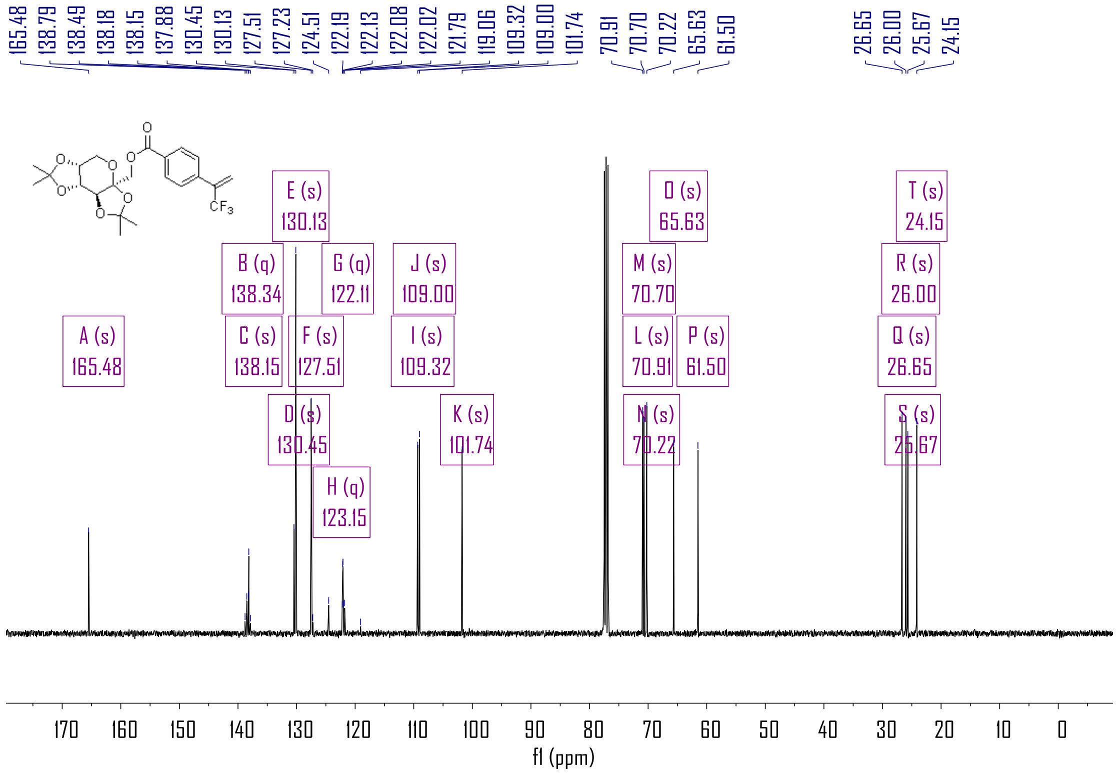

^{13}C NMR spectra for **1t**.

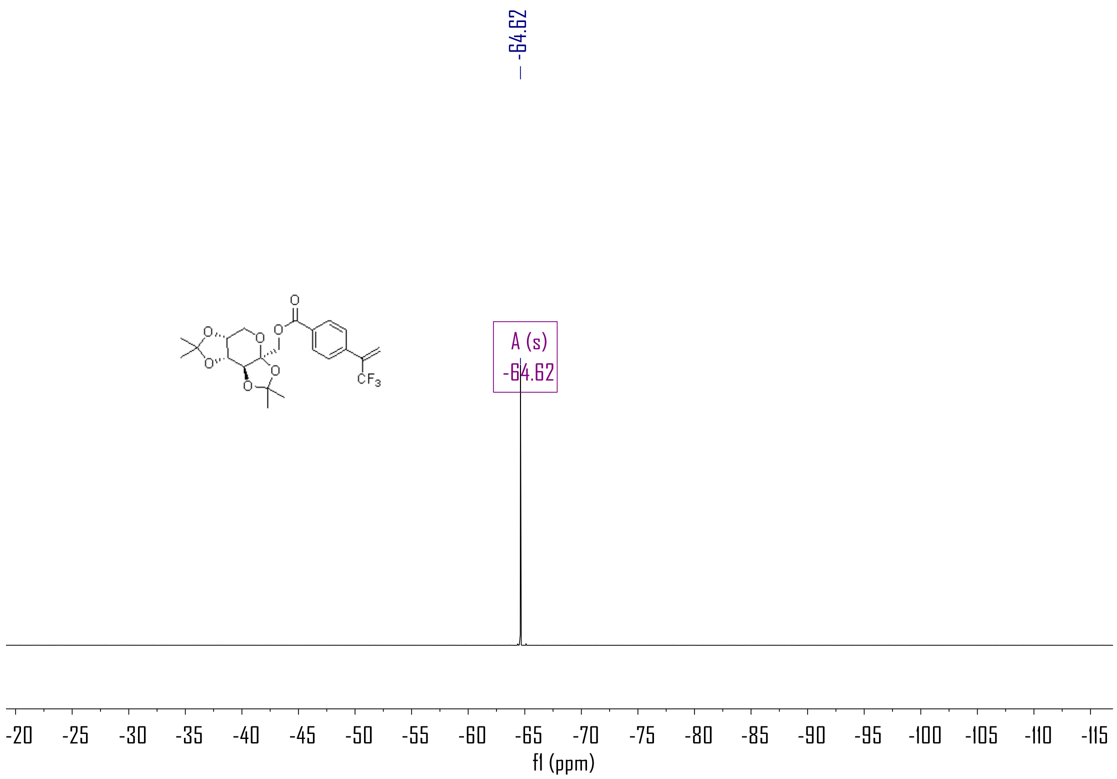

^{19}F NMR spectra for **1t**.

2.2. Preparation of NHPI Esters

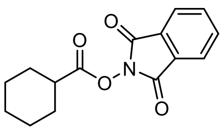

2a

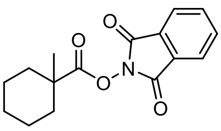

2b

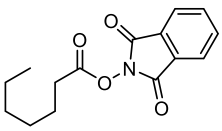

2c

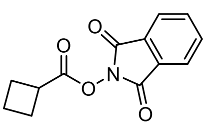

2d

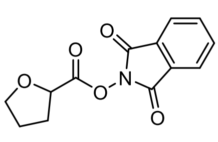

2e

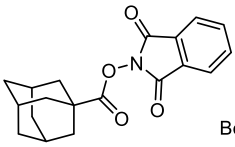

2f

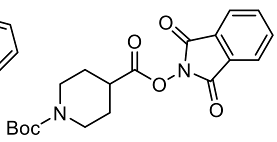

2g

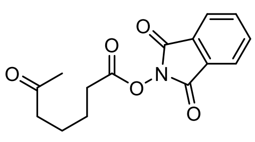

2h

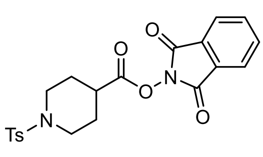

2i

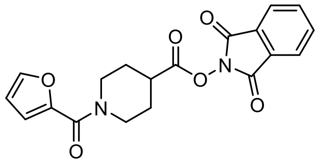

2j

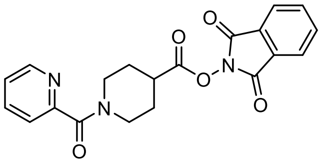

2k

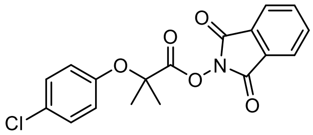

2l

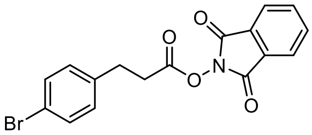

2m

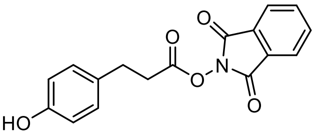

2n

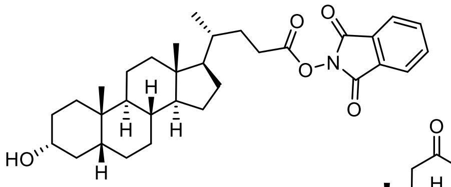

2o

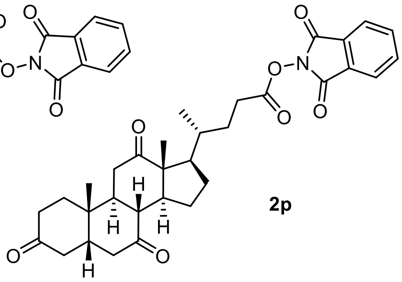

2p

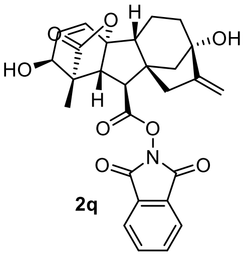

2q

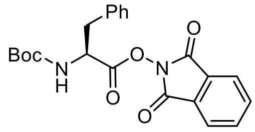

2r

List of NHPI esters.

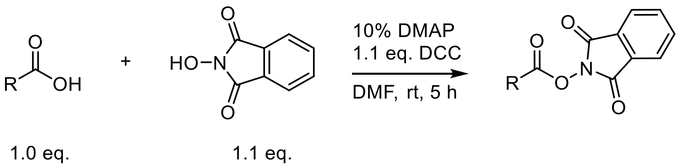

Preparation of NHPI esters using carboxylic acids and *N*-hydroxyphthalimide.

According to the reported literature, NHPI esters (**2a**-**2r**) were conveniently synthesized in gram scale.[7] To a solution of carboxylic acid (1.0 equiv., 10 mmol), 4-dimethylaminopyridine (DMAP) (10 mol%, 1 mmol, 122 mg) and *N*-

hydroxyphthalimide (1.1 equiv., 11 mmol, 1.79 g) in DMF (25 mL), *N*,*N*'-dicyclohexylcarbodiimide (DCC) (1.1 equiv., 11 mmol, 2.27 g) was added. The reaction mixture was stirred at room temperature for 5 hours (TLC tracking detection). The mixture was purified by column chromatography to afford the corresponding NHPI esters. **2a**,[8] **2b**,[9] **2c**,[10] **2d**,[8] **2e**,[11] **2f**,[11] **2g**,[12] **2h**,[7] **2i**,[7] **2j**,[7] **2k**,[7] **2l**,[13] **2m**,[7] **2n**,[7] **2o**,[7] **2p**,[7] **2r**[14] were known compounds, and the data match the reported.

2.3. Preparation of Alkyl Bromides

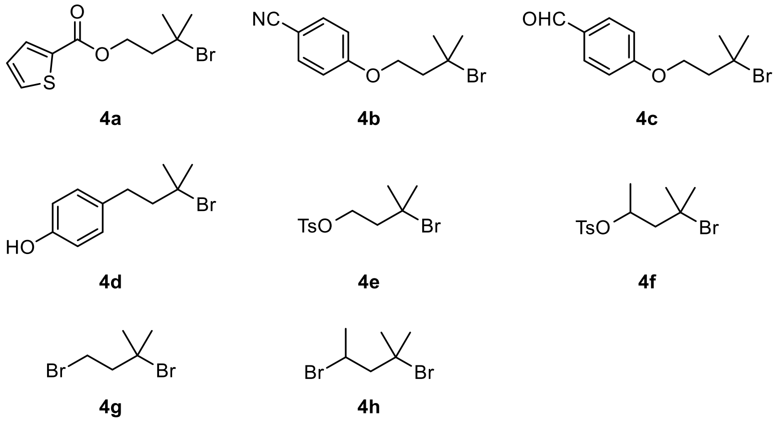

List of alkyl bromides.

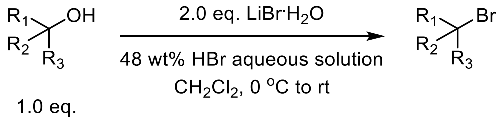

Preparation of alkyl bromides.

According to the reported literature, alkyl bromides (**4a-4f**) were conveniently synthesized in gram scale.[15] To a solution of alcohol (1.0 equiv.) in CH_2Cl_2 (1.0 M) was added LiBr·H_2O (2.0 equiv.) in 48 wt% HBr aqueous solution (1.0 M) at 0 °C. The mixture was allowed to warm to room temperature and stirred for 8-16 hours (TLC tracking detection). The mixture was purified by column chromatography to afford the corresponding alkyl bromide. **4c**,[15] **4d**,[16] **4e**,[15] **4f**[15] were known compounds, and the data match the reported. **4g** was commercial available.

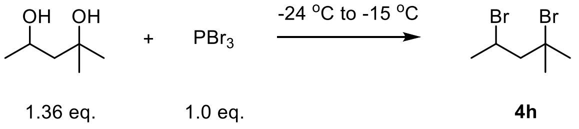

Preparation of alkyl bromide **4h**.

According to the reported literature, **4h** was conveniently synthesized.[17] To a four-necked reaction flask with mechanical stirrer, a thermometer, a calcium chloride tube and a dropping funnel were equipped. To the flask, 2-methyl-2,4-pentanediol (1.36 equiv., 15 mmol, 1.77 g) was added and cooled to -24 °C. Then (1.0 equiv., 11 mmol, 2.98 g) PBr_3 was added dropwise over 1 hour, and the temperature was kept below -15 °C. After the addition of PBr_3, the mixture was purified by column chromatography to afford 2-methyl-2,4-dibromopentane (**4h**).

3. General Procedure for Table 1

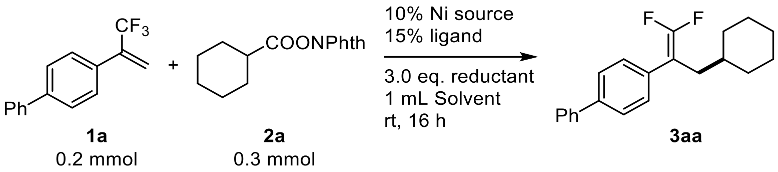

Entry	Nickel source	Ligand	Reductant	Solvent	Yield^{a}/%
1	$NiBr_2$(diglyme)	**L1**	Zn	DMAc	23
2	$NiBr_2$(diglyme)	**L2**	Zn	DMAc	32
3	$NiBr_2$(diglyme)	**L3**	Zn	DMAc	47
4	$NiBr_2$(diglyme)	**L4**	Zn	DMAc	79
5	$NiCl_2$	**L4**	Zn	DMAc	33
6	$Ni(NO_3)_2$	**L4**	Zn	DMAc	<5
7	$Ni(acac)_2$	**L4**	Zn	DMAc	26
8	$NiCl_2(Py)_4$	**L4**	Zn	DMAc	75
9	$NiCl_2(PPh_3)_2$	**L4**	Zn	DMAc	23
10	$NiCl_2(PCy_3)_2$	**L4**	Zn	DMAc	17
11	$NiBr_2$(diglyme)	**L4**	Zn	1,4-Dioxane	<5
12	$NiBr_2$(diglyme)	**L4**	Zn	DME	22
13	$NiBr_2$(diglyme)	**L4**	Zn	THF	43
14	$NiBr_2$(diglyme)	**L4**	Zn	MeCN	<5
15	$NiBr_2$(diglyme)	**L4**	Zn	NMP	54
16	$NiBr_2$(diglyme)	**L4**	Zn	DMF	60
17	***$NiBr_2$(diglyme)***	***L4***	***Zn***	***DMSO***	***95 (92^{b})***
18	$NiBr_2$(diglyme)	**L4**	Mn	DMSO	64
19	$NiBr_2$(diglyme)	**L4**	DEMS/Na_2CO_3	DMSO	18
20	$NiBr_2$(diglyme)	**L4**	$(BPin)_2/K_3PO_4$	DMSO	22

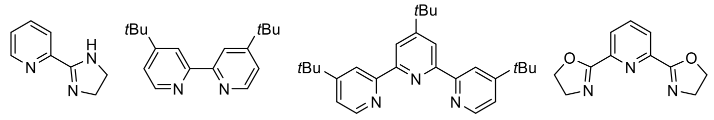

L1 **L2** **L3** **L4**

^{a} GC yield. Triphenylmethane as internal standard. ^{b} Isolated yield. rt = room temperature. NPhth = phthalimide. Diglyme = 2-methoxyethyl ether. acac = Acetylacetone. Py = pyridine. Cy = cyclohexyl. DMAc = *N*,*N*-dimethylacetamide. DME = 1,2-dimethoxyethane. THF = tetrahydrofuran. NMP = 1-methyl-2-pyrrolidinone. DMF = *N*,*N*-dimethylformamide. DMSO =

dimethyl sulfoxide. DEMS = diethoxymethylsilane. $(BPin)_2$ = bis(pinacolato)diboron.

Nickel source (0.02 mmol, 10 mol%), ligand (0.03 mmol, 15 mol%) and reductant (0.6 mmol, 3.0 equiv.) were added to a Schlenk tube equipped with a stir bar. The Schlenk tube was evacuated and filled with argon (three cycles). To these solids, 1 mL solvent was added under argon atmosphere. Then, **1a** (0.2 mmol, 1.0 equiv.) and **2a** (0.3 mmol, 1.5 equiv.) were added and stirred at room temperature (~20 °C) for 16 hours. The yield was determined by GC with triphenylmethane as internal standard.

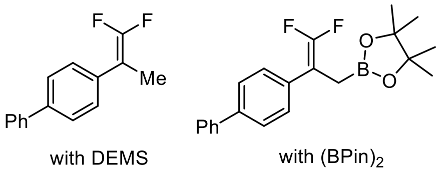

Major by-products

4. General Procedure for the Defluorinative Reductive Cross-Coupling

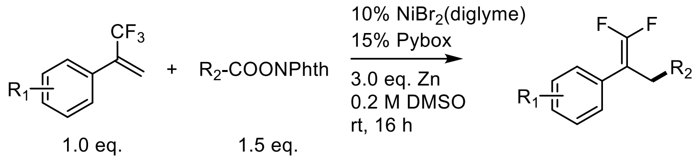

General procedure for the defluorinative reductive cross-coupling.

NiBr$_2$(diglyme) (0.02 mmol, 10 mol%, 7.1 mg), Pybox (0.03 mmol, 15 mol%, 6.5 mg) and Zn (0.6 mmol, 3.0 equiv., 39 mg) were added to a Schlenk tube equipped with a stir bar. The Schlenk tube was evacuated and filled with argon (three cycles). To these solids, 1 mL DMSO (0.2 M) was added under argon atmosphere. Then, trifluoromethyl alkene (0.2 mmol, 1.0 equiv.) and redox-active ester (0.3 mmol, 1.5 equiv.) were added and stirred at room temperature (~20 ℃) for 16 hours. The mixture was purified by column chromatography to afford the desired product.

5. Examples Described in Table 2

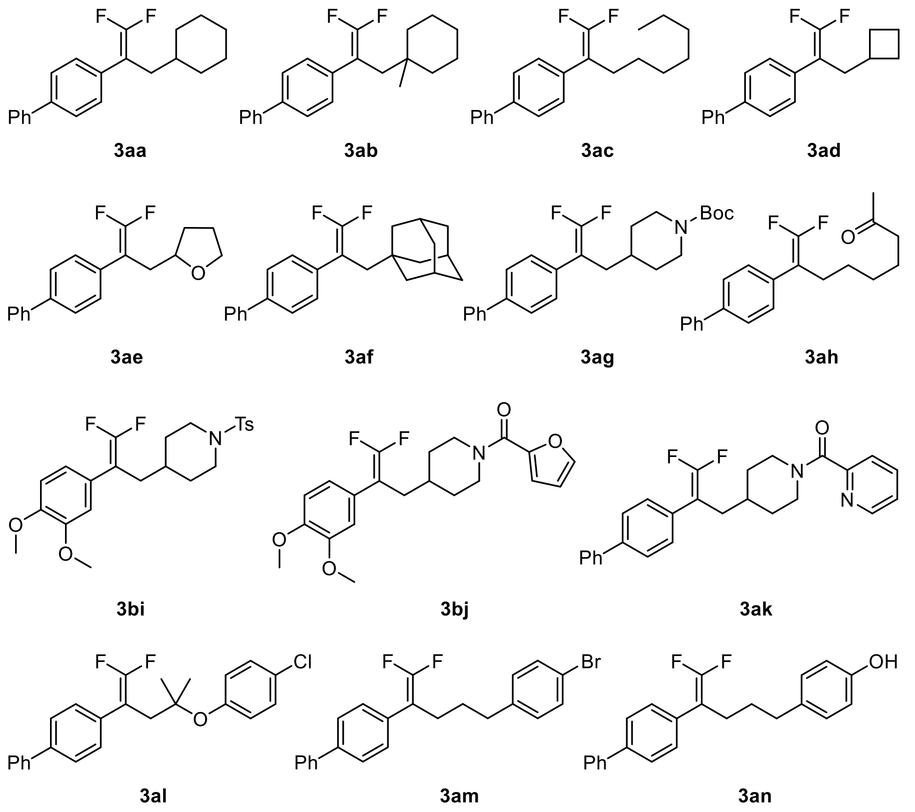

List of substrates in Table 2.

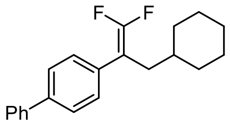

4-(3-cyclohexyl-1,1-difluoroprop-1-en-2-yl)-1,1'-biphenyl (**3aa**)

Following general procedure, **1a** and **2a** were used. The product was isolated by column chromatography as white solid (57.5 mg, 0.184 mmol, 92%).

Selectivity (desired C-F cleavage product : addition by-product) > 50:1.

R_f (petroleum ether) = 0.75.

^{1}H NMR (400 MHz, Chloroform-*d*) δ 7.69 – 7.59 (m, 4H), 7.52 – 7.34 (m, 5H), 2.40 – 2.30 (m, 2H), 1.87 – 1.61 (m, 5H), 1.45 – 1.28 (m, 1H), 1.27 – 1.10 (m, 3H), 1.07 – 0.91 (m, 2H).

^{13}C NMR (101 MHz, Chloroform-*d*) δ 154.23 (dd, *J* = 290.7, 286.3 Hz), 140.72, 139.99, 133.21 (dd, *J* = 4.6, 4.2 Hz), 128.92, 128.75 (dd, *J* = 3.4, 3.4 Hz), 127.48, 127.18, 127.13, 90.94 (dd, *J* = 22.3, 12.3 Hz), 35.90 (dd, *J* = 2.5, 2.5 Hz), 35.27, 33.04, 26.57, 26.21.

^{19}F NMR (376 MHz, Chloroform-*d*) δ -90.64 (d, *J* = 43.4 Hz), -91.19 (d, *J* = 43.3 Hz).

HRMS (EI) calcd for $C_{21}H_{22}F_2^+$ [M^+] 312.16841, found 312.16798.

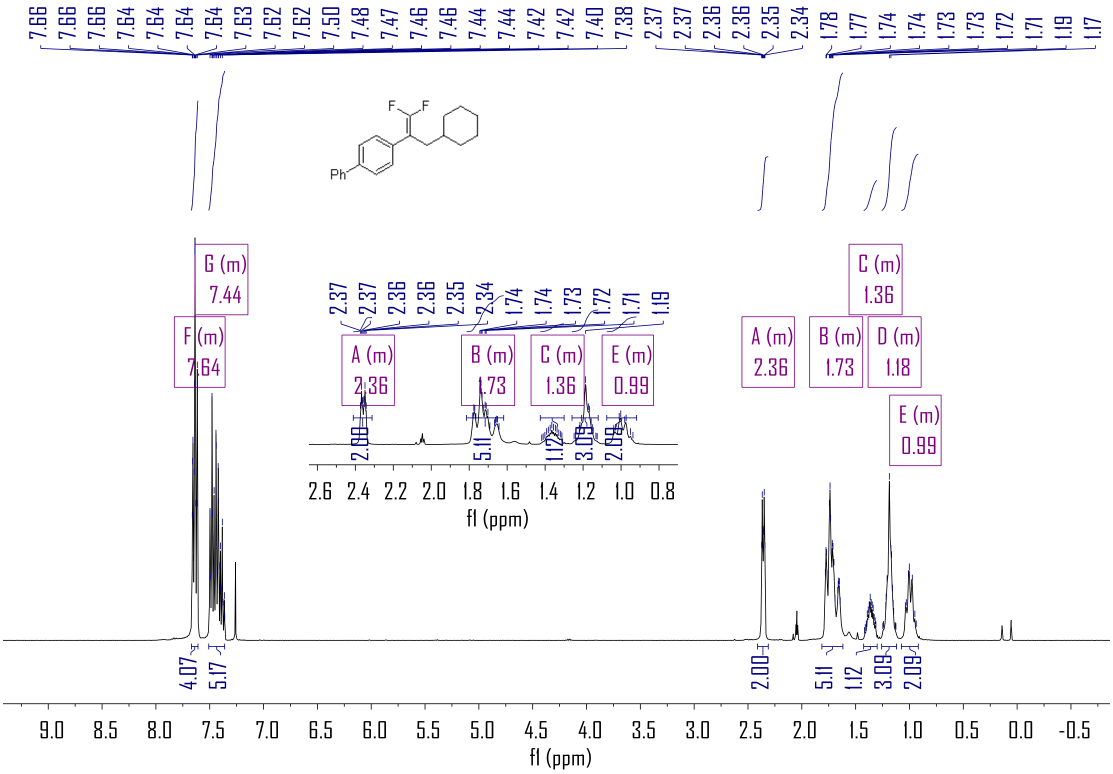

^{1}H NMR spectra for **3aa**.

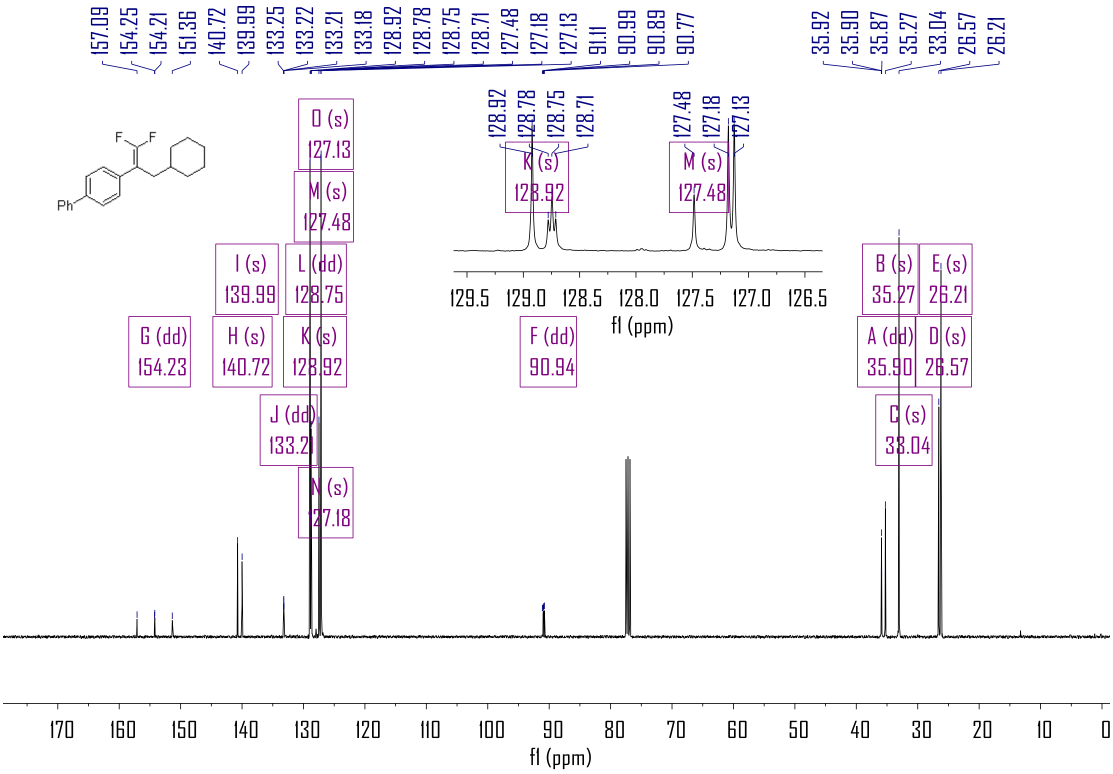

^{13}C NMR spectra for **3aa**.

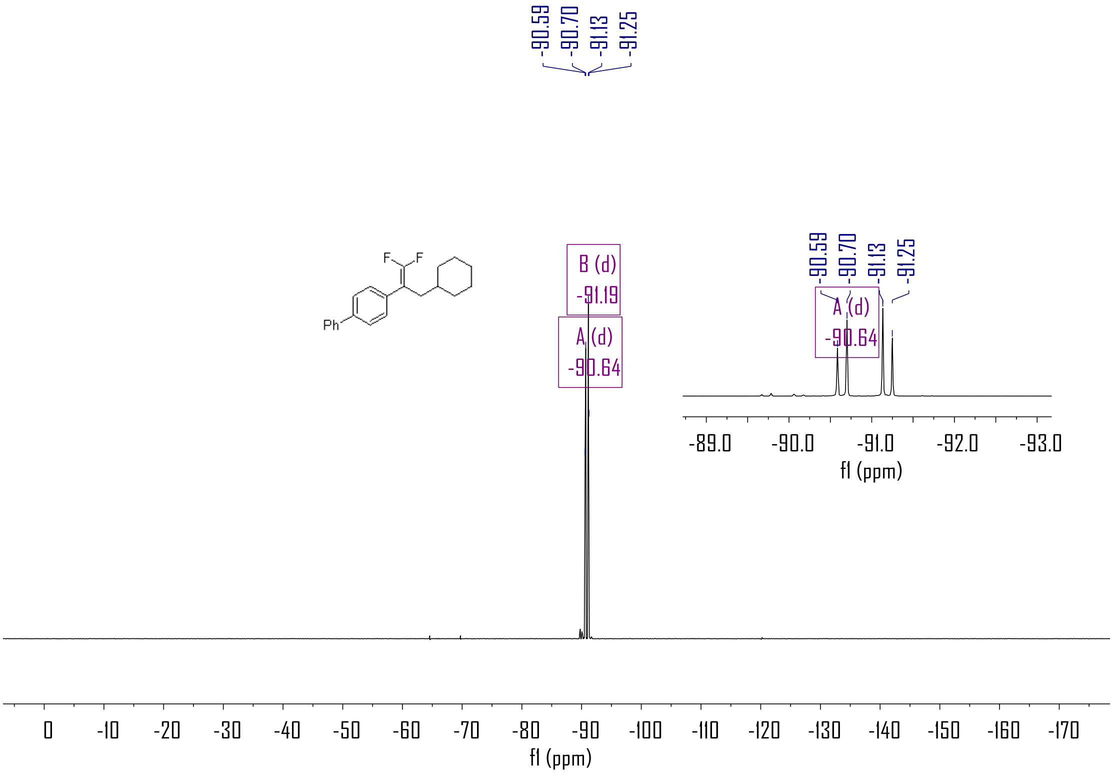

^{19}F NMR spectra for **3aa**.

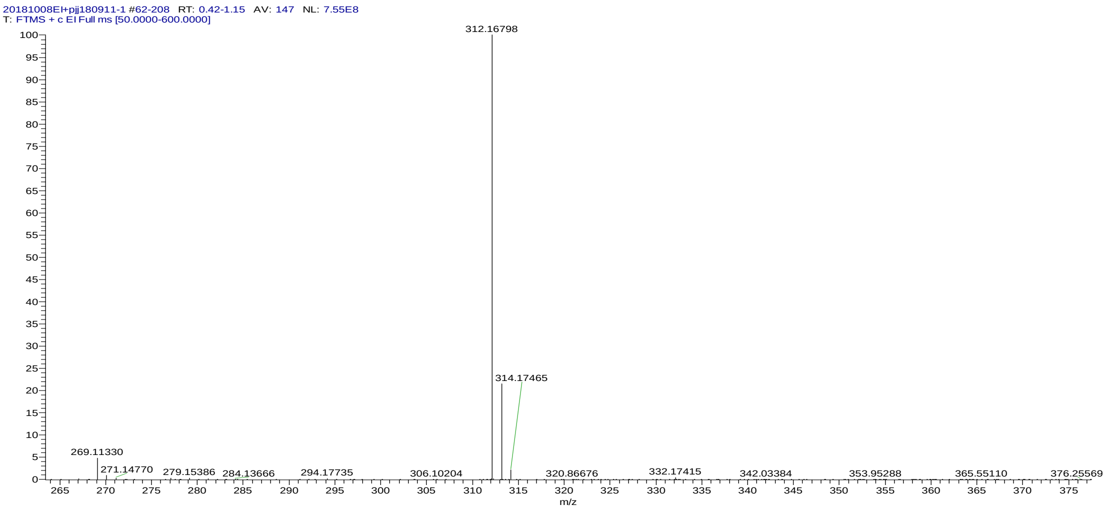

HRMS spectra for **3aa**.

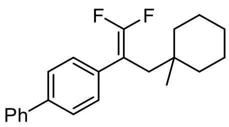

4-(1,1-difluoro-3-(1-methylcyclohexyl)prop-1-en-2-yl)-1,1'-biphenyl (**3ab**)

Following general procedure, **1a** and **2b** were used. The product was isolated by column chromatography as white solid (59.4 mg, 0.182 mmol, 91%).

<u>Selectivity (desired C-F cleavage product : addition by-product)</u> > 50:1.

R$_f$ (petroleum ether) = 0.80.

^{1}H NMR (400 MHz, Chloroform-*d*) δ 7.70 – 7.57 (m, 4H), 7.54 – 7.33 (m, 5H), 2.45 – 2.42 (m, 2H), 1.53 – 1.33 (m, 5H), 1.33 – 1.17 (m, 5H), 0.84 (s, 3H).

^{13}C NMR (101 MHz, Chloroform-*d*) δ 154.55 (dd, *J* = 290.0, 287.7 Hz), 140.70, 139.77, 134.98 (dd, *J* = 4.4, 2.9 Hz), 128.97 (dd, *J* = 2.6, 2.6 Hz), 128.90, 127.45, 127.09, 126.98, 90.46 (dd, *J* = 21.7, 12.9 Hz), 40.36, 38.17, 35.37 (dd, *J* = 2.5, 2.5 Hz), 26.45, 24.82, 22.16.

^{19}F NMR (376 MHz, Chloroform-*d*) δ -88.94 (d, *J* = 40.5 Hz), -91.68 (d, *J* = 40.2 Hz).

HRMS (EI) calcd for $C_{22}H_{24}F_2^+$ [M$^+$] 326.18406, found 326.18411.

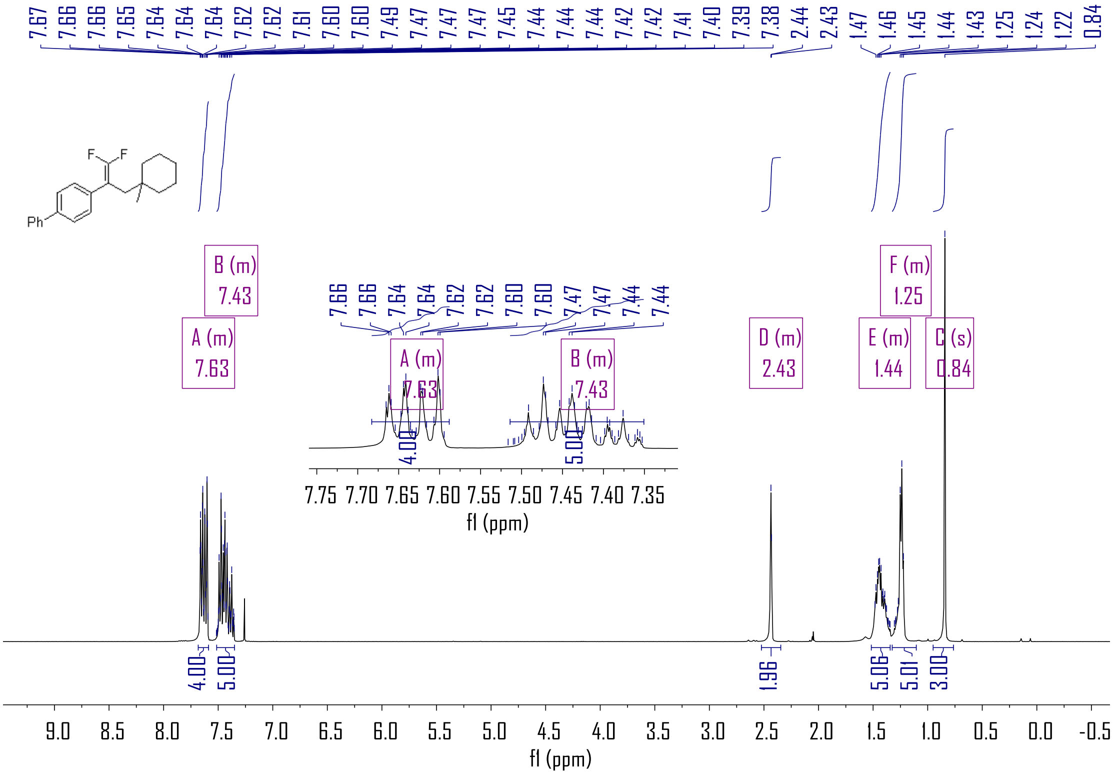

^{1}H NMR spectra for **3ab**.

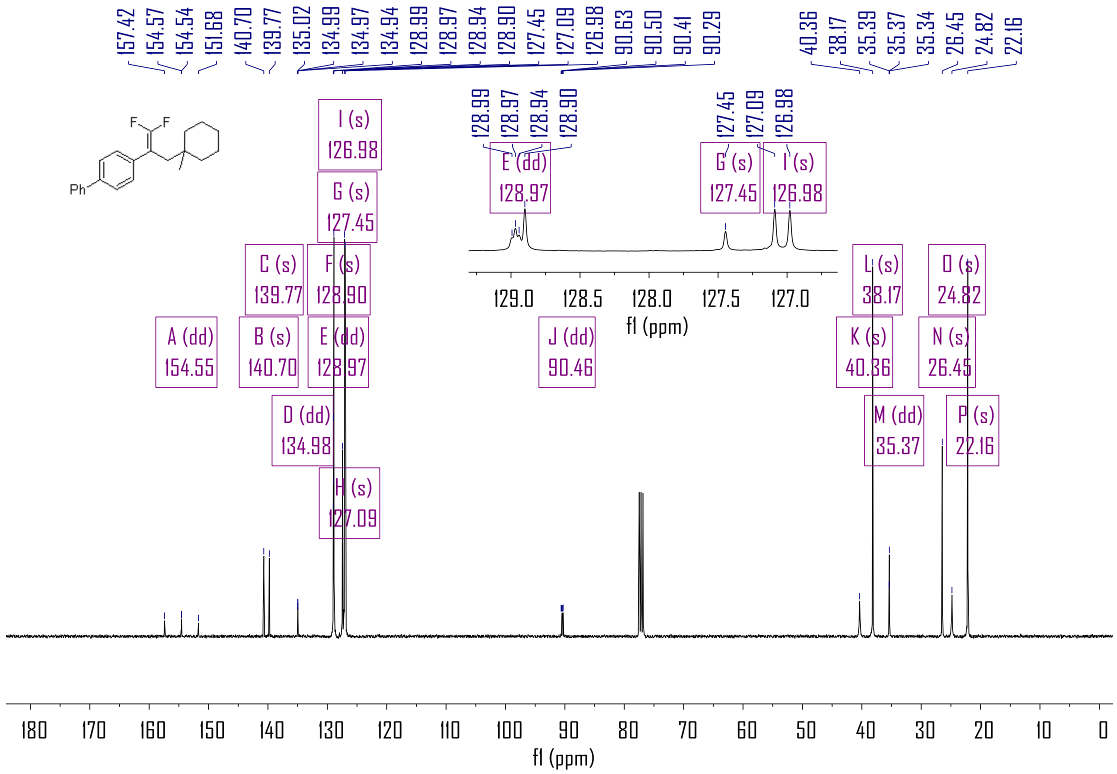

^{13}C NMR spectra for **3ab**.

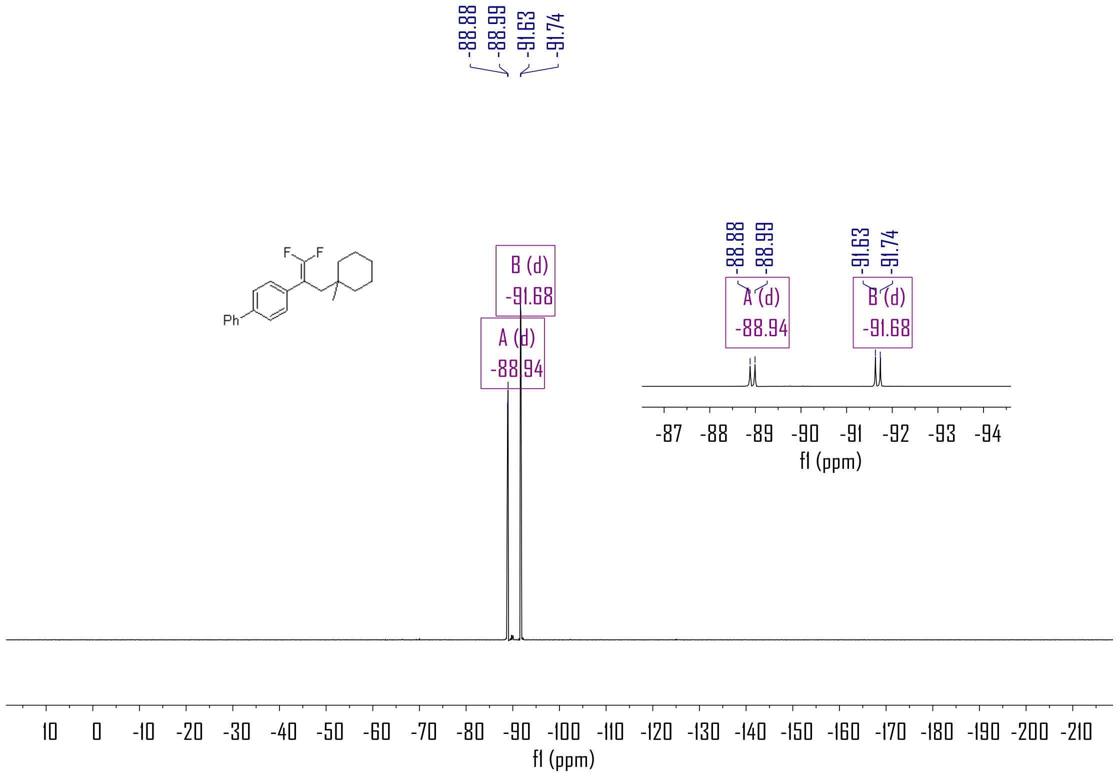

^{19}F NMR spectra for **3ab**.

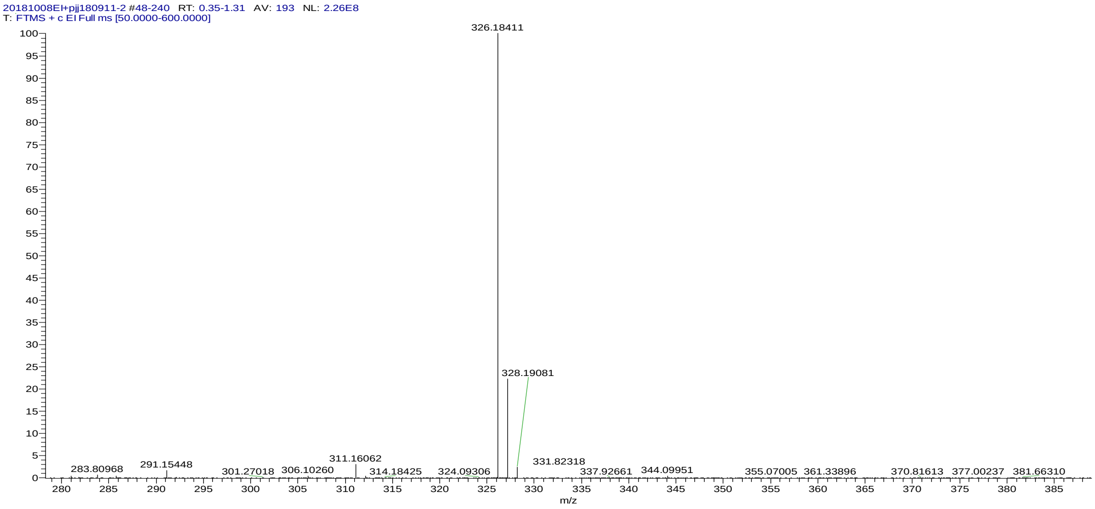

HRMS spectra for **3ab**.

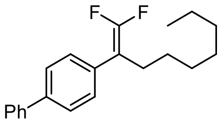

4-(1,1-difluoronon-1-en-2-yl)-1,1'-biphenyl (**3ac**)

Following general procedure, **1a** and **2c** were used. The product was isolated by column chromatography as colorless oil (51.6 mg, 0.164 mmol, 82%).

Selectivity (desired C-F cleavage product : addition by-product) > 50:1.

R_f (petroleum ether) = 0.85.

^{1}H NMR (400 MHz, Chloroform-*d*) δ 7.68 – 7.57 (m, 4H), 7.53 – 7.33 (m, 5H), 2.45 (ddd, *J* = 10.2, 6.0, 2.4 Hz, 2H), 1.49 – 1.38 (m, 2H), 1.38 – 1.20 (m, 8H), 0.90 (t, *J* = 7.6 Hz, 3H).

^{13}C NMR (101 MHz, Chloroform-*d*) δ 153.81 (dd, *J* = 289.8, 287.2 Hz), 140.76, 140.06, 132.99 (dd, *J* = 2.9, 2.0 Hz), 128.93, 128.71 (dd, *J* = 3.4, 3.4 Hz), 127.49, 127.20, 127.14, 92.34 (dd, *J* = 20.6, 13.8 Hz), 31.94, 29.17, 29.12, 27.99 (dd, *J* = 2.5, 2.5 Hz), 27.68, 22.77, 14.22.

^{19}F NMR (376 MHz, Chloroform-*d*) δ -91.32 (dt, *J* = 44.1, 2.4 Hz), -91.48 (d, *J* = 44.0 Hz).

HRMS (EI) calcd for $C_{21}H_{24}F_2^+$ [M^+] 314.18406, found 314.18372.

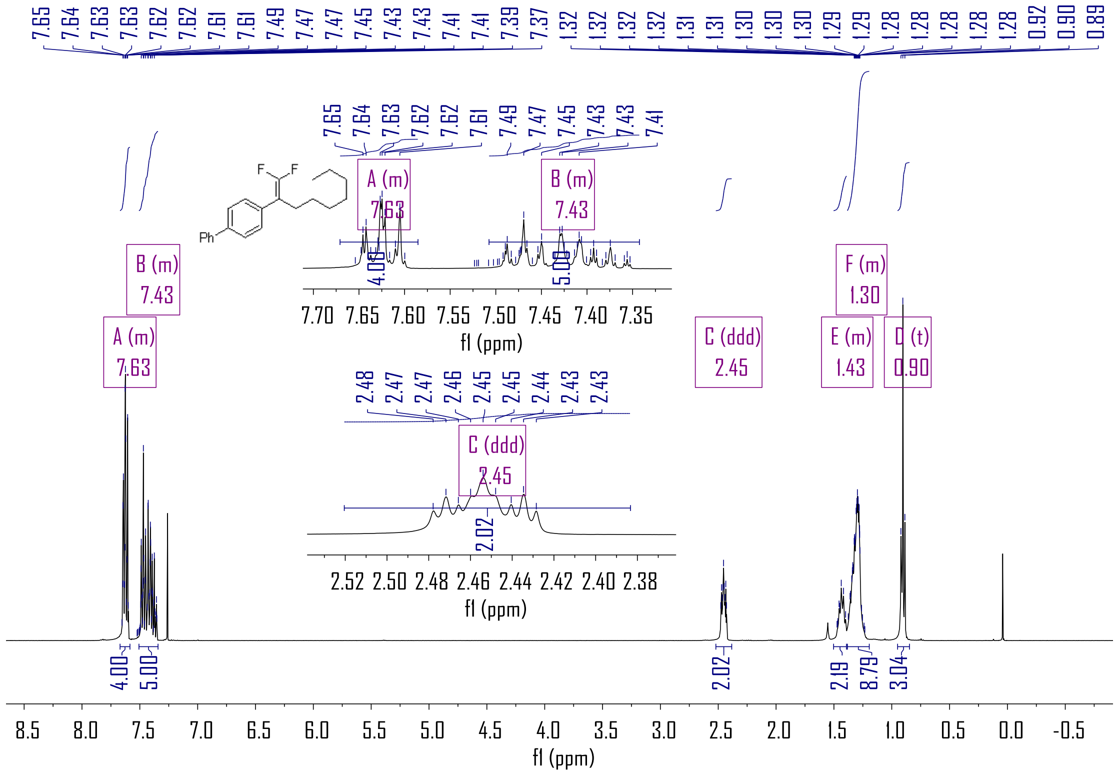

^{1}H NMR spectra for **3ac**.

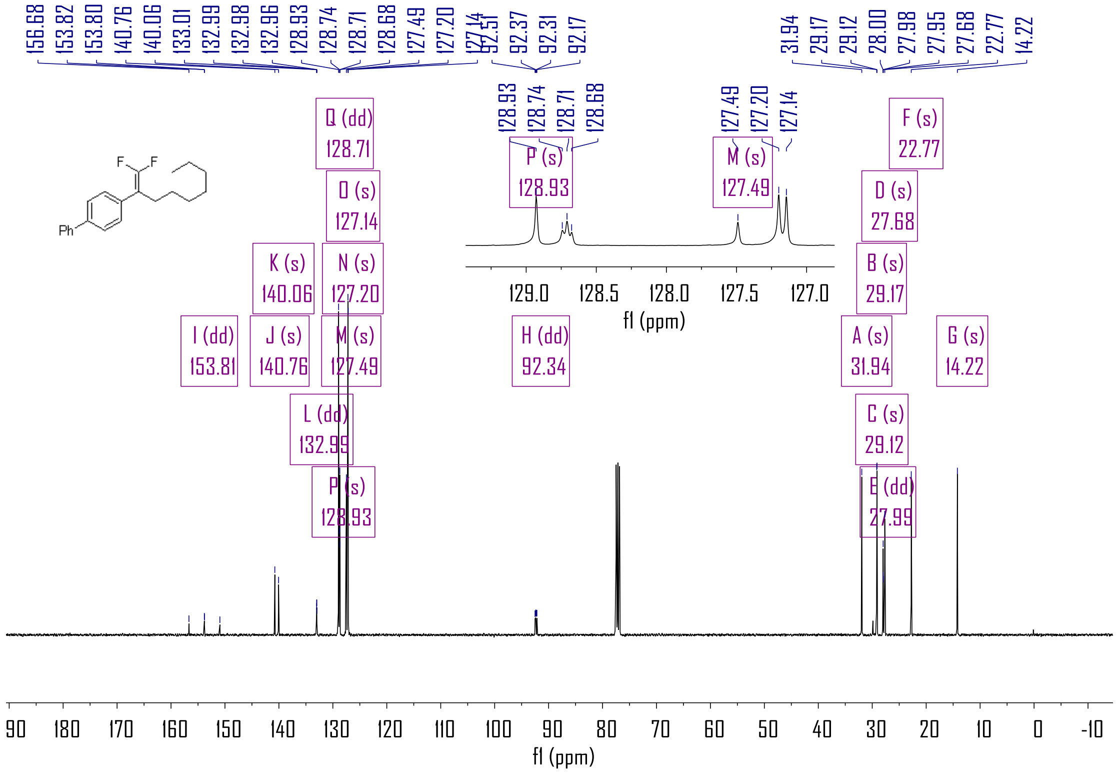

^{13}C NMR spectra for **3ac**.

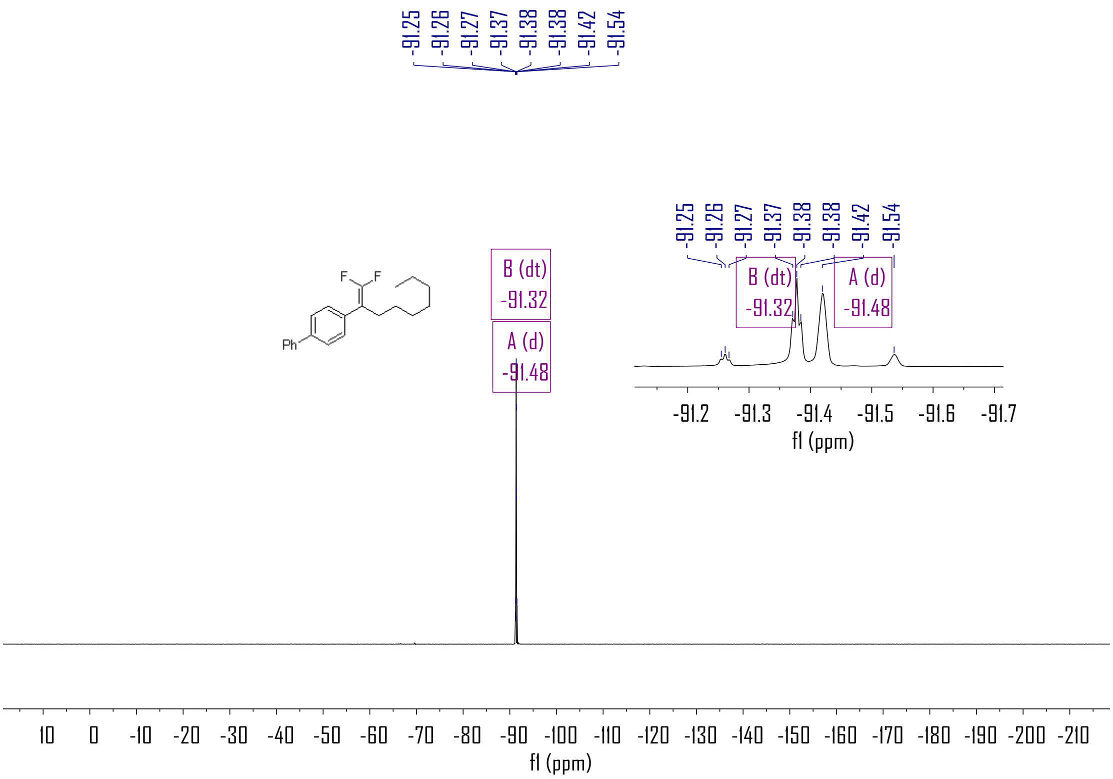

^{19}F NMR spectra for **3ac**.

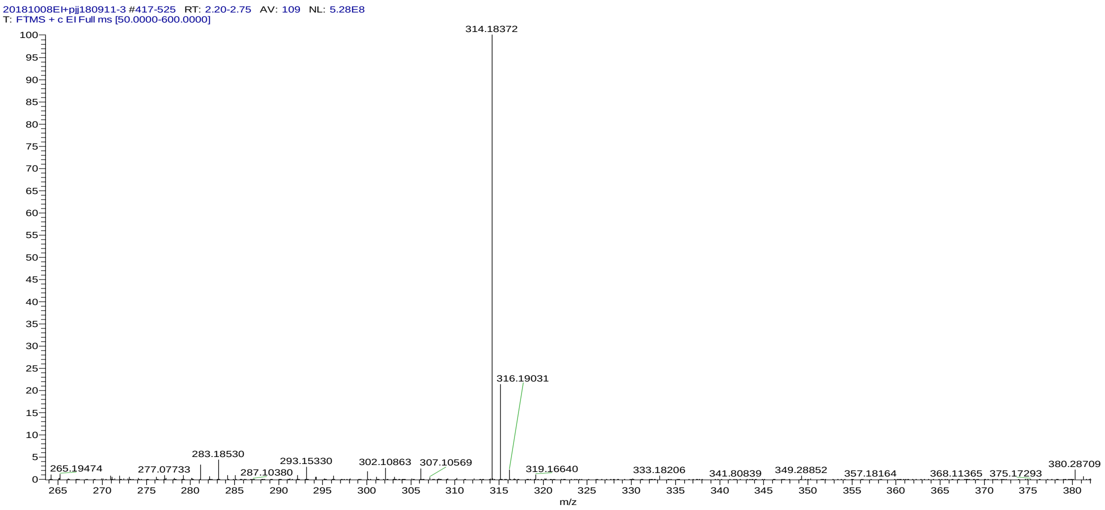

HRMS spectra for **3ac**.

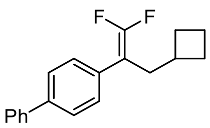

4-(3-cyclobutyl-1,1-difluoroprop-1-en-2-yl)-1,1'-biphenyl (**3ad**)

Following general procedure, **1a** and **2d** were used. The product was isolated by column chromatography as white solid (53.5 mg, 0.188 mmol, 94%)

Selectivity (desired C-F cleavage product : addition by-product) > 50:1.

R_f (petroleum ether) = 0.82.

^{1}H NMR (400 MHz, Chloroform-*d*) δ 7.68 – 7.57 (m, 4H), 7.52 – 7.43 (m, 2H), 7.43 – 7.34 (m, 3H), 2.58 – 2.52 (m, 2H), 2.39 (hept, *J* = 7.7 Hz, 1H), 2.10 – 1.95 (m, 2H), 1.88 – 1.78 (m, 2H), 1.76 – 1.63 (m, 2H).

^{13}C NMR (101 MHz, Chloroform-*d*) δ 154.21 (dd, *J* = 289.8, 286.6 Hz), 140.72, 140.07, 133.05 (dd, *J* = 3.8, 2.3 Hz), 128.92, 128.80 (dd, *J* = 3.2, 3.2 Hz), 127.48, 127.13, 91.14 (dd, *J* = 21.2, 13.7 Hz), 34.67, 34.43 (dd, *J* = 2.5, 2.5 Hz), 28.07, 18.29.

^{19}F NMR (376 MHz, Chloroform-*d*) δ -91.52 (d, *J* = 44.6 Hz), -91.72 (d, *J* = 44.6 Hz).

HRMS (EI) calcd for $C_{19}H_{18}F_2^+$ [M$^+$] 284.13711, found 284.13681.

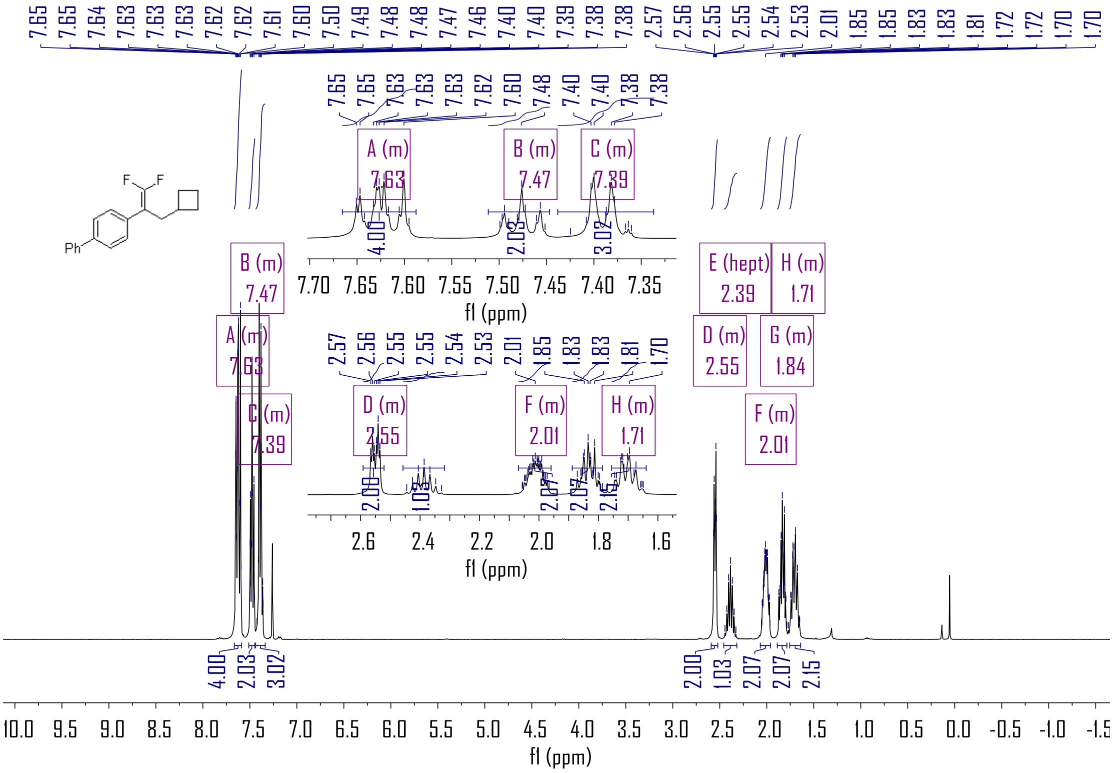

^{1}H NMR spectra for **3ad**.

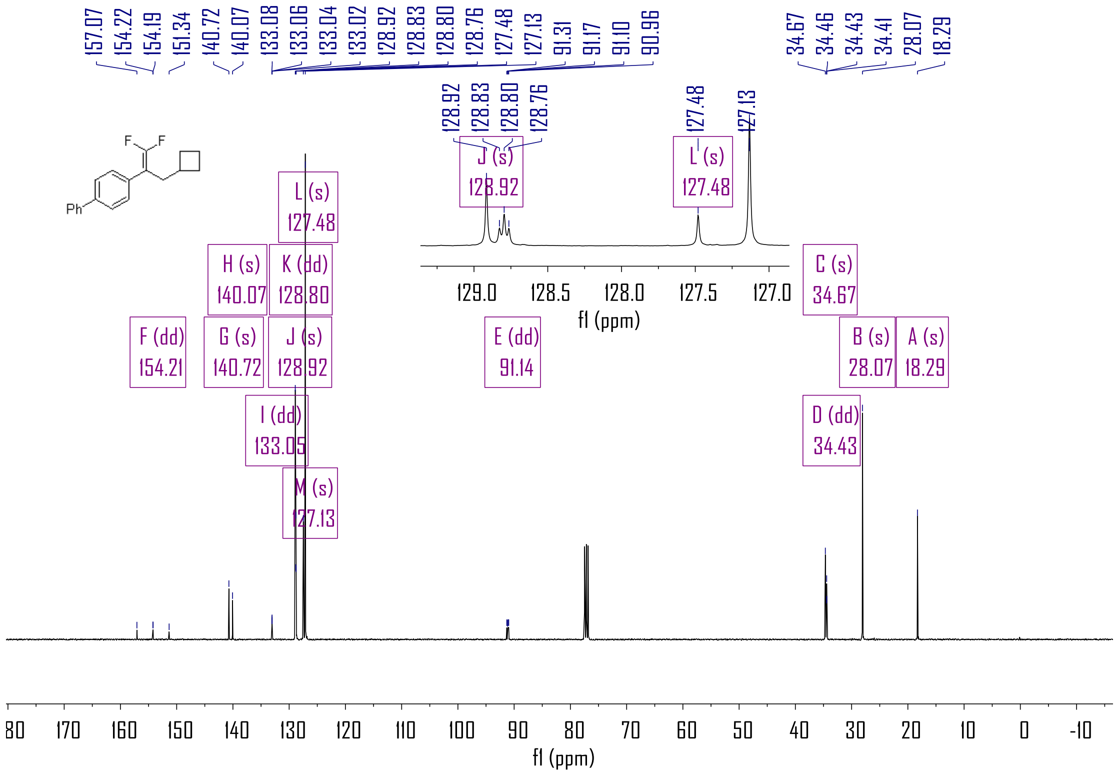

^{13}C NMR spectra for **3ad**.

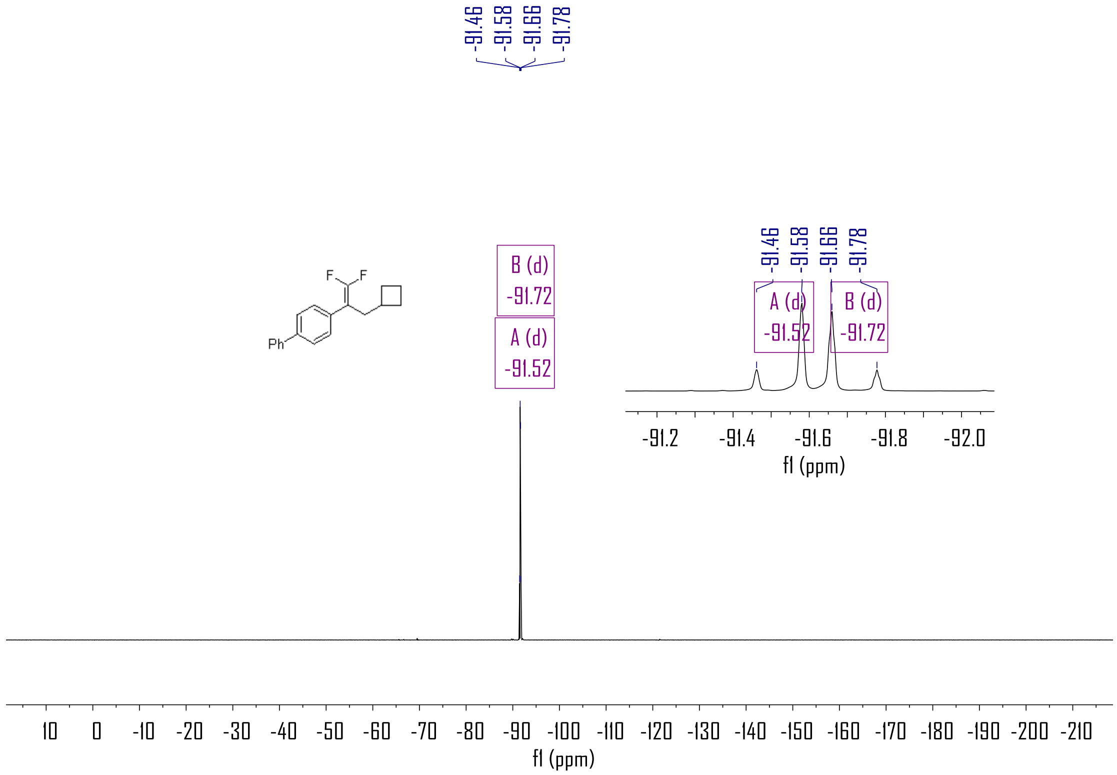

^{19}F NMR spectra for **3ad**.

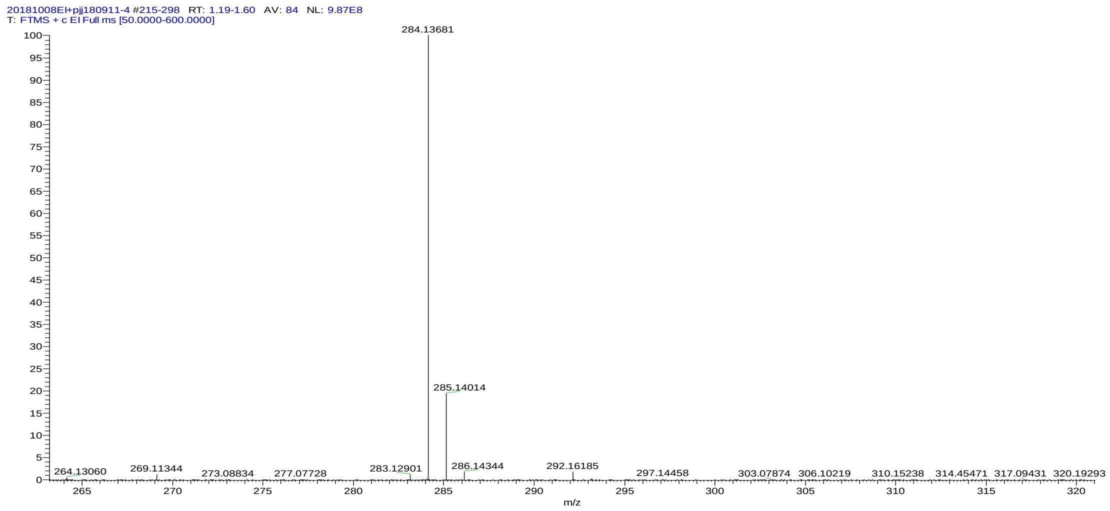

HRMS spectra for **3ad**.

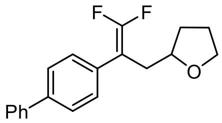

2-(2-([1,1'-biphenyl]-4-yl)-3,3-difluoroallyl)tetrahydrofuran (**3ae**)

Following general procedure, **1a** and **2e** were used. The product was isolated by column chromatography as white solid (55.8 mg, 0.186 mmol, 93%).

Selectivity (desired C-F cleavage product : addition by-product) > 50:1.

R_f (petroleum ether : ethyl acetate = 50:1) = 0.27.

^{1}H NMR (400 MHz, Chloroform-*d*) δ 7.67 – 7.56 (m, 4H), 7.51 – 7.42 (m, 4H), 7.41 – 7.32 (m, 1H), 3.95 – 3.84 (m, 2H), 3.78 – 3.68 (m, 1H), 2.82 – 2.71 (m, 1H), 2.63 – 2.50 (m, 1H), 2.04 – 1.89 (m, 2H), 1.89 – 1.74 (m, 1H), 1.67 – 1.49 (m, 1H).

^{13}C NMR (101 MHz, Chloroform-*d*) δ 154.45 (dd, *J* = 288.3, 288.3 Hz), 140.61, 140.19, 132.56, 128.90, 128.78 (dd, *J* = 3.3, 3.3 Hz), 127.50, 127.22, 127.10, 89.91 (dd, *J* = 19.2, 16.8 Hz), 77.13 (dd, *J* = 3.0, 3.0 Hz), 67.92, 33.85, 31.06, 25.69.

^{19}F NMR (376 MHz, Chloroform-*d*) δ -90.12.

HRMS (APCI) calcd for $C_{19}H_{19}OF_2^+$ [$(M+H)^+$] 301.13985, found 301.13922.

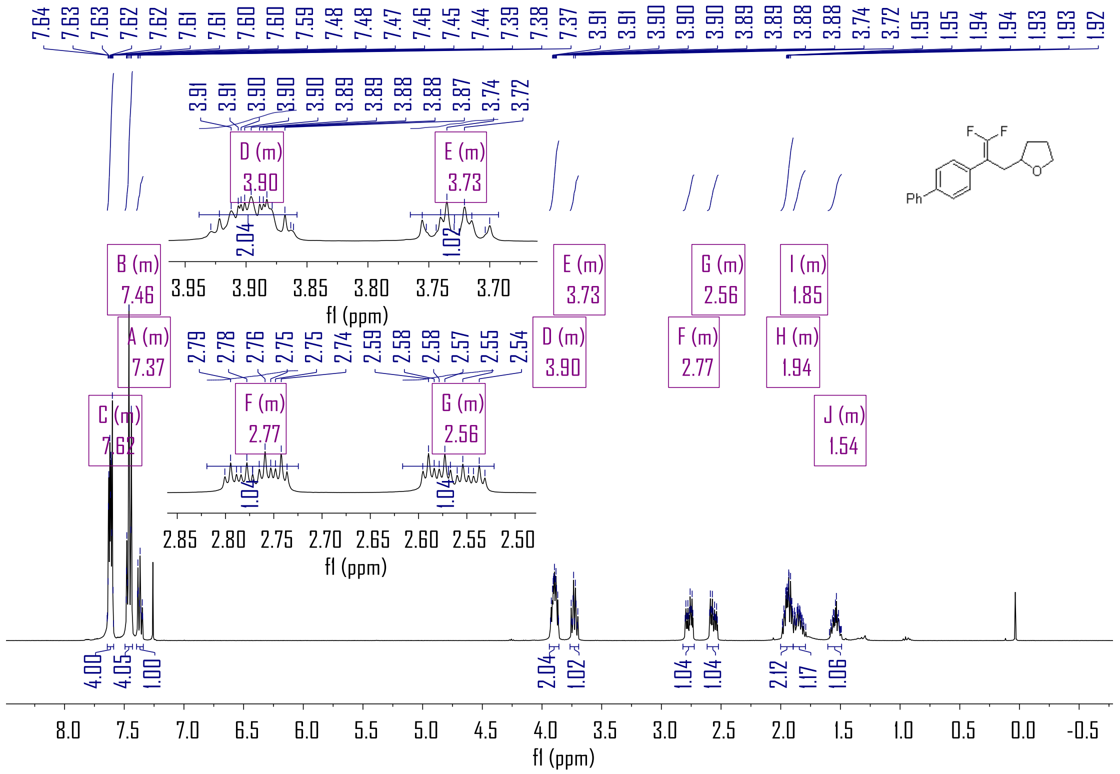

^{1}H NMR spectra for **3ae**.

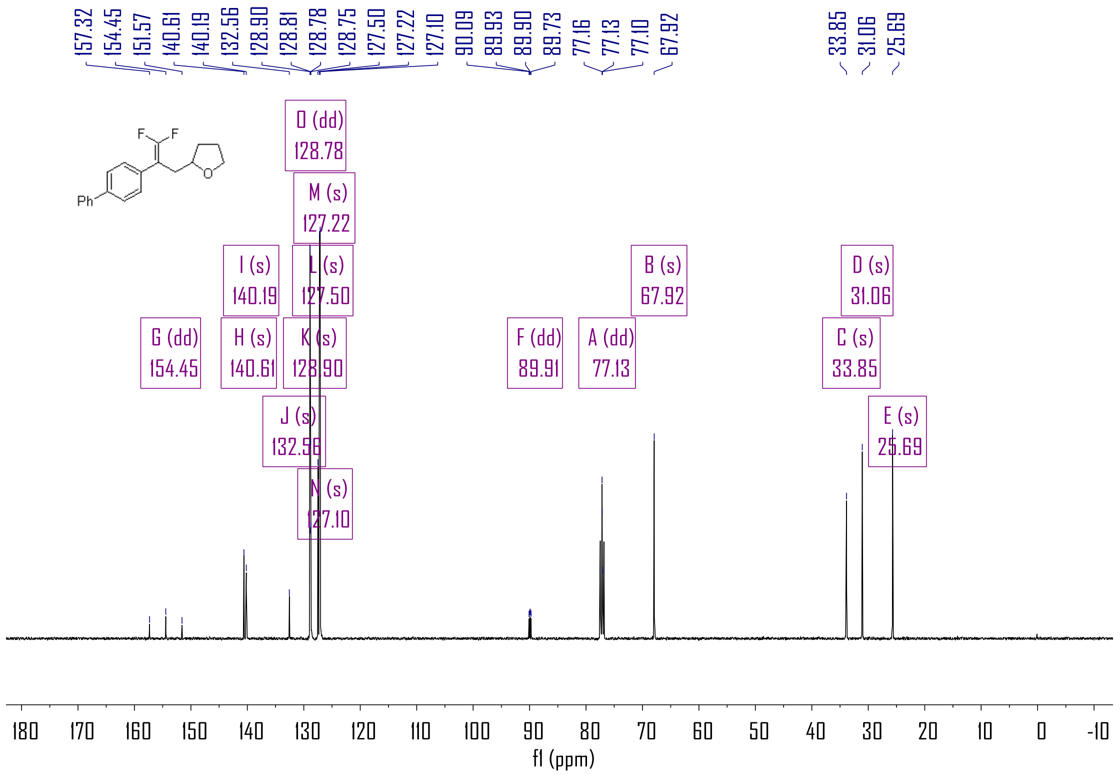

^{13}C NMR spectra for **3ae**.

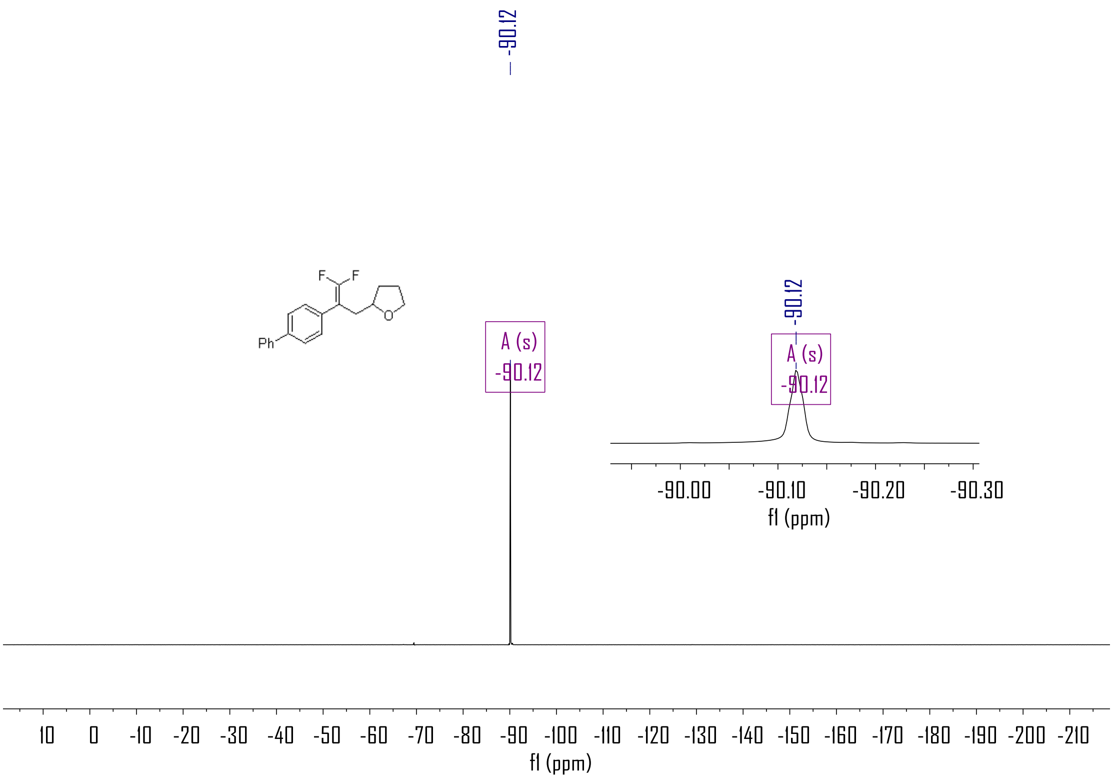

^{19}F NMR spectra for **3ae**.

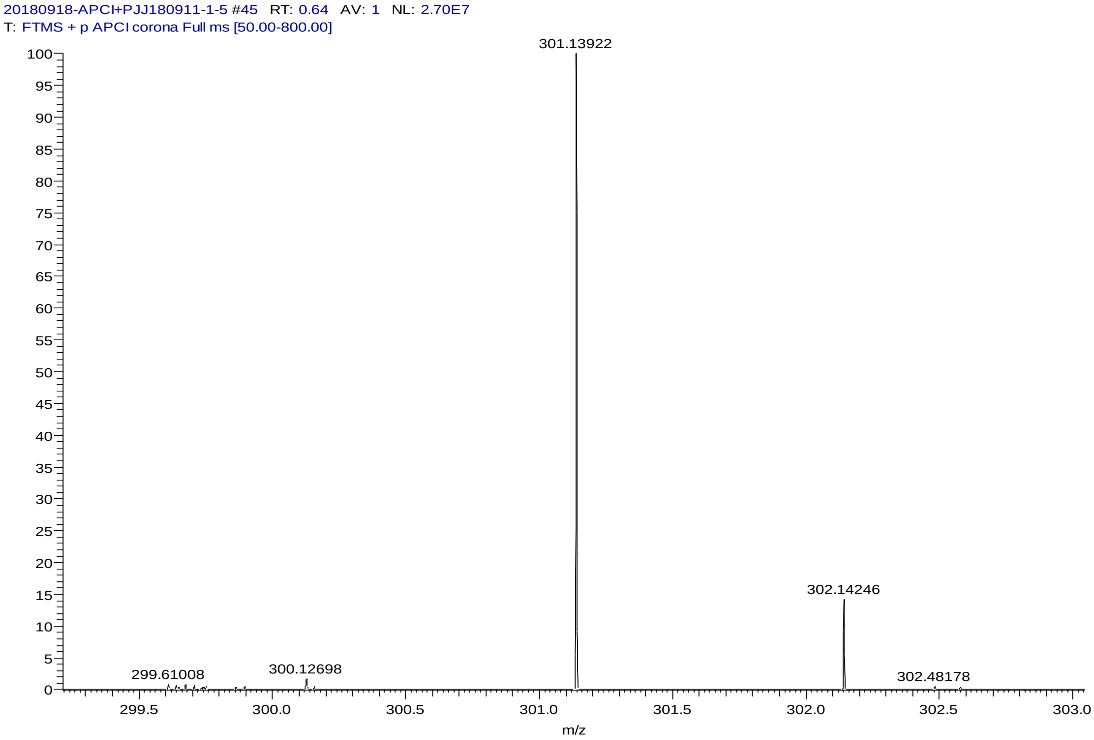

HRMS spectra for **3ae**.

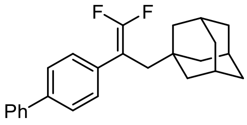

(3*r*,5*r*,7*r*)-1-(2-([1,1'-biphenyl]-4-yl)-3,3-difluoroallyl)adamantane (**3af**)

Following general procedure, **1a** and **2f** were used. The product was isolated by column chromatography as white solid (69.3 mg, 0.190 mmol, 95%).

Selectivity (desired C-F cleavage product : addition by-product) > 50:1.

R_f (petroleum ether) = 0.84.

^{1}H NMR (400 MHz, Chloroform-*d*) δ 7.69 – 7.57 (m, 4H), 7.51 – 7.40 (m, 4H), 7.42 – 7.33 (m, 1H), 2.32 – 2.25 (m, 2H), 1.92 (s, 3H), 1.73 – 1.57 (m, 6H), 1.49 – 1.43 (m, 6H).

^{13}C NMR (101 MHz, Chloroform-*d*) δ 154.56 (dd, *J* = 290.9, 287.3 Hz), 140.65, 139.64, 134.98 (dd, *J* = 4.7, 3.0 Hz), 128.89, 128.80 (dd, *J* = 3.0, 3.0 Hz), 127.43, 127.07, 126.98, 89.59 (dd, *J* = 22.0, 12.2 Hz), 42.80, 41.90, 37.00, 34.81 (dd, *J* = 2.4, 2.4 Hz), 28.73.

^{19}F NMR (376 MHz, Chloroform-*d*) δ -88.42 (d, *J* = 40.5 Hz), -91.57 (d, *J* = 39.3 Hz).

HRMS (EI) calcd for $C_{25}H_{26}F_2^+$ [M^+] 364.19971, found 364.19945.

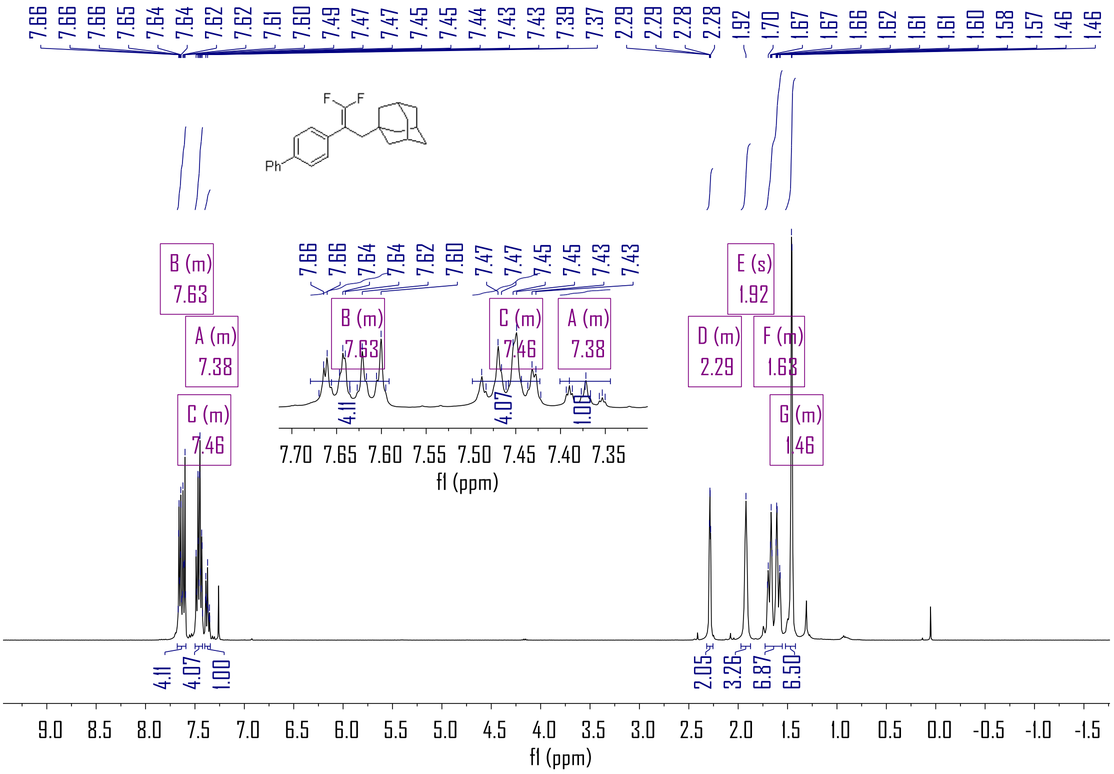

^{1}H NMR spectra for **3af**.

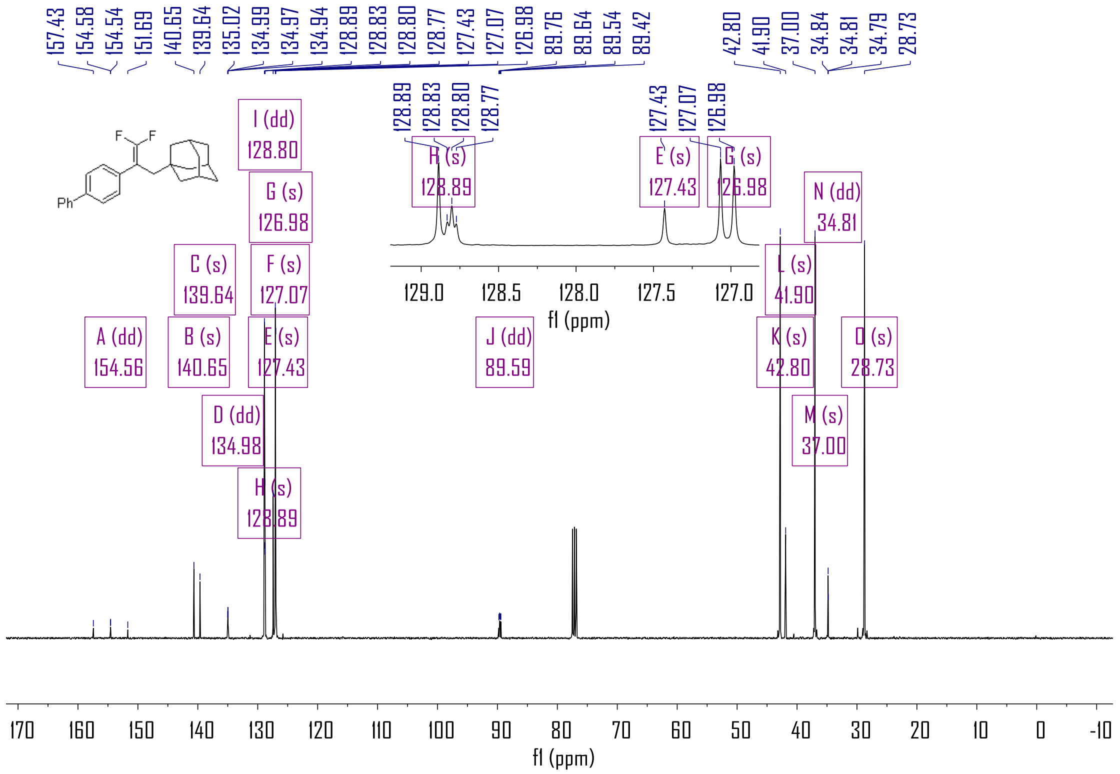

^{13}C NMR spectra for **3af**.

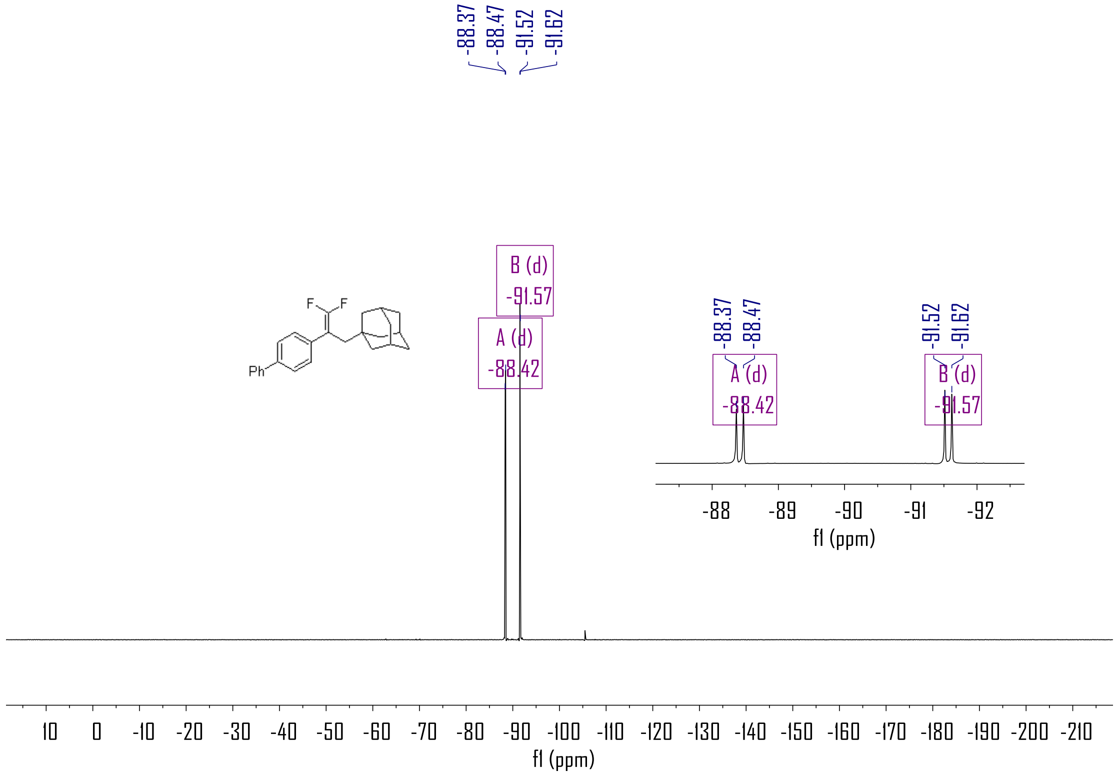

^{19}F NMR spectra for **3af**.

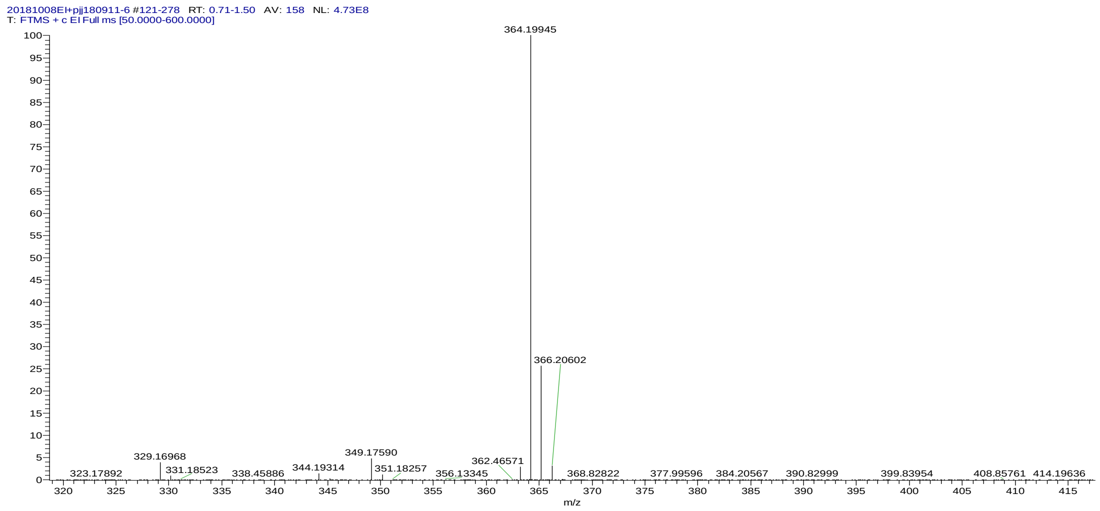

HRMS spectra for **3af**.

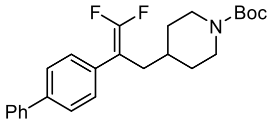

tert-butyl 4-(2-([1,1'-biphenyl]-4-yl)-3,3-difluoroallyl)piperidine-1-carboxylate (**3ag**)

Following general procedure, **1a** and **2g** were used. The product was isolated by column chromatography as colorless oil (80.2 mg, 0.194 mmol, 97%).

Selectivity (desired C-F cleavage product : addition by-product) > 50:1.

R_f (petroleum ether : ethyl acetate = 20:1) = 0.39.

^{1}H NMR (400 MHz, Chloroform-*d*) δ 7.65 – 7.57 (m, 4H), 7.50 – 7.41 (m, 2H), 7.44 – 7.31 (m, 3H), 4.07 (d, *J* = 11.1 Hz, 2H), 2.61 (t, *J* = 12.2 Hz, 2H), 2.43 – 2.34 (m, 2H), 1.71 – 1.62 (m, 2H), 1.53 – 1.41 (m, 10H), 1.16 (qd, *J* = 12.3, 4.4 Hz, 2H).

^{13}C NMR (101 MHz, Chloroform-*d*) δ 154.86, 154.23 (dd, *J* = 291.3, 286.9 Hz), 140.51, 140.21, 132.63 (dd, *J* = 3.8, 3.8 Hz), 128.91, 128.62 (dd, *J* = 3.4, 3.4 Hz), 127.54, 127.27, 127.07, 90.34 (dd, *J* = 22.0, 12.9 Hz), 79.36, 43.88 (brs), 34.37, 31.83, 28.55.

^{19}F NMR (376 MHz, Chloroform-*d*) δ -90.06 (d, *J* = 41.9 Hz), -90.53 (d, *J* = 41.8 Hz).

HRMS (ESI) calcd for $C_{25}H_{29}O_2NF_2Na^+$ [$(M+Na)^+$] 436.20586, found 436.20508.

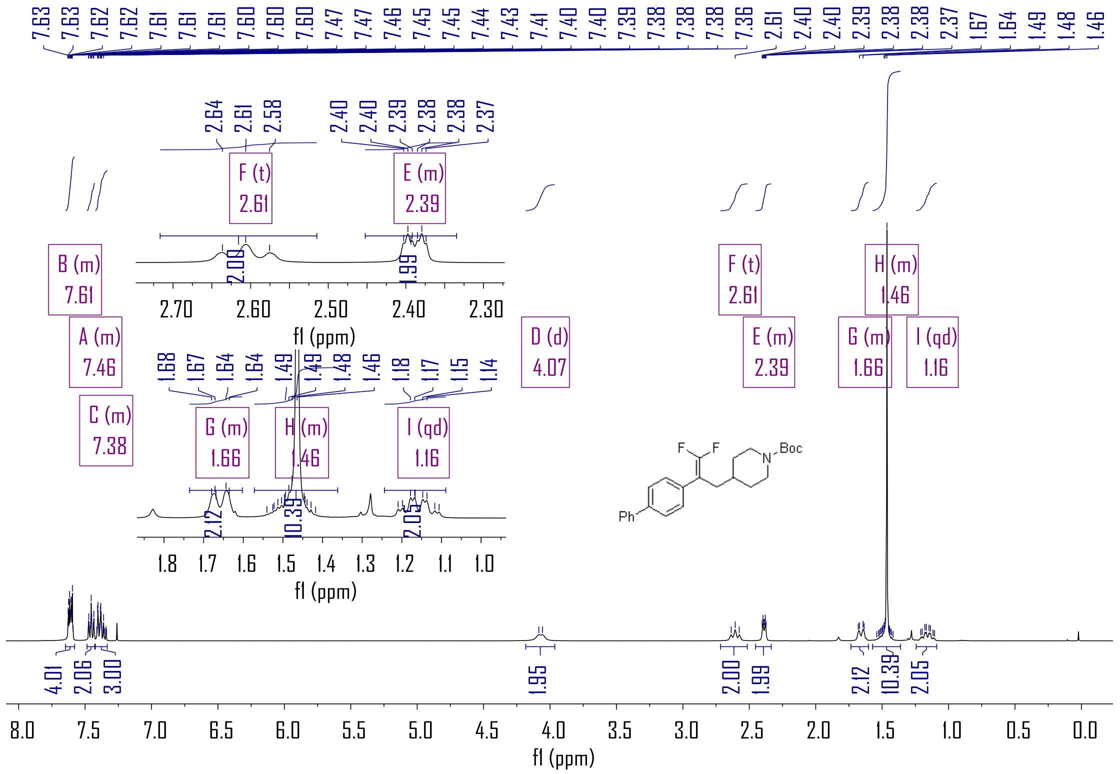

^{1}H NMR spectra for **3ag**.

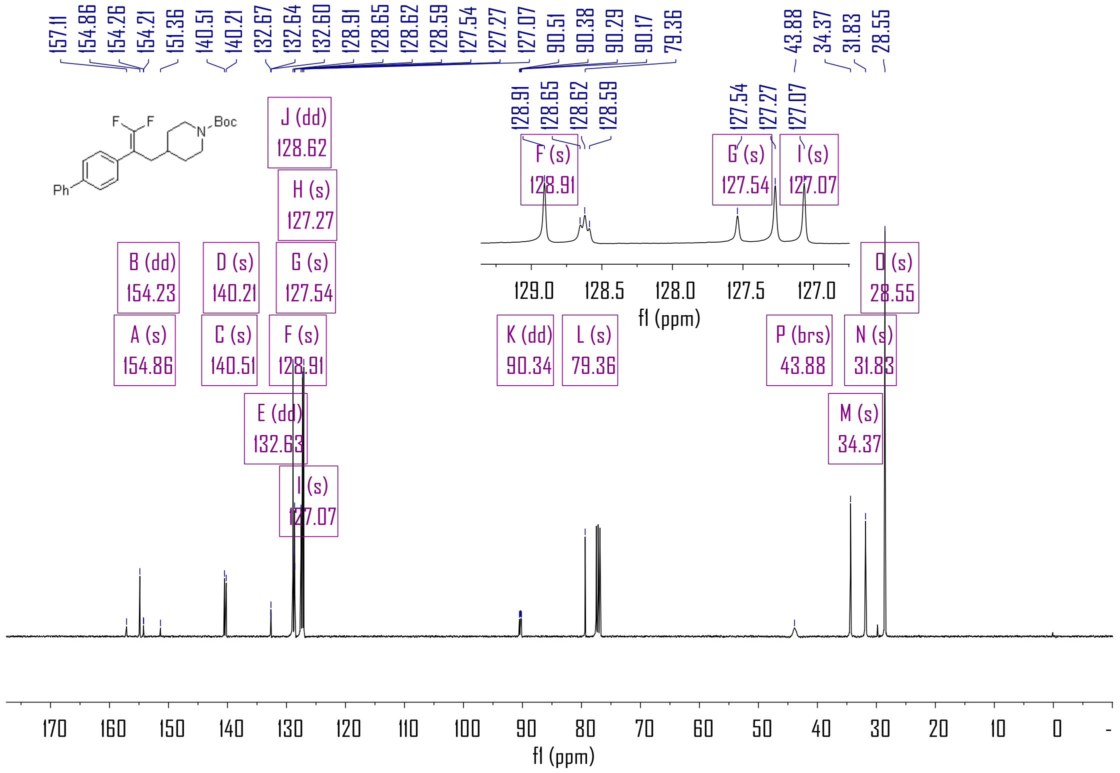

^{13}C NMR spectra for **3ag**.

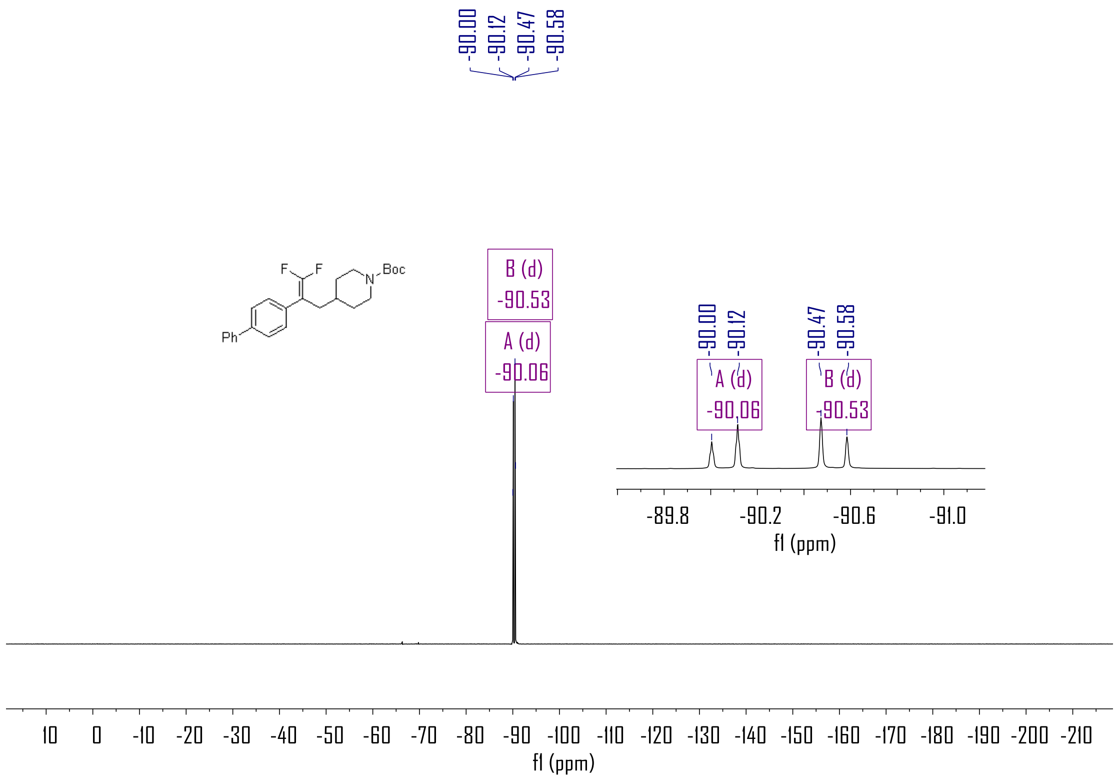

^{19}F NMR spectra for **3ag**.

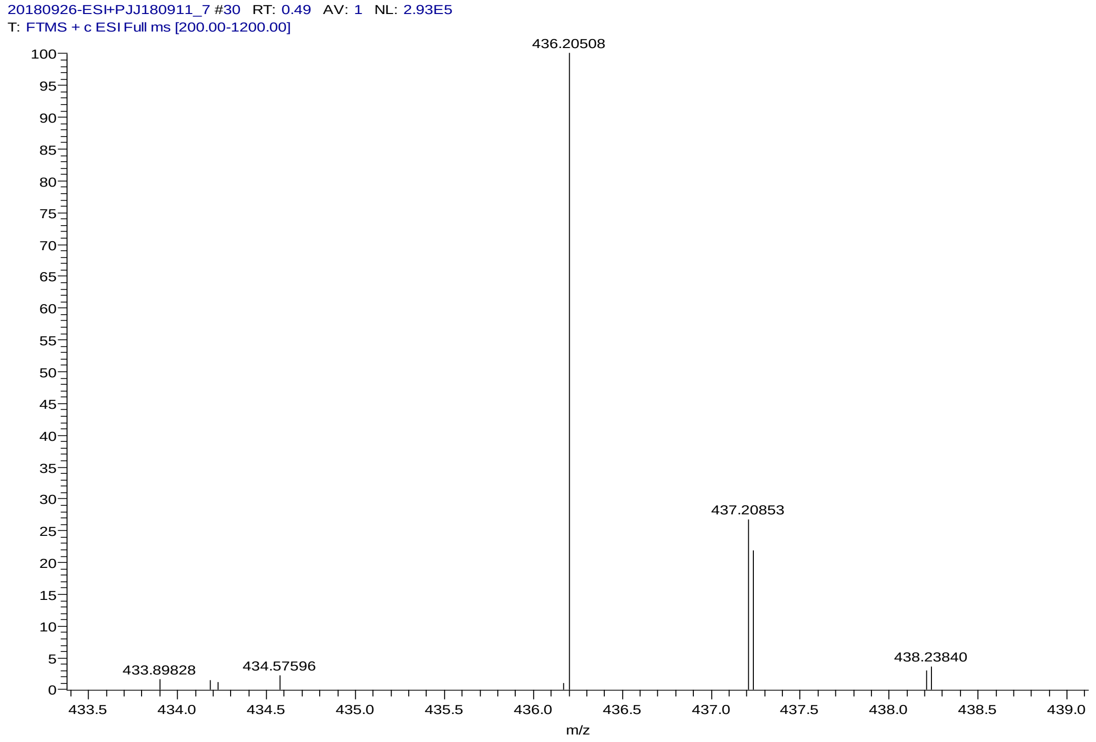

HRMS spectra for **3ag**.

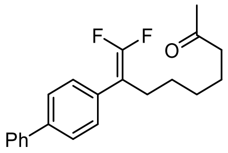

8-([1,1'-biphenyl]-4-yl)-9,9-difluoronon-8-en-2-one (**3ah**)

Following general procedure, **1a** and **2h** were used. The product was isolated by column chromatography as colorless oil (51.2 mg, 0.156 mmol, 78%).

Selectivity (desired C-F cleavage product : addition by-product) > 50:1.

R_f (petroleum ether : ethyl acetate = 20:1) = 0.32.

^{1}H NMR (400 MHz, Chloroform-*d*) δ 7.66 – 7.56 (m, 4H), 7.50 – 7.41 (m, 2H), 7.43 – 7.31 (m, 3H), 2.49 – 2.40 (m, 2H), 2.40 (t, *J* = 7.4 Hz, 2H), 2.11 (s, 3H), 1.63 – 1.52 (m, 2H), 1.48 – 1.38 (m, 2H), 1.37 – 1.27 (m, 2H).

^{13}C NMR (101 MHz, Chloroform-*d*) δ 209.12, 153.77 (dd, *J* = 289.8, 287.6 Hz), 140.64, 140.11, 132.71, 128.91, 128.66 (dd, *J* = 3.3, 3.3 Hz), 127.49, 127.20, 127.10, 92.08 (dd, *J* = 19.8, 14.7 Hz), 43.66, 29.97, 28.60, 27.65 (dd, *J* = 2.3, 2.3 Hz), 27.43, 23.52.

^{19}F NMR (376 MHz, Chloroform-*d*) δ -91.15 (dt, *J* = 43.8, 2.4 Hz), -91.29 (d, *J* = 43.4 Hz).

HRMS (ESI) calcd for $C_{21}H_{22}OF_2Na^+$ [(M+Na)$^+$] 351.15309, found 351.15308.

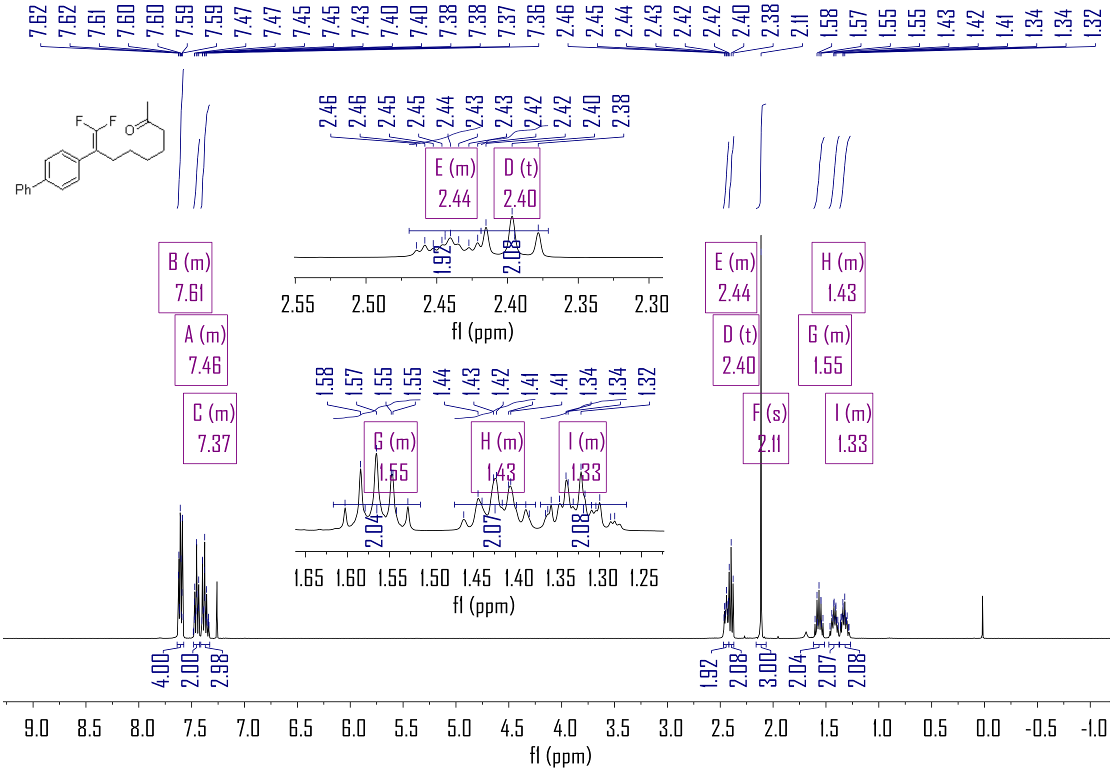

^{1}H NMR spectra for **3ah**.

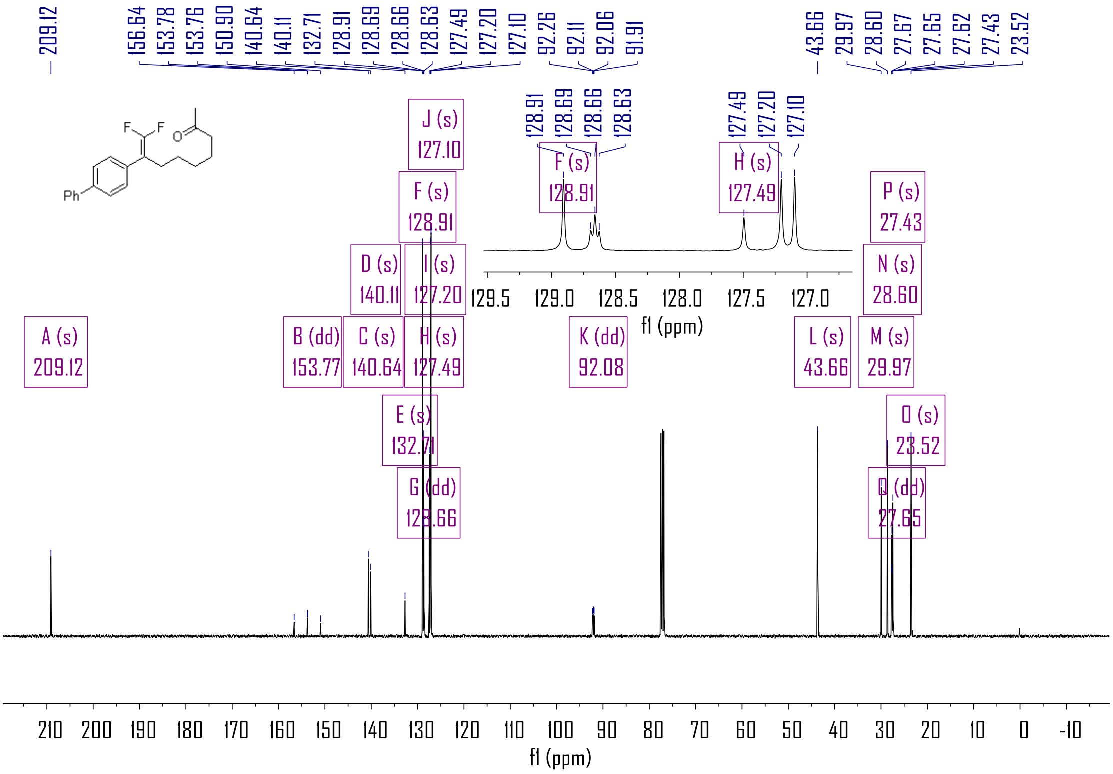

^{13}C NMR spectra for **3ah**.

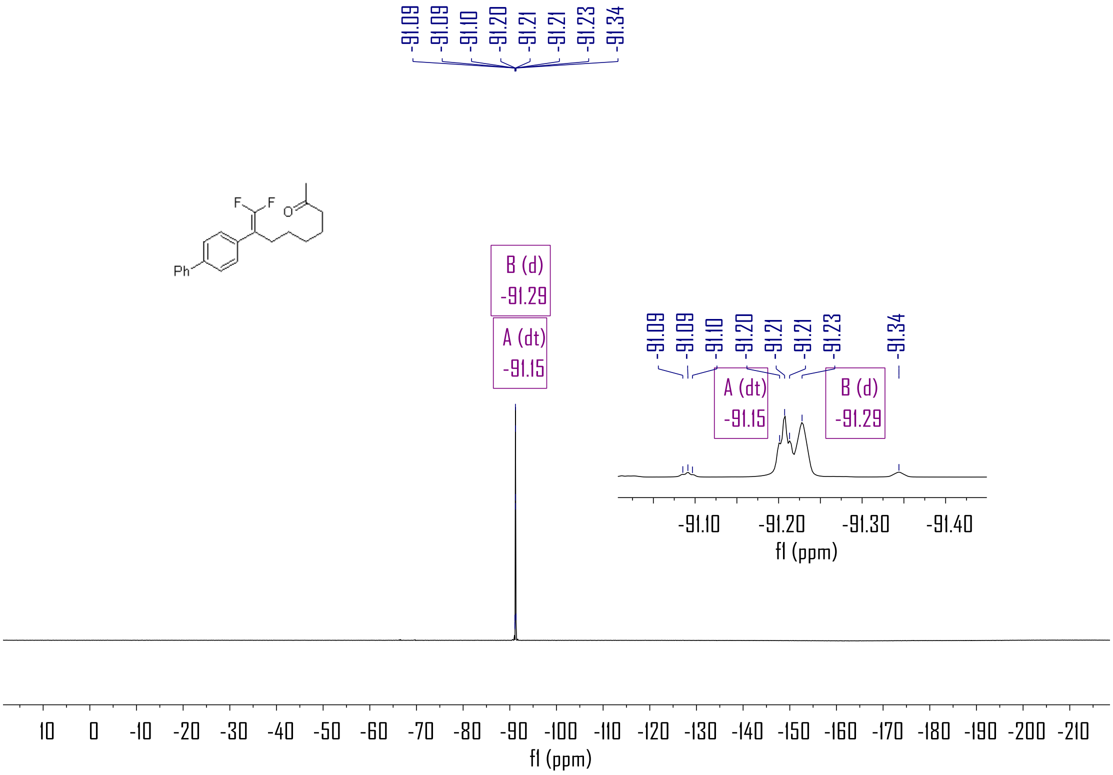

^{19}F NMR spectra for **3ah**.

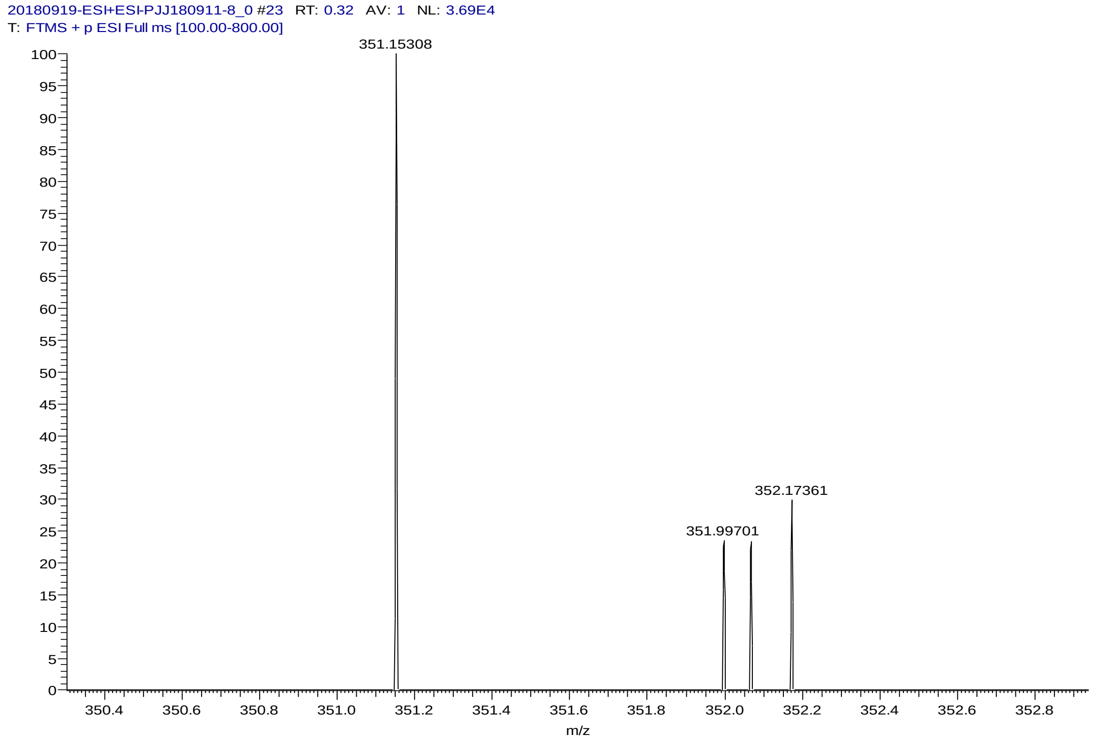

HRMS spectra for **3ah**.

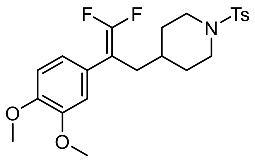

4-(2-(3,4-dimethoxyphenyl)-3,3-difluoroallyl)-1-tosylpiperidine (**3bi**)

Following general procedure, **1b** and **2i** were used. The product was isolated by column chromatography as colorless oil (56.9 mg, 0.126 mmol, 63%)

Selectivity (desired C-F cleavage product : addition by-product) > 50:1.

R_f (petroleum ether : ethyl acetate = 5:1) = 0.21.

^{1}H NMR (400 MHz, Chloroform-*d*) δ 7.58 (d, *J* = 8.1 Hz, 2H), 7.27 (d, *J* = 8.4 Hz, 2H), 6.83 – 6.72 (m, 3H), 3.84 (s, 3H), 3.83 (s, 3H), 3.71 (d, *J* = 11.6 Hz, 2H), 2.39 (s, 3H), 2.27 (d, *J* = 7.0 Hz, 2H), 2.14 – 2.02 (m, 2H), 1.66 (dd, *J* = 13.4, 3.3 Hz, 2H), 1.32 (qd, *J* = 12.3, 4.2 Hz, 2H), 1.17 (ddt, *J* = 11.3, 7.6, 3.8 Hz, 1H).

^{13}C NMR (101 MHz, Chloroform-*d*) δ 153.94 (dd, *J* = 288.1, 288.1 Hz), 148.86, 148.36, 143.47, 132.97, 129.60, 127.71, 125.78, 120.65 (dd, *J* = 3.2, 3.2 Hz), 111.49 (dd, *J* = 3.4, 3.4 Hz), 111.16, 90.07 (dd, *J* = 19.1, 16.1 Hz), 56.01, 55.88, 46.31, 34.08, 33.45, 31.09, 21.51.

^{19}F NMR (376 MHz, Chloroform-*d*) δ -91.28.

HRMS (ESI) calcd for $C_{23}H_{27}O_4NF_2NaS^+$ [(M+Na)$^+$] 474.15211, found 474.15176.

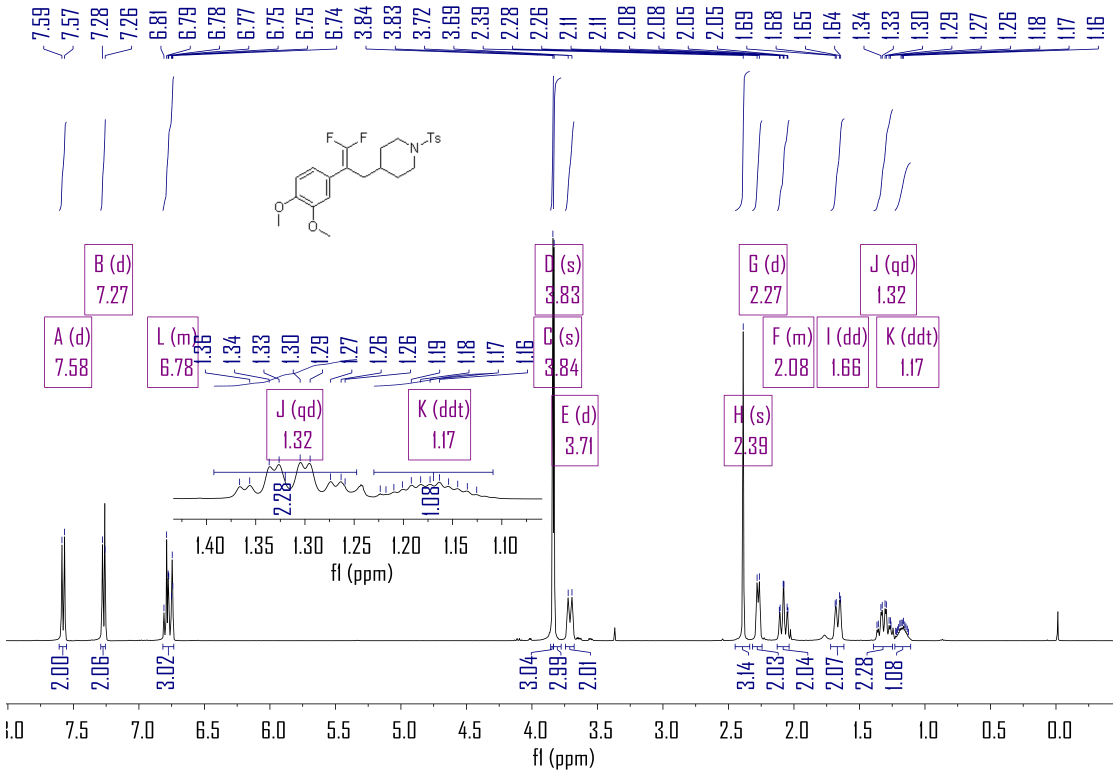

^{1}H NMR spectra for **3bi**.

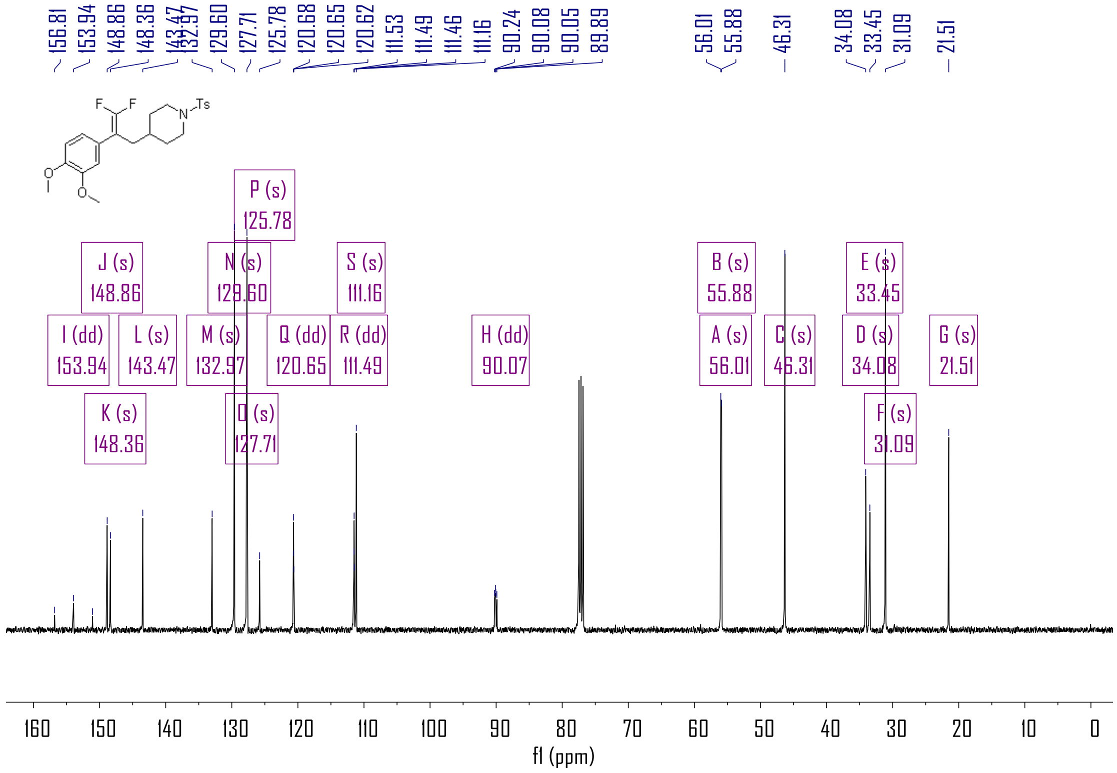

^{13}C NMR spectra for **3bi**.

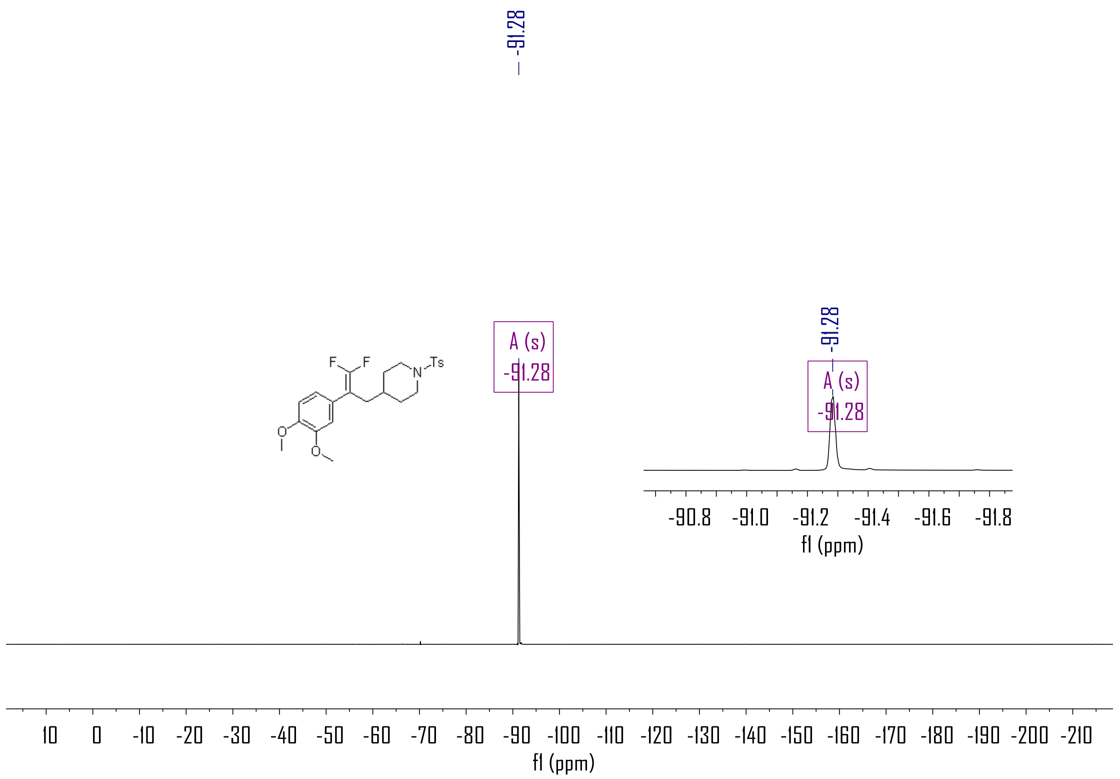

^{19}F NMR spectra for **3bi**.

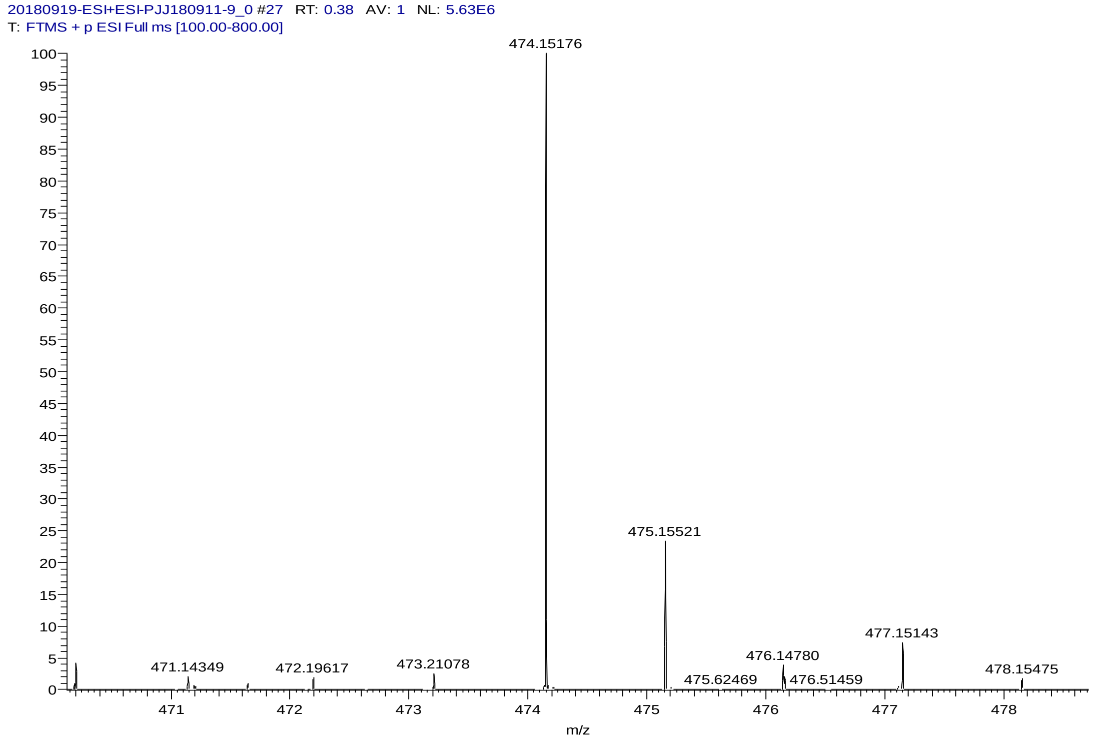

HRMS spectra for **3bi**.

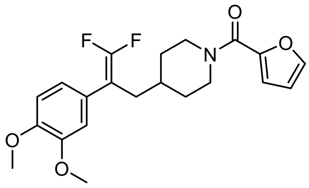

(4-(2-(3,4-dimethoxyphenyl)-3,3-difluoroallyl)piperidin-1-yl)(furan-2-yl)methanone (**3bj**)

Following general procedure, **1b** and **2j** were used. The product was isolated by column chromatography as colorless oil (61.0 mg, 0.156 mmol, 78%).

Selectivity (desired C-F cleavage product : addition by-product) > 50:1.

R_f (petroleum ether : ethyl acetate = 2:1) = 0.25.

^{1}H NMR (400 MHz, Chloroform-*d*) δ 7.44 (dd, *J* = 1.8, 0.8 Hz, 1H), 6.91 (dd, *J* = 3.4, 0.9 Hz, 1H), 6.85 (s, 2H), 6.81 (s, 1H), 6.44 (dd, *J* = 3.5, 1.8 Hz, 1H), 4.47 (brs, 2H), 3.87 (s, 3H), 3.87 (s, 3H), 2.37 – 2.29 (m, 2H), 1.73 (d, *J* = 12.6 Hz, 2H), 1.64 – 1.52 (m, 1H), 1.33 – 1.17 (m, 2H).

^{13}C NMR (101 MHz, Chloroform-*d*) δ 159.25, 154.04 (dd, *J* = 288.3, 288.3 Hz), 148.94, 148.45, 148.15, 143.54, 126.02, 120.74 (dd, *J* = 3.1, 3.1 Hz), 115.85, 111.60 (dd, *J* = 3.5, 3.5 Hz), 111.23, 111.20, 90.21 (dd, *J* = 17.7, 17.7 Hz), 56.06, 55.93, 46.48 (brs), 43.43 (brs), 34.60 (dd, *J* = 2.6, 2.6 Hz), 34.47, 32.20 (brs).

^{19}F NMR (376 MHz, Chloroform-*d*) δ -91.29.

HRMS (ESI) calcd for $C_{21}H_{23}O_4NF_2Na^+$ [$(M+Na)^+$] 414.14874, found 414.14880.

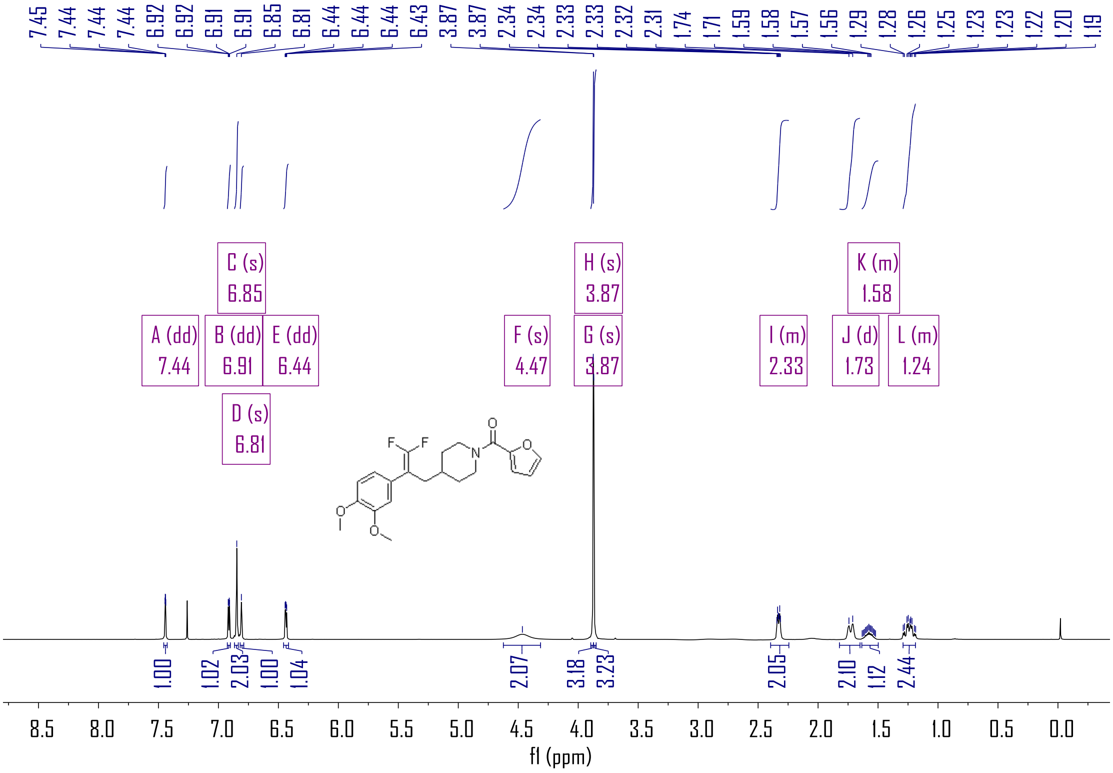

^{1}H NMR spectra for **3bj**.

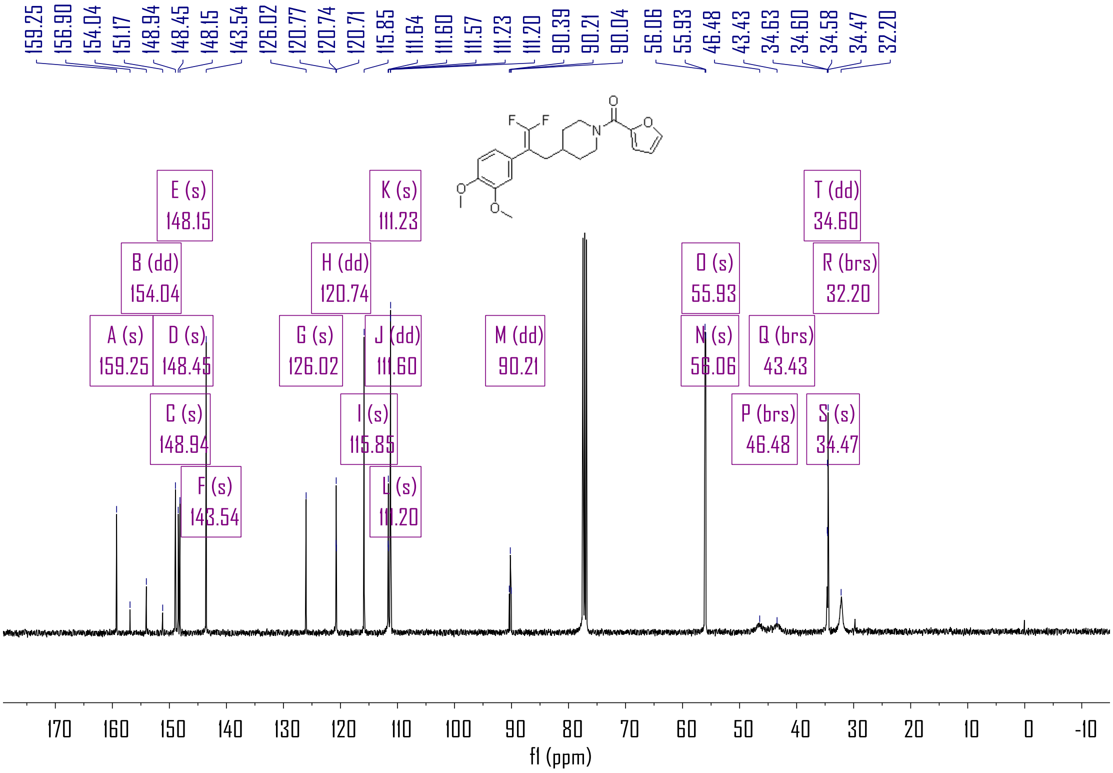

^{13}C NMR spectra for **3bj**.

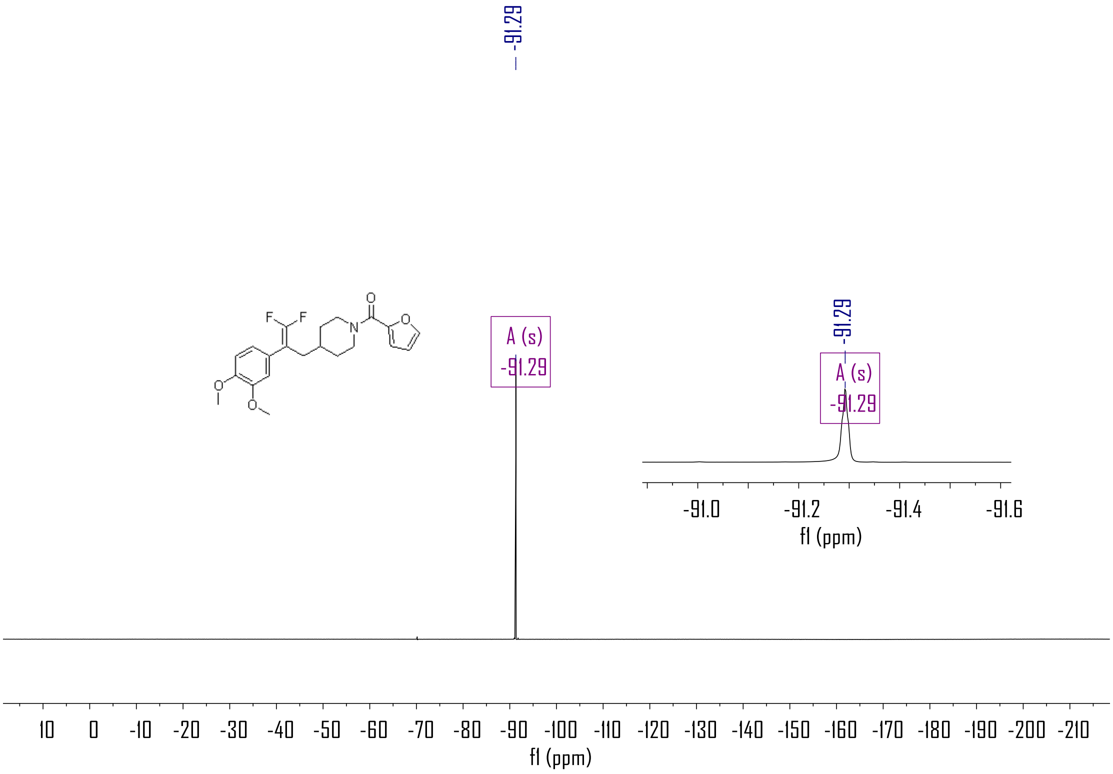

^{19}F NMR spectra for **3bj**.

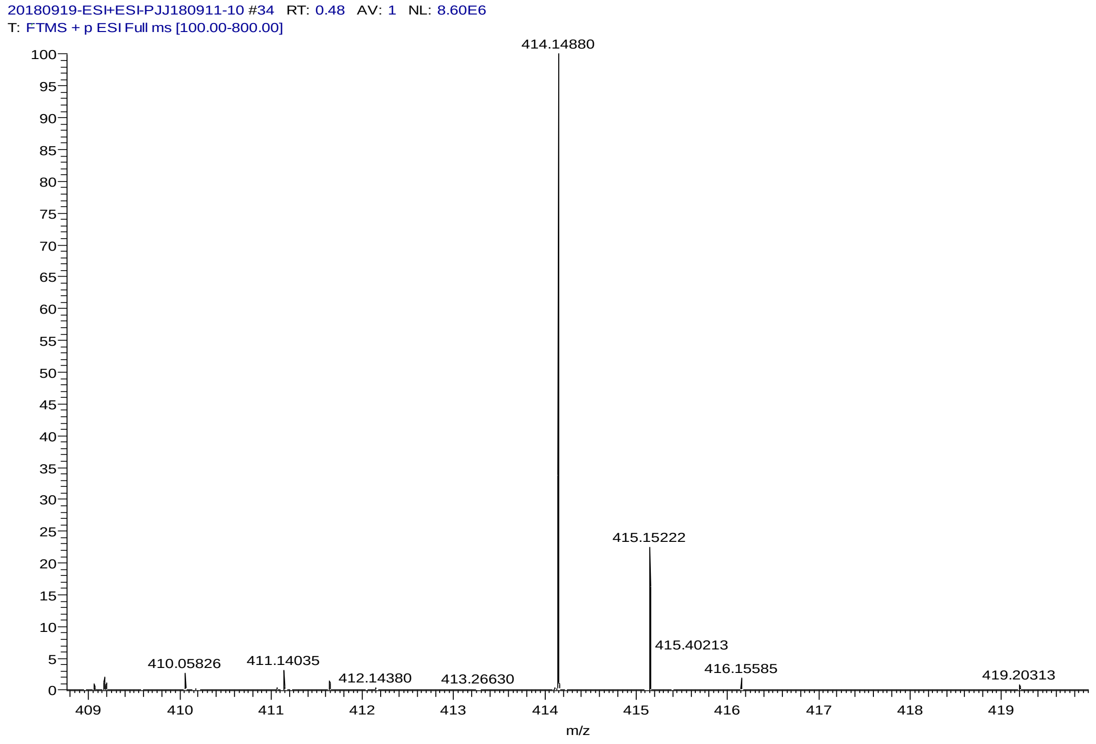

HRMS spectra for **3bj**.

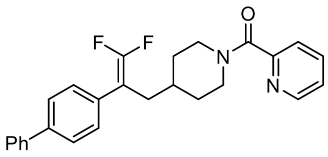

(4-(2-([1,1'-biphenyl]-4-yl)-3,3-difluoroallyl)piperidin-1-yl)(pyridin-2-yl)methanone (**3ak**)

Following general procedure, **1a** and **2k** were used. The product was isolated by column chromatography as colorless oil (75.3 mg, 0.180 mmol, 90%).

Selectivity (desired C-F cleavage product : addition by-product) > 50:1.

R_f (petroleum ether : ethyl acetate = 2:1) = 0.13.

^{1}H NMR (400 MHz, Chloroform-*d*) δ 8.57 (s, 1H), 7.76 (td, *J* = 7.7, 1.4 Hz, 1H), 7.62 – 7.55 (m, 5H), 7.47 – 7.29 (m, 6H), 4.73 – 4.66 (m, 1H), 3.88 – 3.81 (m, 1H), 2.94 (td, *J* = 13.5, 12.8, 2.5 Hz, 1H), 2.70 (td, *J* = 12.8, 2.9 Hz, 1H), 2.45 – 2.38 (m, 2H), 1.85 – 1.78 (m, 1H), 1.69 – 1.56 (m, 2H), 1.46 – 1.20 (m, 2H).

^{13}C NMR (101 MHz, Chloroform-*d*) δ 167.47, 154.40, 154.13 (dd, *J* = 291.4, 287.1 Hz), 148.33, 140.34, 140.16, 137.16, 132.36 (dd, *J* = 3.9, 3.9 Hz), 128.84, 128.52 (dd, *J* = 3.3, 3.3 Hz), 127.49, 127.22, 126.97, 124.33, 123.42, 90.15 (dd, *J* = 21.9, 13.0 Hz), 47.26, 42.44, 34.36 (dd, *J* = 2.6, 2.6 Hz), 34.09, 32.31, 31.49.

^{19}F NMR (376 MHz, Chloroform-*d*) δ -89.82 (dt, *J* = 41.3, 2.6 Hz), -90.35 (d, *J* = 41.5 Hz).

HRMS (ESI) calcd for $C_{26}H_{24}ON_2F_2Na^+$ [$(M+Na)^+$] 441.17489, found 441.17413.

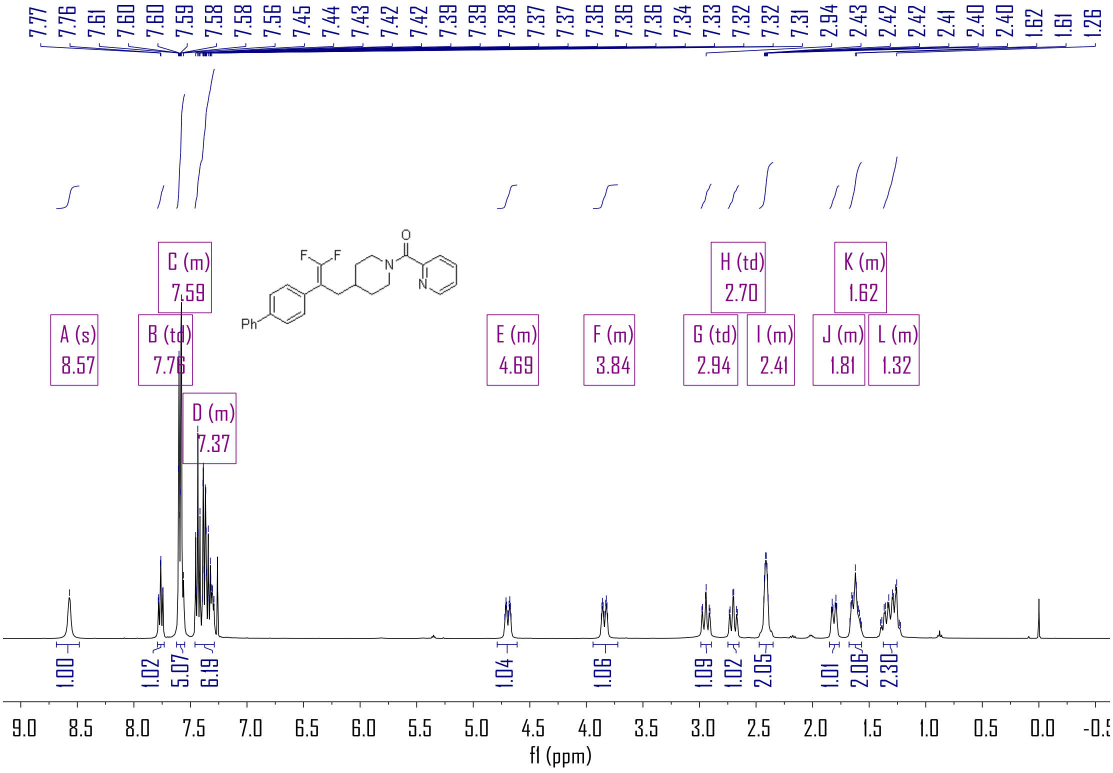

^{1}H NMR spectra for **3ak**.

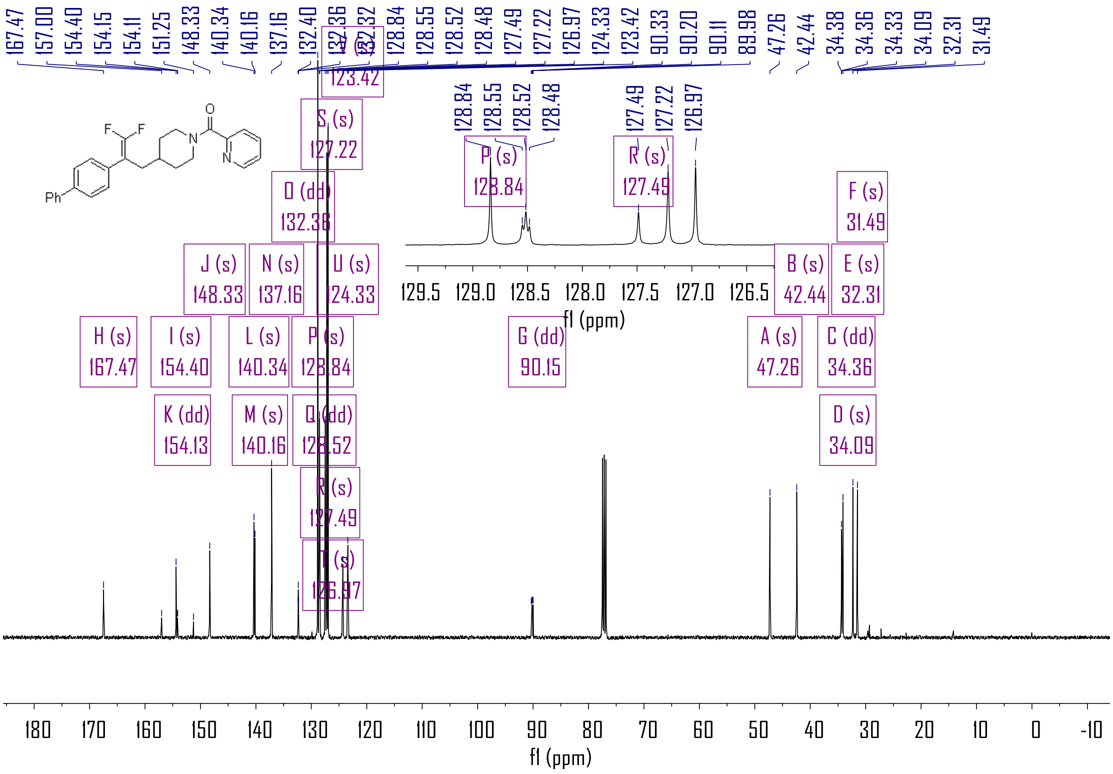

^{13}C NMR spectra for **3ak**.

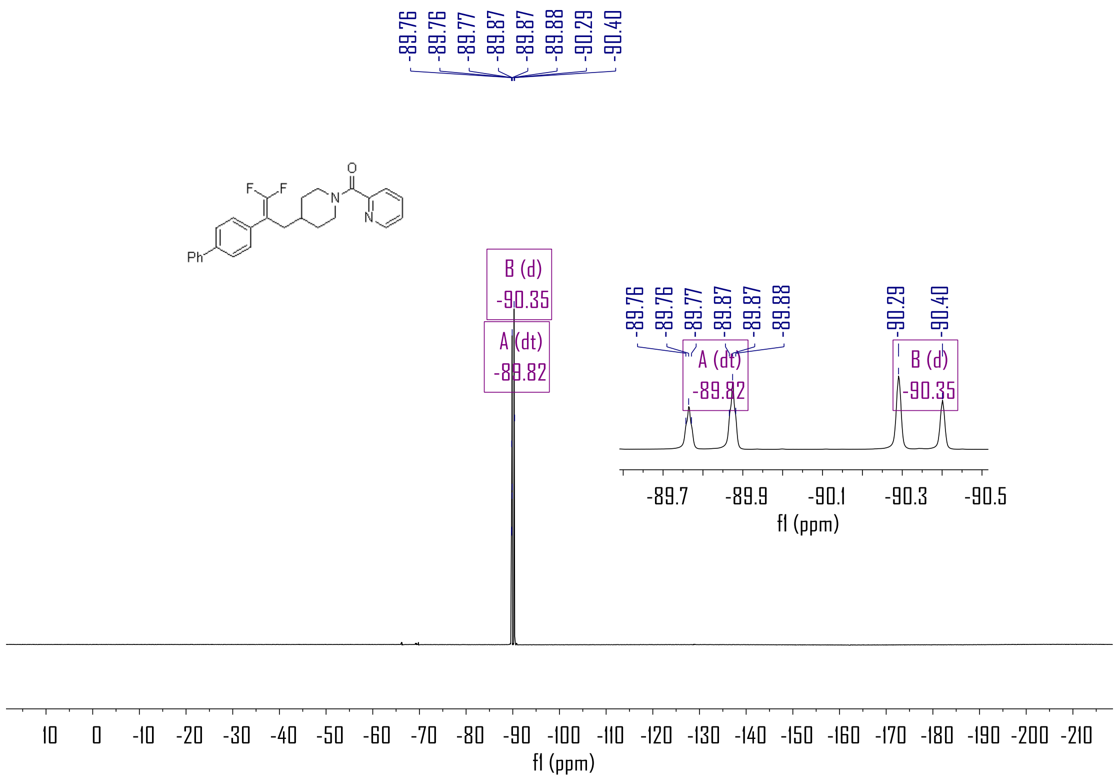

^{19}F NMR spectra for **3ak**.

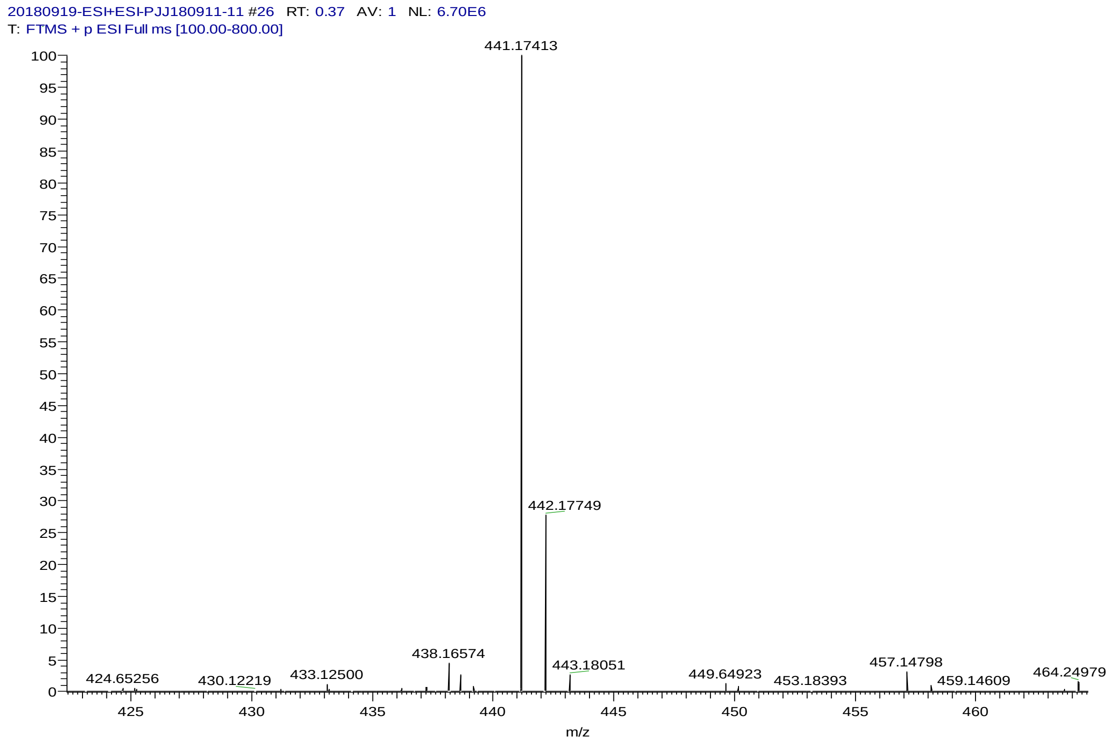

HRMS spectra for **3ak**.

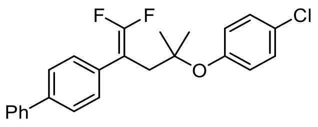

4-(4-chlorophenoxy)-1,1-difluoro-4-methylpent-1-en-2-yl)-1,1'-biphenyl (**3al**)

Following general procedure, **1a** and **2l** were used. The product was isolated by column chromatography as colorless oil (66.2 mg, 0.166 mmol, 83%).

Selectivity (desired C-F cleavage product : addition by-product) > 50:1.

R_f (petroleum ether) = 0.43.

^{1}H NMR (400 MHz, Chloroform-*d*) δ 7.65 – 7.59 (m, 4H), 7.50 – 7.42 (m, 4H), 7.40 – 7.35 (m, 1H), 7.19 (dd, *J* = 8.8, 0.6 Hz, 2H), 6.77 (dd, *J* = 9.0, 0.6 Hz, 2H), 2.92 – 2.85 (m, 2H), 1.23 (s, 6H).

^{13}C NMR (101 MHz, Chloroform-*d*) δ 154.90 (dd, *J* = 291.1, 289.0 Hz), 153.65, 140.56, 140.09, 133.67 (dd, *J* = 4.4, 3.2 Hz), 129.02 (dd, *J* = 2.4, 2.4 Hz), 128.93, 128.53, 127.54, 127.12, 127.09, 124.98, 89.74 (dd, *J* = 21.0, 14.8 Hz), 81.46 (dd, *J* = 3.2, 3.2 Hz), 40.50, 26.56.

^{19}F NMR (376 MHz, Chloroform-*d*) δ -88.24 (dt, *J* = 37.0, 3.0 Hz), -90.14 (d, *J* = 37.0 Hz).

HRMS (ESI) calcd for $C_{24}H_{22}OF_2Cl^+$ $[(M+H)^+]$ 399.1322, found 399.1327.

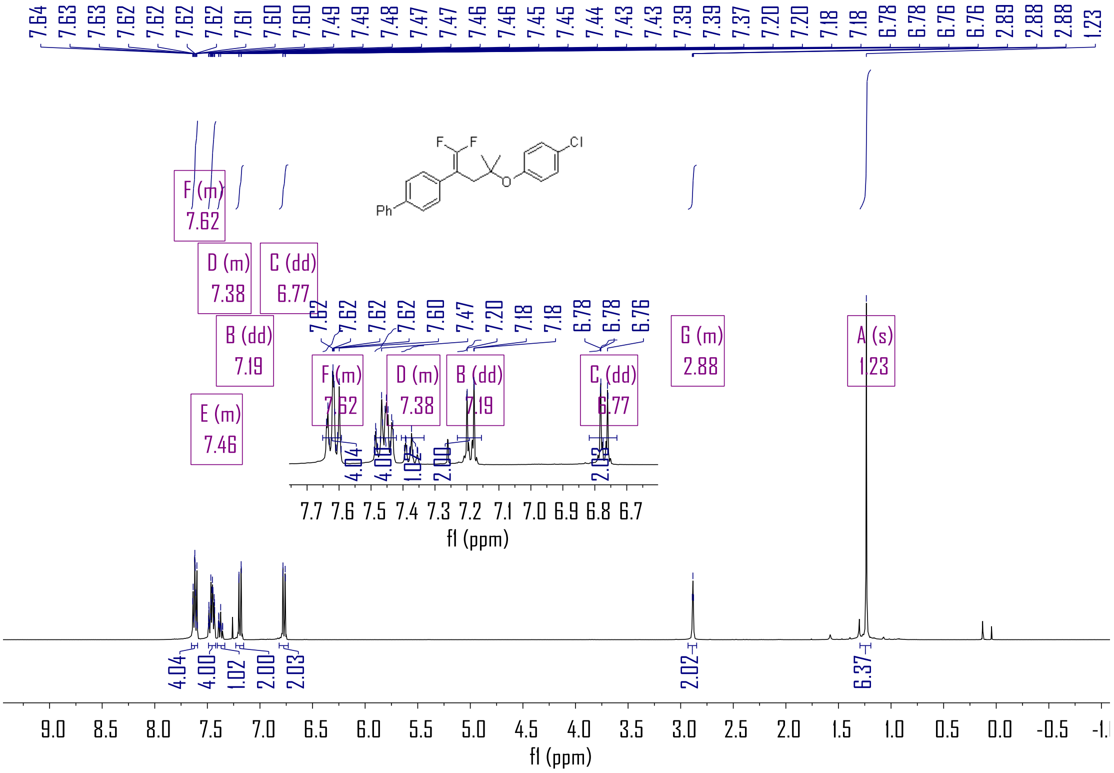

^{1}H NMR spectra for **3al**.

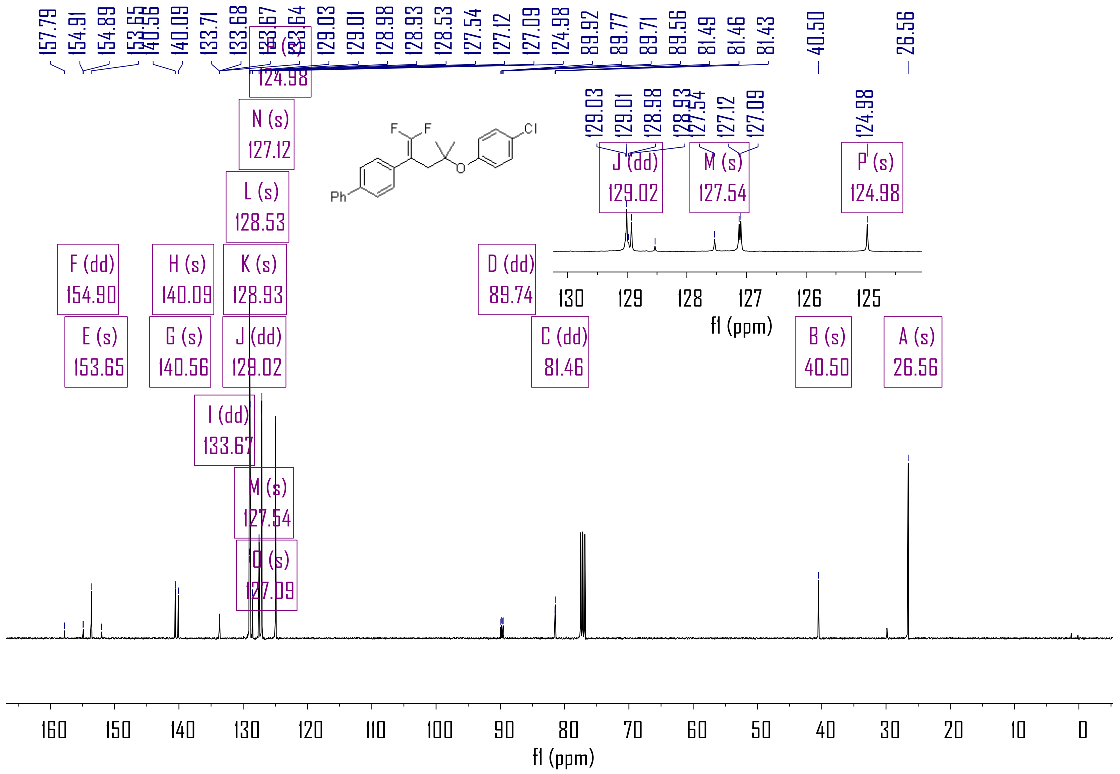

^{13}C NMR spectra for **3al**.

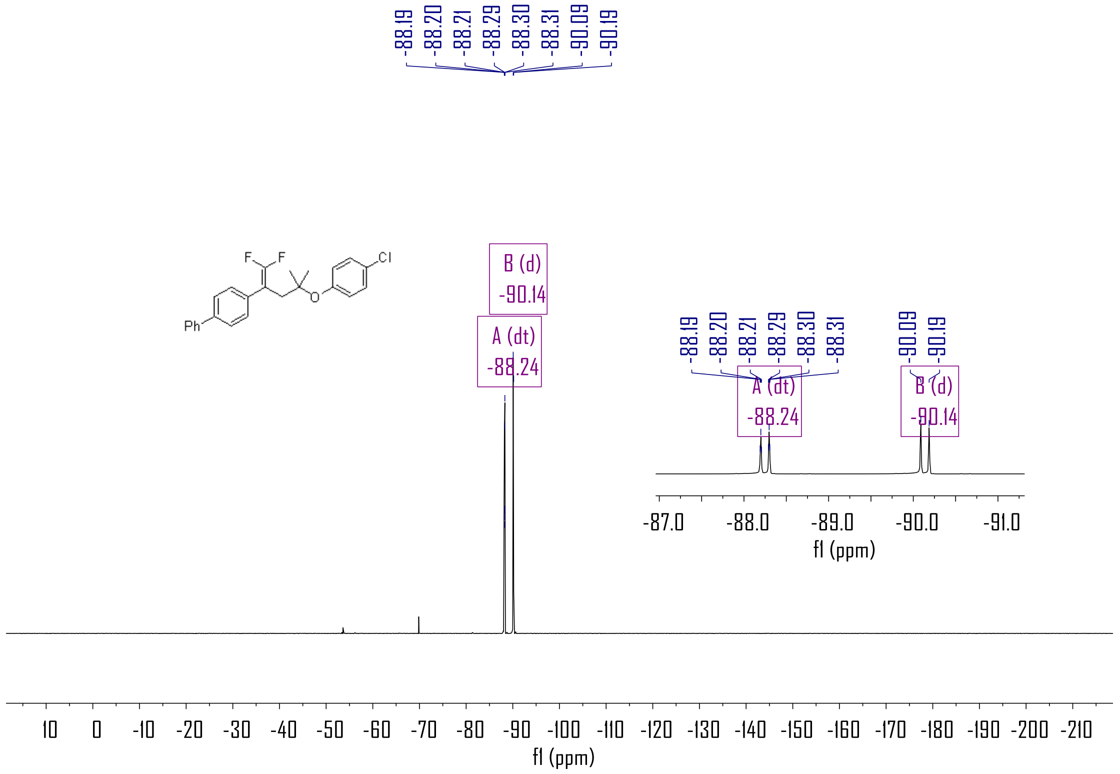

^{19}F NMR spectra for **3al**.

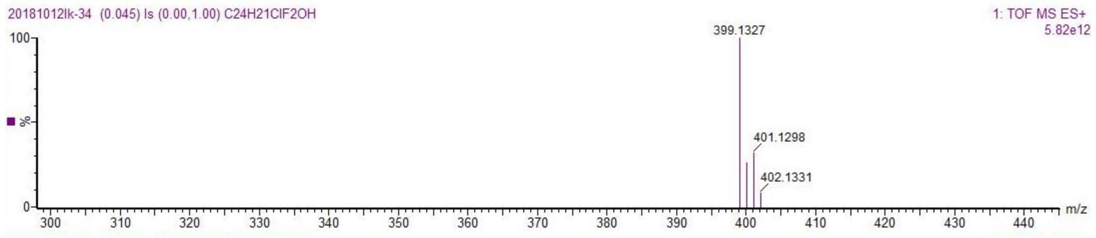

HRMS spectra for **3al**.

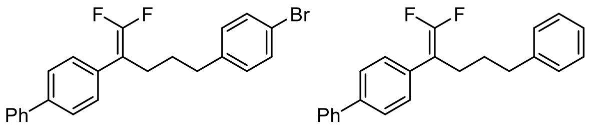

3am **3am'**

4-(5-(4-bromophenyl)-1,1-difluoropent-1-en-2-yl)-1,1'-biphenyl (**3am**)
4-(1,1-difluoro-5-phenylpent-1-en-2-yl)-1,1'-biphenyl (**3am'**)
Following general procedure, **1a** and **2m** were used. The product was isolated by column chromatography as colorless oil, 65.8 mg inseparable mixture was obtained, **3am** (0.140 mmol, 70%) and **3am'** (0.024 mmol, 12%).

Selectivity (desired C-F cleavage product : addition by-product) > 50:1.

R_f (petroleum ether) = 0.50.

^{1}H NMR (400 MHz, Chloroform-*d*) δ 7.70 – 7.57 (m, 4H), 7.52 – 7.43 (m, 2H), 7.45 – 7.34 (m, 5H), 7.08 – 6.98 (m, 2H), 2.65 – 2.58 (m, 2H), 2.53 – 2.45 (m, 2H), 1.80 – 1.68 (m, 2H).

^{13}C NMR (101 MHz, Chloroform-*d*) δ 153.79 (dd, *J* = 290.6, 287.5 Hz), 140.83, 140.59, 140.21, 132.48 (dd, *J* = 3.7, 2.4 Hz), 131.47, 130.25, 128.95, 128.66 (dd, *J* = 3.3, 3.3 Hz), 127.56, 127.27, 127.13, 119.69, 91.88 (dd, *J* = 20.8, 13.9 Hz), 34.70, 29.35 (dd, *J* = 2.6, 2.6 Hz), 27.12.

^{19}F NMR (376 MHz, Chloroform-*d*) δ -90.68 (dt, *J* = 42.4, 2.3 Hz), -90.83 (d, *J* = 42.7 Hz).

HRMS (EI) calcd for $C_{23}H_{19}BrF_2^+$ [M^+] 412.06327, found 412.06355.

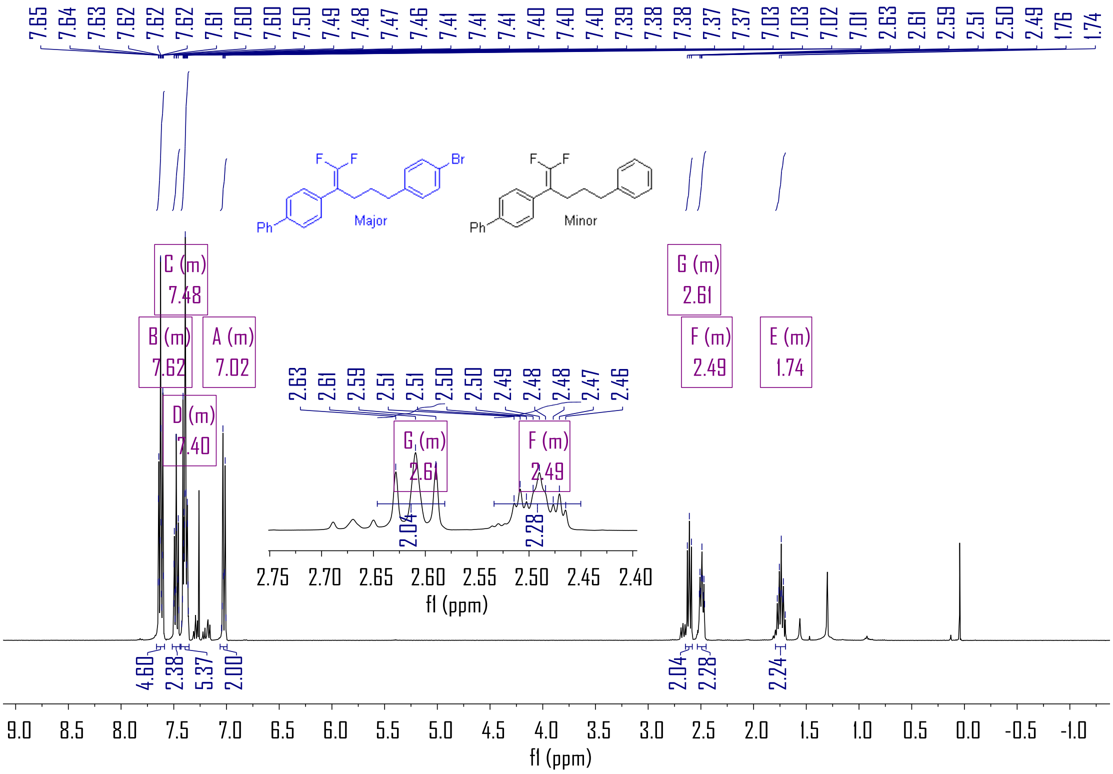

^{1}H NMR spectra for **3am** & **3am’**.

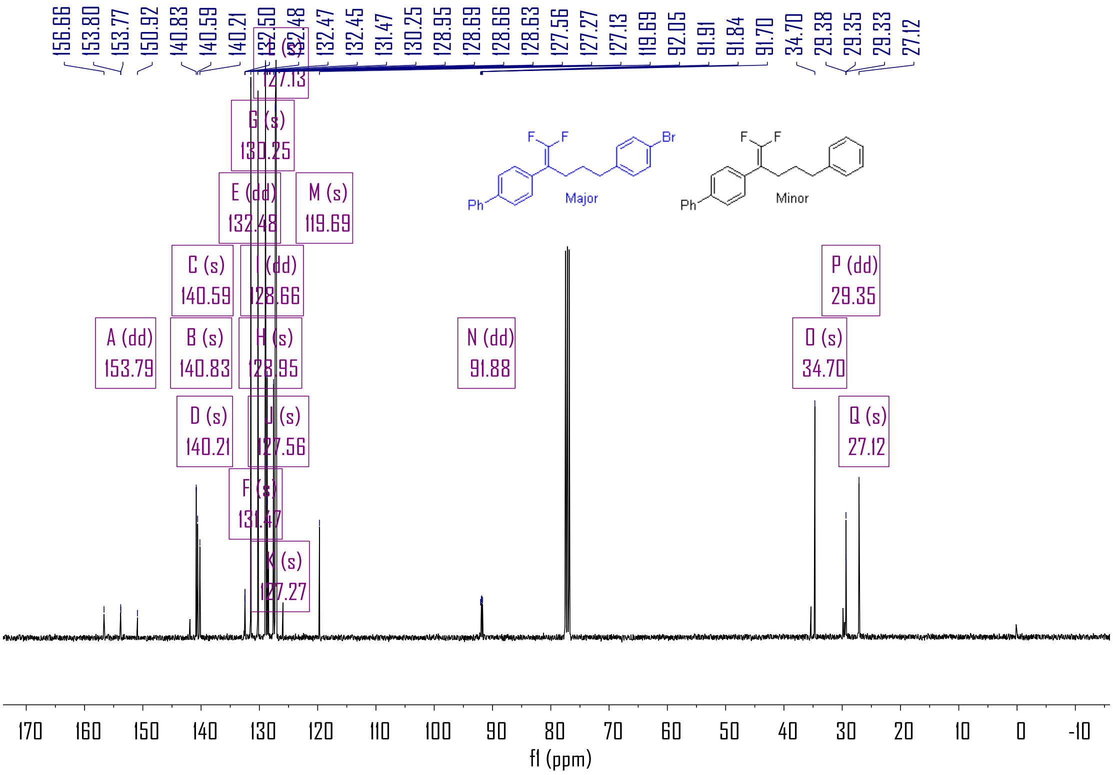

^{13}C NMR spectra for **3am** & **3am’**.

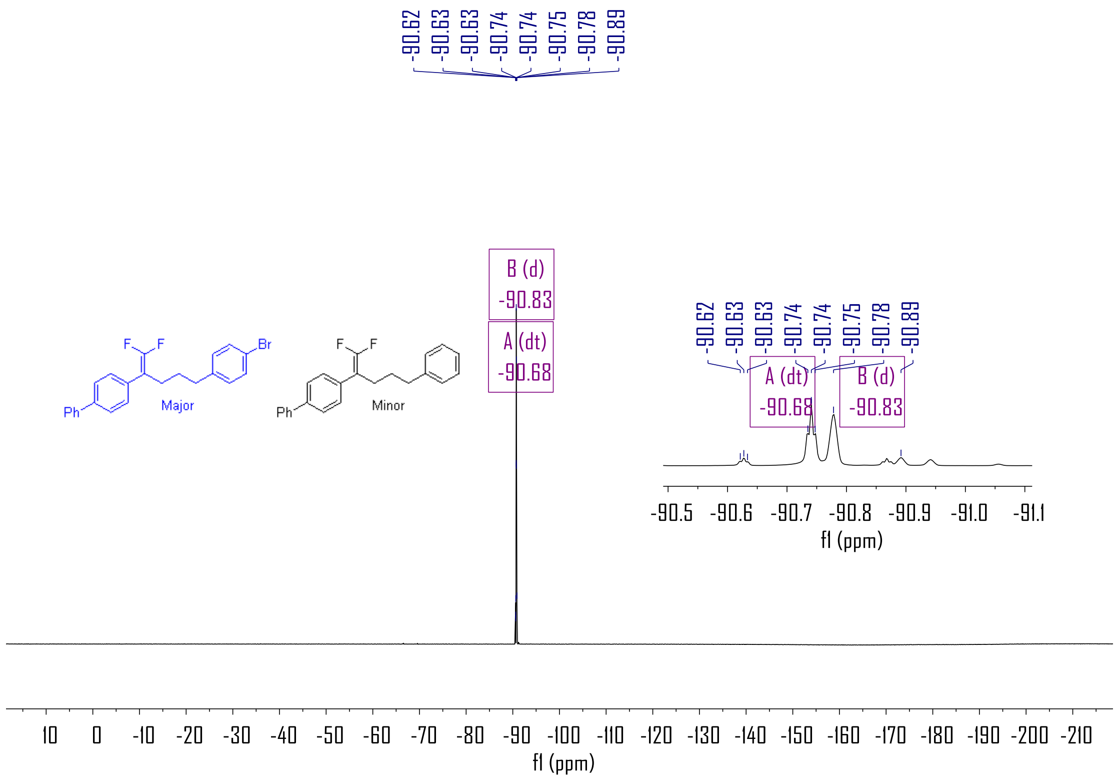

^{19}F NMR spectra for **3am** & **3am'**.

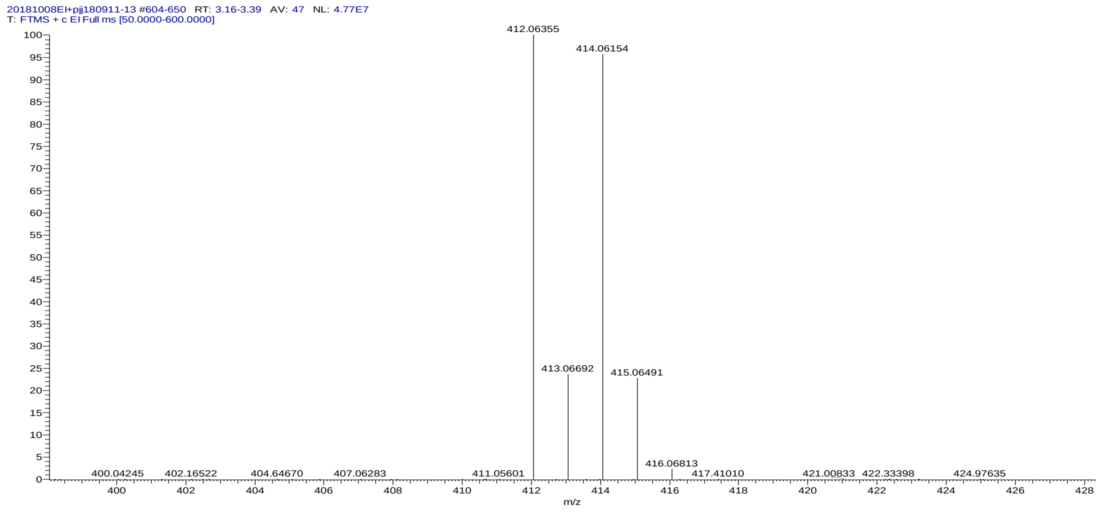

HRMS spectra for **3am**.

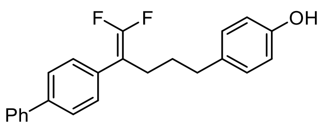

4-(4-([1,1'-biphenyl]-4-yl)-5,5-difluoropent-4-en-1-yl)phenol (**3an**)

Following general procedure, **1a** and **2n** were used. The product was isolated by column chromatography as white solid (49.7 mg, 0.142 mmol, 71%)

__**Selectivity (desired C-F cleavage product : addition by-product)**__ > 50:1.

R_f (petroleum ether : ethyl acetate = 5:1) = 0.48.

^{1}H NMR (400 MHz, Chloroform-*d*) δ 7.64 – 7.57 (m, 4H), 7.50 – 7.43 (m, 2H), 7.41 – 7.33 (m, 3H), 7.01 (d, *J* = 7.9 Hz, 2H), 6.84 – 6.68 (m, 2H), 2.58 (t, *J* = 7.7 Hz, 2H), 2.47 (t, *J* = 7.5 Hz, 2H), 1.71 (p, *J* = 7.5 Hz, 2H).

^{13}C NMR (101 MHz, Chloroform-*d*) δ 153.76 (dd, *J* = 290.5, 287.2 Hz), 153.68, 140.67, 140.11, 134.15, 132.66 (dd, *J* = 4.0, 2.8 Hz), 129.57, 128.94, 128.69 (dd, *J* = 3.3, 3.3 Hz), 127.52, 127.23, 127.14, 115.25, 92.04 (dd, *J* = 21.3, 13.3 Hz), 34.44, 29.78 (dd, *J* = 2.5, 2.5 Hz), 27.17.

^{19}F NMR (376 MHz, Chloroform-*d*) δ -90.87 (d, *J* = 43.1 Hz), -91.06 (d, *J* = 43.1 Hz).

HRMS (APCI) calcd for $C_{23}H_{21}OF_2^+$ [(M+H)$^+$] 351.15550, found 351.15549.

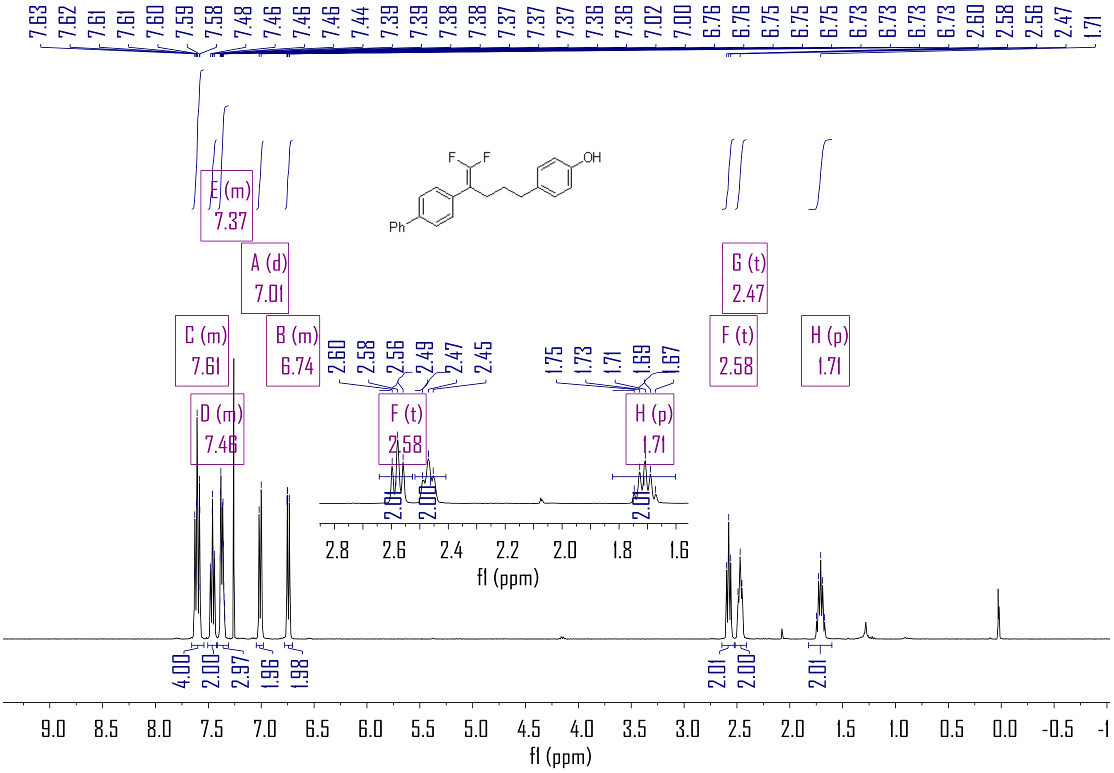

^{1}H NMR spectra for **3an**.

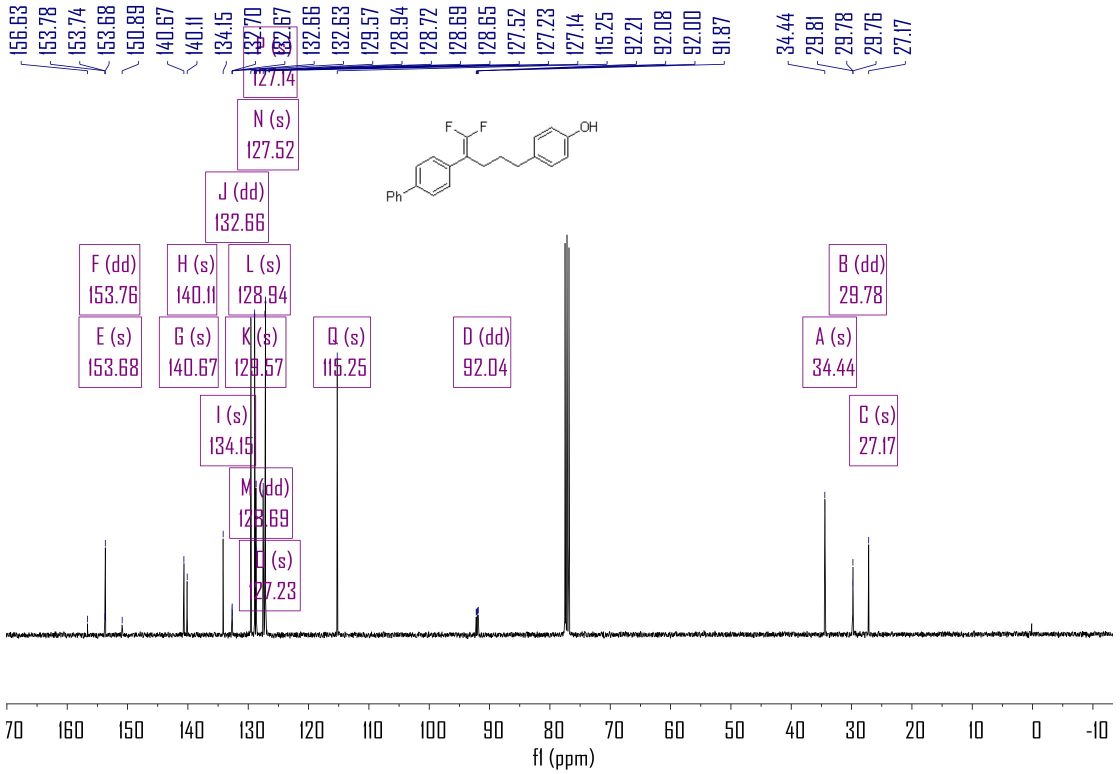

^{13}C NMR spectra for **3an**.

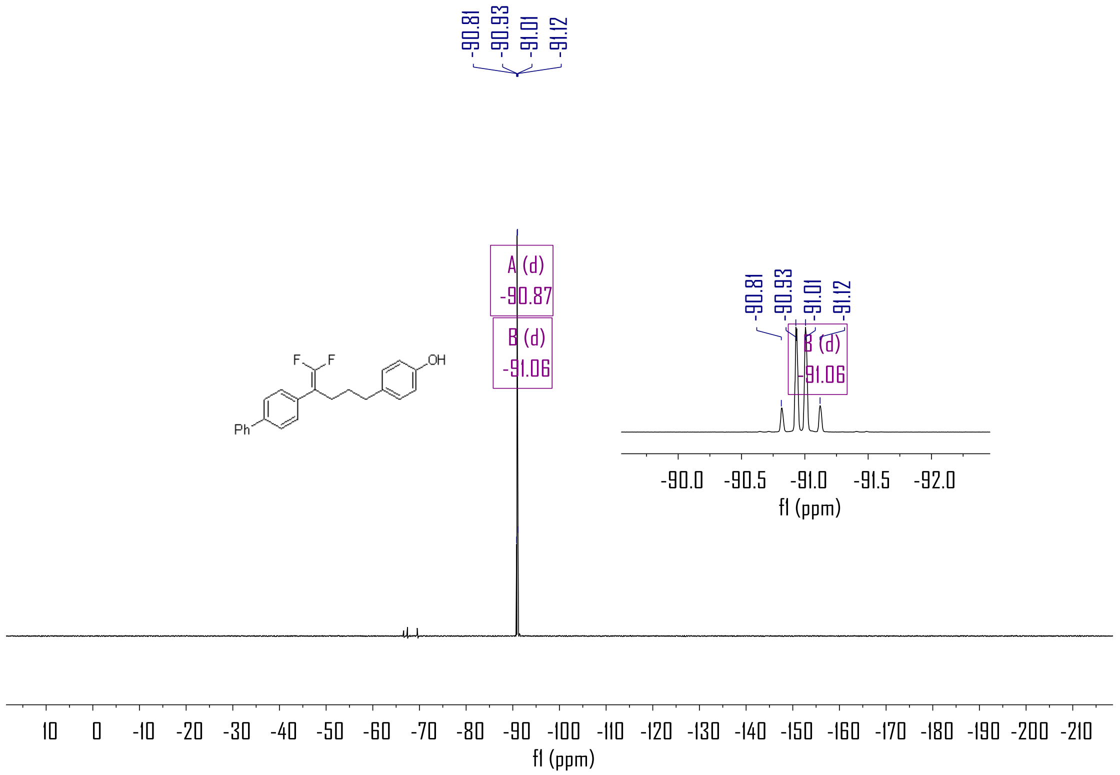

^{19}F NMR spectra for **3an**.

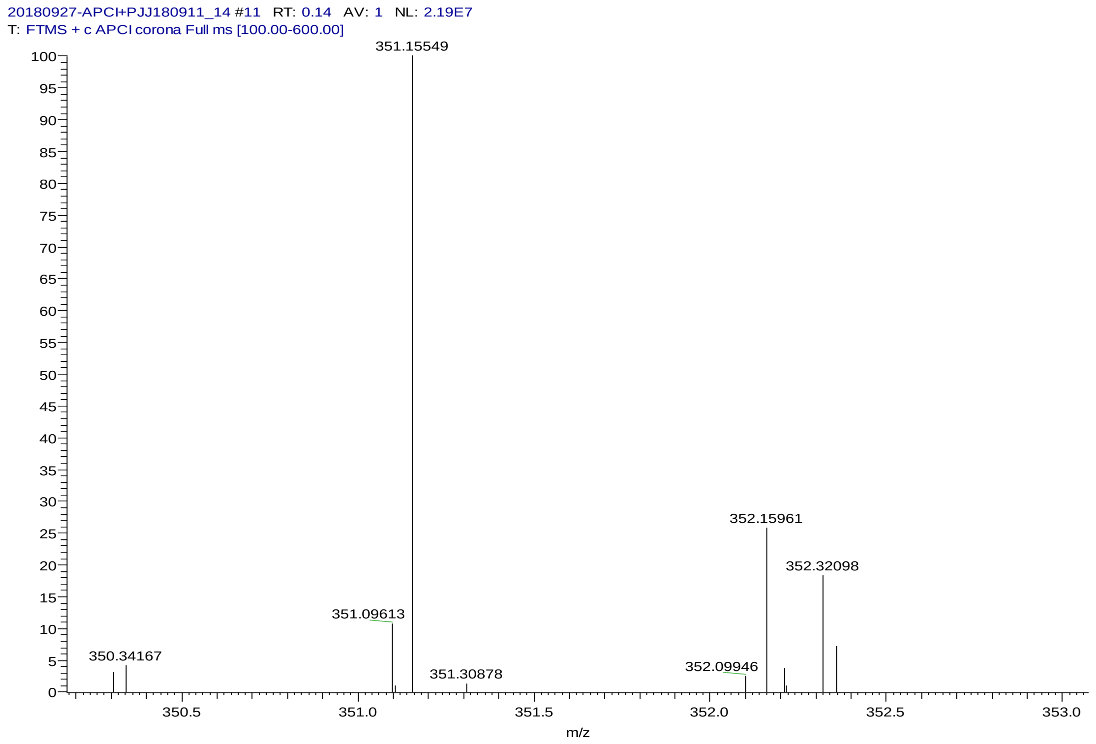

HRMS spectra for **3an**.

6. One-Pot Synthesis at Gram Scale

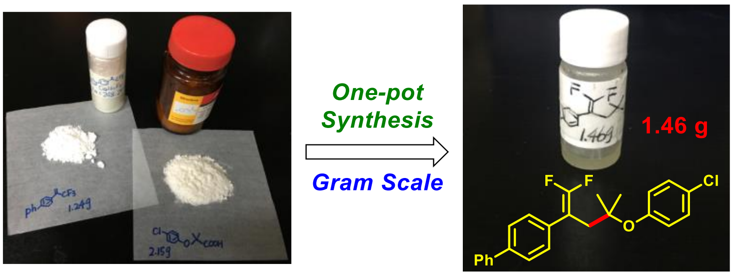

3al, 0.2 mmol scale, 79%
5.0 mmol scale, 73%

One-pot synthesis at gram scale.

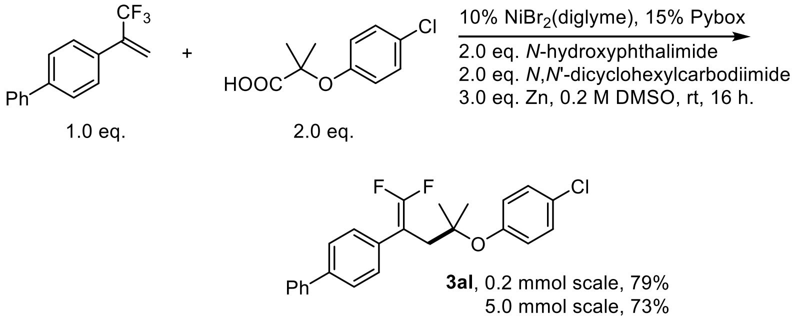

Preparation of **3al** in one pot.

$NiBr_2$(diglyme) (10 mol%), Pybox (15 mol%), *N*-hydroxyphthalimide (2.0 equiv.), *N*,*N*'-dicyclohexylcarbodiimide (2.0 equiv.), and Zn (3.0 equiv.) were added to a Schlenk tube equipped with a stir bar. The Schlenk tube was evacuated and filled with argon (three cycles). To these solids, DMSO (0.2 M) was added under argon atmosphere. Then, trifluoromethyl alkene (1.0 equiv.) and carboxylic acid (2.0 equiv.) were added and stirred at room temperature (~20 °C) for 16 hours. The mixture was purified by column chromatography to afford the desired product. For 0.2 mmol scale reaction, **3al** was obtained as colorless oil (62.9 mg, 0.158 mmol, 79%). For 5.0 mmol scale reaction, **3al** was obtained as colorless oil (1.46 g, 3.66 mmol, 73%).

7. Examples Described in Table 3

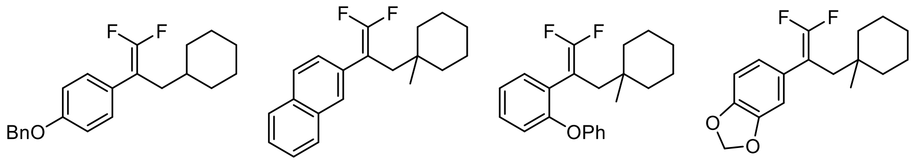

3ca **3db** **3eb** **3fb**

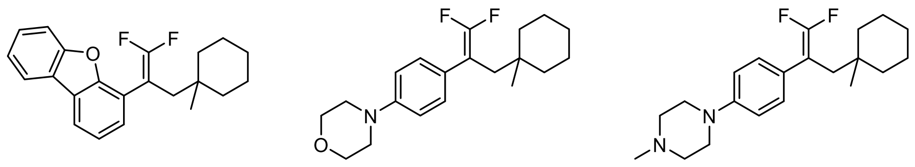

3gb **3hb** **3ib**

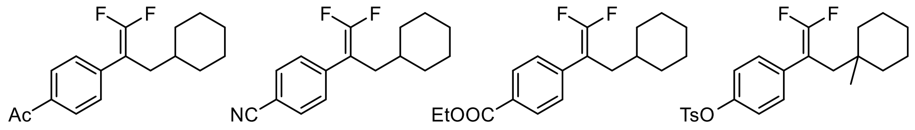

3ja **3ka** **3la** **3mb**

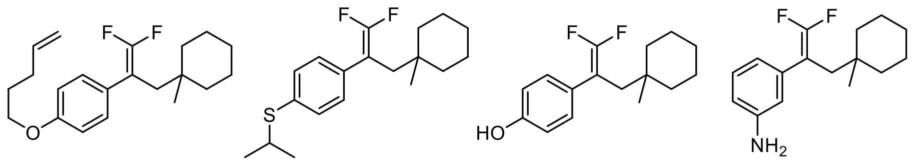

3nb **3ob** **3pb** **3qb**

List of substrates in Table 3.

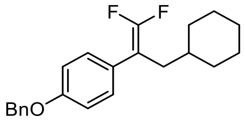

1-(benzyloxy)-4-(3-cyclohexyl-1,1-difluoroprop-1-en-2-yl)benzene (**3ca**)

Following general procedure, **1c** and **2a** were used. The product was isolated by column chromatography as white solid (47.9 mg, 0.140 mmol, 70%).

Selectivity (desired C-F cleavage product : addition by-product) > 50:1.

R_f (petroleum ether) = 0.60.

^{1}H NMR (400 MHz, Chloroform-*d*) δ 7.51 – 7.27 (m, 5H), 7.26 – 7.17 (m, 2H), 7.01 – 6.91 (m, 2H), 5.04 (s, 2H), 2.43 – 2.09 (m, 2H), 1.77 – 1.55 (m, 5H), 1.32 – 1.05 (m, 4H), 0.99 – 0.83 (m, 2H).

^{13}C NMR (101 MHz, Chloroform-*d*) δ 157.94, 154.01 (dd, *J* = 289.1, 285.4 Hz), 137.05, 129.51 (dd, *J* = 3.3, 3.3 Hz), 128.72, 128.13, 127.63, 126.64 (dd, *J* = 4.5, 3.4 Hz), 114.82, 90.60 (dd, *J* = 22.1, 12.8 Hz), 70.13, 35.76 (dd, *J* = 2.5, 2.5 Hz), 35.43, 32.99, 26.56, 26.21.

^{19}F NMR (376 MHz, Chloroform-*d*) δ -92.17 (d, *J* = 46.5 Hz), -92.58 (d, *J* = 46.5 Hz).
HRMS (APCI) calcd for $C_{22}H_{25}OF_2^+$ [(M+H)$^+$] 343.18680, found 343.18597.

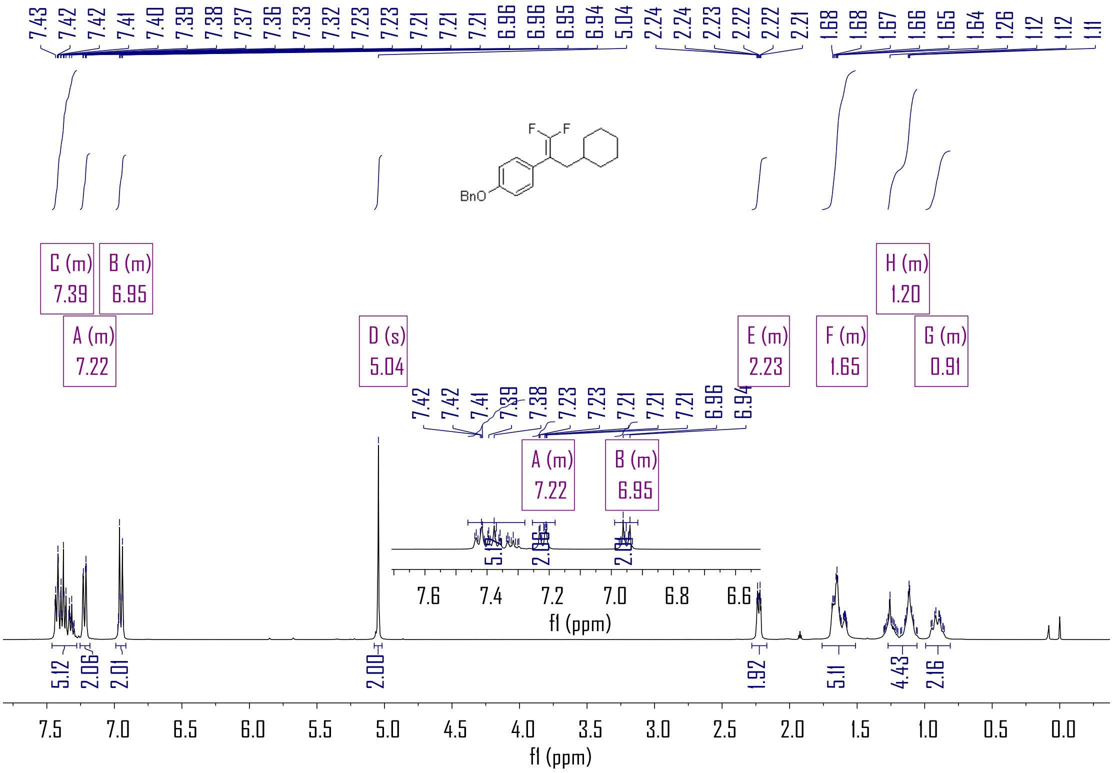

^{1}H NMR spectra for **3ca**.

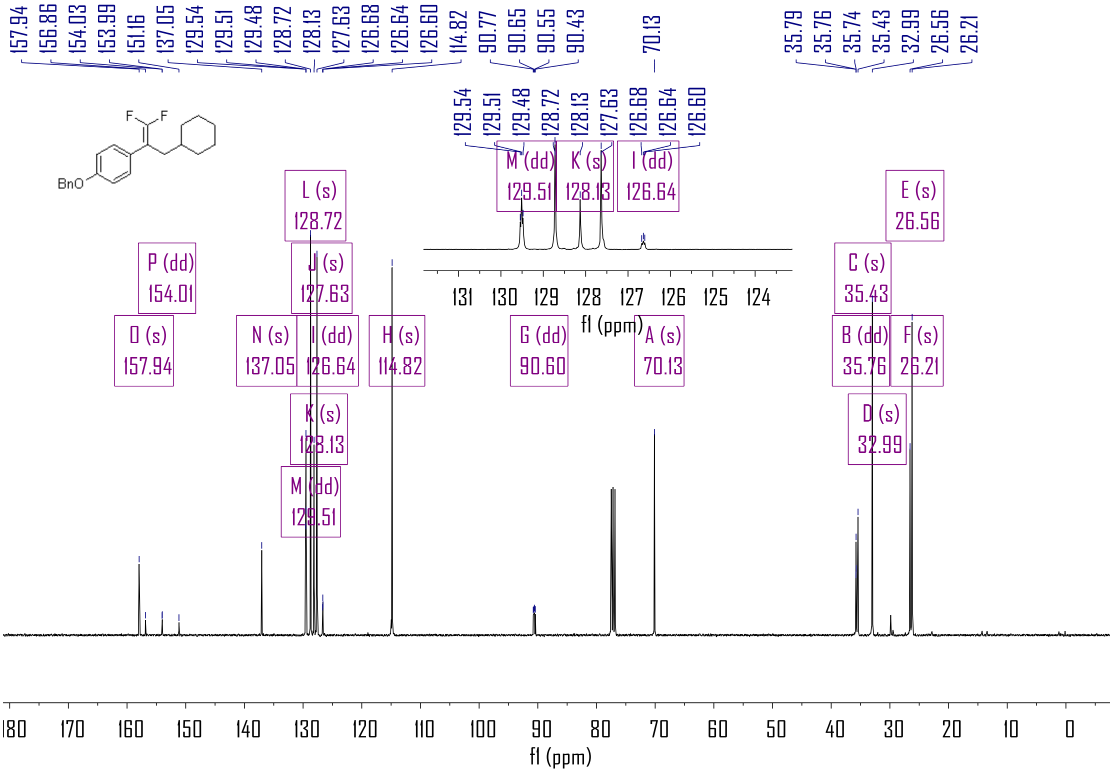

^{13}C NMR spectra for **3ca**.

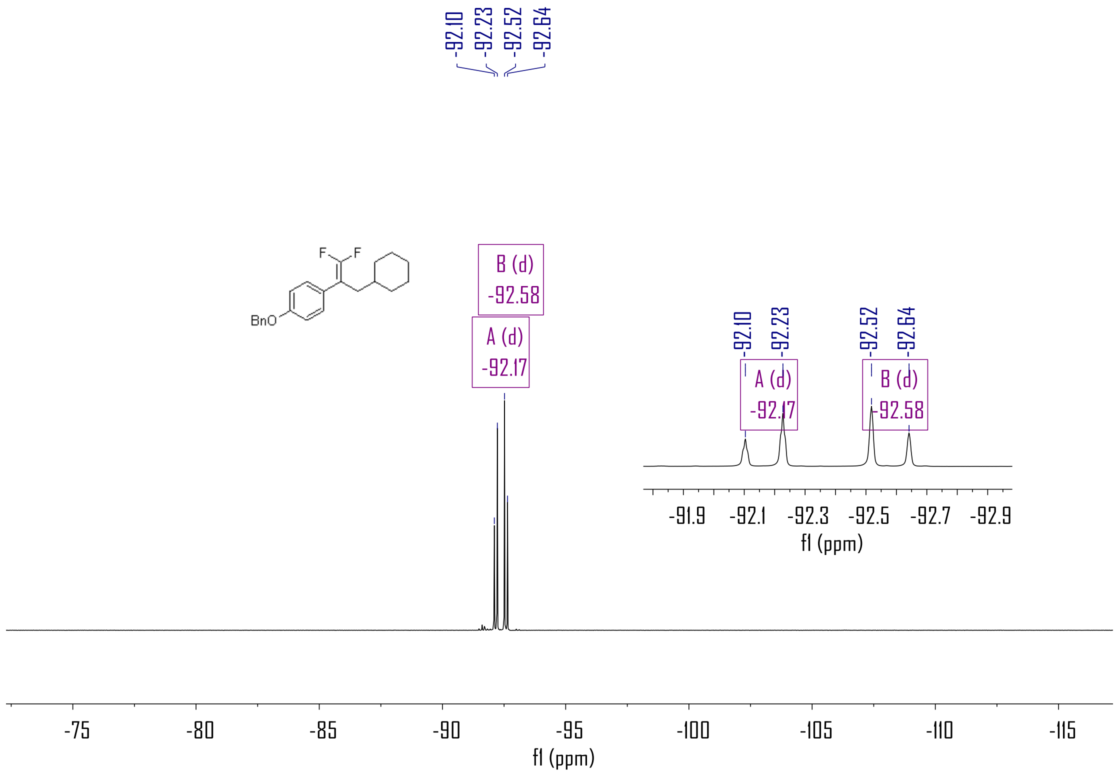

^{19}F NMR spectra for **3ca**.

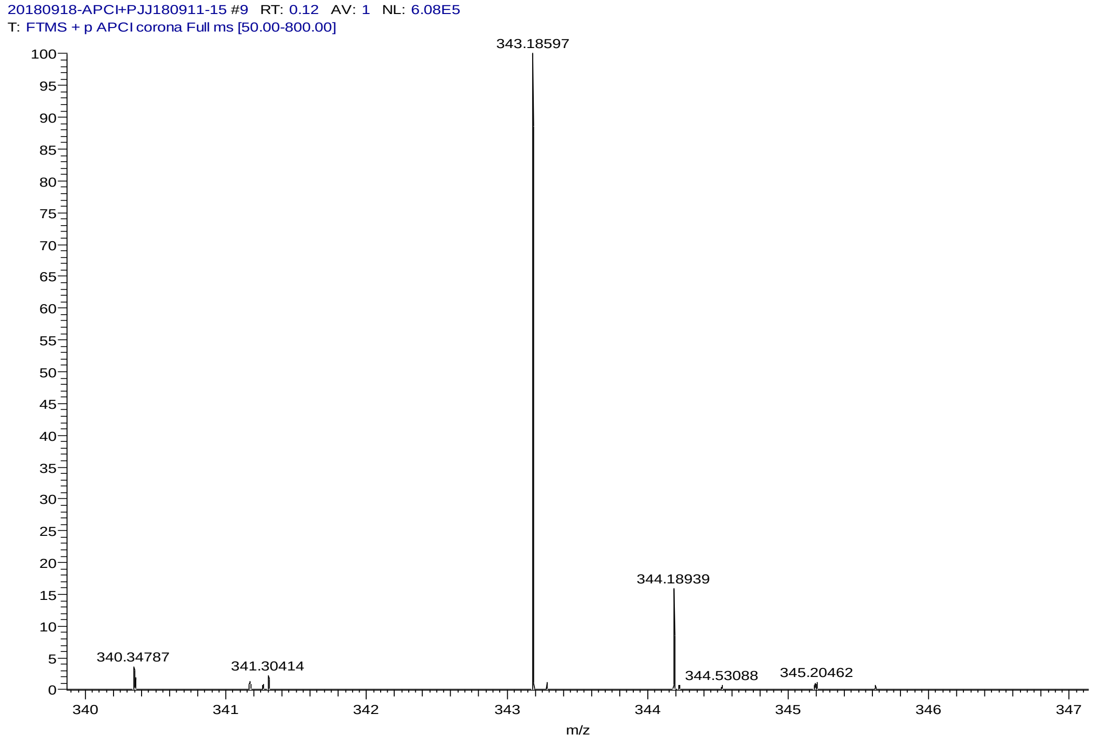

HRMS spectra for **3ca**.

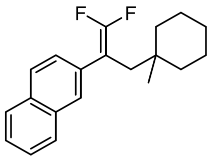

2-(1,1-difluoro-3-(1-methylcyclohexyl)prop-1-en-2-yl)naphthalene (**3db**)

Following general procedure, **1d** and **2b** were used. The product was isolated by column chromatography as colorless oil (57.7 mg, 0.192 mmol, 96%).

Selectivity (desired C-F cleavage product : addition by-product) > 50:1.

R_f (petroleum ether) = 0.88.

^{1}H NMR (400 MHz, Chloroform-*d*) δ 7.92 – 7.78 (m, 4H), 7.64 – 7.42 (m, 3H), 2.52 – 2.48 (m, 2H), 1.52 – 1.29 (m, 5H), 1.26 – 1.17 (m, 5H), 0.82 (s, 3H).

^{13}C NMR (101 MHz, Chloroform-*d*) δ 154.72 (dd, *J* = 290.1, 287.8 Hz), 133.48 (dd, *J* = 4.8, 2.6 Hz), 133.37, 132.48, 128.00, 127.93, 127.74, 127.52 (dd, *J* = 2.9, 2.9 Hz), 126.70 (dd, *J* = 2.7, 2.7 Hz), 126.28, 126.06, 90.82 (dd, *J* = 21.7, 12.9 Hz), 40.60, 38.16, 35.40 (dd, *J* = 2.5, 2.5 Hz), 26.42, 24.77, 22.13.

^{19}F NMR (376 MHz, Chloroform-*d*) δ -88.85 (d, *J* = 40.6 Hz), -91.81 (d, *J* = 40.1 Hz).

HRMS (EI) calcd for $C_{20}H_{22}F_2^+$ [M^+] 300.16841, found 300.16796.

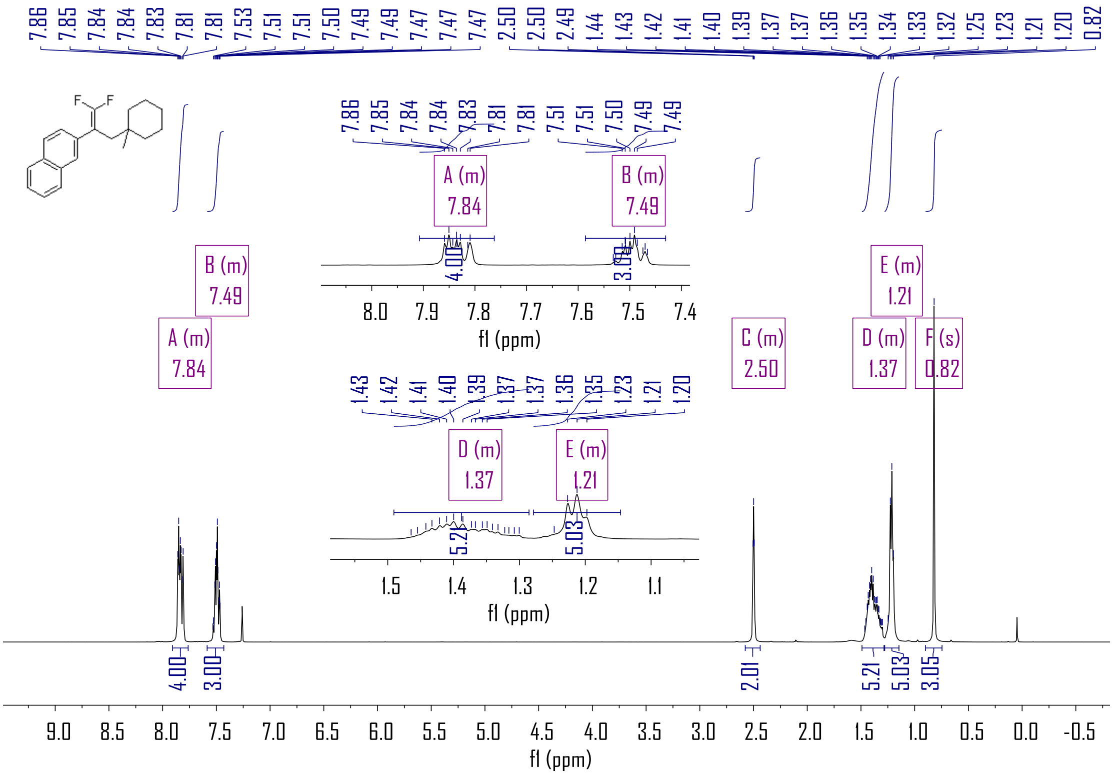

^{1}H NMR spectra for **3db**.

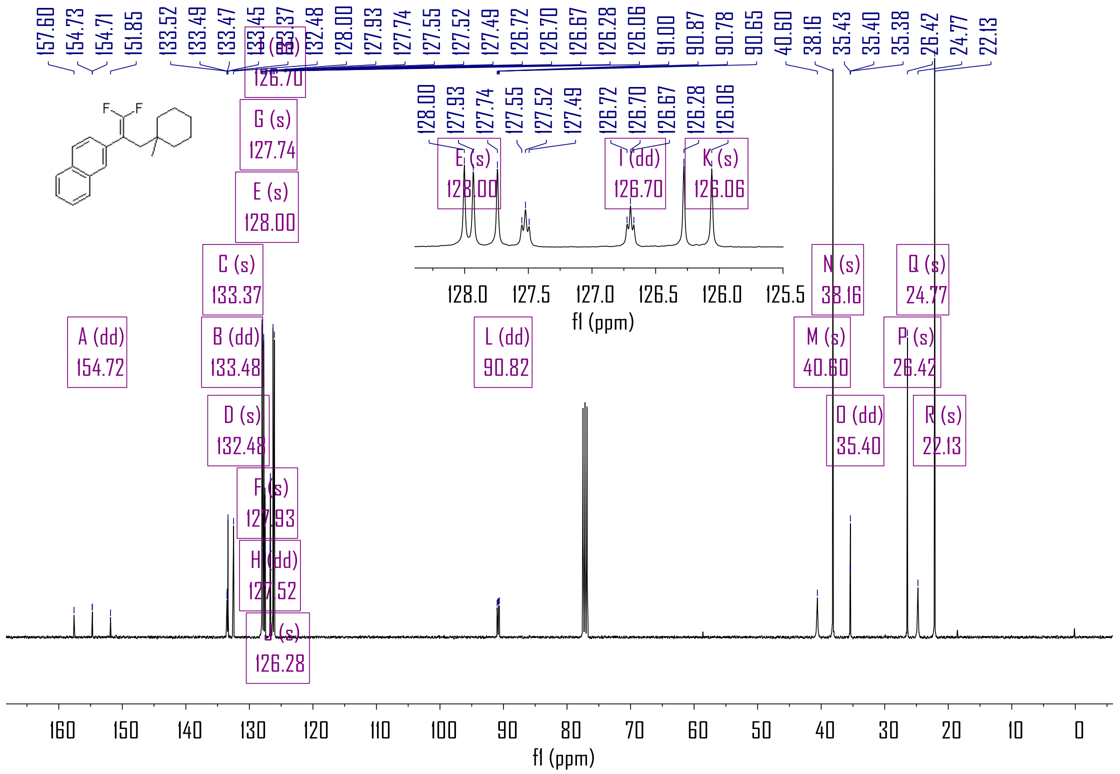

^{13}C NMR spectra for **3db**.

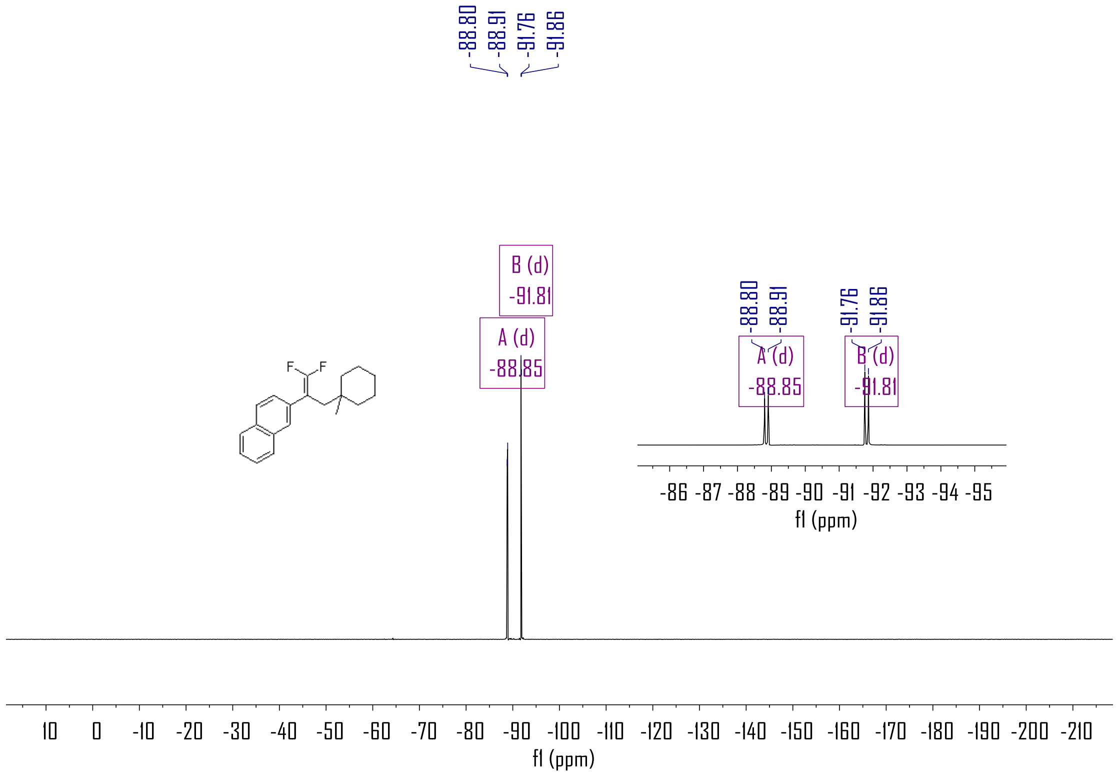

^{19}F NMR spectra for **3db**.

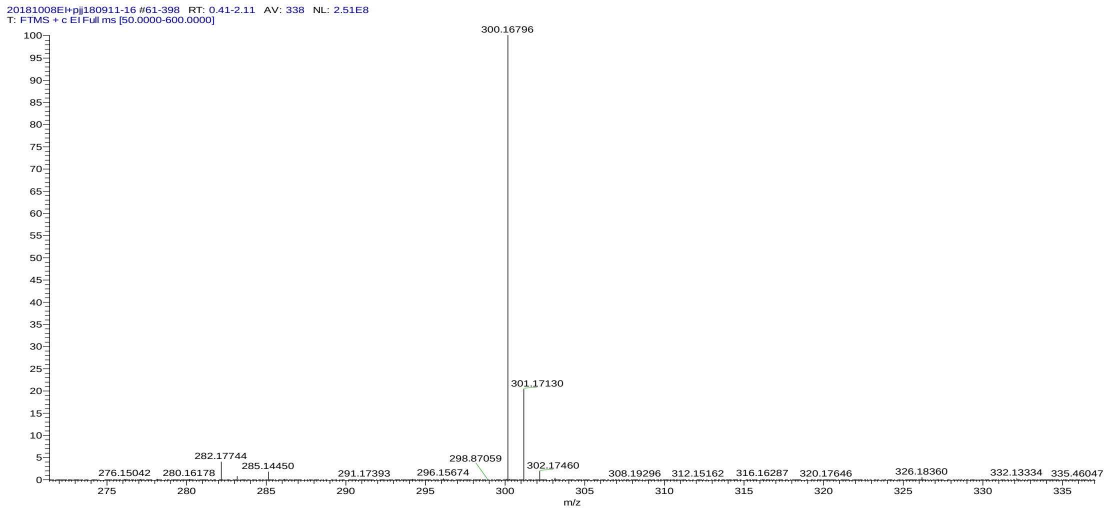

HRMS spectra for **3db**.

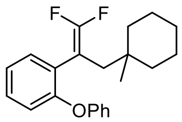

1-(1,1-difluoro-3-(1-methylcyclohexyl)prop-1-en-2-yl)-2-phenoxybenzene (**3eb**)

Following general procedure, **1e** and **2b** were used. The product was isolated by column chromatography as colorless oil (54.8 mg, 0.160 mmol, 80%).

Selectivity (desired C-F cleavage product : addition by-product) > 50:1.

R_f (petroleum ether) = 0.60.

^{1}H NMR (400 MHz, Chloroform-*d*) δ 7.39 – 7.27 (m, 3H), 7.26 – 7.17 (m, 1H), 7.15 – 7.04 (m, 2H), 7.04 – 6.96 (m, 2H), 6.86 (d, *J* = 8.2 Hz, 1H), 2.36 (s, 2H), 1.46 – 1.15 (m, 10H), 0.83 (s, 3H).

^{13}C NMR (101 MHz, Chloroform-*d*) δ 157.01, 154.79 (dd, *J* = 2.3, 1.1 Hz), 154.22 (dd, *J* = 288.1, 288.1 Hz), 131.55 (dd, *J* = 2.2, 2.2 Hz), 129.83, 128.76, 127.30 (dd, *J* = 5.1, 1.9 Hz), 123.35, 123.14, 118.89, 118.68, 86.92 (dd, *J* = 23.8, 15.3 Hz), 40.31 (brs), 37.98, 35.24 (dd, *J* = 2.5, 2.5 Hz), 26.46, 24.86 (brs), 22.16.

^{19}F NMR (376 MHz, Chloroform-*d*) δ -88.05 (d, *J* = 38.1 Hz), -90.37 (d, *J* = 38.1 Hz).

HRMS (APCI) calcd for $C_{22}H_{25}OF_2^+$ [(M+H)$^+$] 343.18680, found 343.18631.

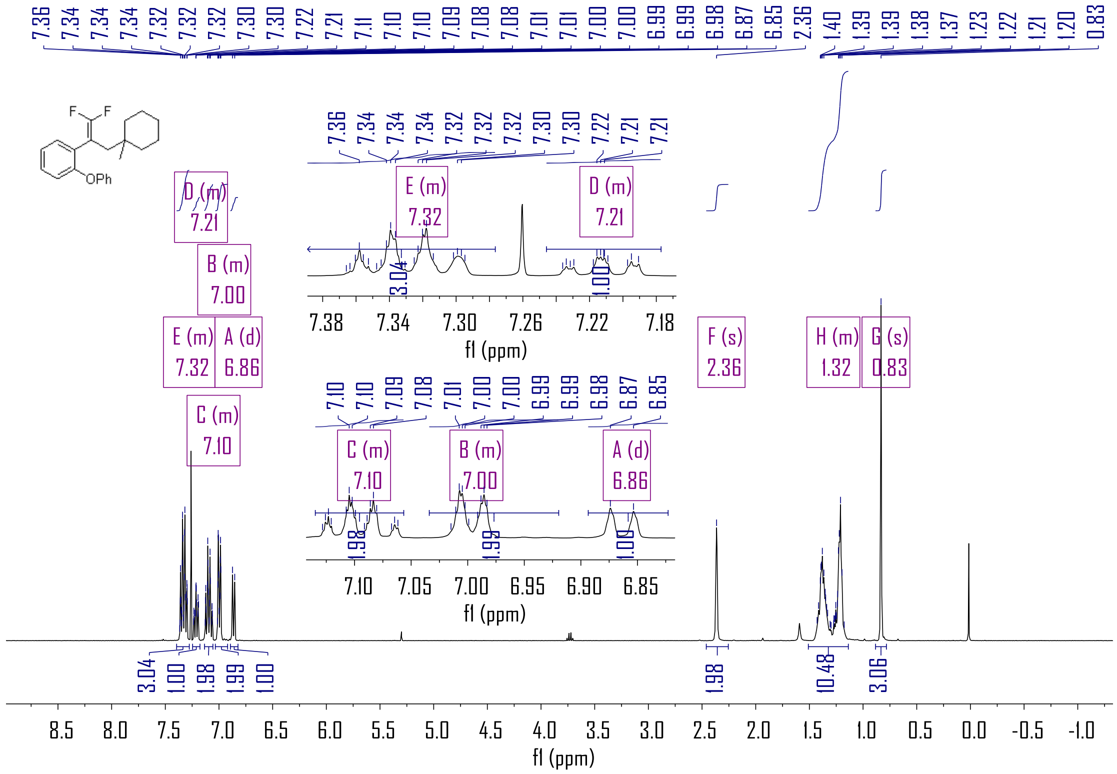

^{1}H NMR spectra for **3eb**.

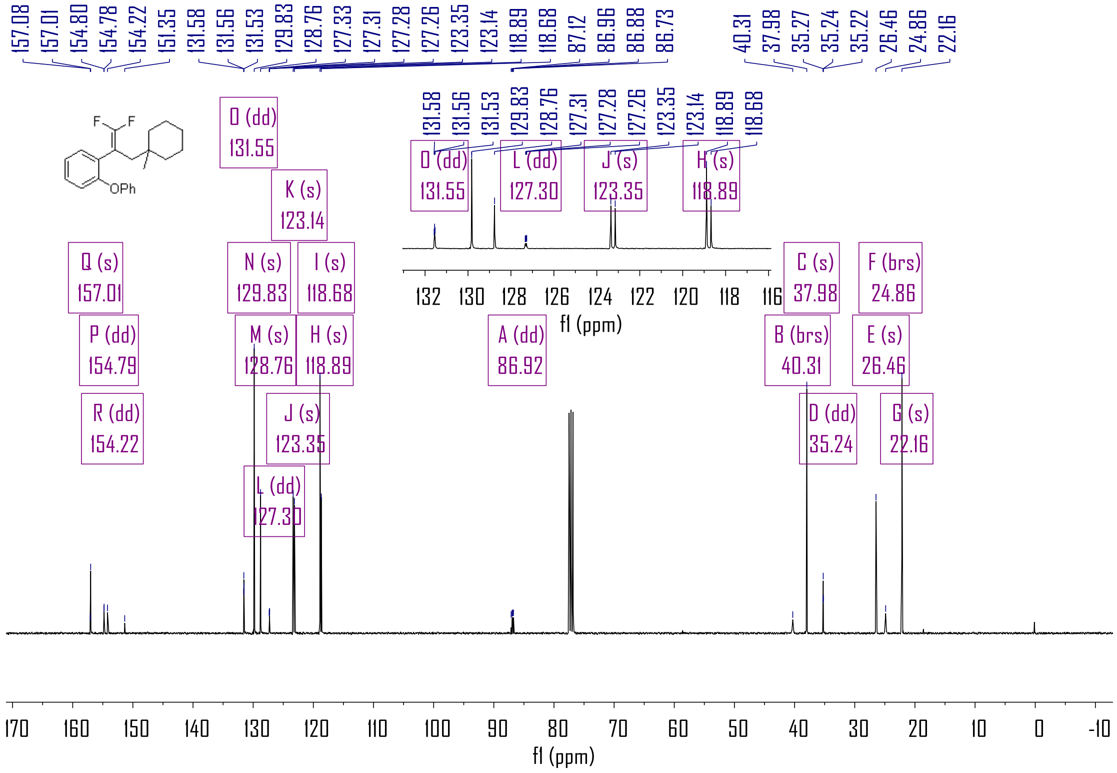

^{13}C NMR spectra for **3eb**.

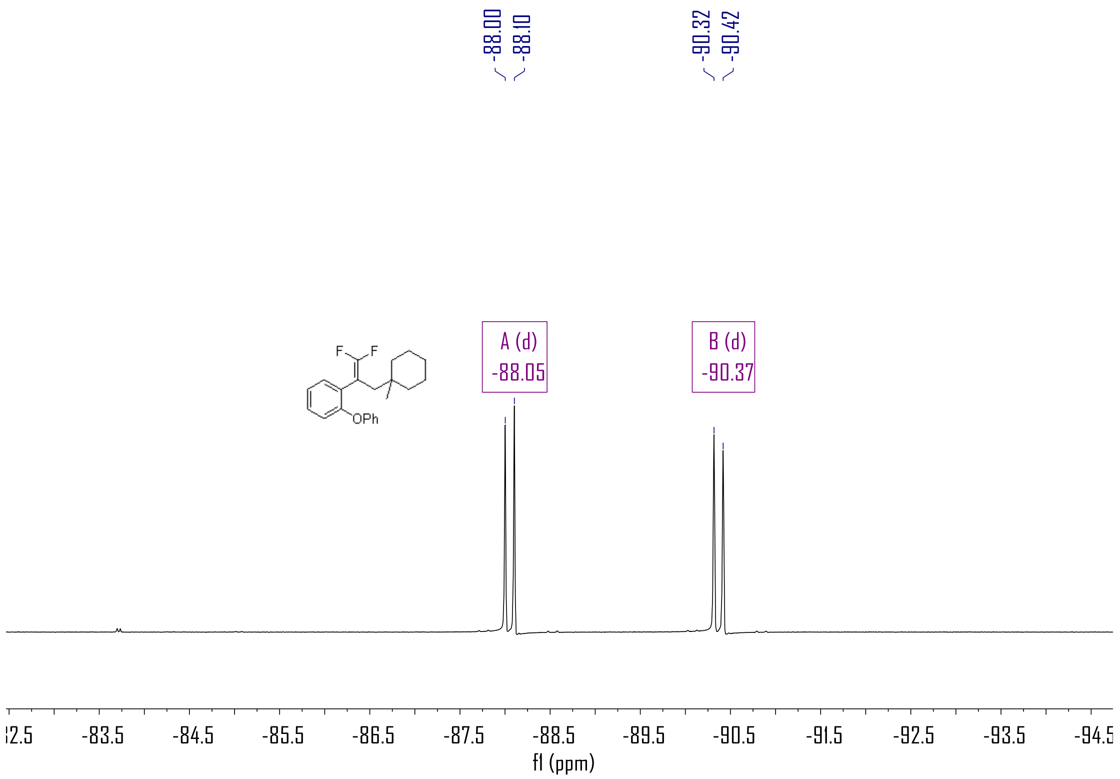

^{19}F NMR spectra for **3eb**.

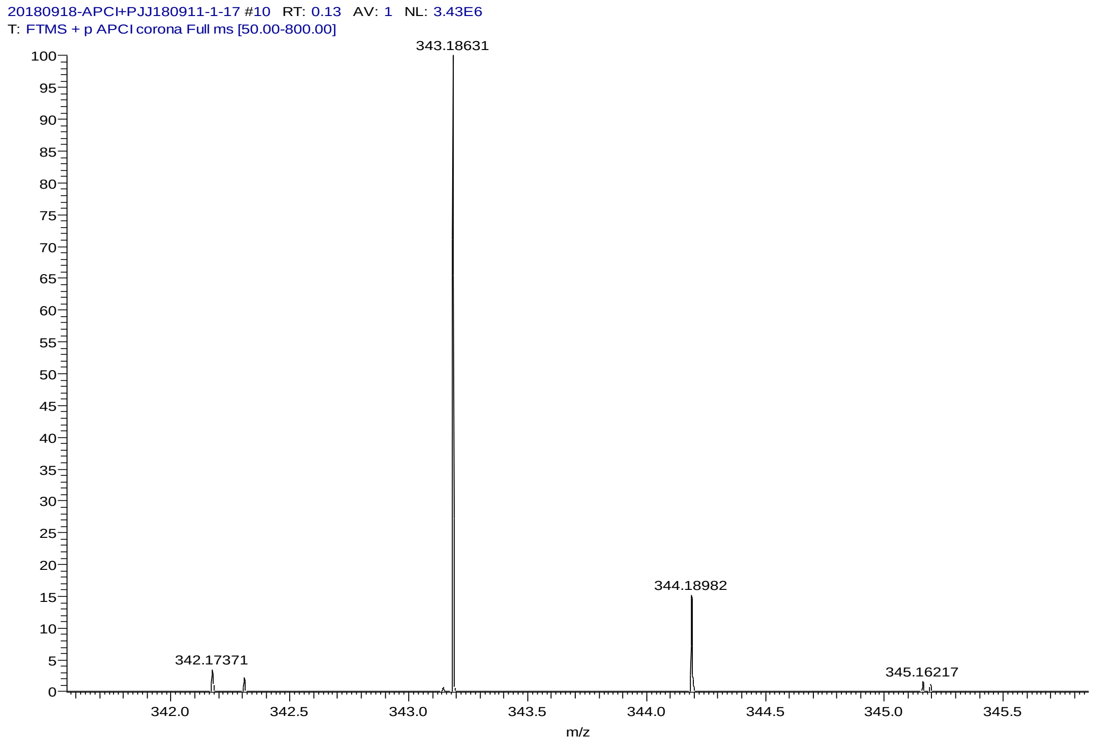

HRMS spectra for **3eb**.

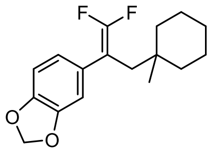

5-(1,1-difluoro-3-(1-methylcyclohexyl)prop-1-en-2-yl)benzo[*d*][1,3]dioxole (**3fb**)

Following general procedure, **1f** and **2b** were used. The product was isolated by column chromatography as colorless oil (54.1 mg, 0.184 mmol, 92%)

Selectivity (desired C-F cleavage product : addition by-product) > 50:1.

R_f (petroleum ether) = 0.65.

^{1}H NMR (400 MHz, Chloroform-*d*) δ 6.80 – 6.78 (m, 1H), 6.78 – 6.76 (m, 2H), 5.96 (s, 2H), 2.31 – 2.27 (m, 2H), 1.47 – 1.13 (m, 10H), 0.77 (s, 3H).

^{13}C NMR (101 MHz, Chloroform-*d*) δ 154.45 (dd, J = 288.9, 287.1 Hz), 147.60, 146.56, 129.66 (dd, J = 4.9, 2.5 Hz), 122.09 (dd, J = 2.8, 2.8 Hz), 109.16 (dd, J = 2.9, 2.9 Hz), 108.24, 101.19, 90.43 (dd, J = 22.2, 13.2 Hz), 40.71 (brs), 38.11, 35.29 (dd, J = 2.4, 2.4 Hz), 26.43, 24.72 (brs), 22.14.

^{19}F NMR (376 MHz, Chloroform-*d*) δ -90.02 (d, J = 42.5 Hz), -92.23 (d, J = 42.4 Hz).

HRMS (APCI) calcd for $C_{17}H_{21}O_2F_2^+$ [(M+H)$^+$] 295.15041, found 295.14966.

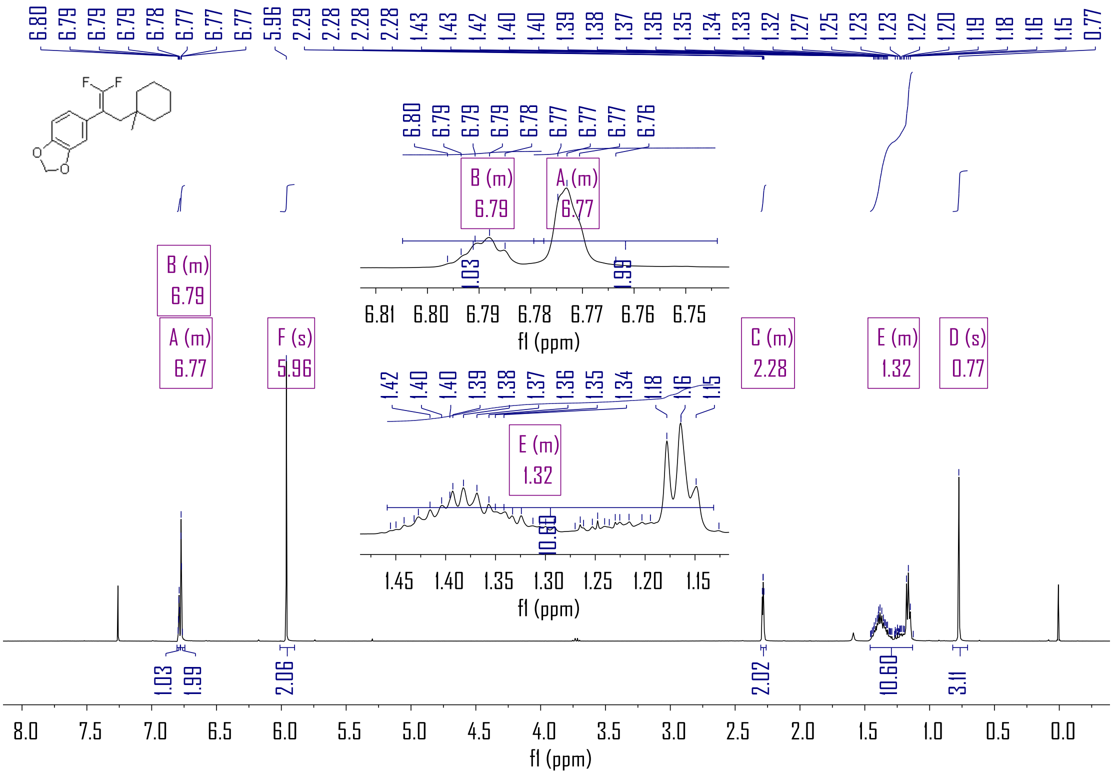

^{1}H NMR spectra for **3fb**.

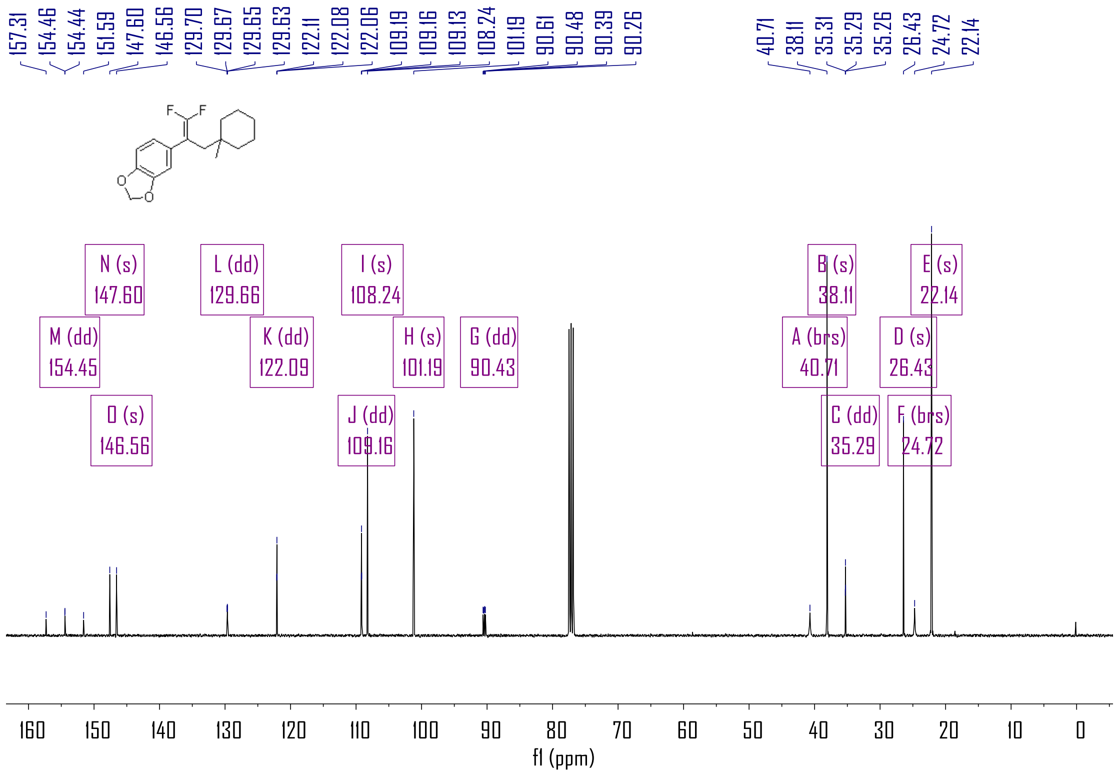

^{13}C NMR spectra for **3fb**.

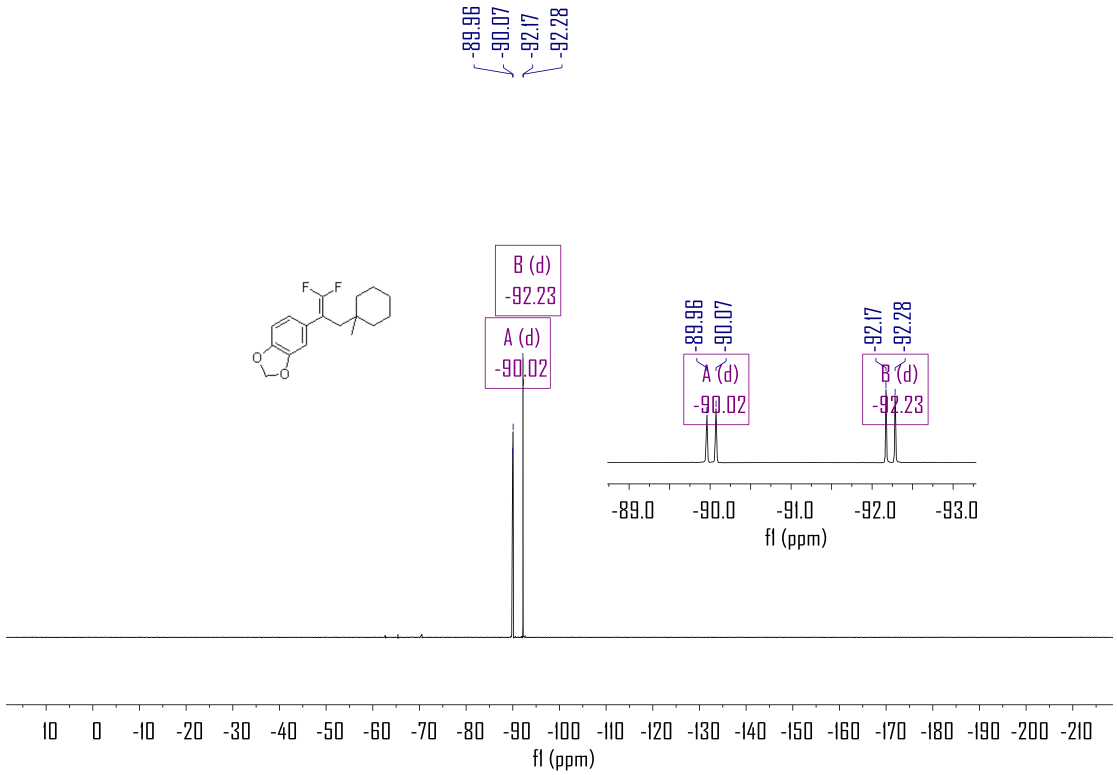

^{19}F NMR spectra for **3fb**.

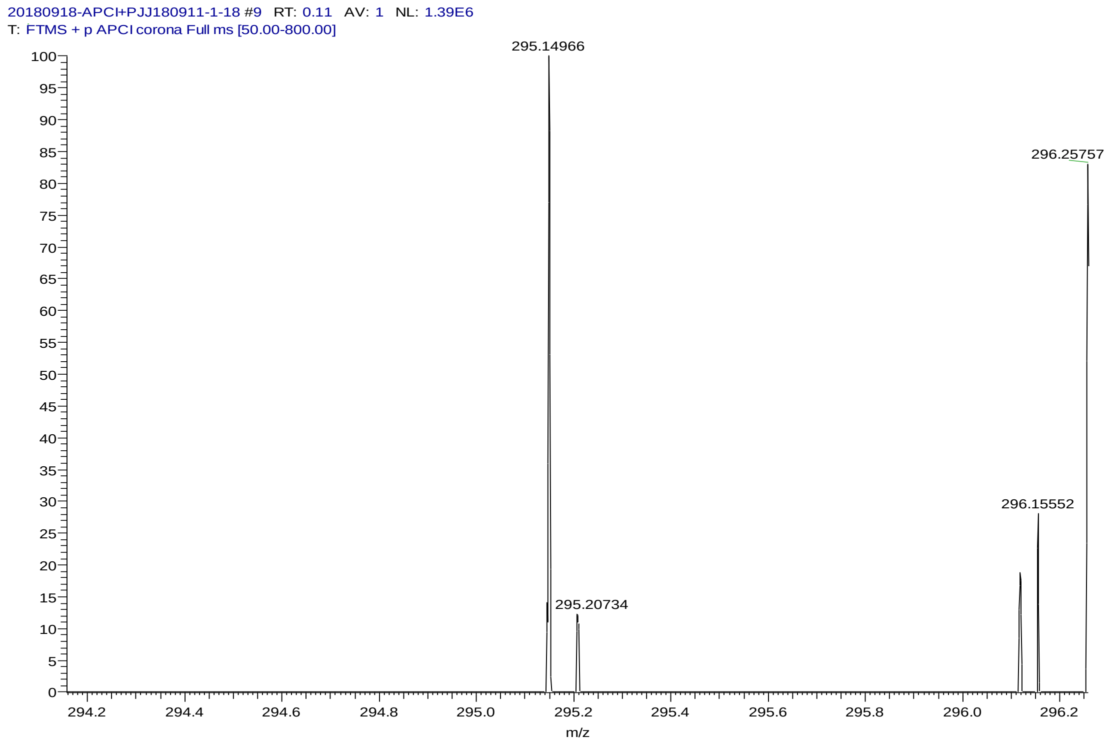

HRMS spectra for **3fb**.

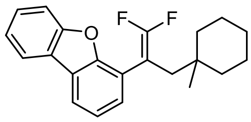

4-(1,1-difluoro-3-(1-methylcyclohexyl)prop-1-en-2-yl)dibenzo[*b*,*d*]furan (**3gb**)

Following general procedure, **1g** and **2b** were used. The product was isolated by column chromatography as colorless oil (56.5 mg, 0.166 mmol, 83%).

Selectivity (desired C-F cleavage product : addition by-product) > 50:1.

R_f (petroleum ether) = 0.70.

^{1}H NMR (400 MHz, Chloroform-*d*) δ 8.02 – 7.94 (m, 1H), 7.91 – 7.84 (m, 1H), 7.72 – 7.61 (m, 1H), 7.49 (ddt, *J* = 8.4, 7.3, 1.2 Hz, 1H), 7.43 – 7.30 (m, 3H), 2.63 – 2.57 (m, 2H), 1.44 – 1.13 (m, 10H), 0.80 (s, 3H).

^{13}C NMR (101 MHz, Chloroform-*d*) δ 156.23, 154.43 (dd, *J* = 290.7, 288.4 Hz), 153.64 (dd, *J* = 2.3, 1.6 Hz), 127.75 (dd, *J* = 2.5, 2.5 Hz), 127.35, 124.62, 124.36, 122.88, 122.77, 120.80, 120.60 (dd, *J* = 5.1, 2.1 Hz), 119.81, 112.00 , 86.16 (dd, *J* = 24.5, 14.8 Hz), 40.06, 37.99, 35.30 (dd, *J* = 2.5, 2.5 Hz), 26.42, 24.70, 22.12.

^{19}F NMR (376 MHz, Chloroform-*d*) δ -86.94 (d, *J* = 35.2 Hz), -88.91 (d, *J* = 35.3 Hz).

HRMS (APCI) calcd for $C_{22}H_{23}OF_2^+$ [(M+H)$^+$] 341.17115, found 341.16993.

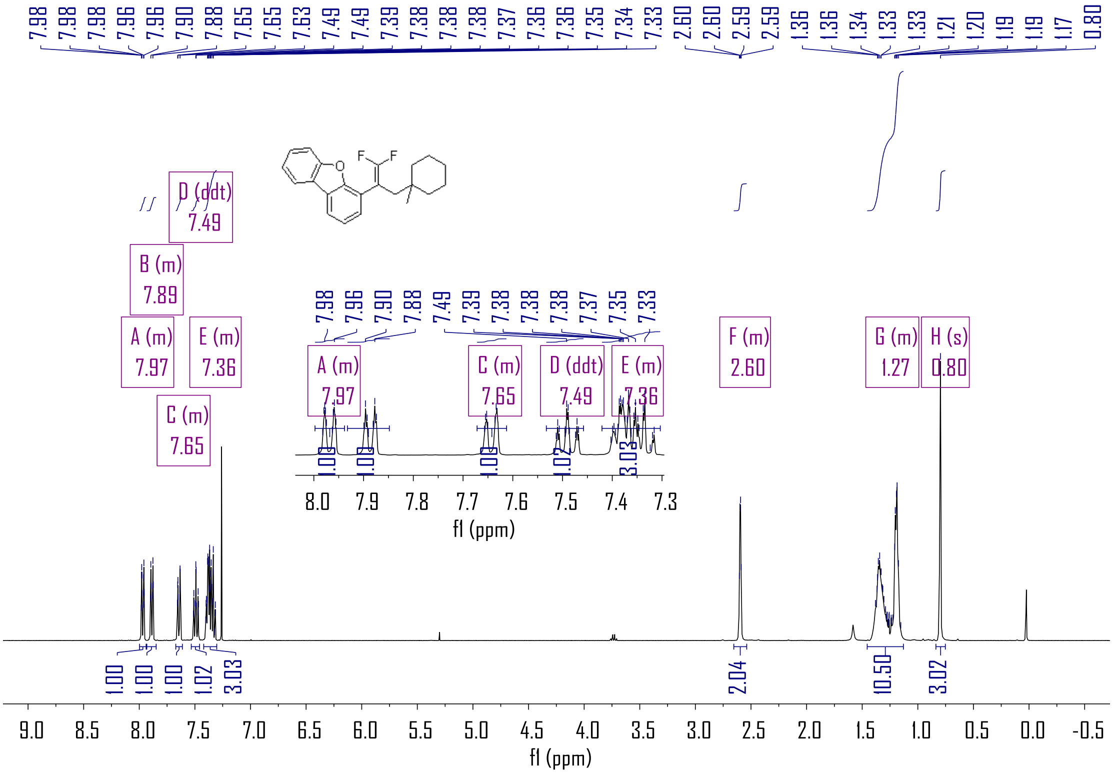

^{1}H NMR spectra for **3gb**.

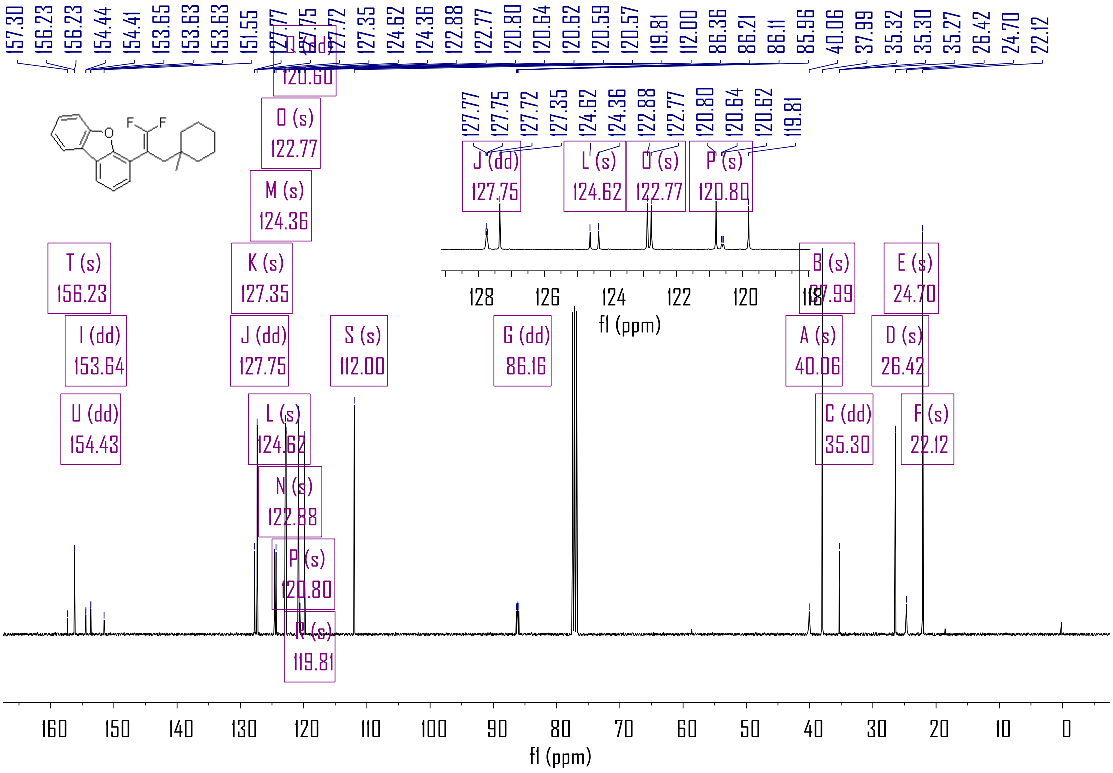

^{13}C NMR spectra for **3gb**.

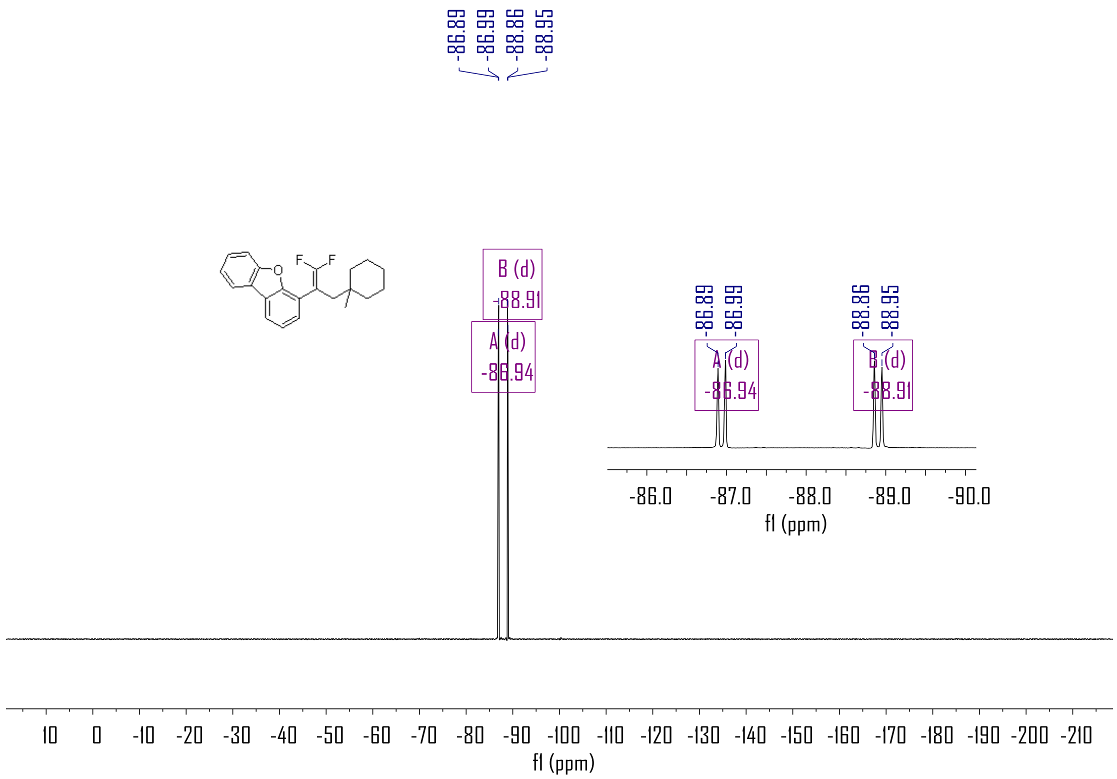

^{19}F NMR spectra for **3gb**.

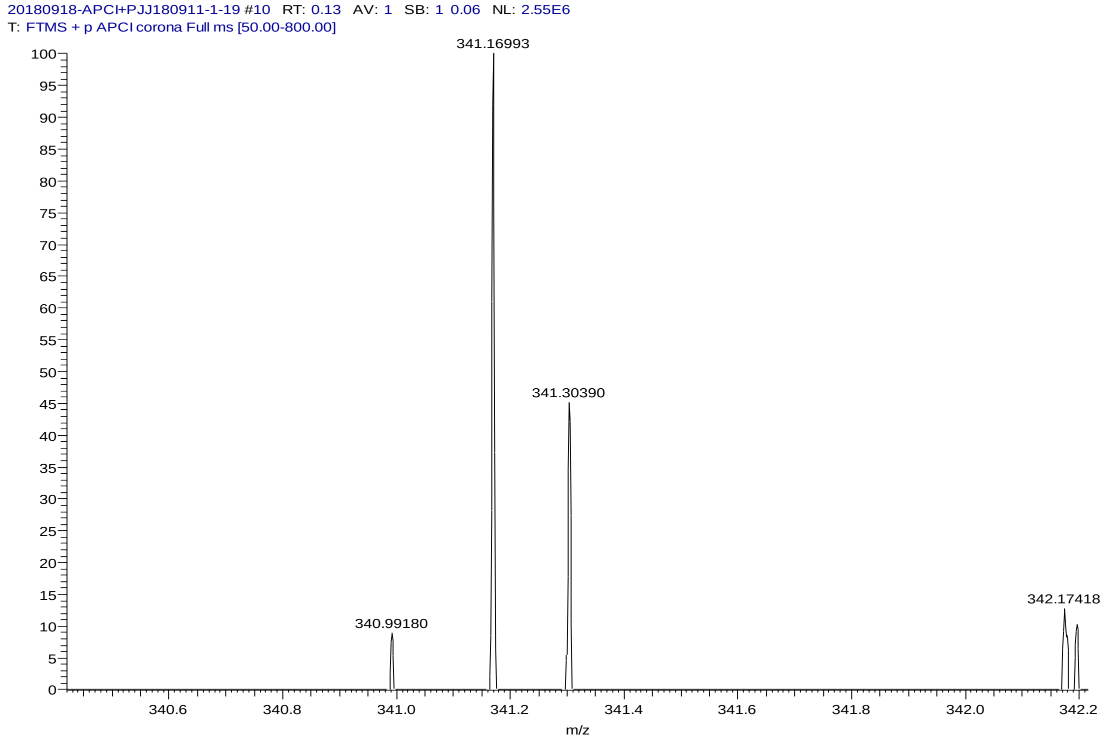

HRMS spectra for **3gb**.

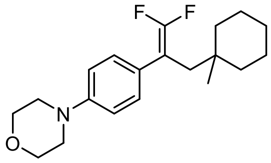

4-(4-(1,1-difluoro-3-(1-methylcyclohexyl)prop-1-en-2-yl)phenyl)morpholine (**3hb**)

Following general procedure, **1h** and **2b** were used. The product was isolated by column chromatography as colorless oil (54.3 mg, 0.162 mmol, 81%)

Selectivity (desired C-F cleavage product : addition by-product) > 50:1.

R_f (petroleum ether : ethyl acetate = 5:1) = 0.72.

^{1}H NMR (400 MHz, Chloroform-*d*) δ 7.22 (dd, *J* = 8.8, 1.4 Hz, 2H), 6.88 (d, *J* = 8.3 Hz, 2H), 3.87 (t, *J* = 4.7 Hz, 4H), 3.18 (t, *J* = 5.0 Hz, 4H), 2.34 – 2.28 (m, 2H), 1.48 – 1.10 (m, 10H), 0.76 (s, 3H).

^{13}C NMR (101 MHz, Chloroform-*d*) δ 154.42 (dd, *J* = 288.7, 286.9 Hz), 149.87, 129.36 (dd, *J* = 2.9, 2.9 Hz), 127.32, 115.32, 90.14 (dd, *J* = 21.5, 13.2 Hz), 67.00, 49.26, 40.33 (brs), 38.15, 35.26 (dd, *J* = 2.6, 2.6 Hz), 26.45, 24.78 (brs), 22.16.

^{19}F NMR (376 MHz, Chloroform-*d*) δ -90.35 (d, *J* = 44.2 Hz), -93.09 (d, *J* = 43.9 Hz).

HRMS (ESI) calcd for $C_{20}H_{28}ONF_2^+$ [(M+H)$^+$] 336.21335, found 336.21313.

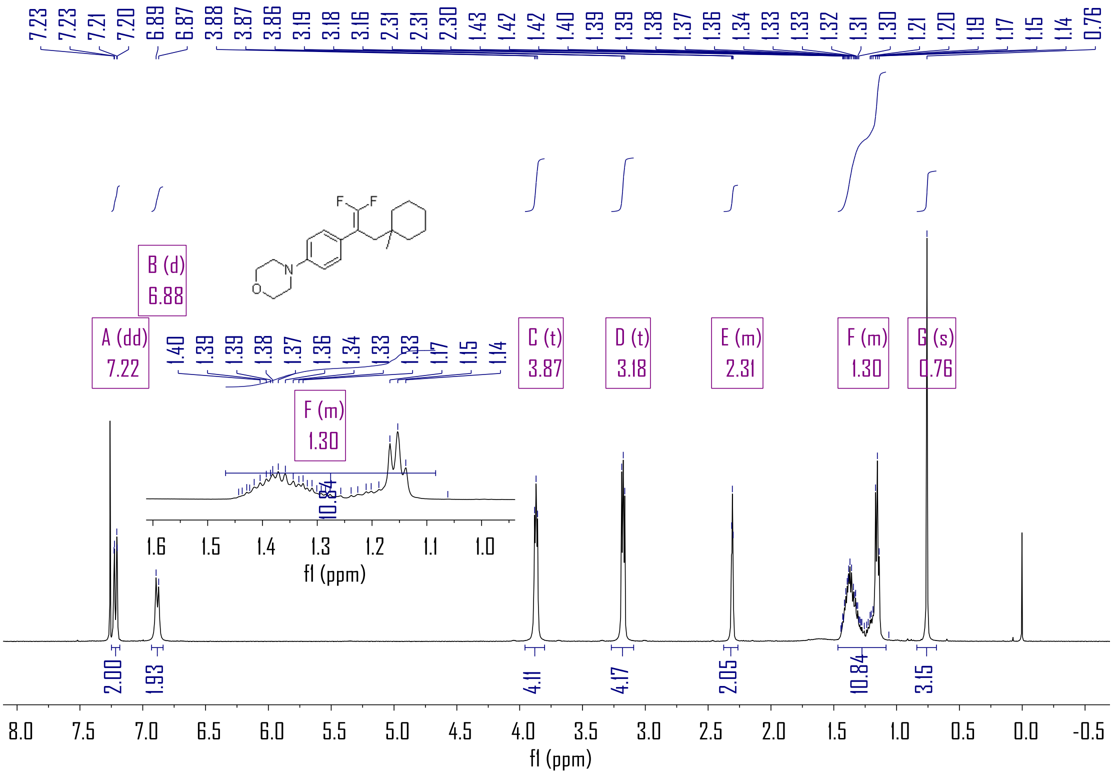

^{1}H NMR spectra for **3hb**.

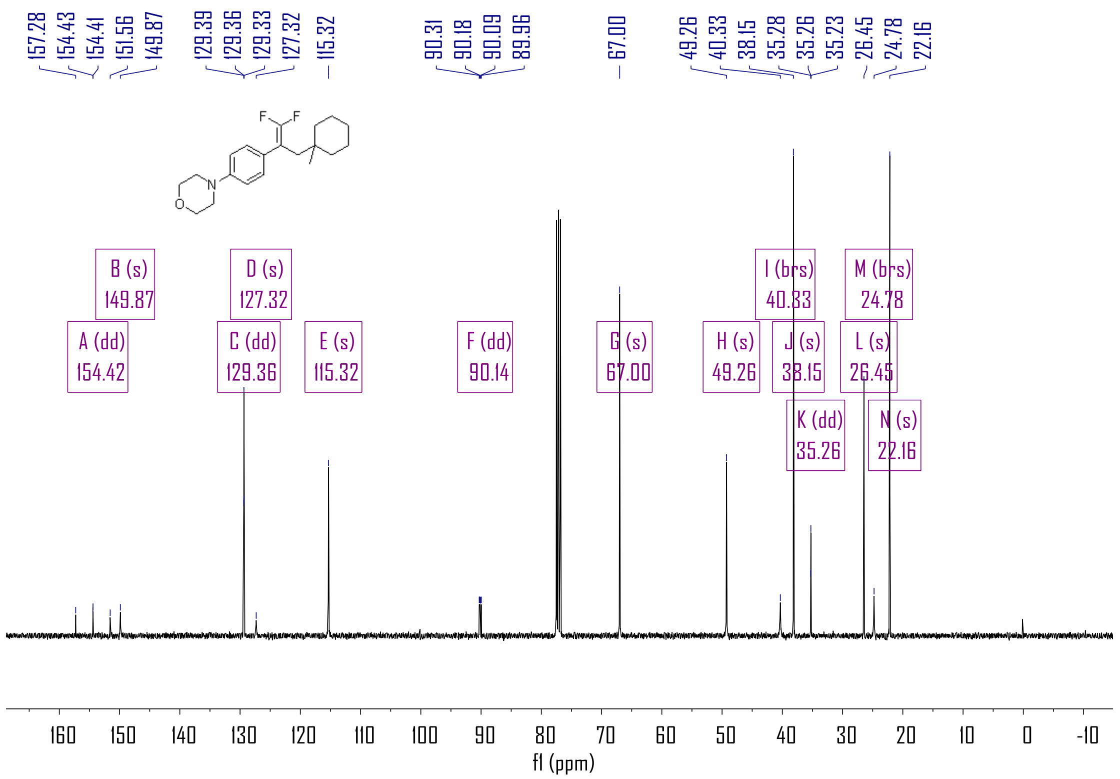

^{13}C NMR spectra for **3hb**.

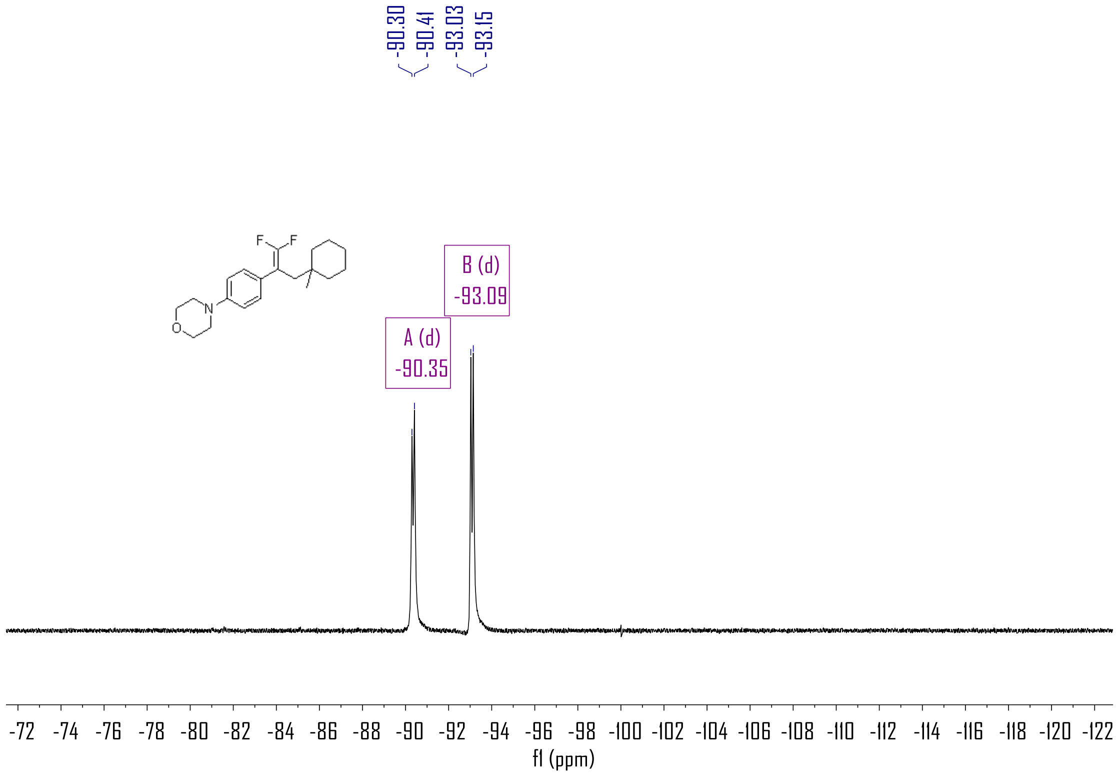

^{19}F NMR spectra for **3hb**.

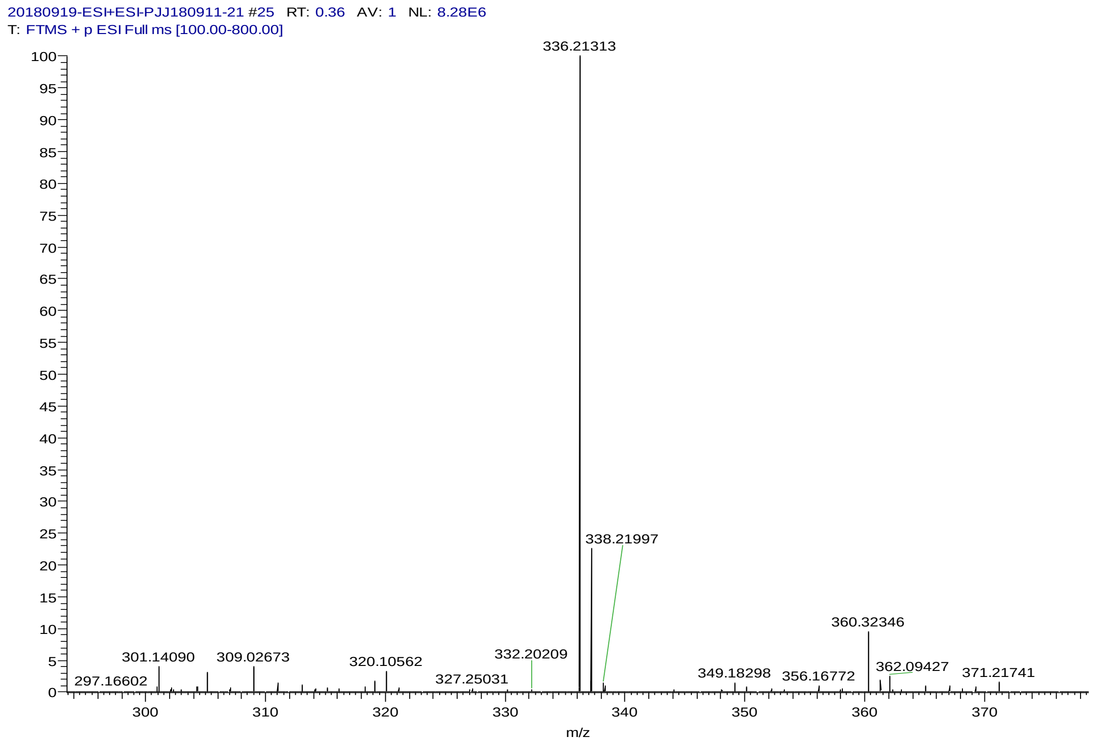

HRMS spectra for **3hb**.

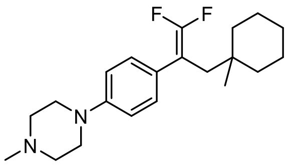

1-(4-(1,1-difluoro-3-(1-methylcyclohexyl)prop-1-en-2-yl)phenyl)-4-methylpiperazine (**3ib**)

Following general procedure, **1i** and **2b** were used. The product was isolated by column chromatography as colorless oil (47.4 mg, 0.136 mmol, 68%).

Selectivity (desired C-F cleavage product : addition by-product) > 50:1.

R_f (tetrahydrofuran) = 0.65.

^{1}H NMR (400 MHz, Chloroform-*d*) δ 7.23 – 7.15 (m, 2H), 6.93 – 6.81 (m, 2H), 3.26 (t, *J* = 5.0 Hz, 4H), 2.66 (t, *J* = 5.0 Hz, 4H), 2.40 (s, 3H), 2.31 – 2.28 (m, 2H), 1.41 – 1.09 (m, 10H), 0.75 (s, 3H).

^{13}C NMR (101 MHz, Chloroform-*d*) δ 154.39 (dd, *J* = 289.1, 287.1 Hz), 149.73, 129.30 (dd, *J* = 2.9, 2.9 Hz), 127.05 (dd, *J* = 4.6, 2.7 Hz), 115.72, 90.14 (dd, *J* = 21.5, 13.3 Hz), 55.04, 48.63, 45.96, 40.33, 38.13, 35.24 (dd, *J* = 2.3, 2.3 Hz), 26.45, 24.77, 22.15.

^{19}F NMR (376 MHz, Chloroform-*d*) δ -90.42 (d, *J* = 43.9 Hz), -93.15 (d, *J* = 43.8 Hz).

HRMS (ESI) calcd for $C_{21}H_{31}N_2F_2^+$ $[(M+H)^+]$ 349.24498, found 349.24448.

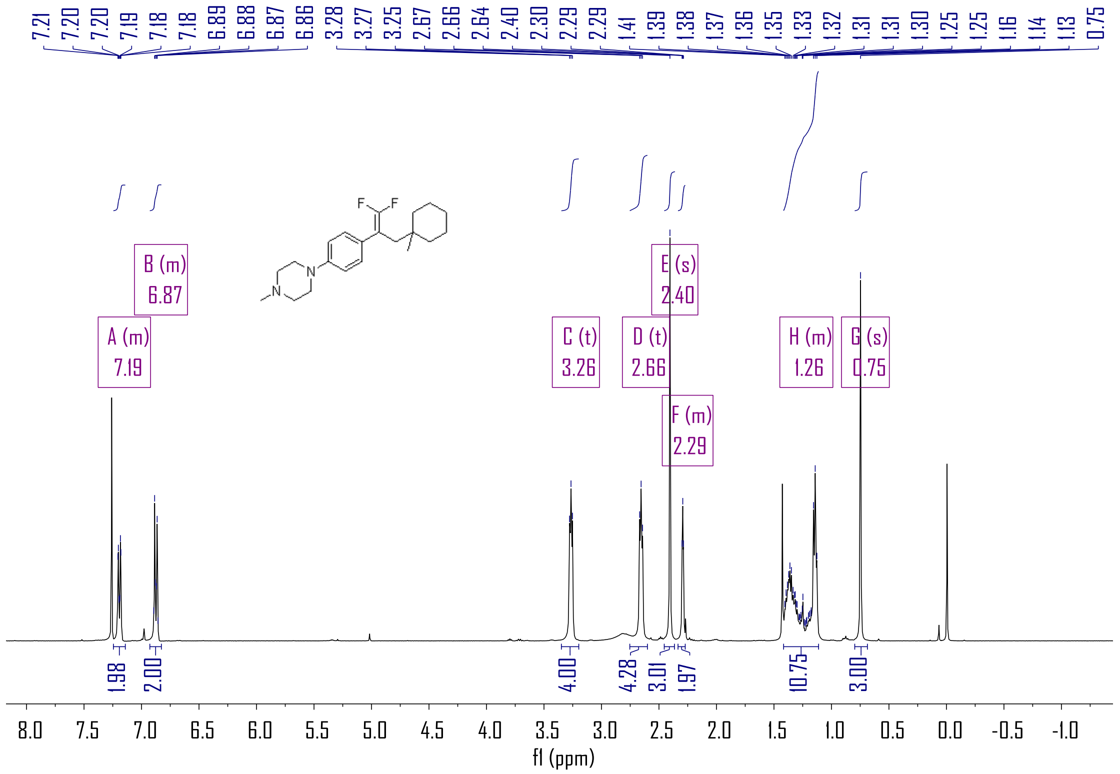

^{1}H NMR spectra for **3ib**.

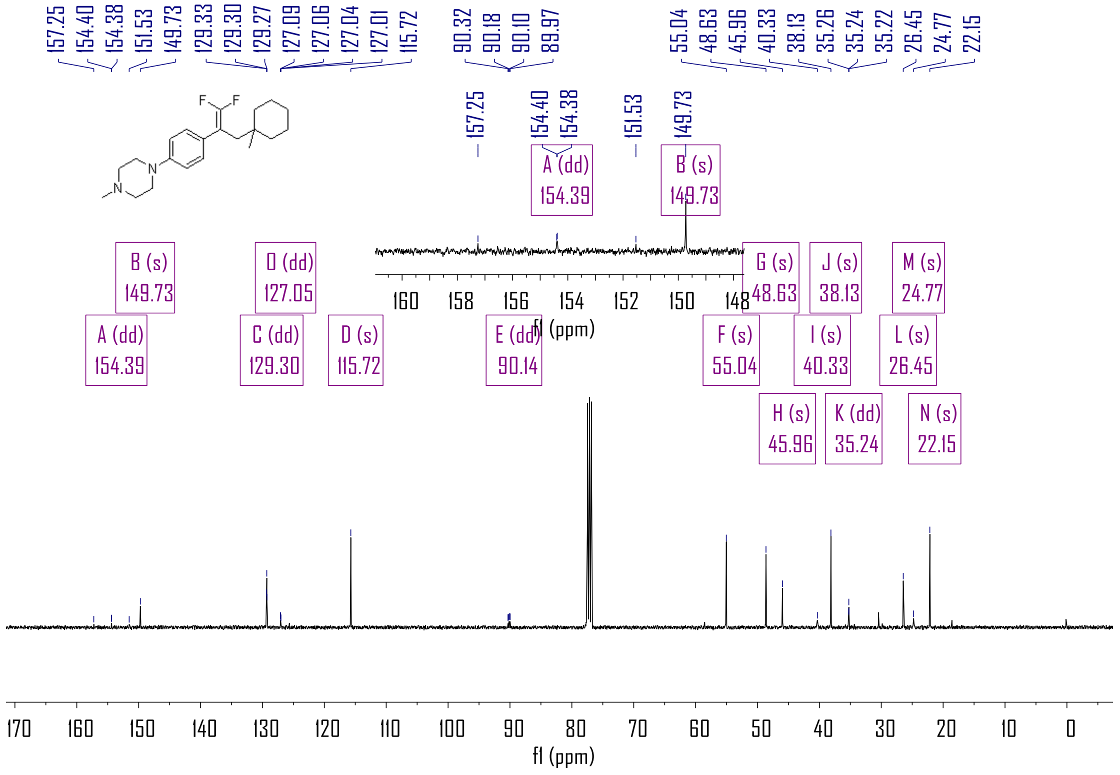

^{13}C NMR spectra for **3ib**.

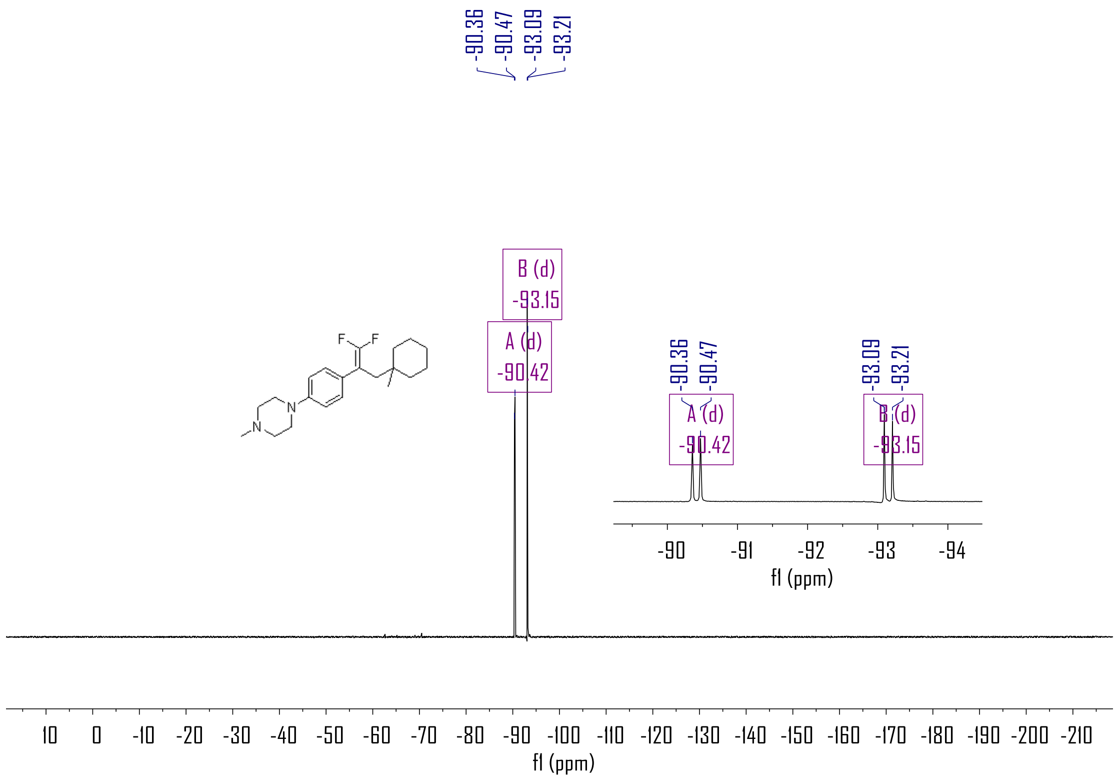

^{19}F NMR spectra for **3ib**.

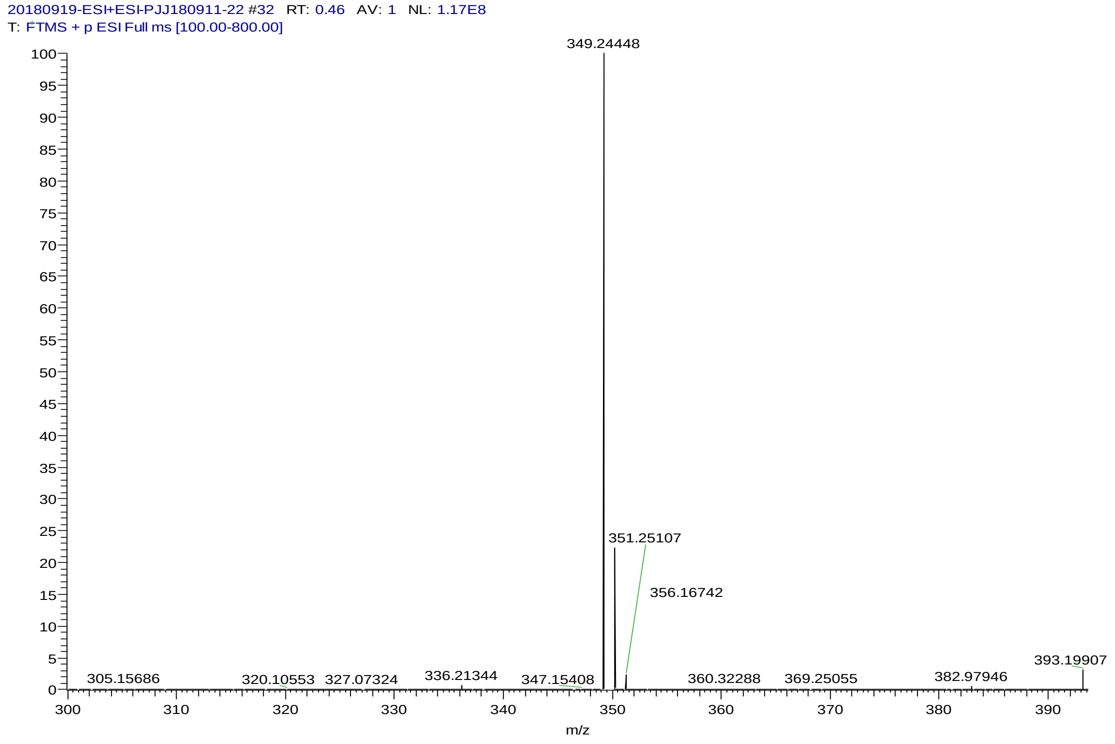

HRMS spectra for **3ib**.

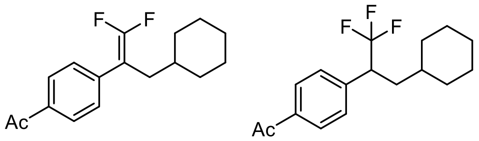

3ja 3ja'

1-(4-(3-cyclohexyl-1,1-difluoroprop-1-en-2-yl)phenyl)ethan-1-one (**3ja**)

1-(4-(3-cyclohexyl-1,1,1-trifluoropropan-2-yl)phenyl)ethan-1-one (**3ja'**)

Following general procedure, **1j** and **2a** were used. The product was isolated by column chromatography as colorless oil, 32.4 mg inseparable mixture was obtained, **3ja** (0.108 mmol, 54%) and **3ja'** (0.008 mmol, 4%).

Selectivity (desired C-F cleavage product : addition by-product) = 14:1.

R_f (petroleum ether : ethyl acetate = 20:1) = 0.60.

^{1}H NMR (400 MHz, Chloroform-*d*) δ 7.94 (d, *J* = 8.5 Hz, 2H), 7.48 – 7.38 (m, 2H), 2.60 (s, 3H), 2.31 (ddd, *J* = 7.3, 2.9, 2.0 Hz, 2H), 1.80 – 1.54 (m, 6H), 1.32 – 1.17 (m, 1H), 1.16 – 1.05 (m, 2H), 0.99 – 0.75 (m, 2H).

^{13}C NMR (101 MHz, Chloroform-*d*) δ 197.67, 154.34 (dd, *J* = 292.6, 287.8 Hz), 139.37 (dd, *J* = 5.0, 3.6 Hz), 135.85, 128.57, 128.50 (dd, *J* = 3.5, 3.5 Hz), 91.00 (dd, *J* = 22.9, 11.7 Hz), 35.99 (dd, *J* = 2.4, 2.4 Hz), 34.95, 32.96, 26.71, 26.45, 26.14.

^{19}F NMR (376 MHz, Chloroform-*d*) (**3ja**) δ -88.75 (d, *J* = 38.6 Hz), -89.50 (d, *J* = 38.7 Hz).

^{19}F NMR (376 MHz, Chloroform-*d*) (**3ja'**) δ -69.57 (d, *J* = 9.3 Hz).

HRMS (APCI) calcd for $C_{17}H_{21}OF_2^+$ [(M+H)$^+$] 279.15550, found 279.15533.

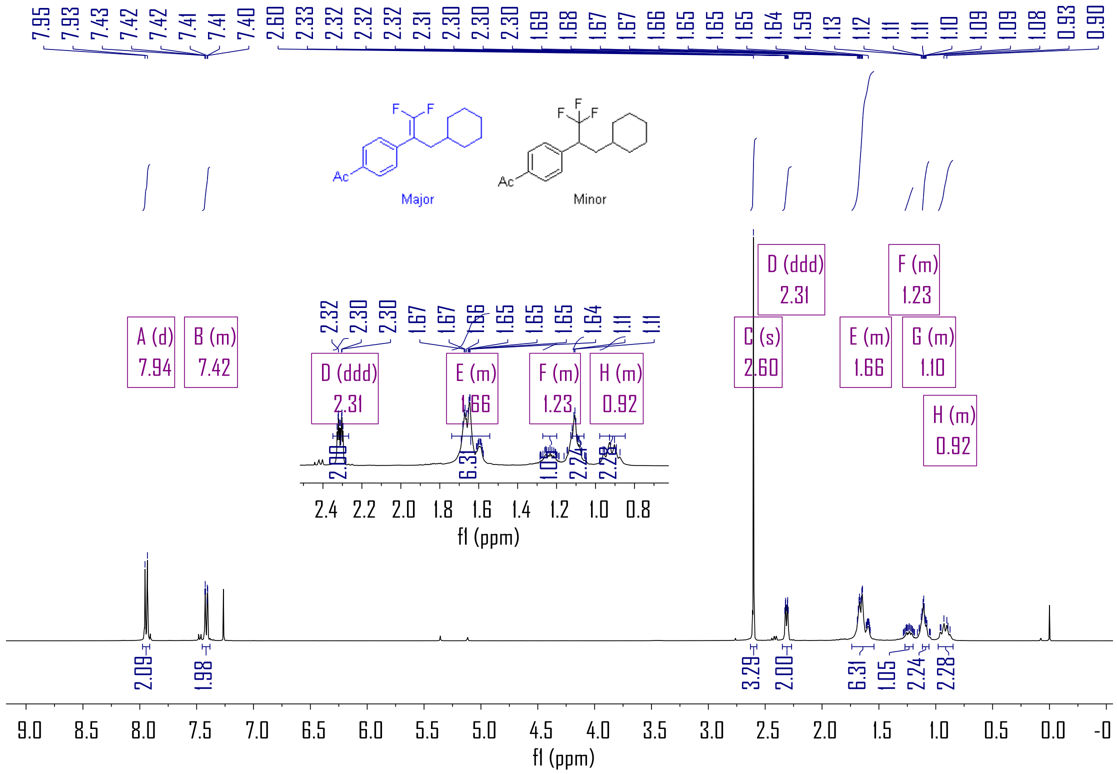

^{1}H NMR spectra for **3ja** & **3ja’**.

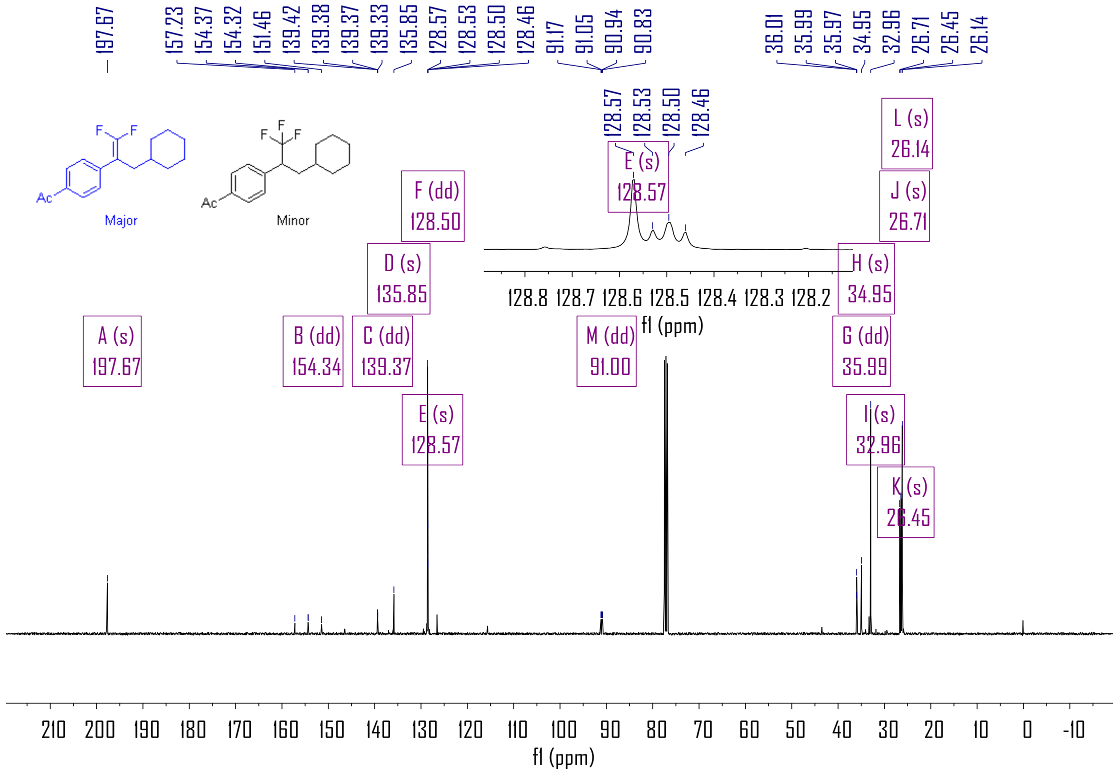

^{13}C NMR spectra for **3ja** & **3ja’**.

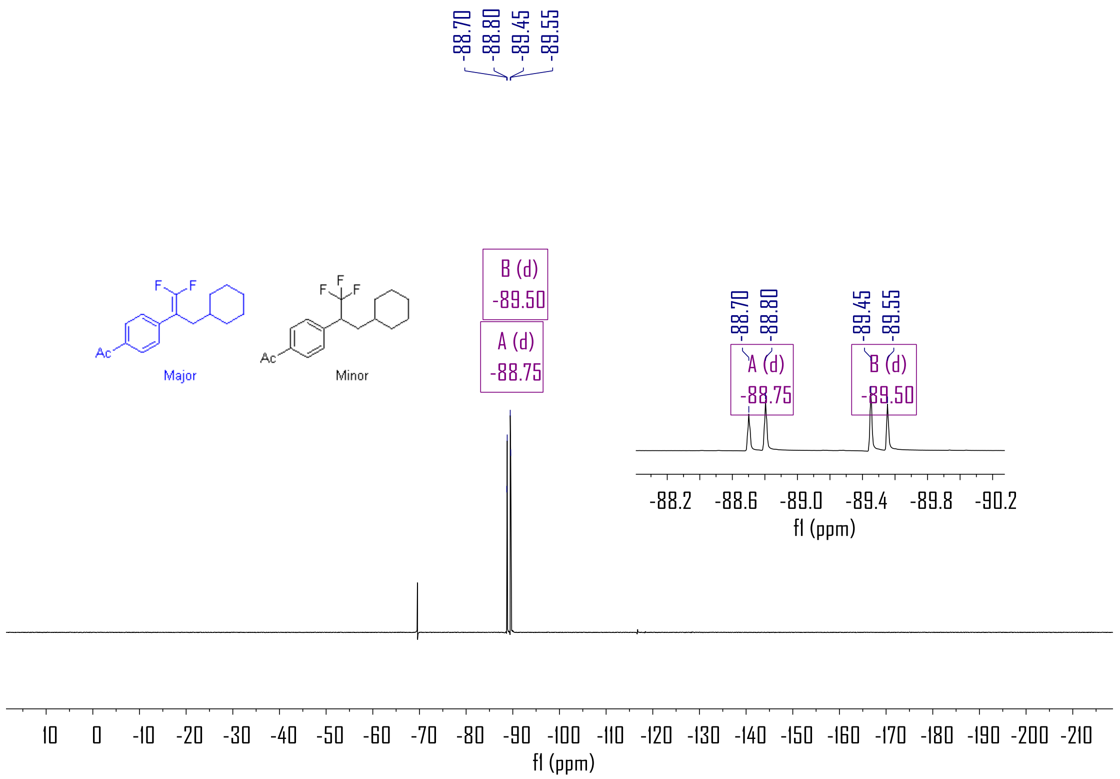

^{19}F NMR spectra for **3ja**.

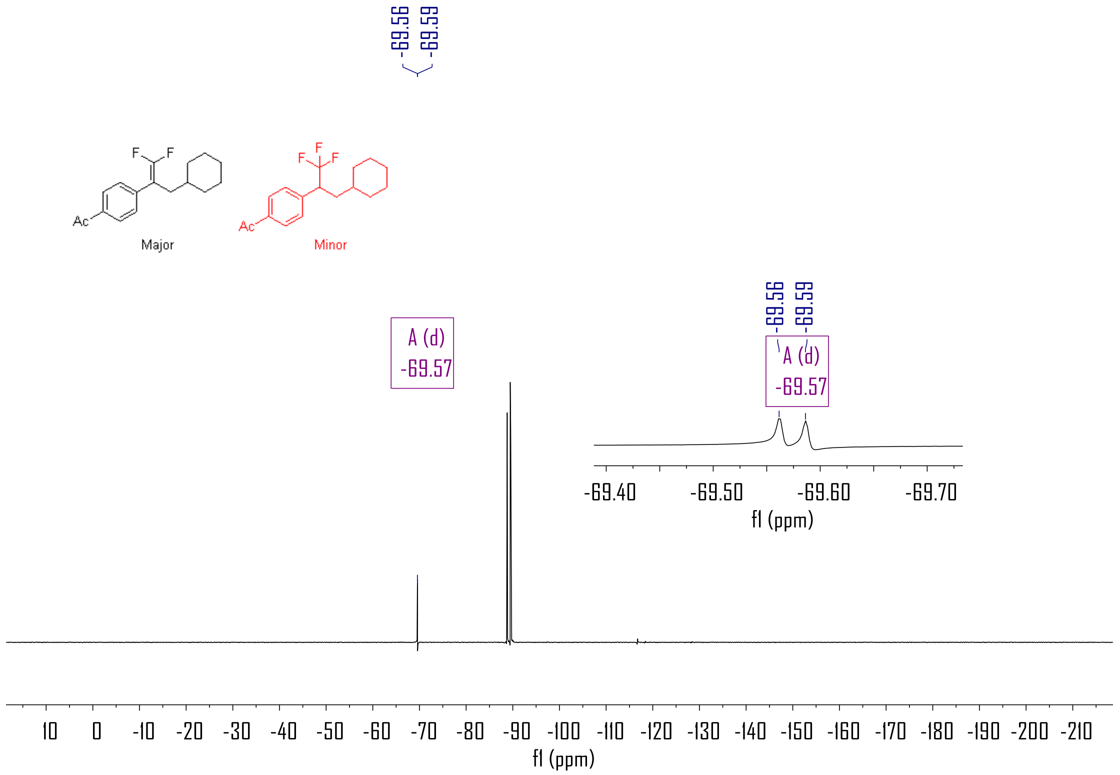

^{19}F NMR spectra for **3ja'**.

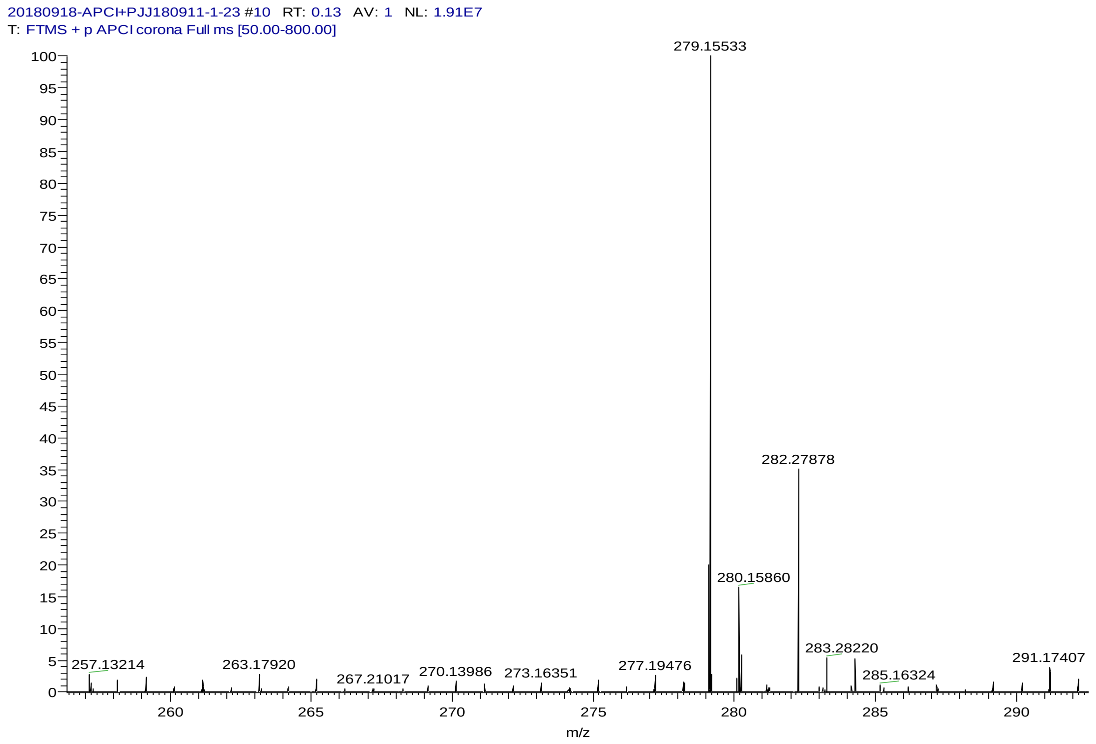

HRMS spectra for **3ja**.

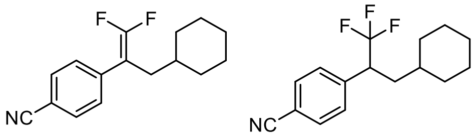

3ka **3ka'**

4-(3-cyclohexyl-1,1-difluoroprop-1-en-2-yl)benzonitrile (**3ka**)

4-(3-cyclohexyl-1,1,1-trifluoropropan-2-yl)benzonitrile (**3ka'**)

Following general procedure, **1k** and **2a** were used. The product was isolated by column chromatography as colorless oil, 44.6 mg inseparable mixture was obtained, **3ka** (0.166 mmol, 83%).

Selectivity (desired C-F cleavage product : addition by-product) = 35:1.

R_f (petroleum ether : ethyl acetate = 20:1) = 0.65.

^{1}H NMR (400 MHz, Chloroform-*d*) δ 7.64 (d, *J* = 8.5 Hz, 2H), 7.42 (dd, *J* = 8.8, 1.2 Hz, 2H), 2.30 (ddd, *J* = 7.3, 2.9, 2.1 Hz, 2H), 1.73 – 1.53 (m, 5H), 1.28 – 1.16 (m, 1H), 1.15 – 1.04 (m, 3H), 0.98 – 0.83 (m, 2H).

^{13}C NMR (101 MHz, Chloroform-*d*) δ 154.42 (dd, *J* = 293.4, 288.6 Hz), 139.29 (dd, *J* = 5.0, 3.8 Hz), 132.31, 128.96 (dd, *J* = 3.6, 3.6 Hz), 118.79, 110.90, 90.70 (dd, *J* = 23.5, 11.3 Hz), 35.96 (dd, *J* = 2.2, 2.2 Hz), 34.76, 32.91, 26.37, 26.08.

^{19}F NMR (376 MHz, Chloroform-*d*) (**3ka**) δ -87.63 (d, *J* = 36.5 Hz), -88.68 (d, *J* = 36.5 Hz).

^{19}F NMR (376 MHz, Chloroform-*d*) (**3ka'**) δ -69.57 (d, *J* = 9.1 Hz).

<u>HRMS (APCI)</u> calcd for $C_{16}H_{18}NF_2^+$ [(M+H)$^+$] 262.14018, found 262.13950.

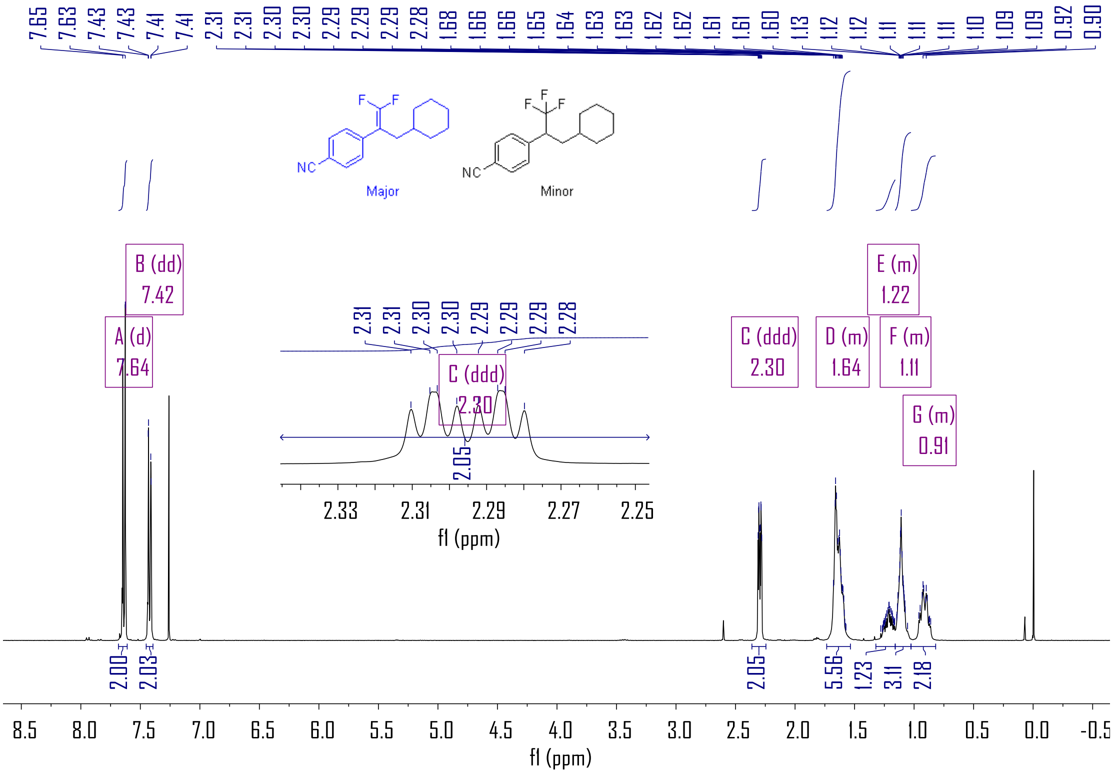

^{1}H NMR spectra for **3ka** & **3ka'**.

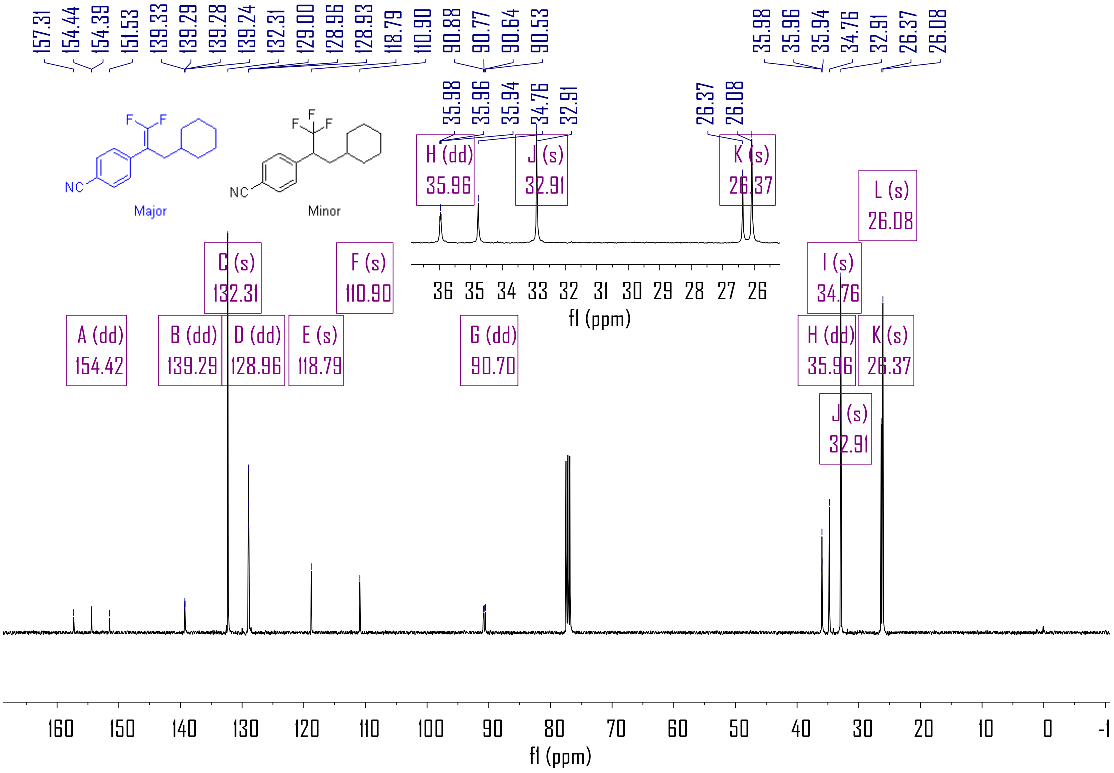

^{13}C NMR spectra for **3ka** & **3ka'**.

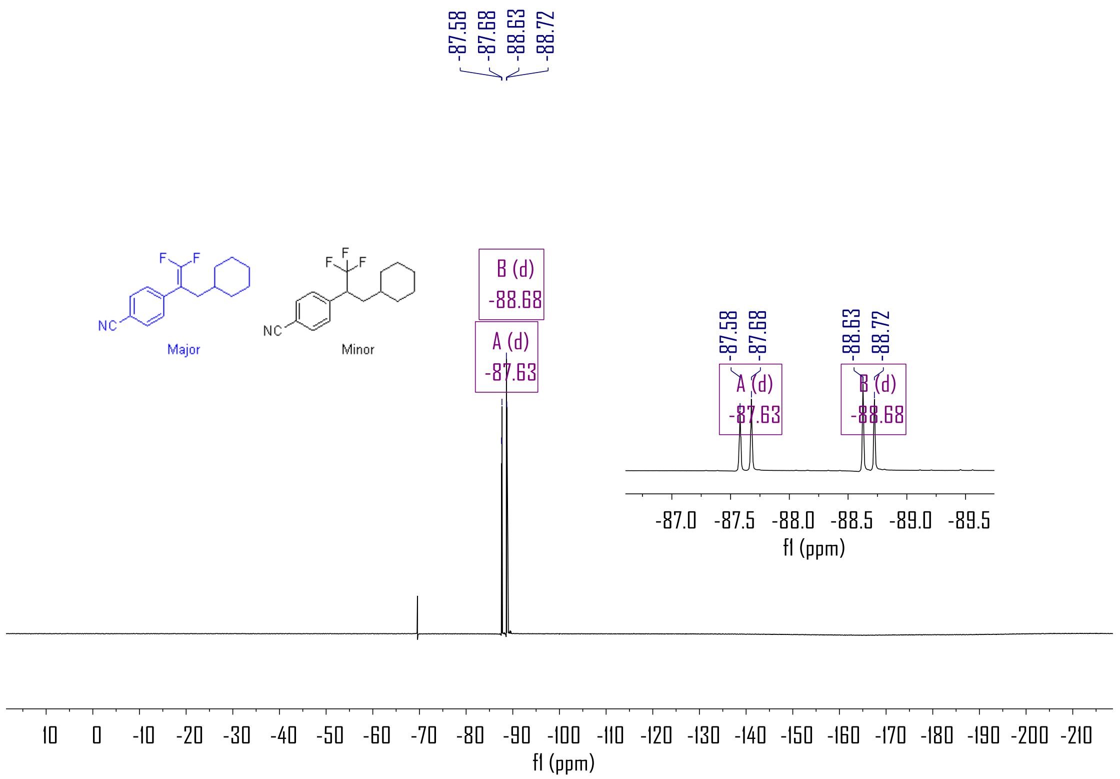

^{19}F NMR spectra for **3ka**.

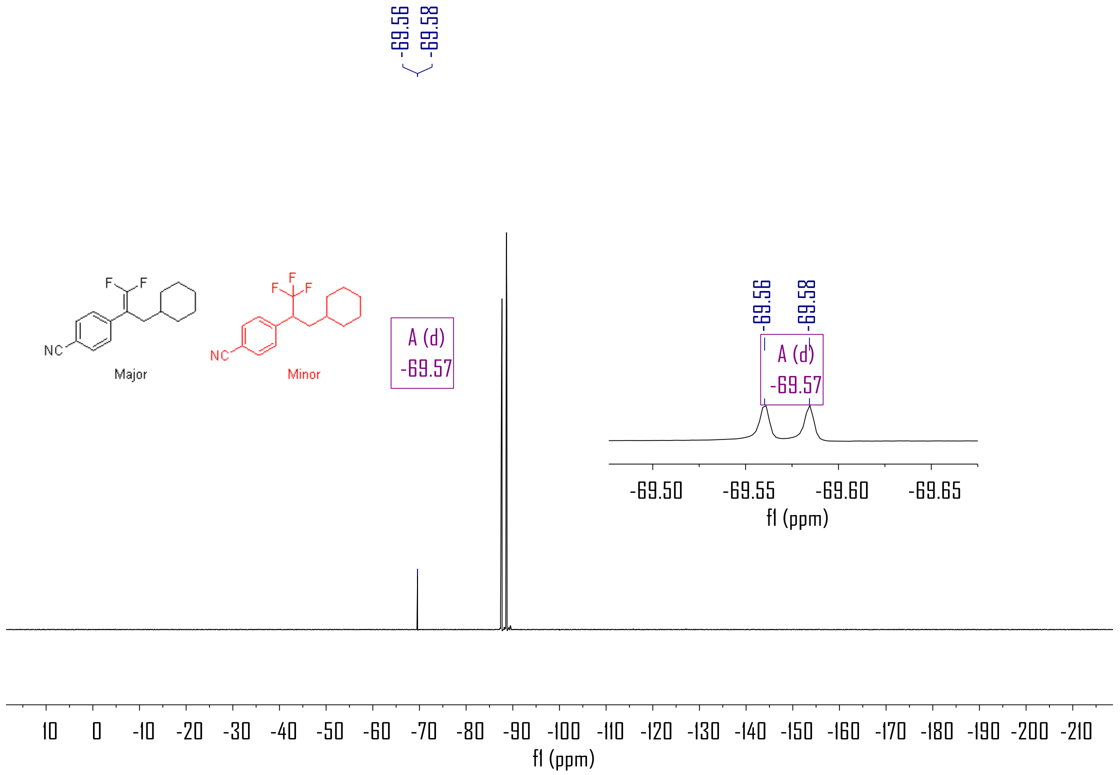

^{19}F NMR spectra for **3ka'**.

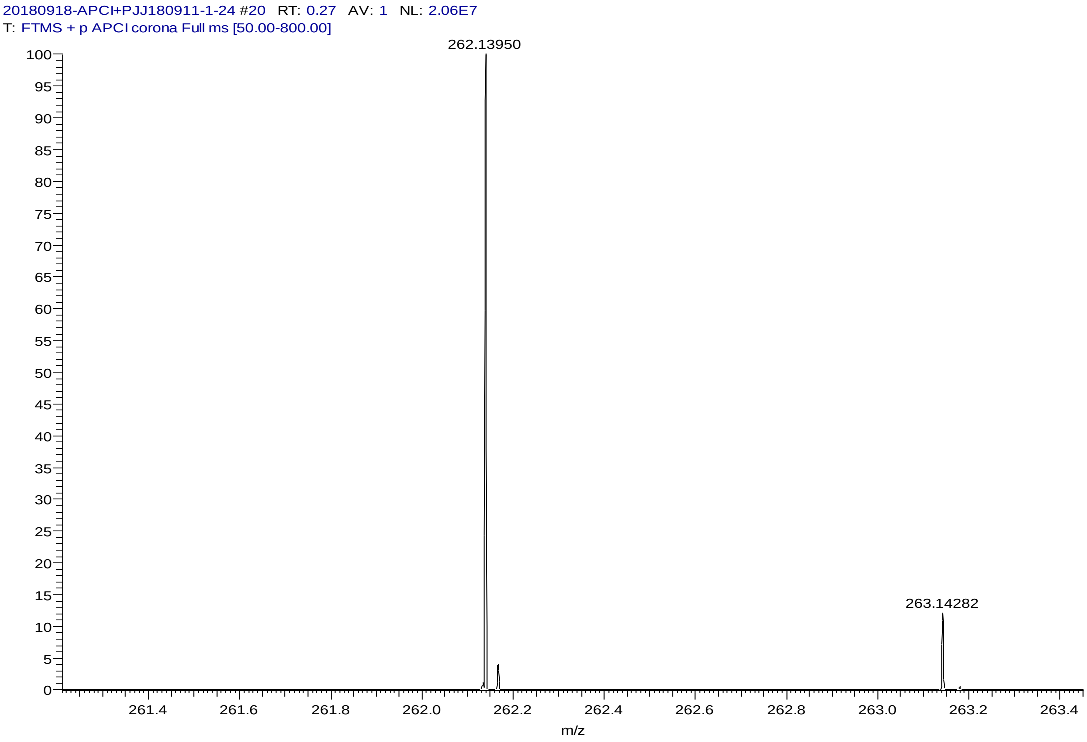

HRMS spectra for **3ka**.

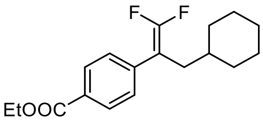

ethyl 4-(3-cyclohexyl-1,1-difluoroprop-1-en-2-yl)benzoate (**3la**)

Following general procedure, **1l** and **2a** were used. The product was isolated by column chromatography as colorless oil (36.9 mg, 0.120 mmol, 60%)

Selectivity (desired C-F cleavage product : addition by-product) > 50:1.

R_f (petroleum ether : ethyl acetate = 20:1) = 0.78.

^{1}H NMR (400 MHz, Chloroform-*d*) δ 8.02 (d, *J* = 8.5 Hz, 2H), 7.43 – 7.35 (m, 2H), 4.38 (q, *J* = 7.1 Hz, 2H), 2.30 (ddd, *J* = 7.2, 2.9, 2.0 Hz, 2H), 1.68 – 1.56 (m, 5H), 1.39 (t, *J* = 7.1 Hz, 3H), 1.28 – 1.17 (m, 1H), 1.15 – 1.05 (m, 3H), 0.98 – 0.85 (m, 2H).

^{13}C NMR (101 MHz, Chloroform-*d*) δ 166.37, 154.28 (dd, *J* = 292.3, 287.5 Hz), 139.01 (dd, *J* = 4.9, 3.5 Hz), 129.71, 129.25, 128.26 (dd, *J* = 3.4, 3.4 Hz), 91.00 (dd, *J* = 22.8, 11.8 Hz), 61.07, 35.93 (dd, *J* = 2.3, 2.3 Hz), 34.99, 32.93, 26.44, 26.13, 14.43.

^{19}F NMR (376 MHz, Chloroform-*d*) δ -89.19 (d, *J* = 39.4 Hz), -89.81 (d, *J* = 39.4 Hz).

HRMS (APCI) calcd for $C_{18}H_{23}O_2F_2^+$ [$(M+H)^+$] 309.16606, found 309.16513.

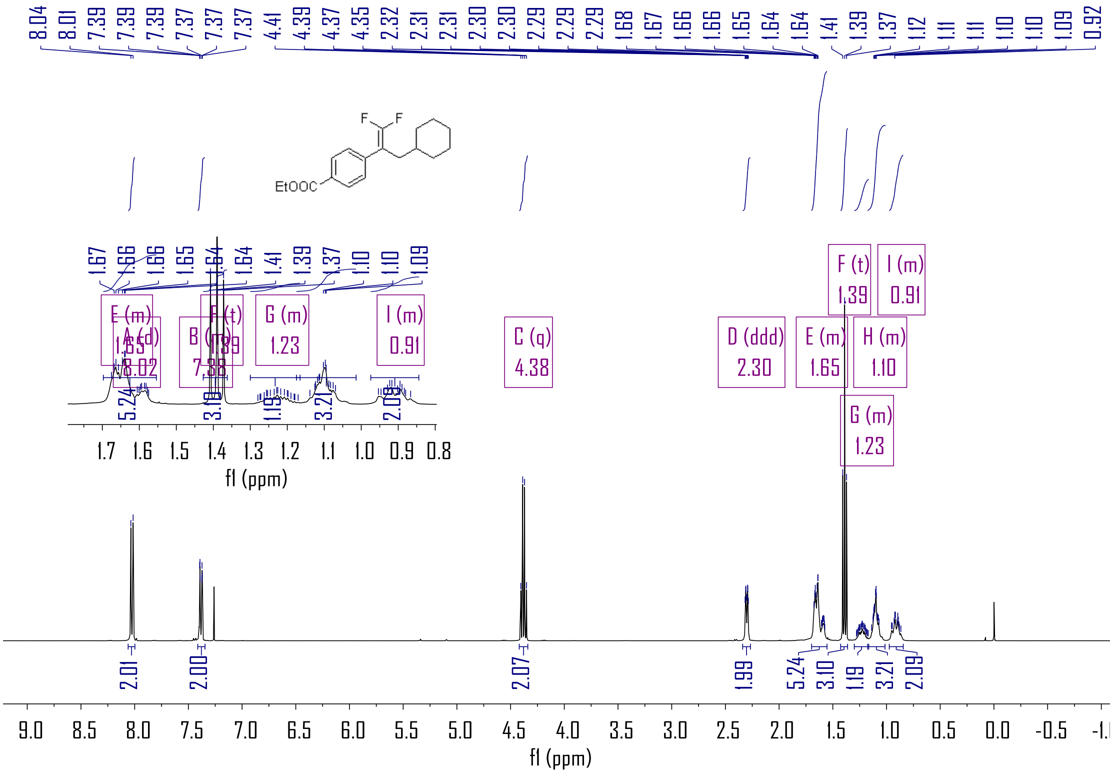

^{1}H NMR spectra for **3la**.

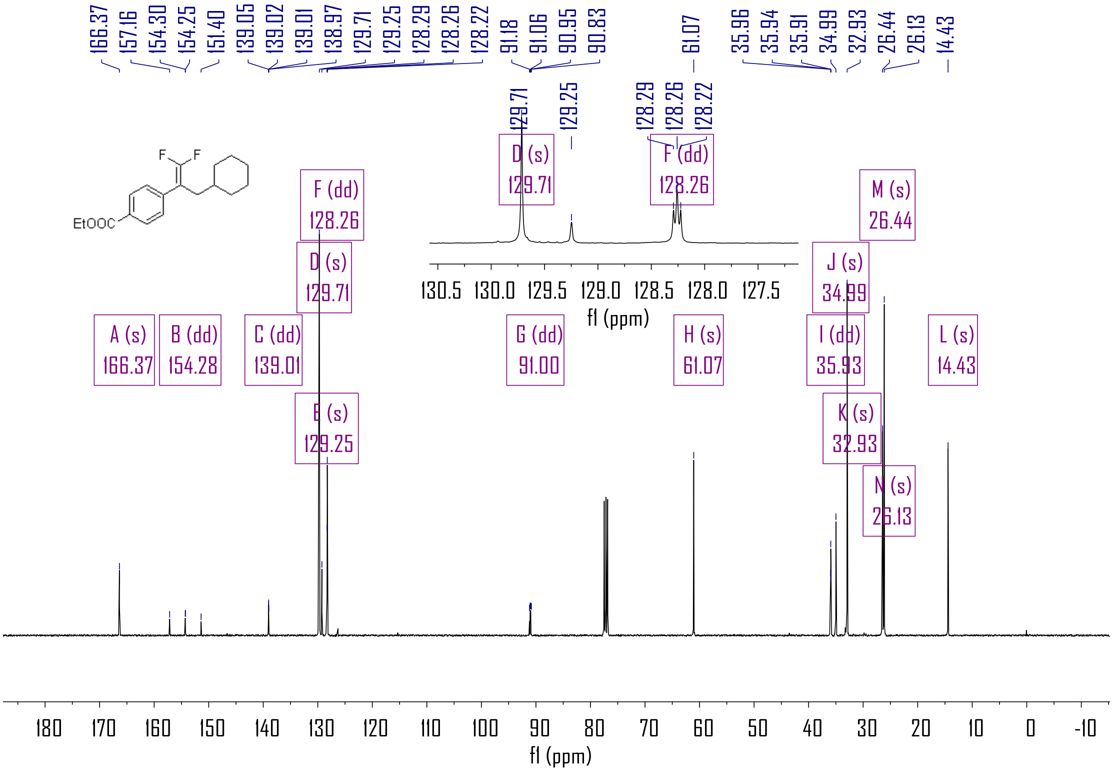

^{13}C NMR spectra for **3la**.

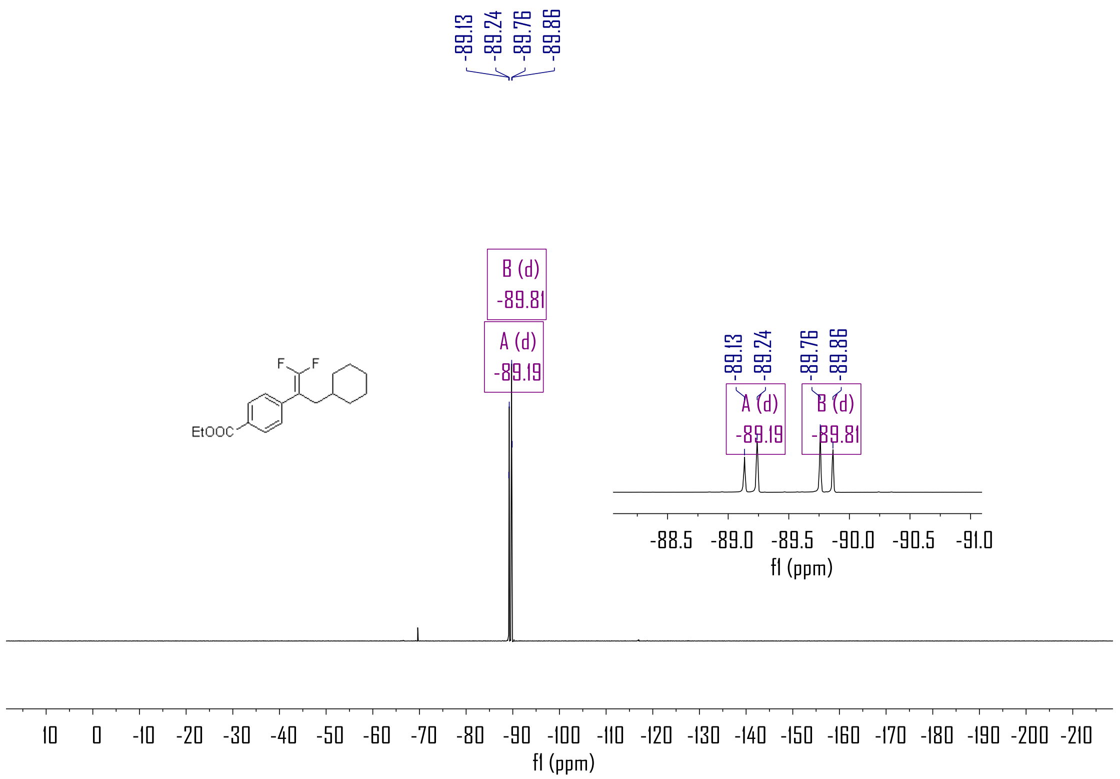

^{19}F NMR spectra for **3la**.

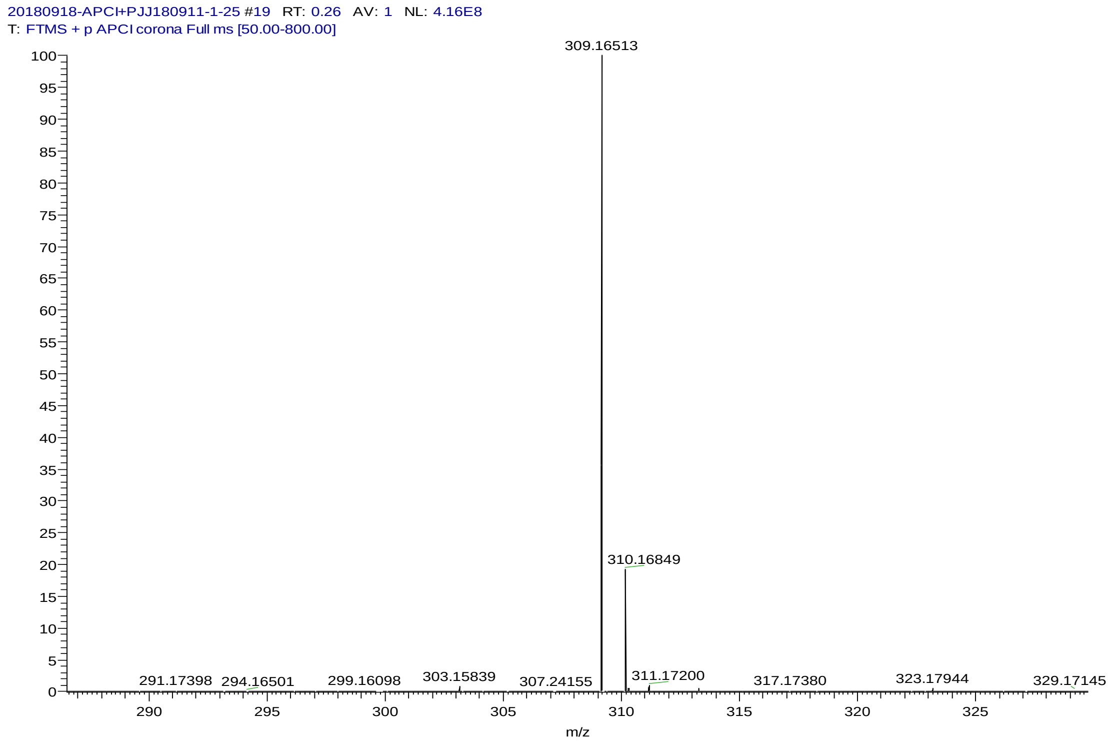

HRMS spectra for **3la**.

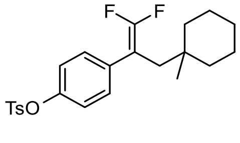

4-(1,1-difluoro-3-(1-methylcyclohexyl)prop-1-en-2-yl)phenyl 4-methylbenzenesulfonate (**3mb**)

Following general procedure, **1m** and **2b** were used. The product was isolated by column chromatography as colorless oil (68.1 mg, 0.162 mmol, 81%).

Selectivity (desired C-F cleavage product : addition by-product) > 50:1.

R_f (petroleum ether : ethyl acetate = 5:1) = 0.75.

^{1}H NMR (400 MHz, Chloroform-*d*) δ 7.66 (d, *J* = 8.3 Hz, 2H), 7.27 (d, *J* = 7.3 Hz, 2H), 7.22 (dd, *J* = 8.7, 1.5 Hz, 2H), 6.94 (d, *J* = 8.7 Hz, 2H), 2.43 (s, 3H), 2.33 – 2.26 (m, 2H), 1.48 – 1.02 (m, 10H), 0.71 (s, 3H).

^{13}C NMR (101 MHz, Chloroform-*d*) δ 154.40 (dd, *J* = 290.4, 288.1 Hz), 148.39, 145.52, 134.98 (dd, *J* = 5.0, 2.7 Hz), 132.20, 129.76, 129.73 (dd, *J* = 3.0, 3.0 Hz), 128.64, 122.33, 89.82 (dd, *J* = 22.5, 13.0 Hz), 40.17, 38.00, 35.26 (dd, *J* = 2.5, 2.5 Hz), 26.30, 24.81, 22.03, 21.79.

^{19}F NMR (376 MHz, Chloroform-*d*) δ -88.66 (d, *J* = 39.3 Hz), -91.35 (d, *J* = 39.2 Hz).

HRMS (APCI) calcd for $C_{23}H_{27}O_3F_2S^+$ [(M+H)$^+$] 421.16435, found 421.16315.

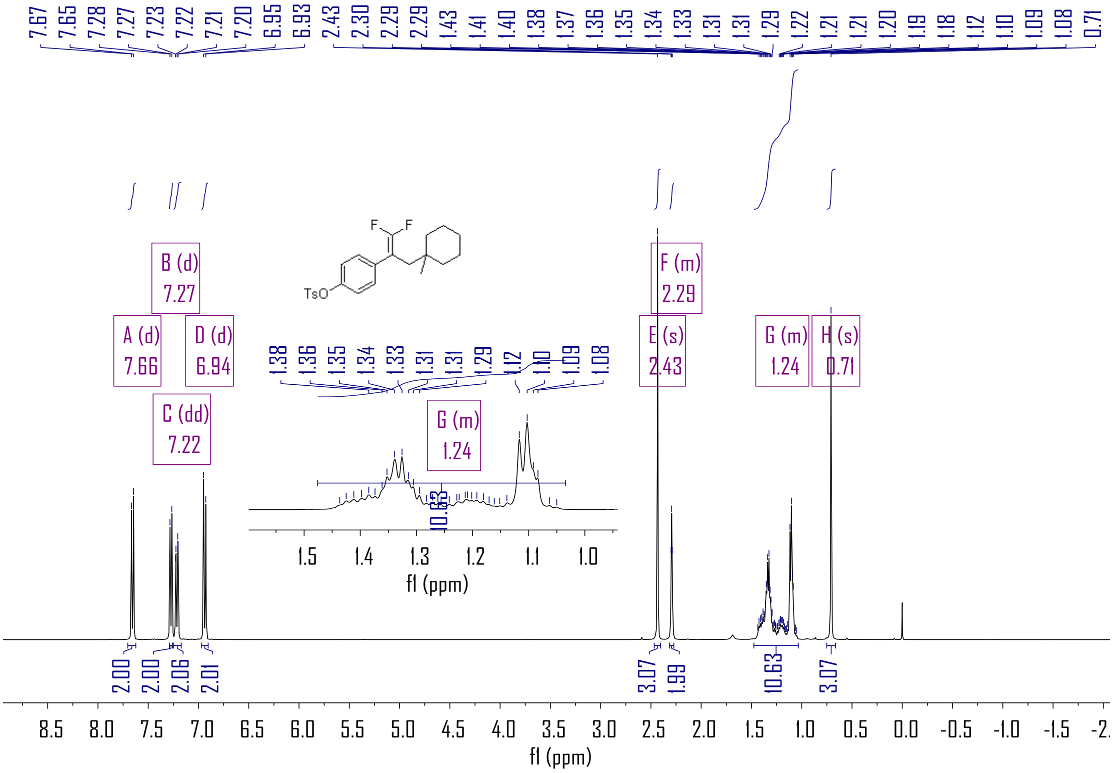

^{1}H NMR spectra for **3mb**.

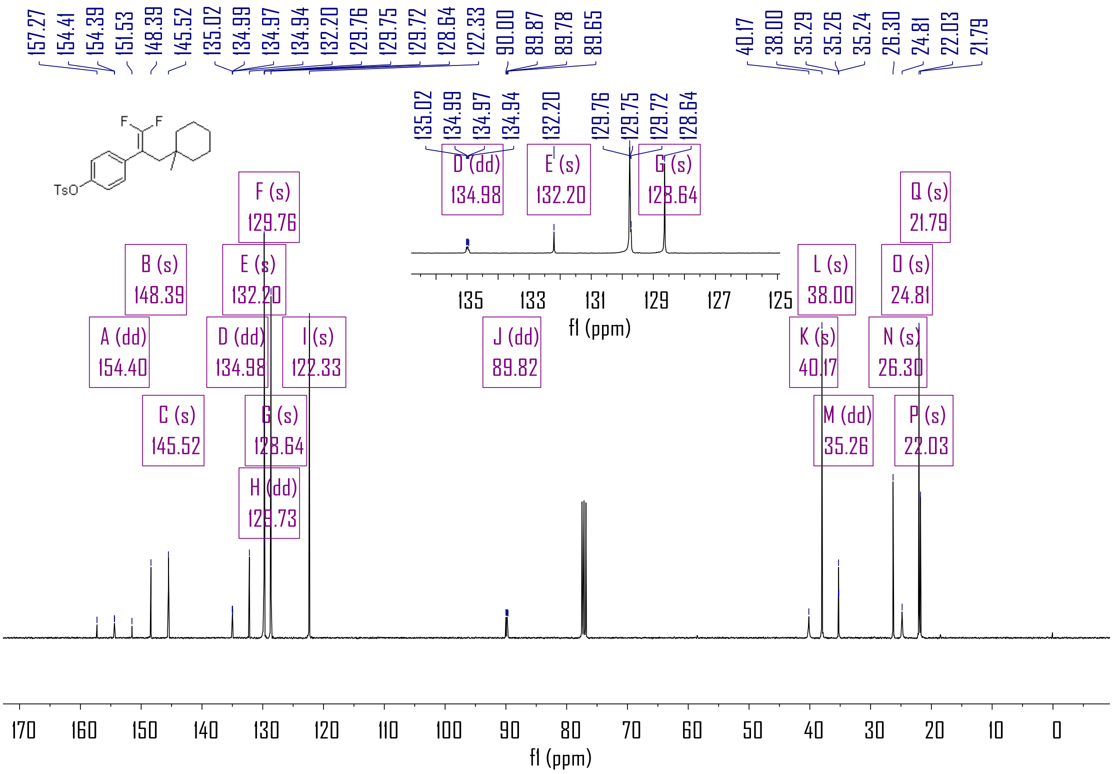

^{13}C NMR spectra for **3mb**.

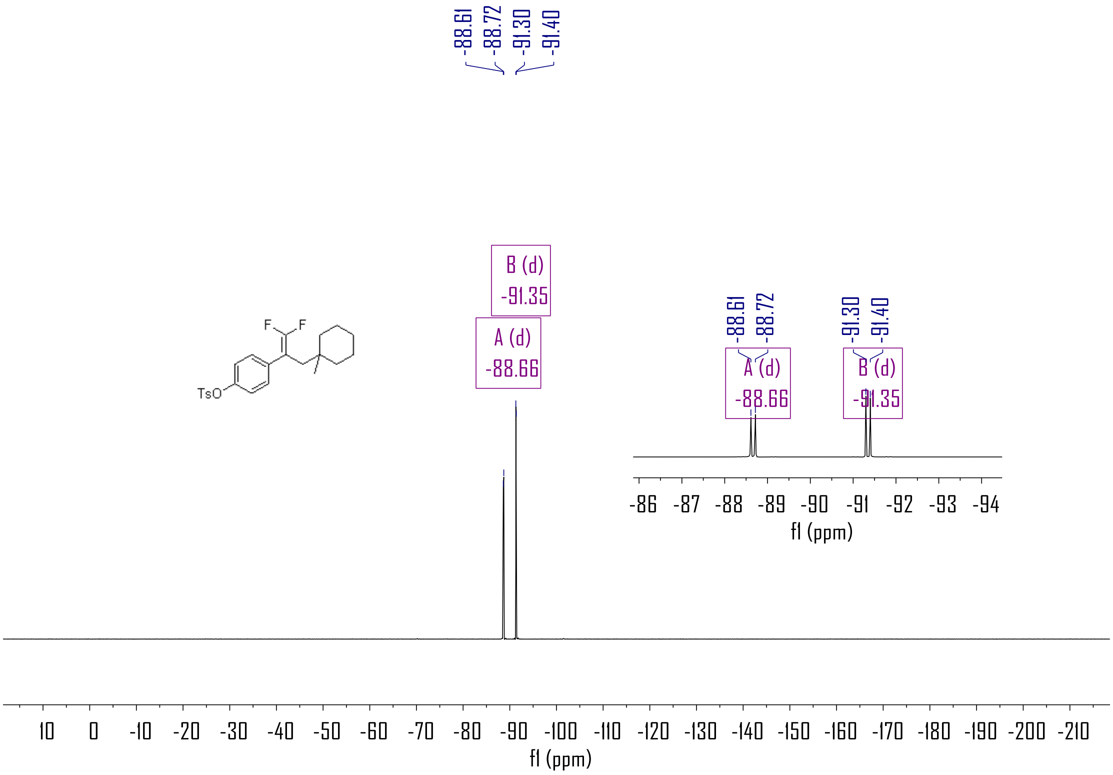

^{19}F NMR spectra for **3mb**.

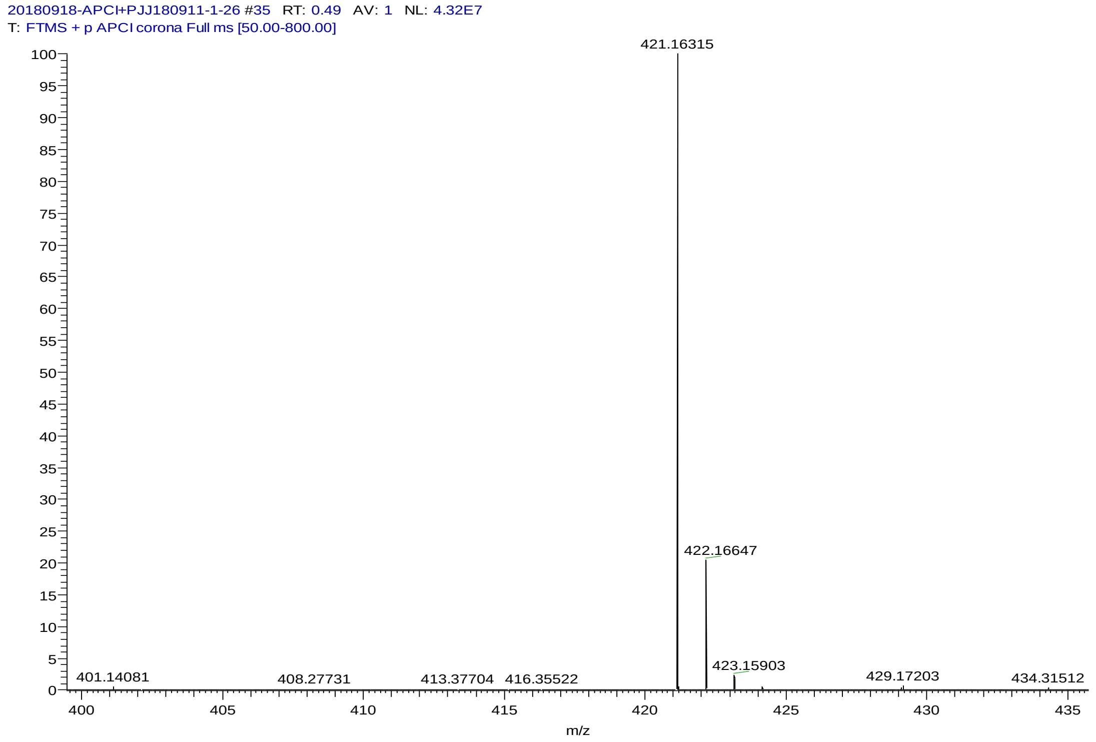

HRMS spectra for **3mb**.

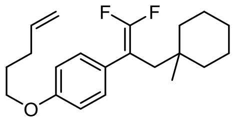

1-(1,1-difluoro-3-(1-methylcyclohexyl)prop-1-en-2-yl)-4-(pent-4-en-1-yloxy)benzene (**3nb**)

Following general procedure, **1n** and **2b** were used. The product was isolated by column chromatography as colorless oil (60.9 mg, 0.182 mmol, 91%).

Selectivity (desired C-F cleavage product : addition by-product) > 50:1.

R_f (petroleum ether : ethyl acetate = 100:1) = 0.75.

^{1}H NMR (400 MHz, Chloroform-*d*) δ 7.22 (dd, *J* = 8.8, 1.6 Hz, 2H), 6.87 (d, *J* = 8.8 Hz, 2H), 5.87 (ddt, *J* = 16.9, 10.2, 6.6 Hz, 1H), 5.08 (dq, *J* = 17.1, 1.7 Hz, 1H), 5.05 – 4.98 (m, 1H), 3.97 (t, *J* = 6.5 Hz, 2H), 2.35 – 2.31 (m, 2H), 2.30 – 2.20 (m, 2H), 1.90 (p, *J* = 6.6 Hz, 2H), 1.45 – 1.13 (m, 10H), 0.77 (s, 3H).

^{13}C NMR (101 MHz, Chloroform-*d*) δ 158.05, 154.39 (dd, *J* = 288.4, 286.7 Hz), 137.94, 129.62 (dd, *J* = 2.8, 2.8 Hz), 127.99 (dd, *J* = 4.7, 2.6 Hz), 115.30, 114.34, 90.16 (dd, *J* = 21.5, 13.3 Hz), 67.25, 40.44, 38.15, 35.25 (dd, *J* = 2.5, 2.5 Hz), 30.27, 28.60, 26.45, 24.81, 22.15.

^{19}F NMR (376 MHz, Chloroform-*d*) δ -90.47 (d, *J* = 43.7 Hz), -93.10 (d, *J* = 43.8 Hz).

HRMS (APCI) calcd for $C_{21}H_{29}OF_2^+$ [$(M+H)^+$] 335.21810, found 335.21722.

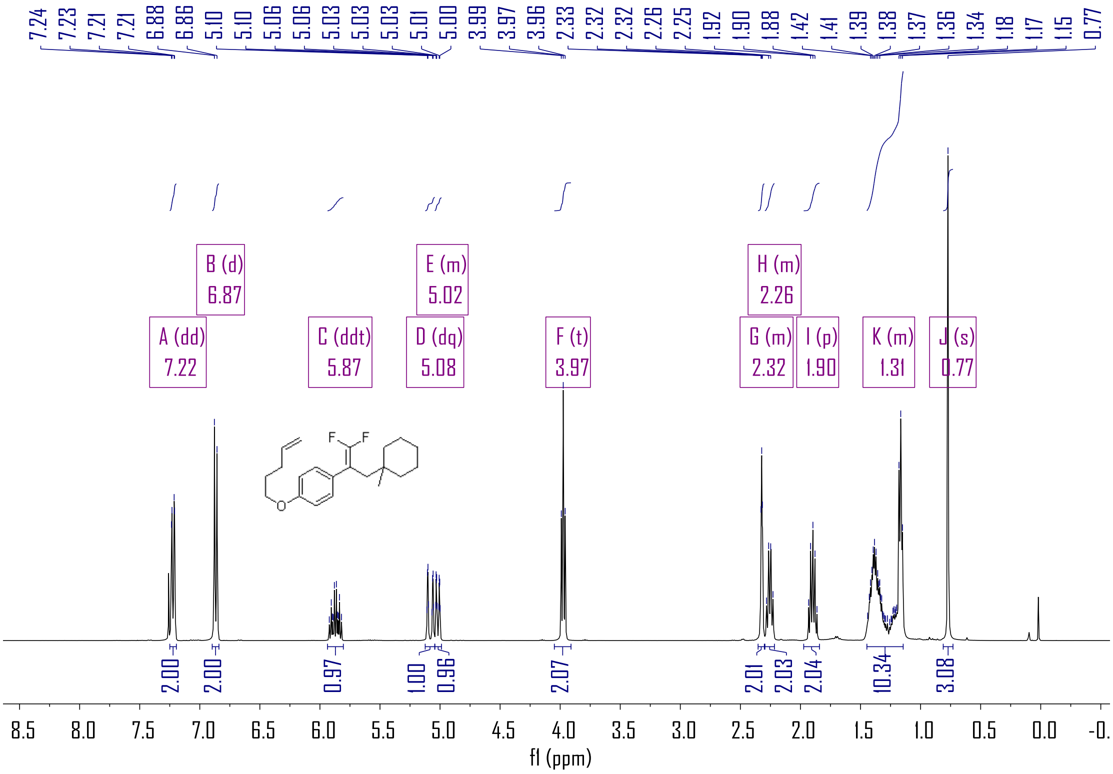

^{1}H NMR spectra for **3nb**.

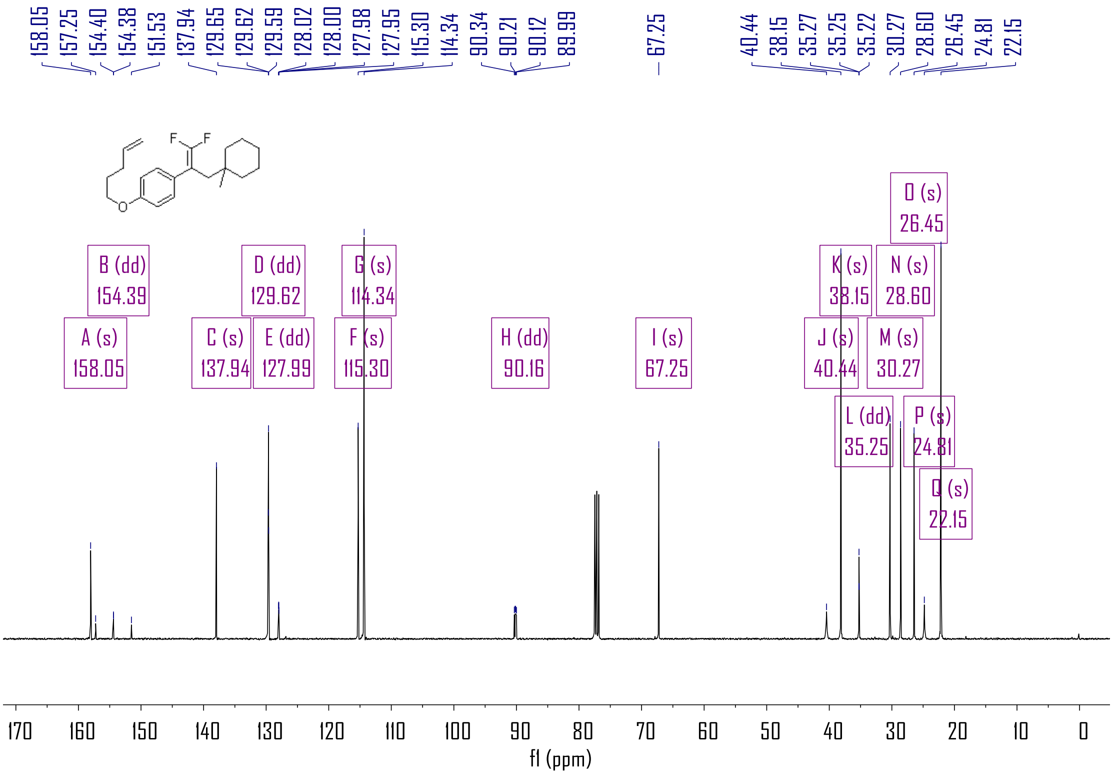

^{13}C NMR spectra for **3nb**.

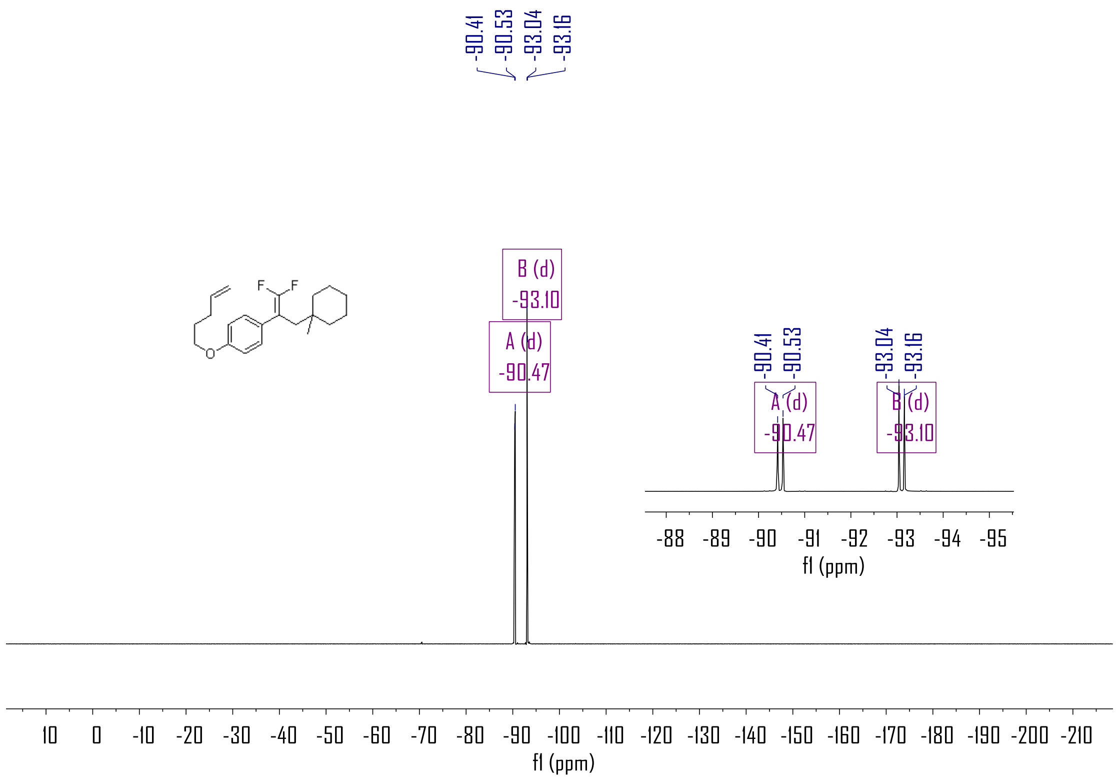

^{19}F NMR spectra for **3nb**.

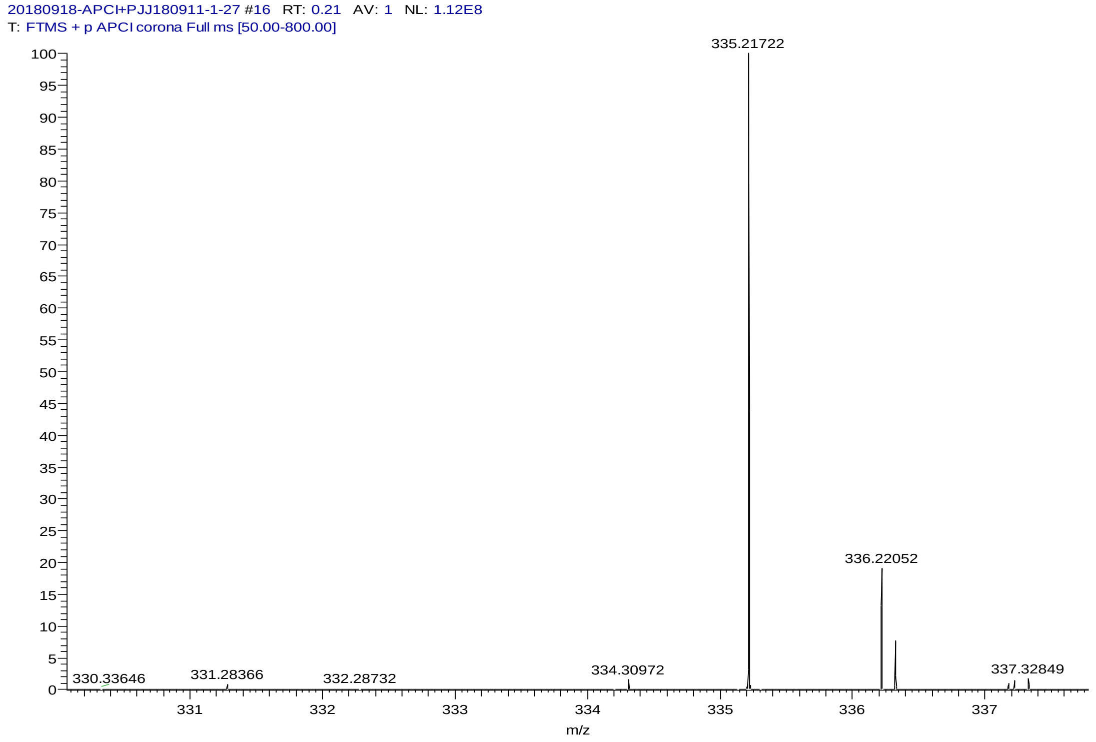

HRMS spectra for **3nb**.

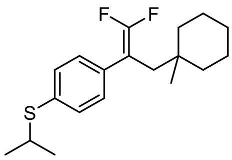

(4-(1,1-difluoro-3-(1-methylcyclohexyl)prop-1-en-2-yl)phenyl)(isopropyl)sulfane (**3ob**)

Following general procedure, **1o** and **2b** were used. The product was isolated by column chromatography as colorless oil (63.6 mg, 0.196 mmol, 98%).

Selectivity (desired C-F cleavage product : addition by-product) > 50:1.

R_f (petroleum ether) = 0.75.

^{1}H NMR (400 MHz, Chloroform-*d*) δ 7.39 – 7.32 (m, 2H), 7.25 – 7.21 (m, 2H), 3.38 (hept, *J* = 6.6 Hz, 1H), 2.37 – 2.29 (m, 2H), 1.48 – 1.10 (m, 16H), 0.75 (s, 3H).

^{13}C NMR (101 MHz, Chloroform-*d*) δ 154.43 (dd, *J* = 290.1, 287.8 Hz), 134.38 (dd, *J* = 4.8, 2.7 Hz), 134.26, 131.59, 128.94 (dd, *J* = 2.8, 2.8 Hz), 90.27 (dd, *J* = 21.9, 12.9 Hz), 40.17, 38.26, 38.09, 35.30 (dd, *J* = 2.3, 2.3 Hz), 26.39, 24.82, 23.21, 22.09.

^{19}F NMR (376 MHz, Chloroform-*d*) δ -89.03 (d, *J* = 40.7 Hz), -91.70 (d, *J* = 40.5 Hz).

HRMS (APCI) calcd for $C_{19}H_{27}F_2S^+$ [$(M+H)^+$] 325.17960, found 325.17914.

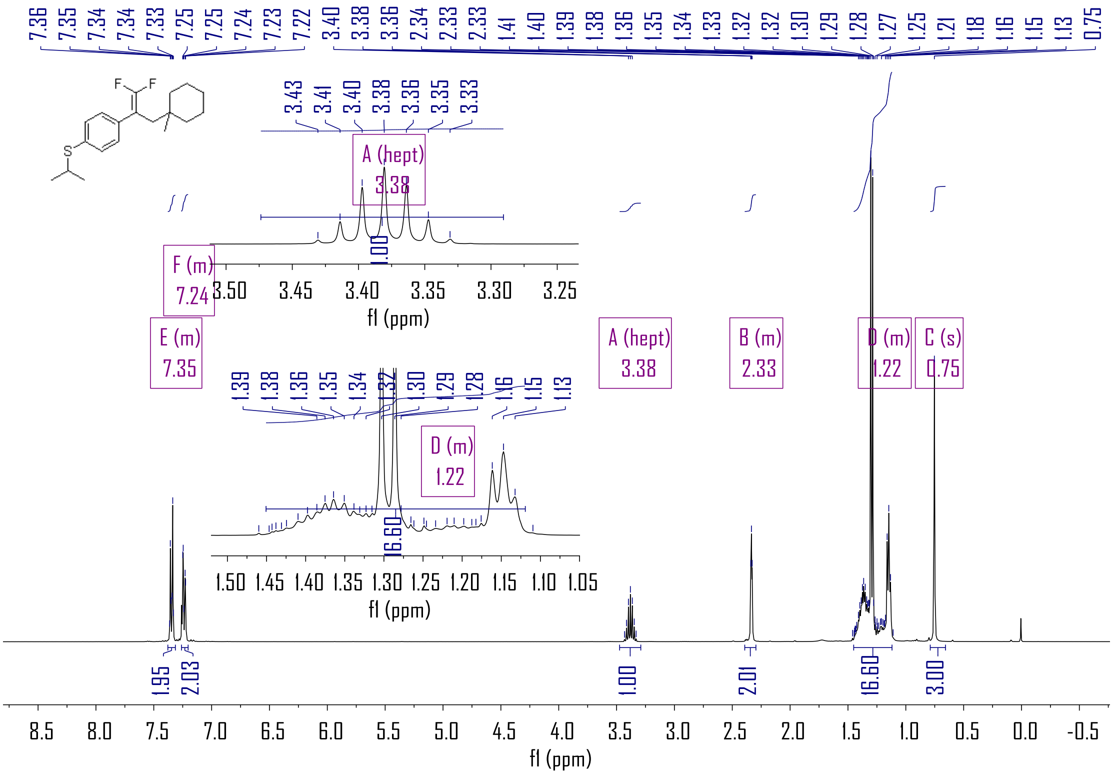

^{1}H NMR spectra for **3ob**.

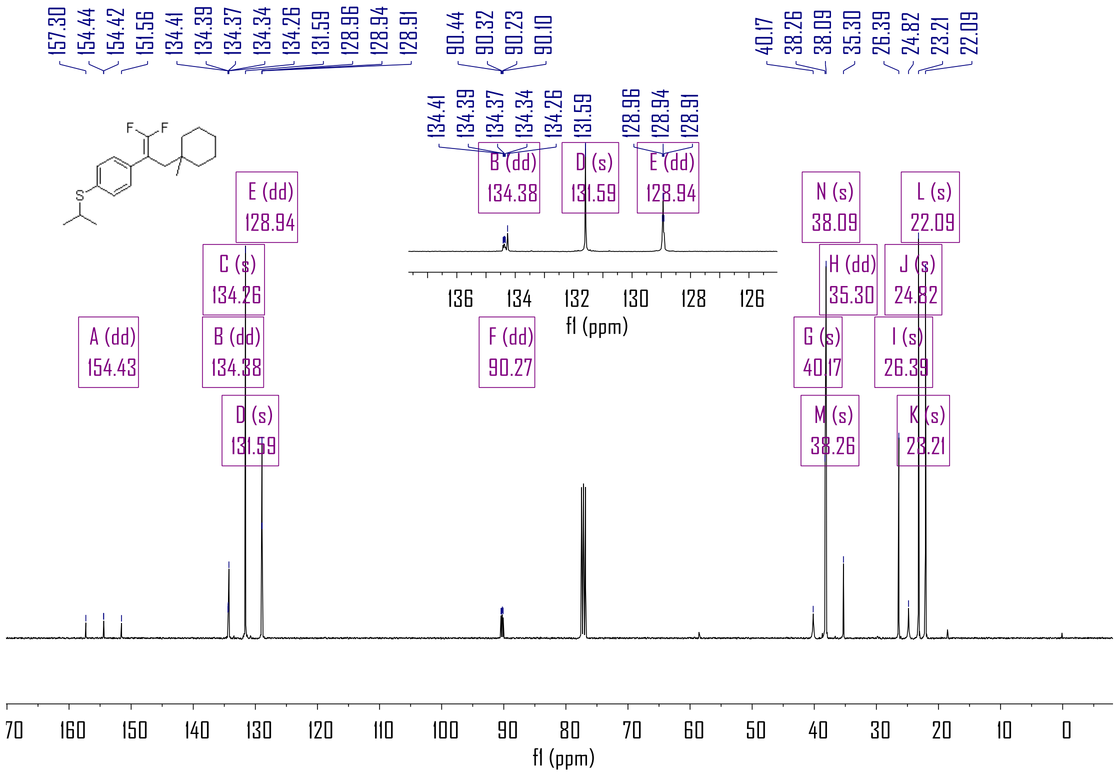

^{13}C NMR spectra for **3ob**.

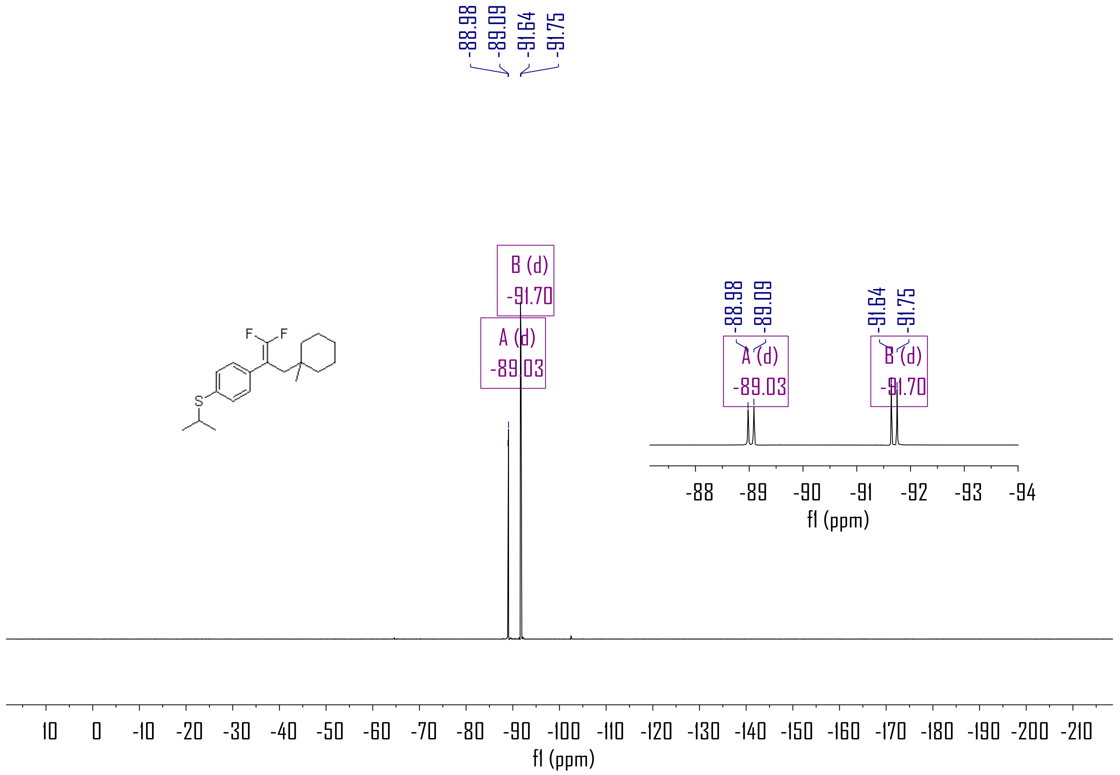

^{19}F NMR spectra for **3ob**.

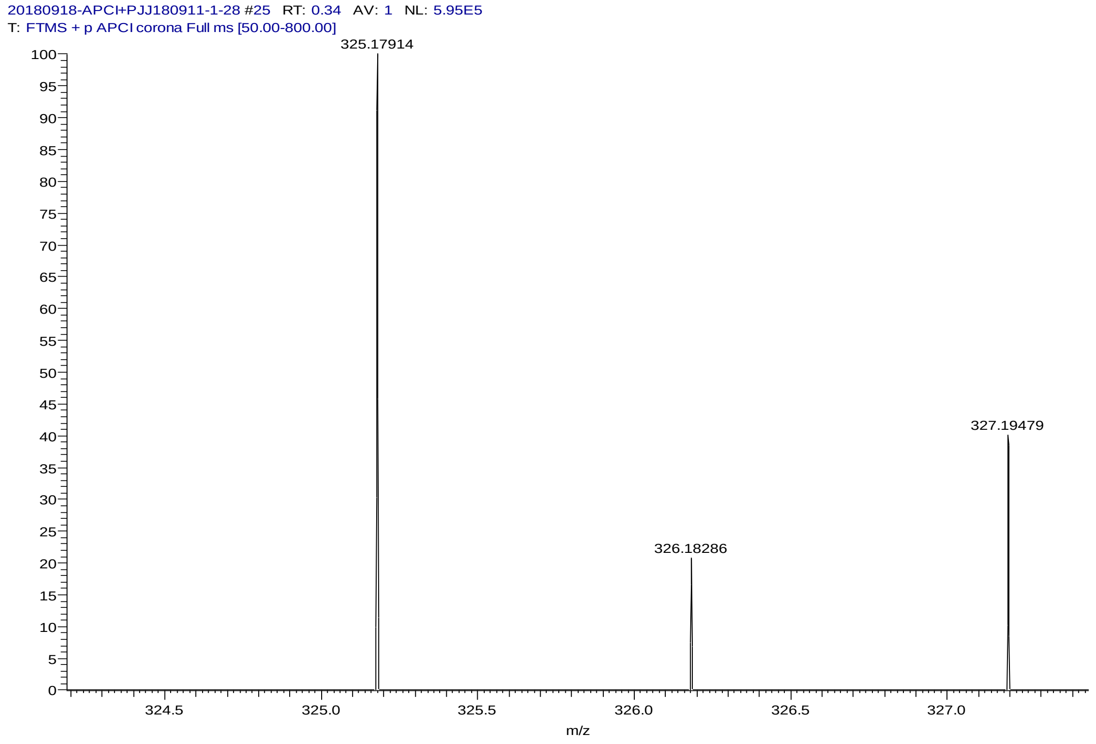

HRMS spectra for **3ob**.

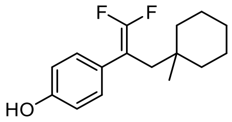

4-(1,1-difluoro-3-(1-methylcyclohexyl)prop-1-en-2-yl)phenol (**3pb**)

Following general procedure, **1p** and **2b** were used. The product was isolated by column chromatography as colorless oil (33.6 mg, 0.126 mmol, 63%).

Selectivity (desired C-F cleavage product : addition by-product) > 50:1.

R_f (petroleum ether : ethyl acetate = 5:1) = 0.63.

^{1}H NMR (400 MHz, Chloroform-*d*) δ 7.18 (dd, *J* = 8.6, 1.5 Hz, 2H), 6.80 (d, *J* = 8.6 Hz, 2H), 2.35 – 2.27 (m, 2H), 1.48 – 1.08 (m, 10H), 0.76 (s, 3H).

^{13}C NMR (101 MHz, Chloroform-*d*) δ 154.49, 154.38 (dd, *J* = 288.4, 287.1 Hz), 129.89 (dd, *J* = 2.8, 2.8 Hz), 128.35 (dd, *J* = 4.8, 2.5 Hz), 115.32, 90.11 (dd, *J* = 21.7, 13.3 Hz), 40.44, 38.13, 35.25 (dd, *J* = 2.4, 2.4 Hz), 26.43, 24.82, 22.13.

^{19}F NMR (376 MHz, Chloroform-*d*) δ -90.41 (d, *J* = 43.6 Hz), -93.00 (dt, *J* = 43.6, 1.5 Hz).

HRMS (ESI) calcd for $C_{16}H_{21}OF_2^+$ [$(M+H)^+$] 267.1555, found 267.1560.

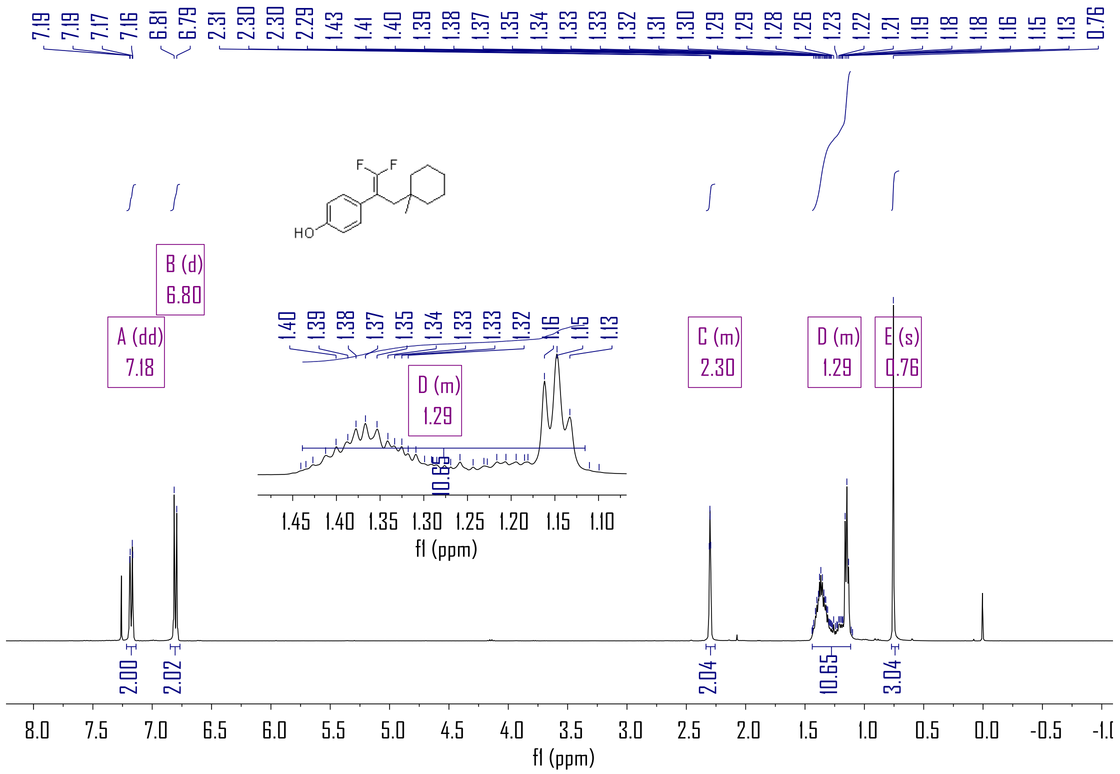

^{1}H NMR spectra for **3pb**.

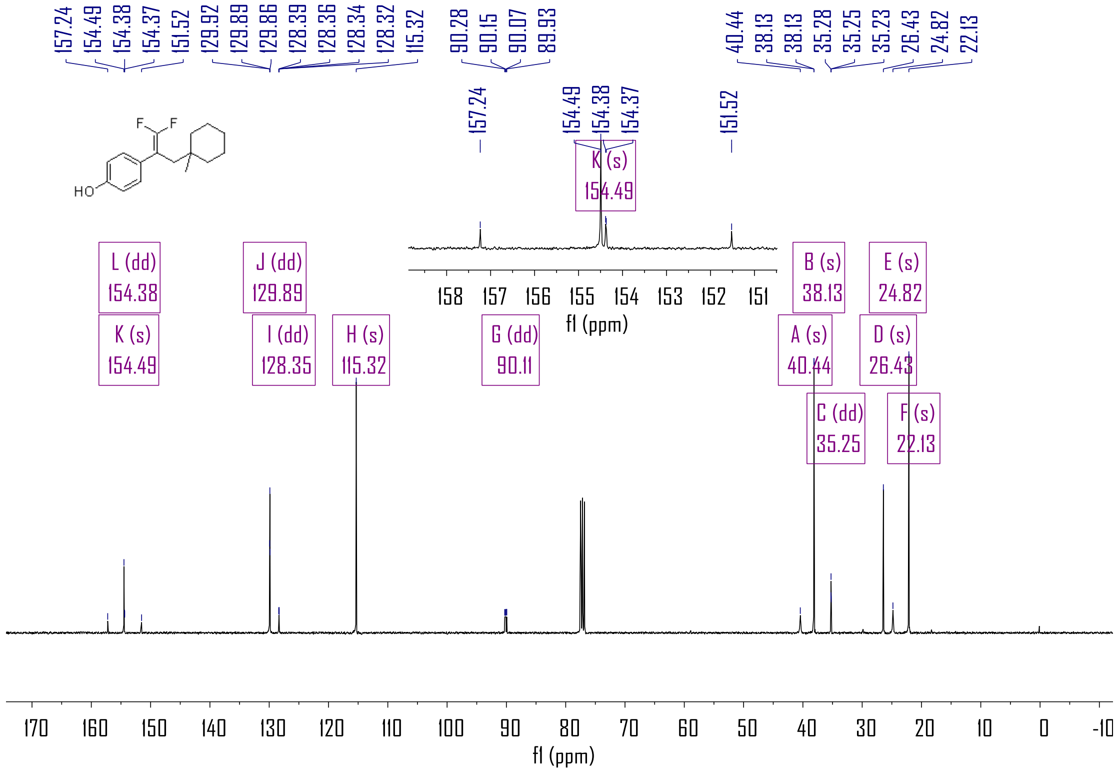

^{13}C NMR spectra for **3pb**.

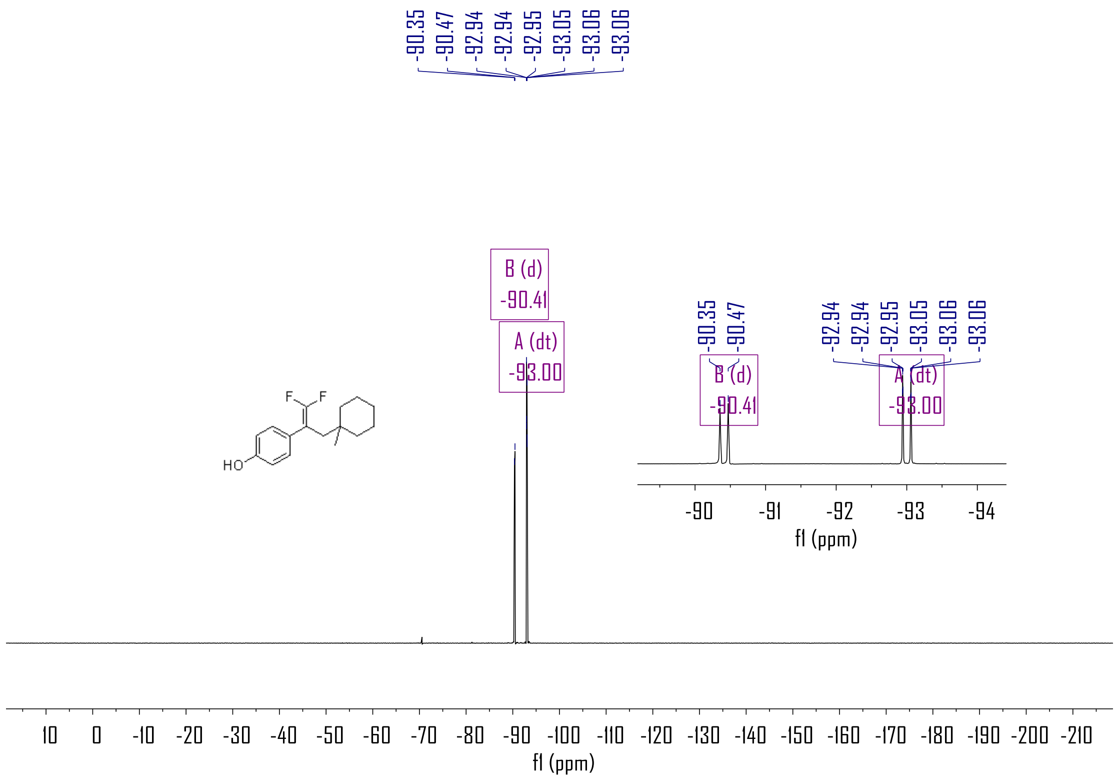

^{19}F NMR spectra for **3pb**.

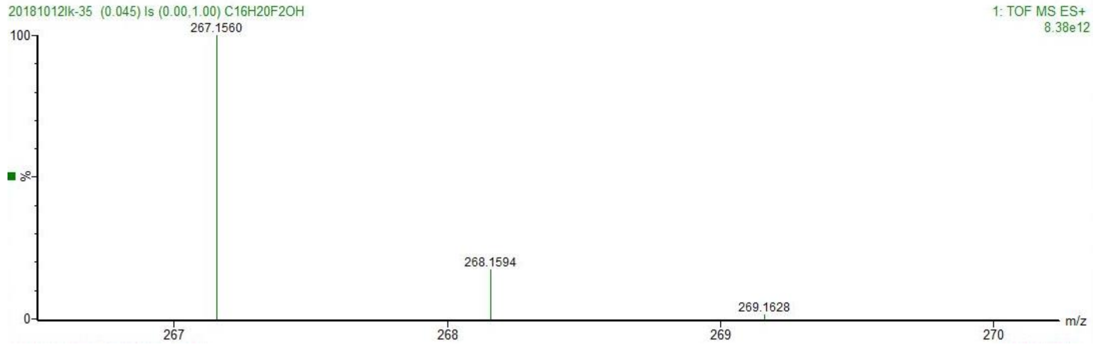

HRMS spectra for **3pb**.

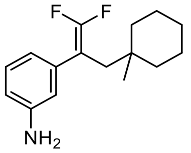

3-(1,1-difluoro-3-(1-methylcyclohexyl)prop-1-en-2-yl)aniline (**3qb**)

Following general procedure, **1q** and **2b** were used. The product was isolated by column chromatography as colorless oil (41.9 mg, 0.158 mmol, 79%).

Selectivity (desired C-F cleavage product : addition by-product) > 50:1.

R_f (petroleum ether : ethyl acetate = 20:1) = 0.33.

^{1}H NMR (400 MHz, Chloroform-*d*) δ 7.12 (t, *J* = 7.8 Hz, 1H), 6.78 – 6.69 (m, 1H), 6.66 (q, *J* = 1.9 Hz, 1H), 6.62 – 6.54 (m, 1H), 3.56 (brs, 2H), 2.31 (dd, *J* = 2.9, 2.0 Hz, 2H), 1.50 – 1.11 (m, 10H), 0.78 (s, 3H).

^{13}C NMR (101 MHz, Chloroform-*d*) δ 154.36 (dd, *J* = 289.7, 286.9 Hz), 146.03, 137.06 (dd, *J* = 4.8, 2.5 Hz), 129.16, 119.26 (dd, *J* = 2.7, 2.7 Hz), 115.57 (dd, *J* = 2.9, 2.9 Hz), 114.11, 90.73 (dd, *J* = 21.5, 13.0 Hz), 40.39, 38.06, 35.28 (dd, *J* = 2.4, 2.4 Hz), 26.43, 24.65, 22.13.

^{19}F NMR (376 MHz, Chloroform-*d*) δ -89.63 (d, *J* = 41.4 Hz), -91.68 (d, *J* = 41.4 Hz).

HRMS (ESI) calcd for $C_{16}H_{22}NF_2^+$ [(M+H)$^+$] 266.17148, found 266.17120.

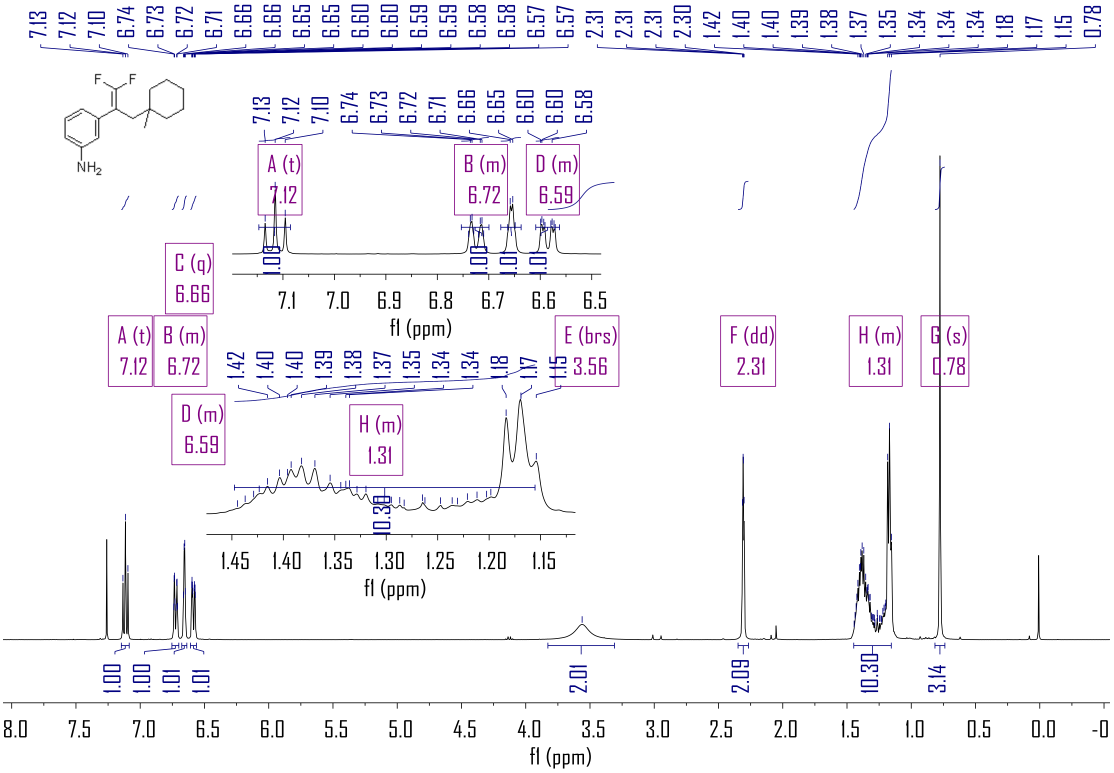

^{1}H NMR spectra for **3qb**.

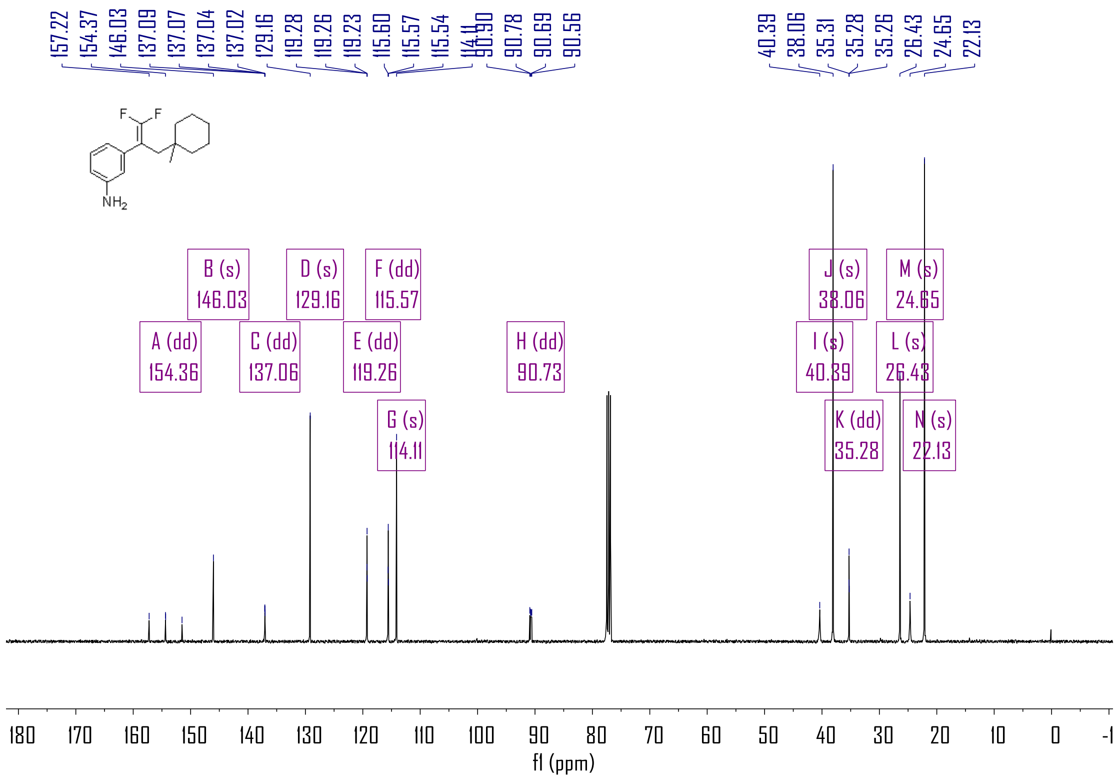

^{13}C NMR spectra for **3qb**.

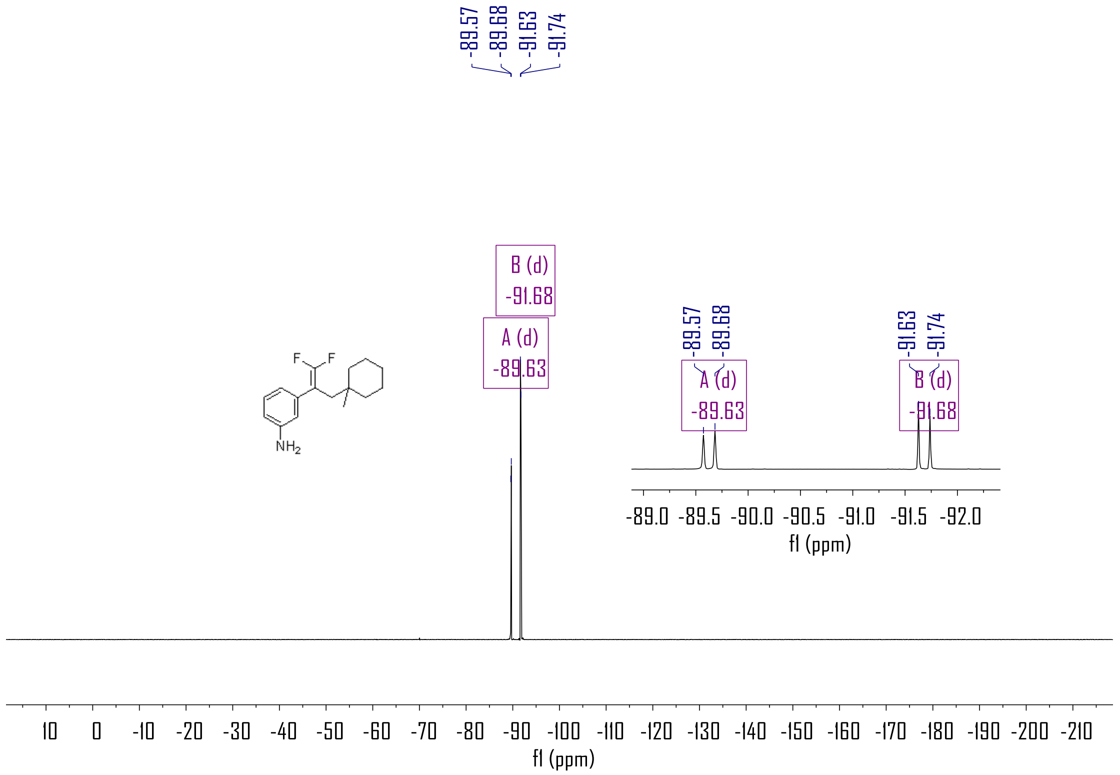

^{19}F NMR spectra for **3qb**.

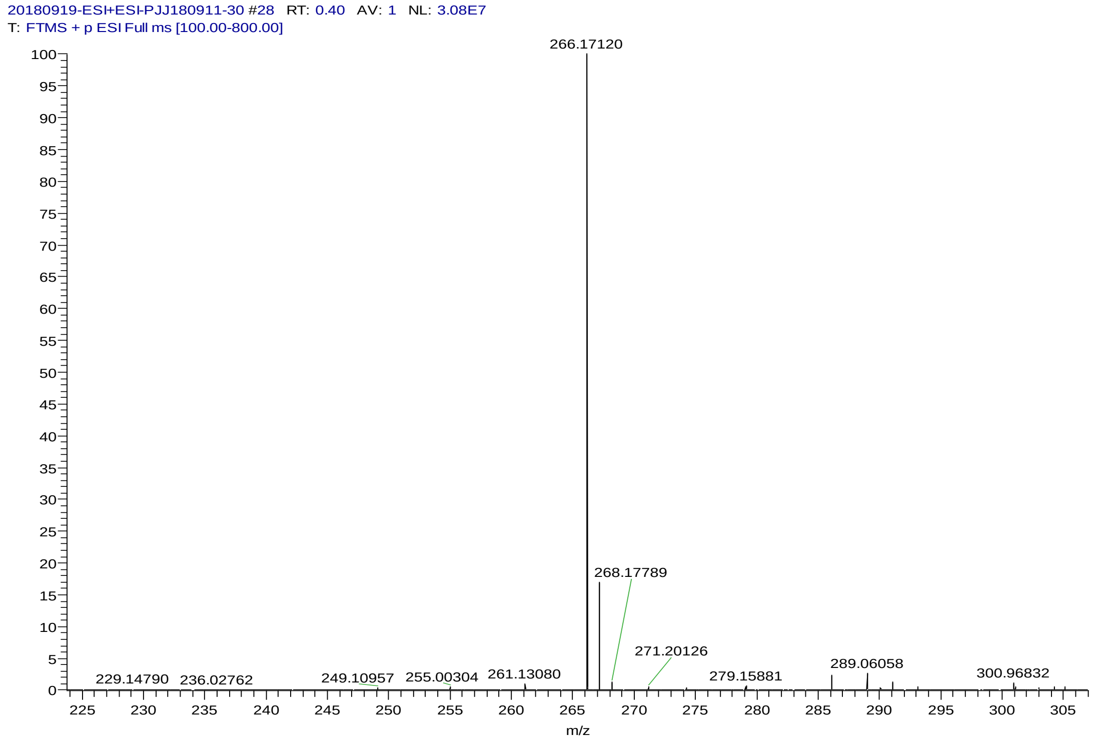

HRMS spectra for **3qb**.

8. Examples Described in Table 4

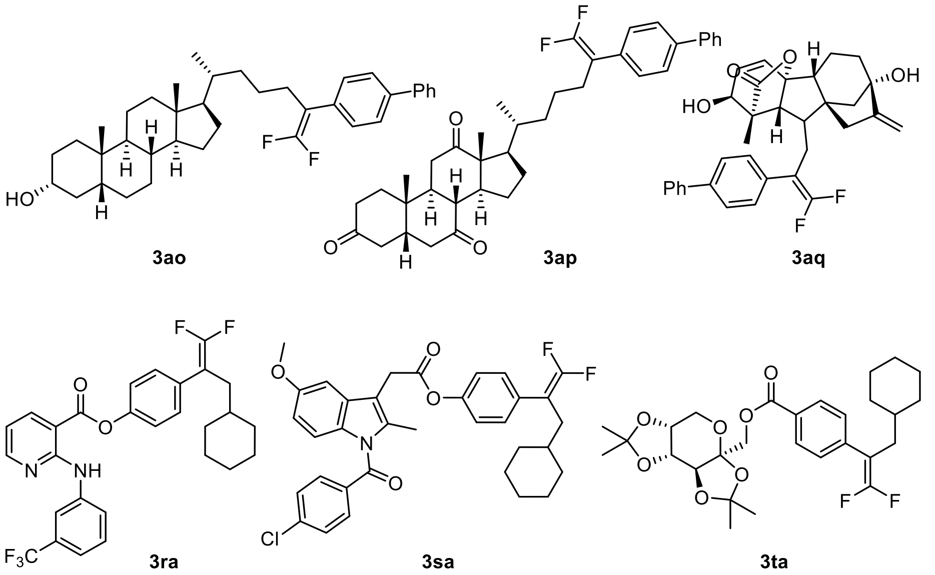

List of substrates in Table 4.

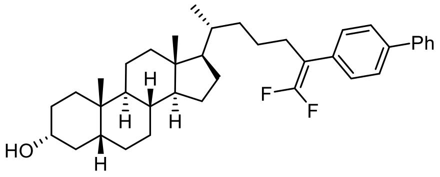

(3*R*,5*R*,8*R*,9*S*,10*S*,13*R*,14*S*,17*R*)-17-((*R*)-6-([1,1'-biphenyl]-4-yl)-7,7-difluorohept-6-en-2-yl)-10,13-dimethylhexadecahydro-1*H*-cyclopenta[*a*]phenanthren-3-ol (**3ao**)

Following general procedure, **1a** and **2o** were used. The product was isolated by column chromatography as colorless oil (82.8 mg, 0.148 mmol, 74%).

Selectivity (desired C-F cleavage product : addition by-product) > 50:1.

R_f (petroleum ether : ethyl acetate = 5:1) = 0.45.

^{1}H NMR (400 MHz, Chloroform-*d*) δ 7.66 – 7.56 (m, 4H), 7.50 – 7.31 (m, 5H), 3.63 (tt, *J* = 11.0, 4.6 Hz, 1H), 2.50 – 2.33 (m, 2H), 1.95 (dt, *J* = 12.3, 3.1 Hz, 1H), 1.91 – 1.74 (m, 5H), 1.71 – 0.95 (m, 22H), 0.92 (s, 3H), 0.87 (d, *J* = 6.5 Hz, 3H), 0.63 (s, 3H).

^{13}C NMR (101 MHz, Chloroform-*d*) δ 153.74 (dd, *J* = 290.5, 286.7 Hz), 140.67, 139.96, 132.91 (dd, *J* = 4.3, 3.0 Hz), 128.88, 128.67 (dd, *J* = 3.4, 3.4 Hz), 127.44, 127.13, 127.08, 92.33 (dd, *J* = 21.5, 12.6 Hz), 71.92, 56.58, 56.31, 42.78, 42.21, 40.55, 40.28, 36.51, 35.94, 35.59, 35.47, 35.37, 34.66, 30.61, 28.35, 27.99, 27.32, 26.55, 24.45 (dd, *J* = 2.5, 2.5 Hz), 24.32, 23.48, 20.93, 18.70, 12.12.

^{19}F NMR (376 MHz, Chloroform-*d*) δ -91.20 (d, *J* = 43.8 Hz), -91.44 (d, *J* = 43.7 Hz).

HRMS (ESI) calcd for $C_{38}H_{51}OF_2^+$ [(M+H)$^+$] 561.3902, found 561.3908.

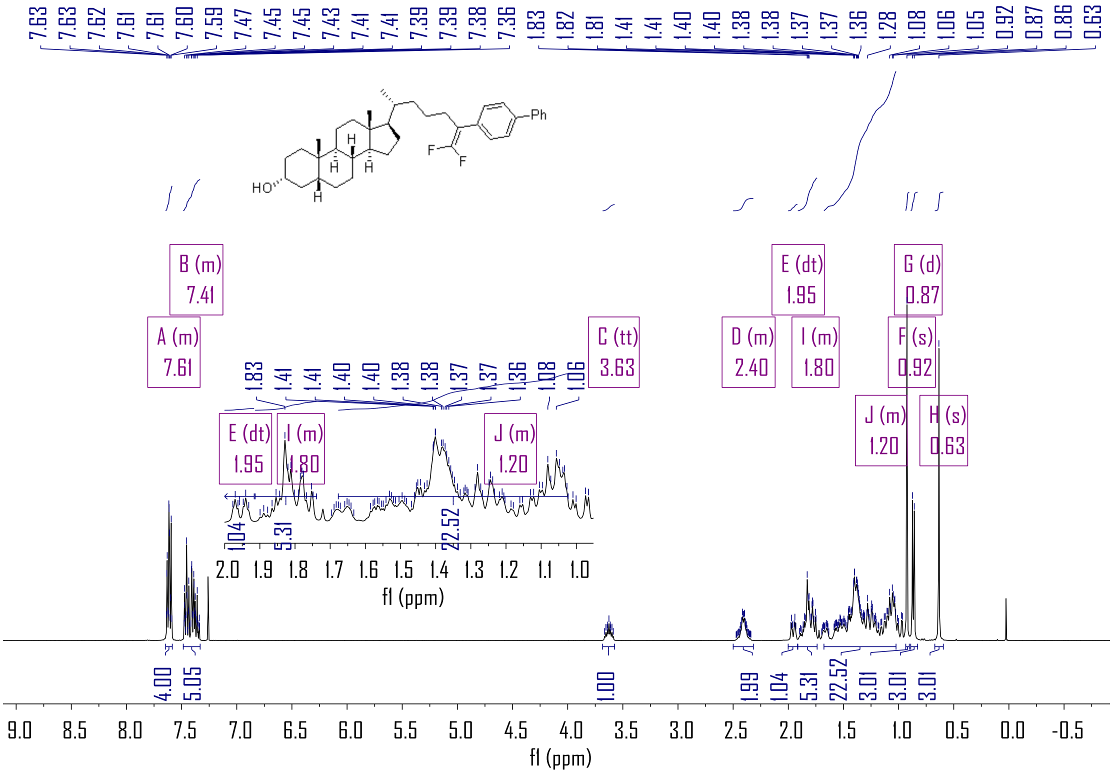

^{1}H NMR spectra for **3ao**.

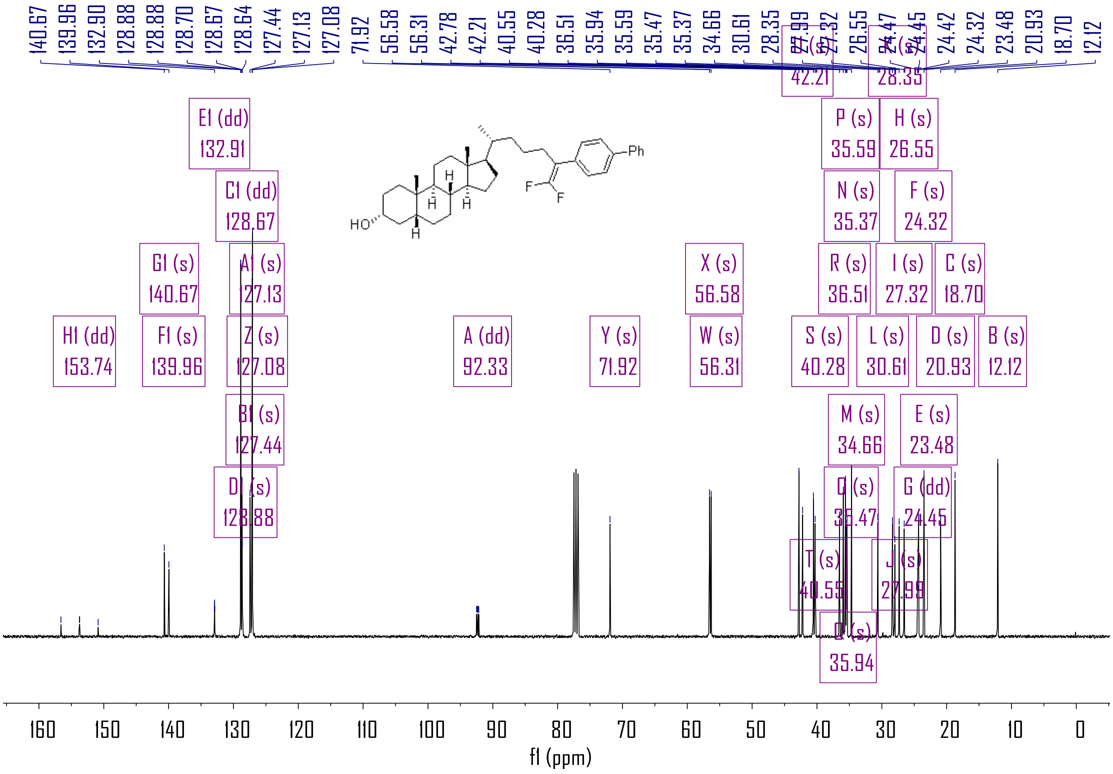

^{13}C NMR spectra for **3ao**.

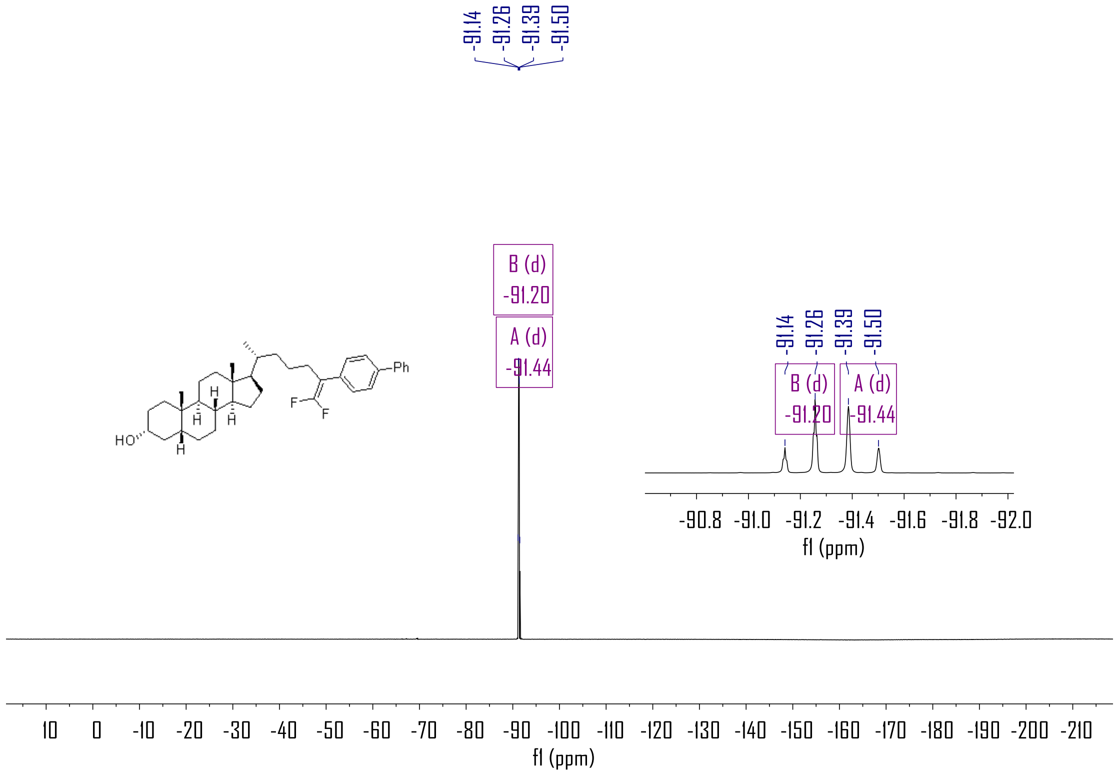

^{19}F NMR spectra for **3ao**.

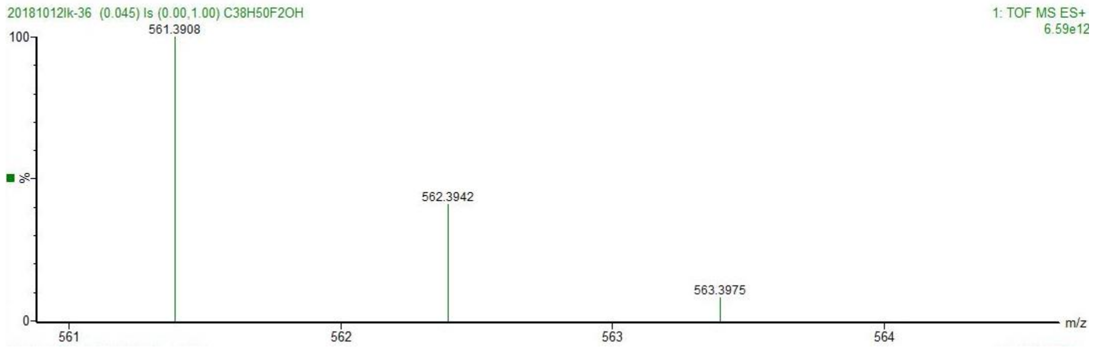

HRMS spectra for **3ao**.

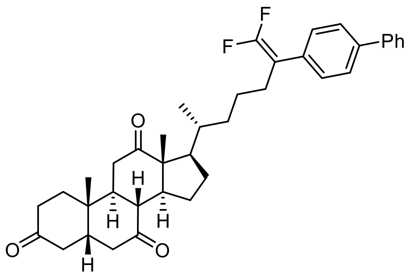

(5*S*,8*R*,9*S*,10*S*,13*R*,14*S*,17*R*)-17-((*R*)-6-([1,1'-biphenyl]-4-yl)-7,7-difluorohept-6-en-2-yl)-10,13-dimethyldodecahydro-3*H*-cyclopenta[*a*]phenanthrene-3,7,12(2*H*,4*H*)-trione (**3ap**)

Following general procedure, **1a** and **2p** were used. The product was isolated by column

chromatography as white solid (95.4 mg, 0.162 mmol, 81%)

Selectivity (desired C-F cleavage product : addition by-product) > 50:1.

R_f (petroleum ether : ethyl acetate = 2:1) = 0.42.

^{1}H NMR (400 MHz, Chloroform-*d*) δ 7.63 – 7.55 (m, 4H), 7.48 – 7.29 (m, 5H), 2.92 – 2.75 (m, 3H), 2.49 – 1.88 (m, 14H), 1.85 – 1.72 (m, 1H), 1.64 – 1.39 (m, 3H), 1.35 (s, 3H), 1.33 – 1.09 (m, 5H), 1.02 (s, 3H), 0.77 (d, *J* = 6.4 Hz, 3H).

^{13}C NMR (101 MHz, Chloroform-*d*) δ 212.07, 209.17, 208.81, 153.64 (dd, *J* = 290.5, 286.8 Hz), 140.51, 139.89, 132.71 (dd, *J* = 3.5, 3.5 Hz), 128.82, 128.60 (dd, *J* = 3.3, 3.3 Hz), 127.39, 127.06, 126.99, 92.20 (dd, *J* = 21.5, 12.7 Hz), 56.88, 51.80, 48.99, 46.80, 45.80, 45.52, 44.98, 42.79, 38.64, 36.47, 35.98, 35.75, 35.23, 34.71, 27.80, 27.76, 25.18, 24.71, 21.87, 18.98, 11.82.

^{19}F NMR (376 MHz, Chloroform-*d*) δ -91.15 (d, *J* = 43.8 Hz), -91.41 (d, *J* = 43.8 Hz).

HRMS (APCI) calcd for $C_{38}H_{45}O_3F_2^+$ [(M+H)$^+$] 587.33313, found 587.33215.

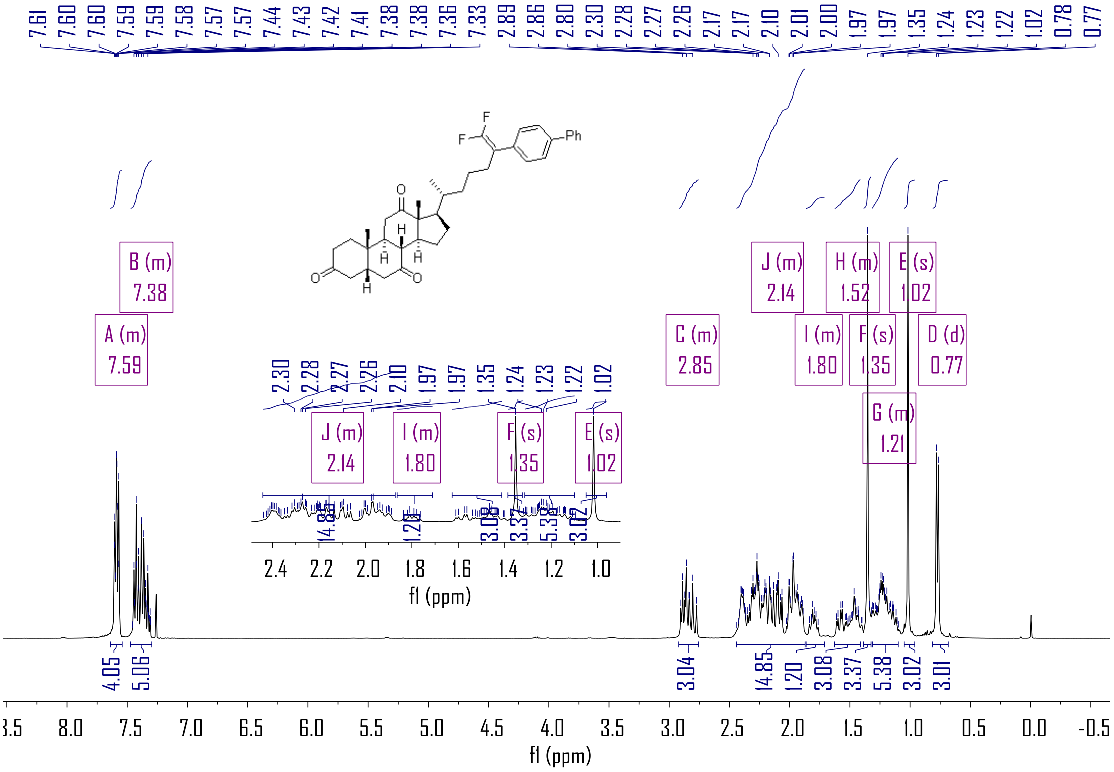

^{1}H NMR spectra for **3ap**.

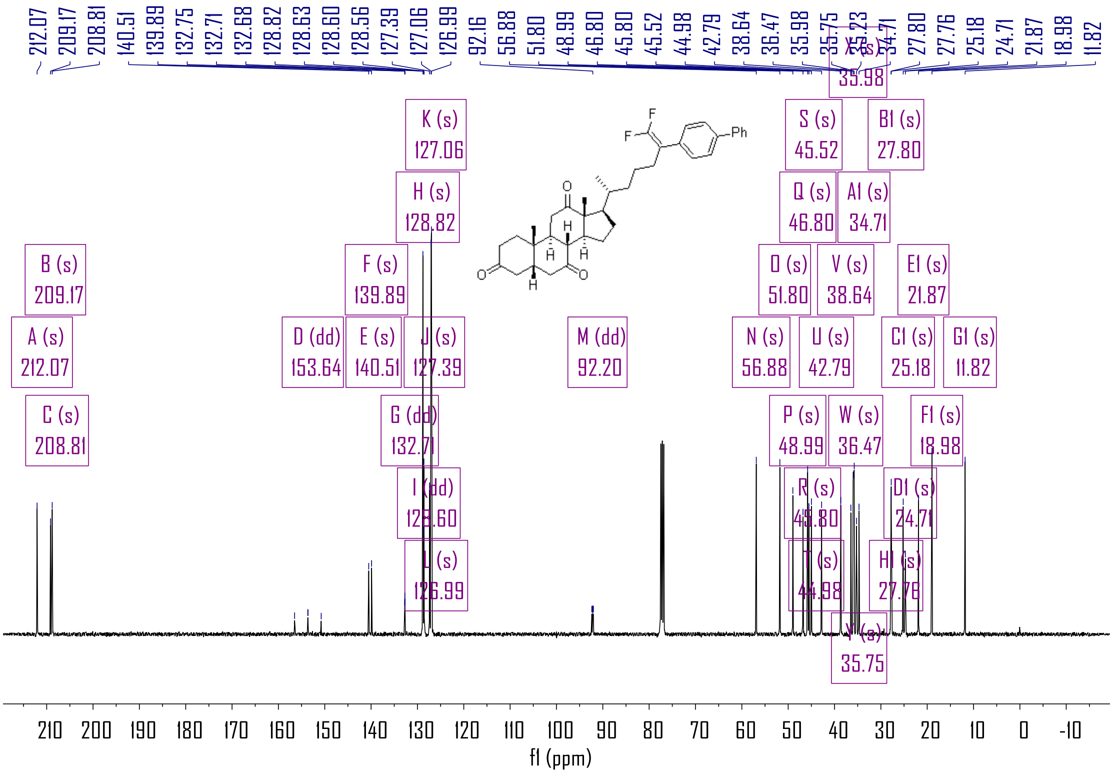

^{13}C NMR spectra for **3ap**.

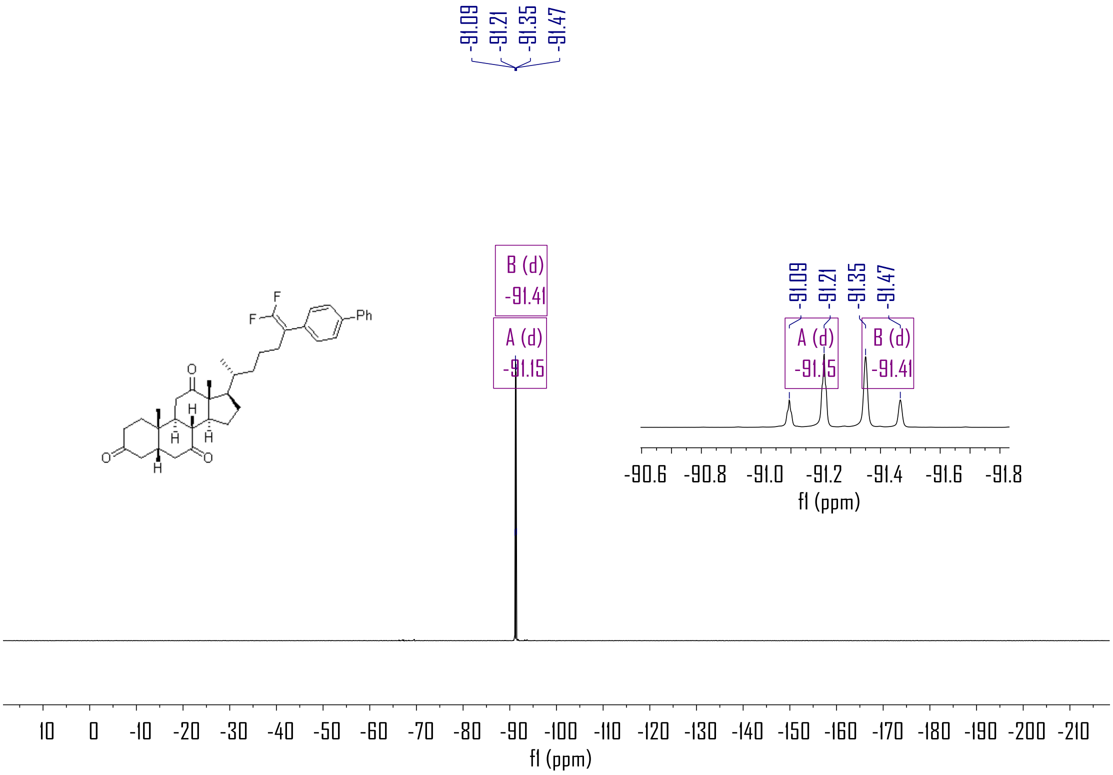

^{19}F NMR spectra for **3ap**.

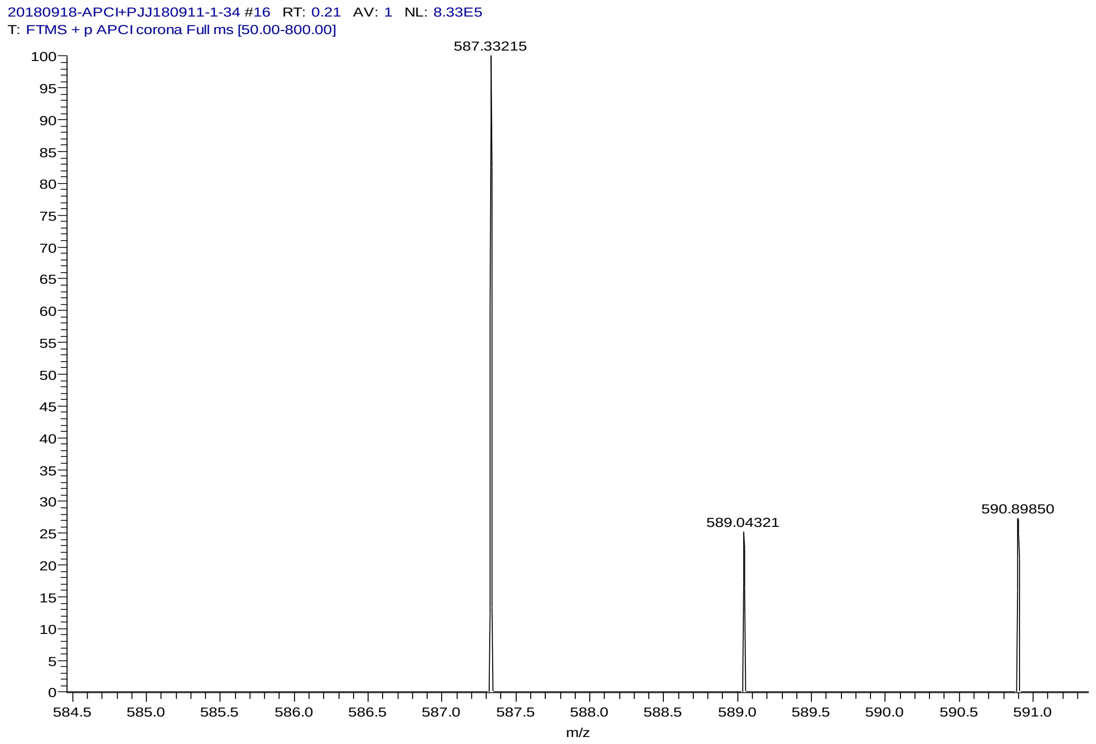

HRMS spectra for **3ap**.

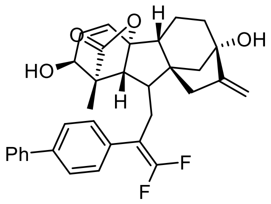

(1*S*,2*S*,4a*R*,4b*R*,7*S*,9a*R*,10a*R*)-10-(2-([1,1'-biphenyl]-4-yl)-3,3-difluoroallyl)-2,7-dihydroxy-1-methyl-8-methylene-1,2,4b,5,6,7,8,9,10,10a-decahydro-4a,1-(epoxymethano)-7,9a-methanobenzo[*a*]azulen-13-one (**3aq**)

Following general procedure, **1a** and **2q** were used. The product was isolated by column chromatography as white solid (23.1 mg, 0.044 mmol, 22%).

Selectivity (desired C-F cleavage product : addition by-product) > 50:1.

R_f (petroleum ether : ethyl acetate = 2:1) = 0.10.

^{1}H NMR (400 MHz, Chloroform-*d*) δ 7.64 – 7.55 (m, 4H), 7.44 (dd, *J* = 8.4, 6.8 Hz, 2H), 7.40 – 7.29 (m, 3H), 6.29 (d, *J* = 9.2 Hz, 1H), 5.80 (dd, *J* = 9.2, 3.6 Hz, 1H), 5.25 (dd, *J* = 3.1, 1.6 Hz, 1H), 5.03 (s, 1H), 4.11 (d, *J* = 3.7 Hz, 1H), 2.79 – 2.55 (m, 3H), 2.49 (d, *J* = 9.6 Hz, 1H), 2.24 – 1.90 (m, 6H), 1.81 – 1.61 (m, 4H), 1.48 (dd, *J* = 10.8, 1.7 Hz, 1H), 1.32 (s, 3H).

^{13}C NMR (101 MHz, Chloroform-*d*) δ 178.92, 156.53, 153.26 (dd, *J* = 291.6, 288.1 Hz), 140.78, 140.32, 133.72, 132.06, 131.35 (dd, *J* = 4.3, 3.1 Hz), 128.92, 128.85 (dd,

J = 3.1, 3.1 Hz), 127.67, 127.52, 127.20, 107.51, 91.00 (dd, J = 20.4, 13.7 Hz), 90.53, 78.17, 70.74, 56.46, 53.85, 51.33, 50.19, 46.91, 41.43, 41.13, 38.58, 29.81, 17.64, 15.46.

^{19}F NMR (376 MHz, Chloroform-*d*) δ -88.41 (d, J = 40.9 Hz), -89.20 (dd, J = 41.0, 3.2 Hz).

HRMS (APCI) calcd for $C_{33}H_{33}O_4F_2^+$ [(M+H)$^+$] 531.23414, found 531.23297.

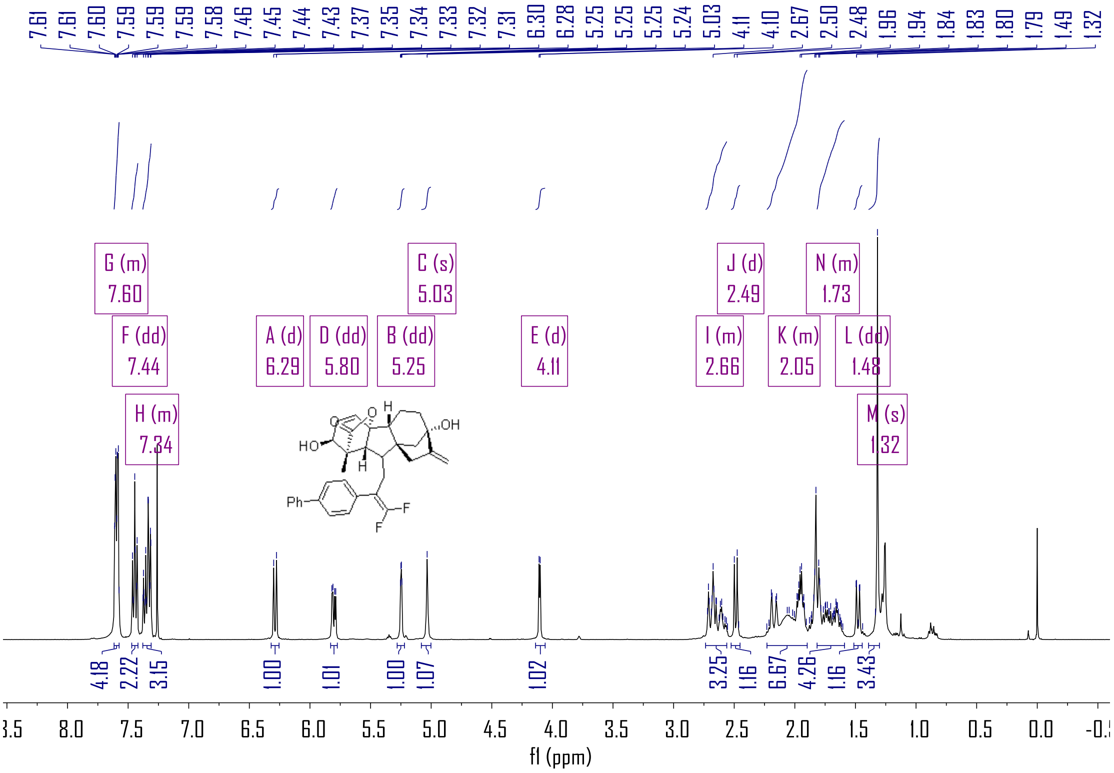

^{1}H NMR spectra for **3aq**.

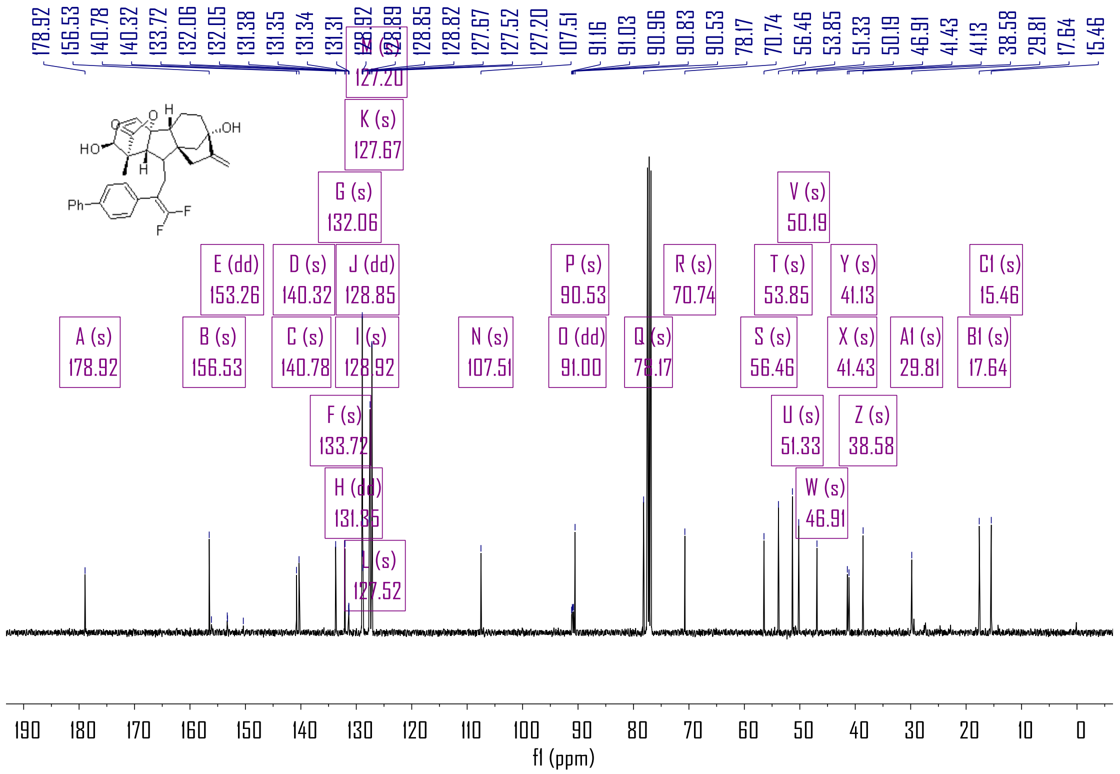

^{13}C NMR spectra for **3aq**.

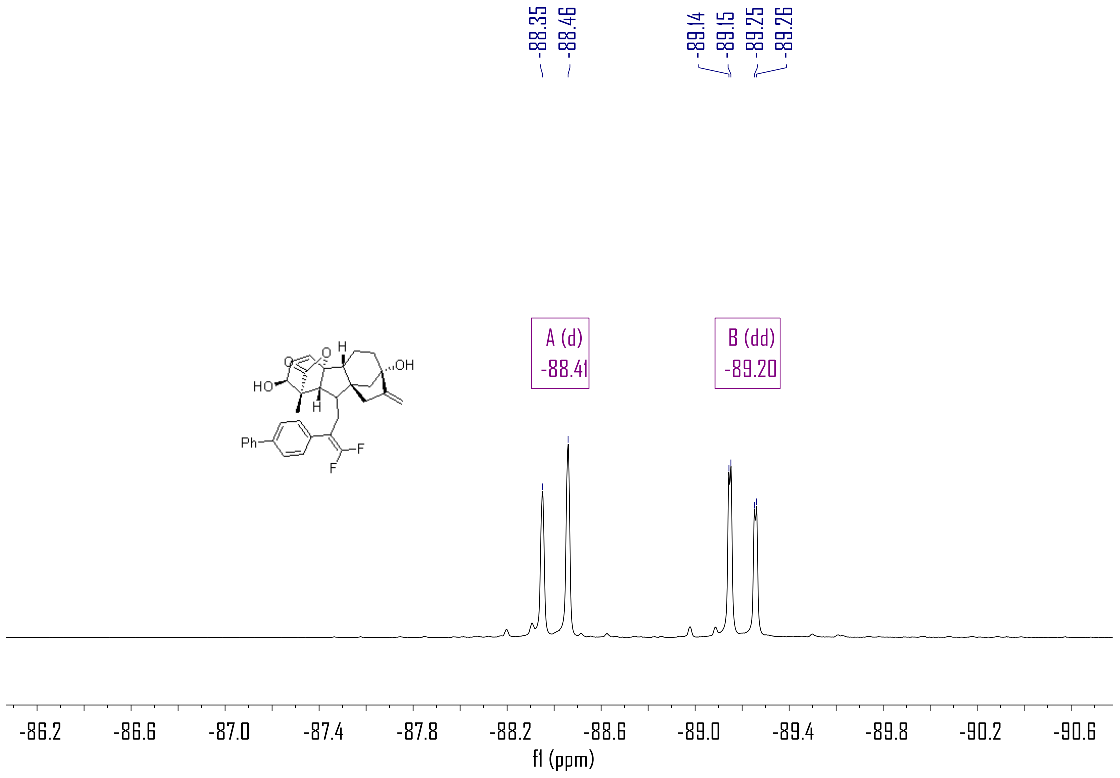

^{19}F NMR spectra for **3aq**.

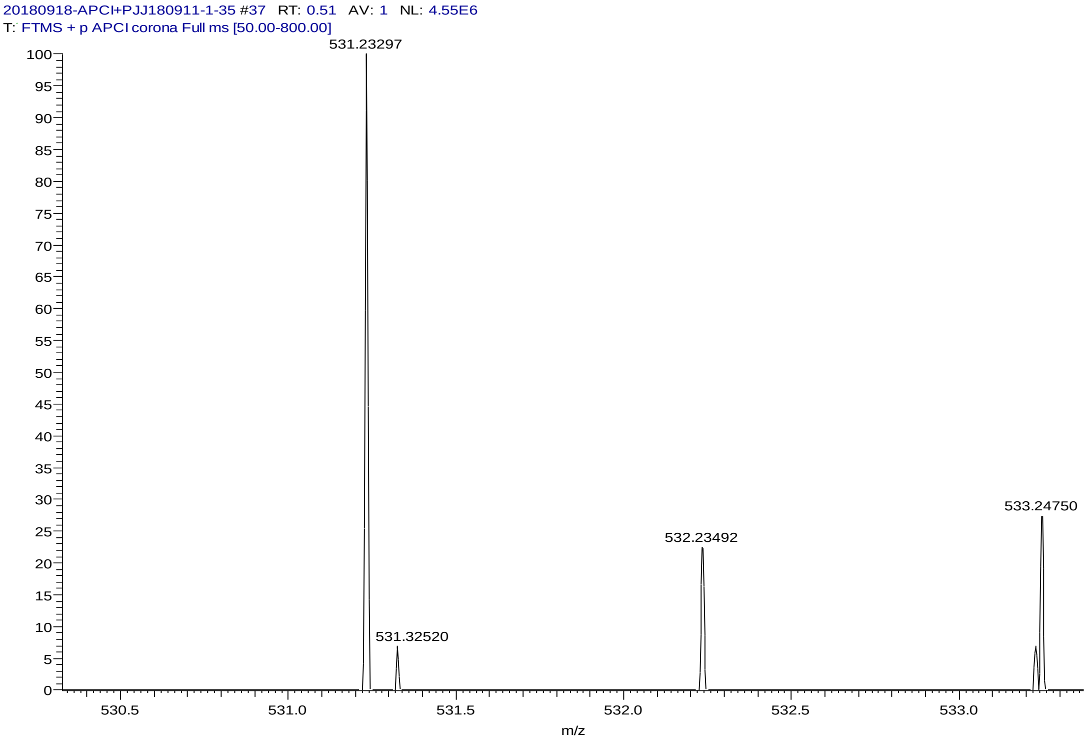

HRMS spectra for **3aq**.

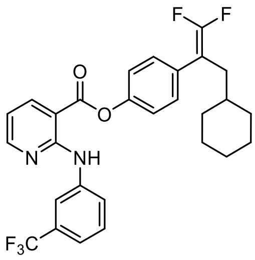

4-(3-cyclohexyl-1,1-difluoroprop-1-en-2-yl)phenyl 2-((3-(trifluoromethyl)phenyl)amino)nicotinate (**3ra**)

Following general procedure, **1r** and **2a** were used. The product was isolated by column chromatography as yellow oil (79.2 mg, 0.153 mmol, 77%).

Selectivity (desired C-F cleavage product : addition by-product) > 50:1.

R_f (petroleum ether : ethyl acetate = 5:1) = 0.74.

^{1}H NMR (400 MHz, Chloroform-*d*) δ 10.28 (s, 1H), 8.51 (d, *J* = 6.4 Hz, 2H), 8.11 (s, 1H), 7.93 – 7.82 (m, 1H), 7.49 – 7.38 (m, 3H), 7.34 – 7.28 (m, 1H), 7.22 (d, *J* = 8.7 Hz, 2H), 6.93 – 6.82 (m, 1H), 2.37 – 2.22 (m, 2H), 1.81 – 1.57 (m, 5H), 1.40 – 0.84 (m, 6H).

^{13}C NMR (101 MHz, Chloroform-*d*) δ 166.50, 156.26, 154.24 (dd, *J* = 290.8, 287.9 Hz), 154.11, 149.29, 140.93, 140.16, 132.49 (dd, *J* = 4.8, 3.1 Hz), 131.29 (q, *J* = 32.1 Hz), 129.69 (dd, *J* = 3.3, 3.3 Hz), 129.38, 124.25 (q, *J* = 272.4 Hz), 123.67, 121.76,

119.43 (q, J = 3.9 Hz), 117.38 (q, J = 4.0 Hz), 114.38, 106.56, 90.50 (dd, J = 22.9, 12.4 Hz), 35.74 (dd, J = 2.4, 2.4 Hz), 35.37, 32.98, 26.51, 26.16.

^{19}F NMR (376 MHz, Chloroform-*d*) δ -62.64 , -90.48 (d, J = 42.3 Hz), -91.06 (d, J = 42.7 Hz).

HRMS (ESI) calcd for $C_{28}H_{26}O_2N_2F_5^+$ [$(M+H)^+$] 517.19090, found 517.19031.

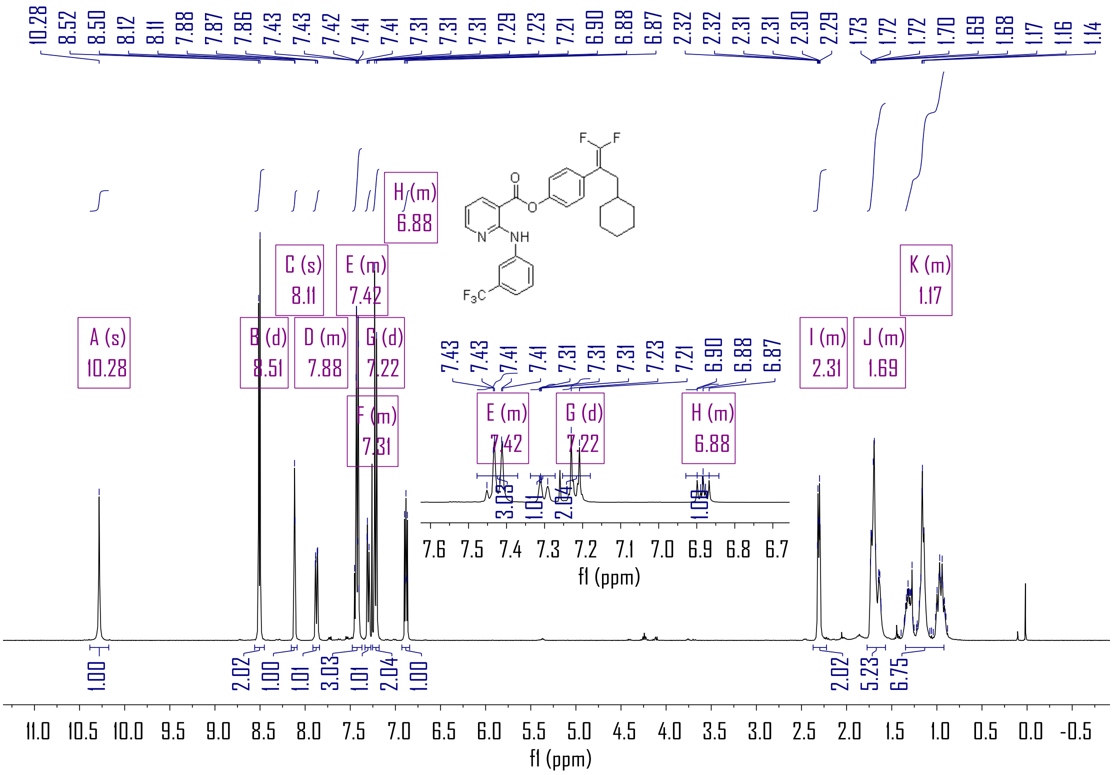

^{1}H NMR spectra for **3ra**.

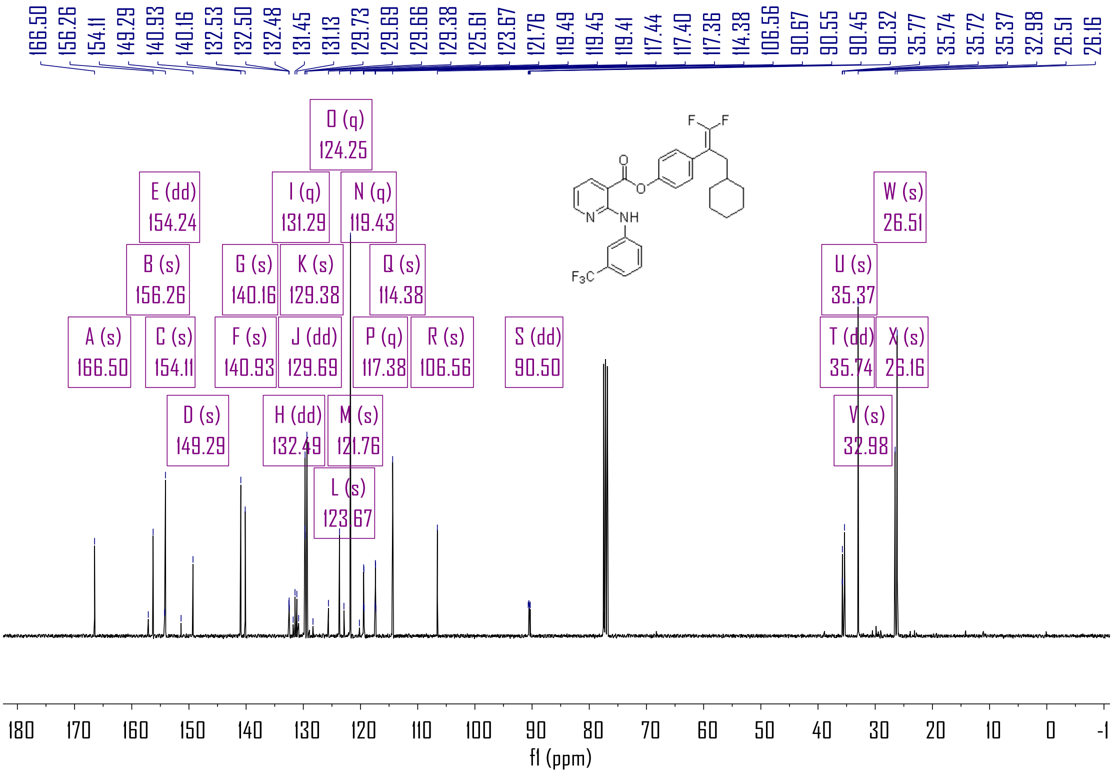

^{13}C NMR spectra for **3ra**.

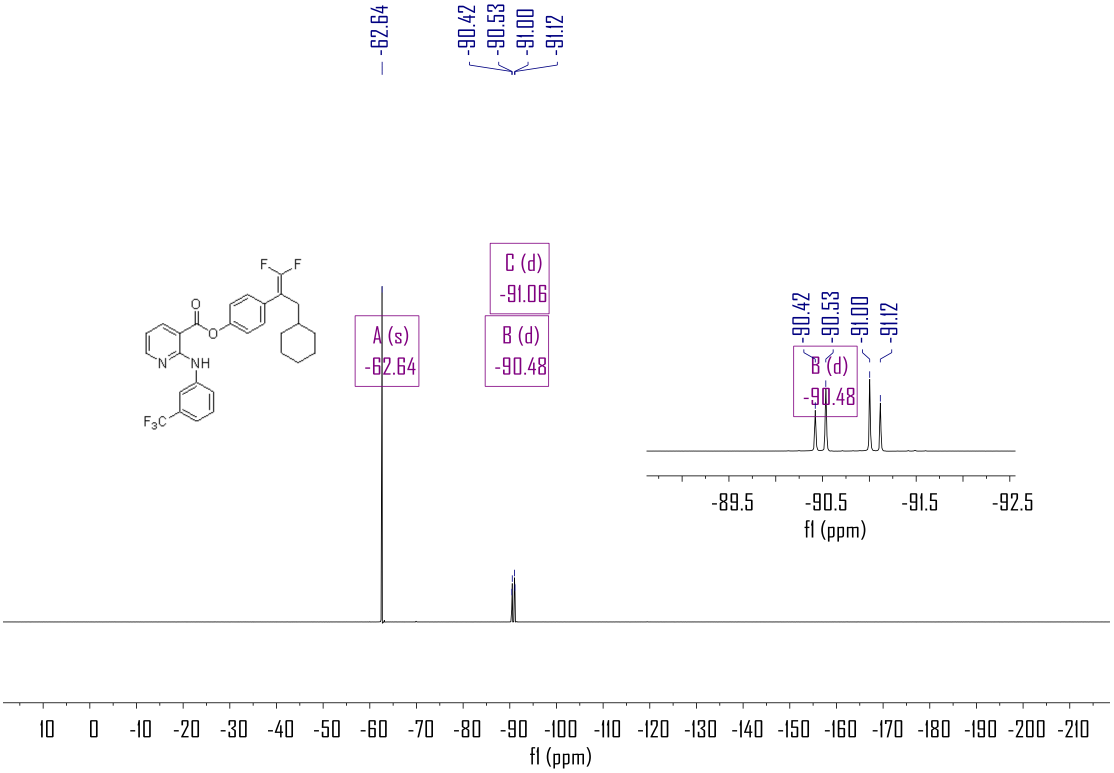

^{19}F NMR spectra for **3ra**.

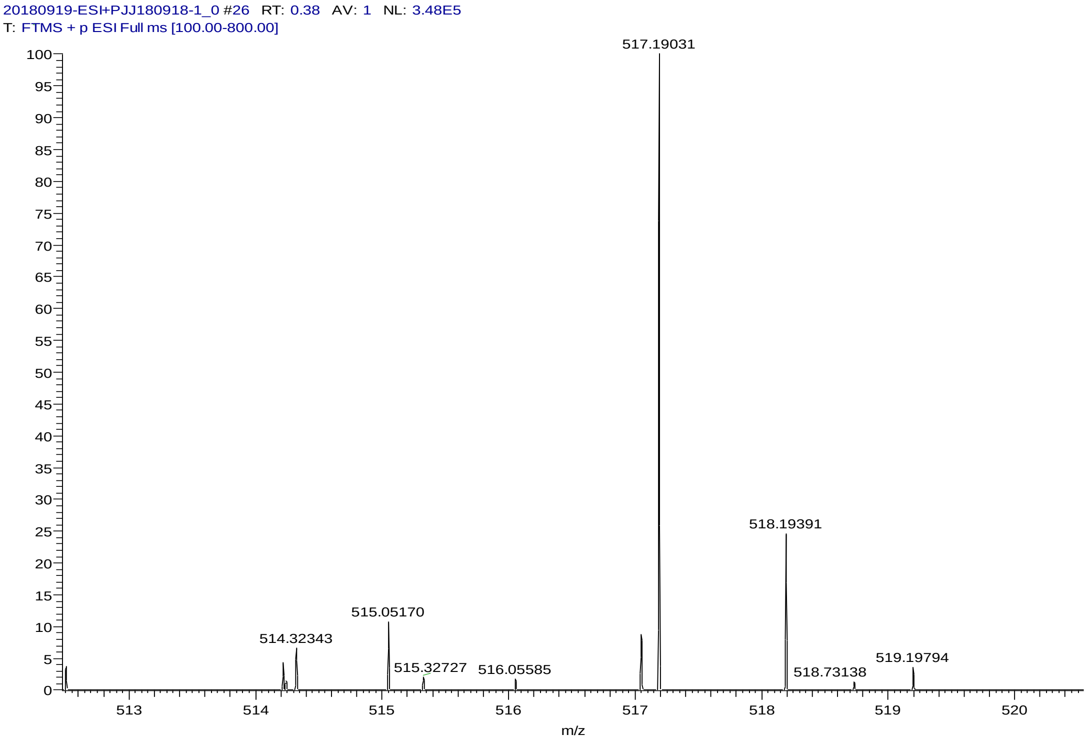

HRMS spectra for **3ra**.

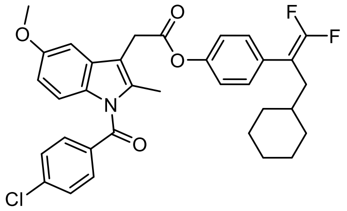

4-(3-cyclohexyl-1,1-difluoroprop-1-en-2-yl)phenyl 2-(1-(4-chlorobenzoyl)-5-methoxy-2-methyl-1*H*-indol-3-yl)acetate (**3sa**)

Following general procedure, **1s** and **2a** were used. The product was isolated by column chromatography as yellow oil (80.7 mg, 0.136 mmol, 68%)

Selectivity (desired C-F cleavage product : addition by-product) > 50:1.

R_f (petroleum ether : ethyl acetate = 5:1) = 0.83.

^{1}H NMR (400 MHz, Chloroform-*d*) δ 7.68 (d, *J* = 8.5 Hz, 2H), 7.48 (d, *J* = 8.5 Hz, 2H), 7.28 (d, *J* = 8.2 Hz, 2H), 7.10 – 7.01 (m, 3H), 6.89 (d, *J* = 9.0 Hz, 1H), 6.70 (dd, *J* = 9.0, 2.5 Hz, 1H), 3.91 (s, 2H), 3.84 (s, 3H), 2.46 (s, 3H), 2.29 – 2.18 (m, 2H), 1.72 – 1.54 (m, 7H), 1.24 (d, *J* = 13.1 Hz, 2H), 0.97 – 0.84 (m, 2H).

^{13}C NMR (101 MHz, Chloroform-*d*) δ 169.34, 168.39, 156.23, 154.11 (dd, *J* = 290.3, 286.2 Hz), 149.66, 139.46, 136.33, 133.91, 131.90 (dd, *J* = 3.7, 3.3 Hz), 131.31, 130.95, 130.58, 129.40 (dd, *J* = 3.4, 3.4 Hz), 129.25, 121.42, 115.13, 112.05, 111.90, 101.32, 90.48 (dd, *J* = 22.7, 12.7 Hz), 55.84, 35.66, 35.32, 32.92, 30.70, 26.47, 26.11, 13.54.

^{19}F NMR (376 MHz, Chloroform-*d*) δ -90.88 (d, J = 43.1 Hz), -91.39 (d, J = 43.2 Hz).

HRMS (ESI) calcd for $C_{34}H_{33}O_4NClF_2^+$ [(M+H)$^+$] 592.20607, found 592.20544.

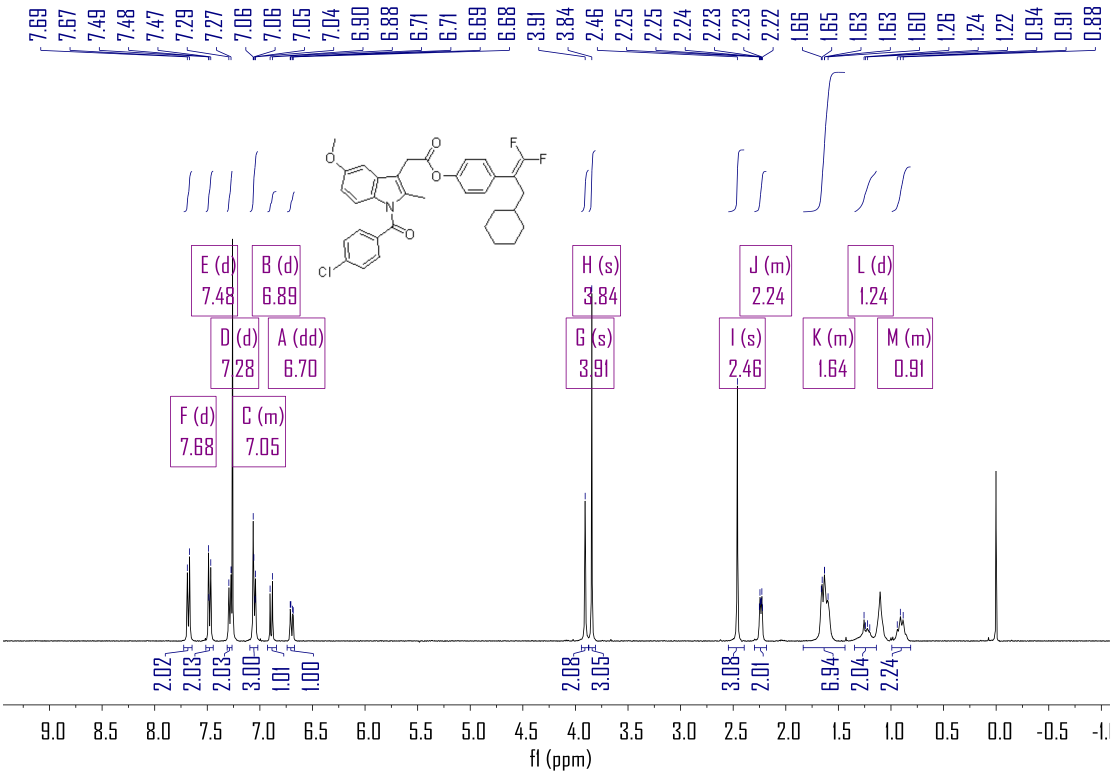

^{1}H NMR spectra for **3sa**.

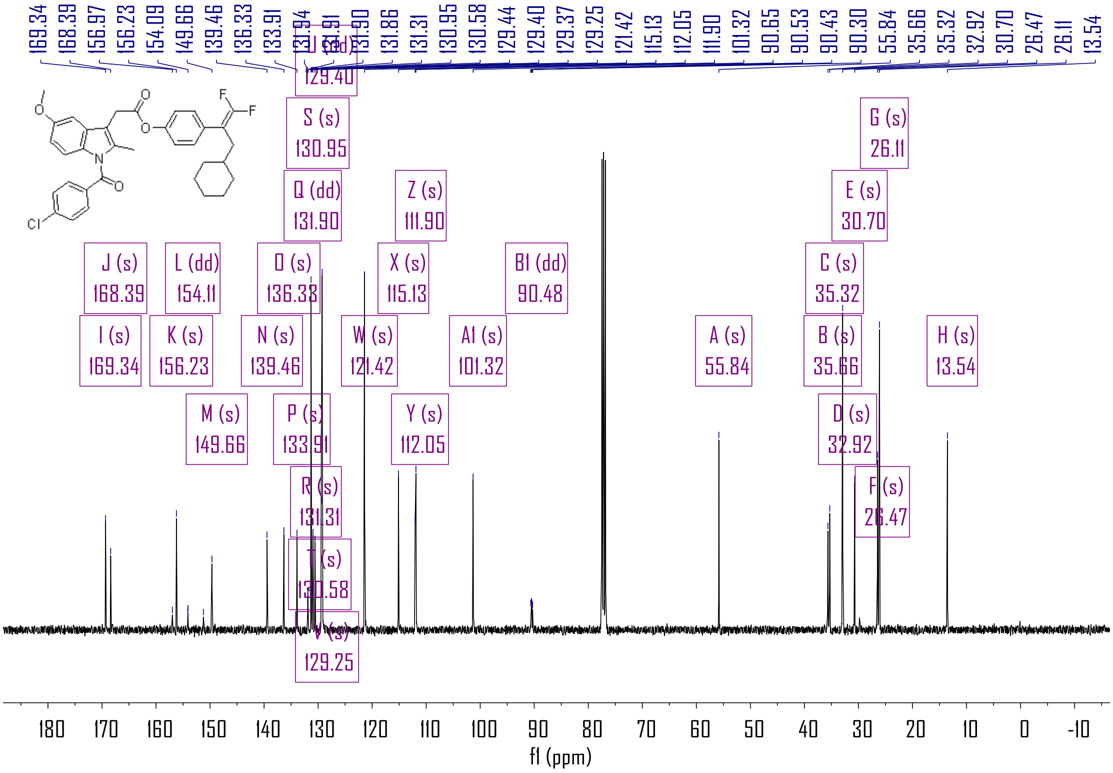

^{13}C NMR spectra for **3sa**.

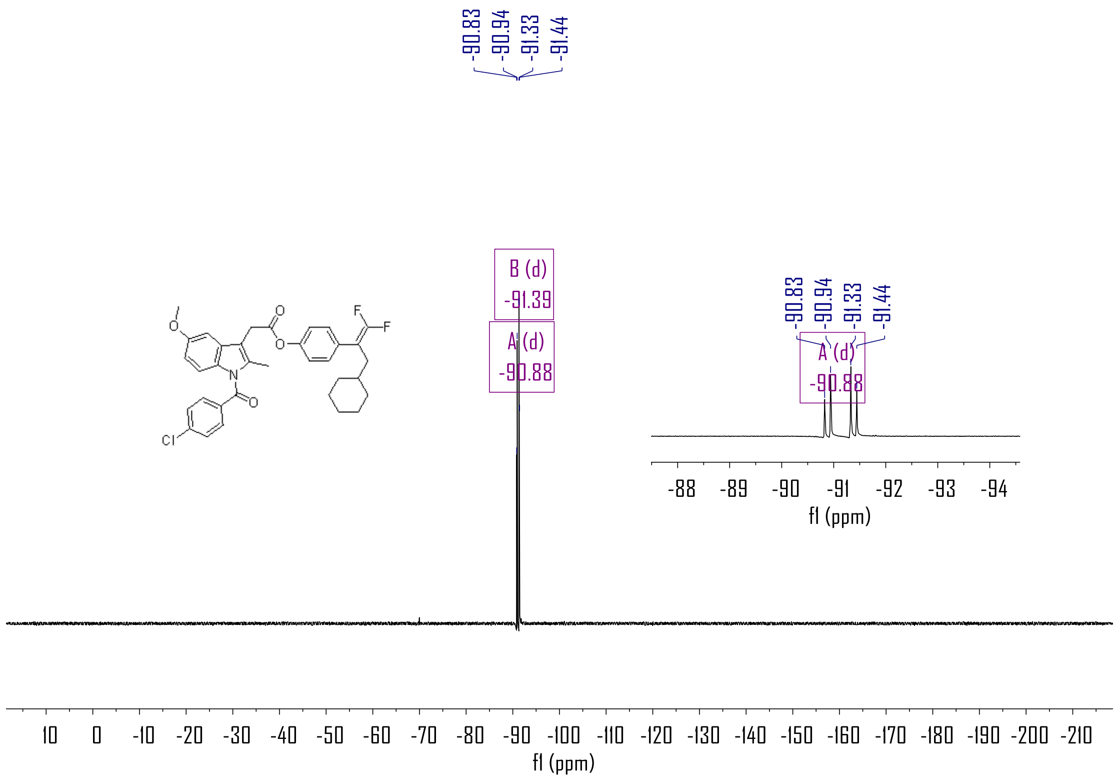

^{19}F NMR spectra for **3sa**.

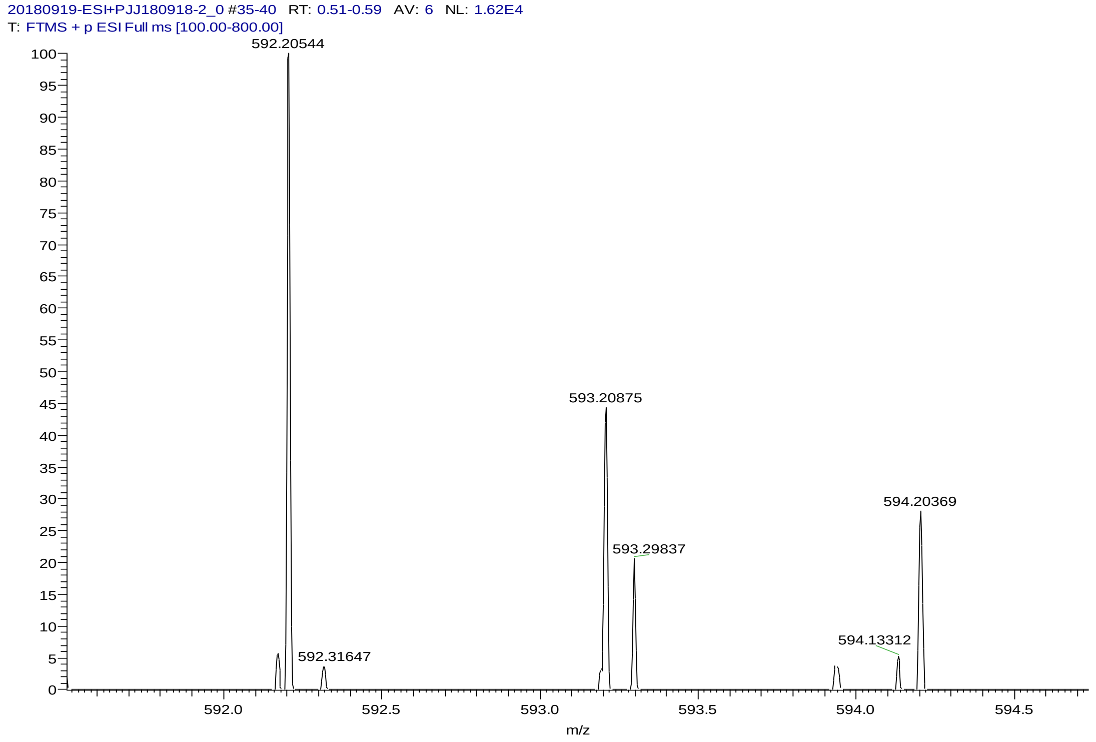

HRMS spectra for **3sa**.

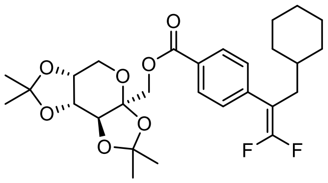

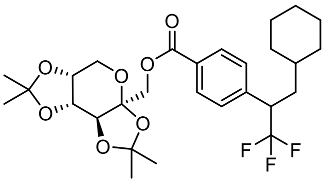

3ta **3ta'**

((3a*S*,5a*R*,8a*R*,8b*S*)-2,2,7,7-tetramethyltetrahydro-3a*H*-bis([1,3]dioxolo)[4,5-*b*:4',5'-*d*]pyran-3a-yl)methyl 4-(3-cyclohexyl-1,1-difluoroprop-1-en-2-yl)benzoate (**3ta**)
((3a*S*,5a*R*,8a*R*,8b*S*)-2,2,7,7-tetramethyltetrahydro-3a*H*-bis([1,3]dioxolo)[4,5-*b*:4',5'-*d*]pyran-3a-yl)methyl 4-(3-cyclohexyl-1,1,1-trifluoropropan-2-yl)benzoate (**3ta'**)
Following general procedure, **1t** and **2a** were used. The product was isolated by column chromatography as colorless oil, 90.1 mg inseparable mixture was obtained, **3ta** (0.150 mmol, 75%) and **3am'** (0.022 mmol, 11%).

Selectivity (desired C-F cleavage product : addition by-product) = 7:1.

R_f (petroleum ether : ethyl acetate = 5:1) = 0.72.

^{1}H NMR (400 MHz, Chloroform-*d*) δ 8.05 (d, *J* = 8.5 Hz, 2H), 7.38 (dd, *J* = 8.5, 1.4 Hz, 2H), 4.72 – 4.58 (m, 2H), 4.46 (d, *J* = 2.7 Hz, 1H), 4.33 (d, *J* = 11.9 Hz, 1H), 4.26 (dd, *J* = 7.9, 1.6 Hz, 1H), 3.96 (dd, *J* = 13.0, 1.9 Hz, 1H), 3.80 (d, *J* = 13.0 Hz, 1H), 2.38 – 2.26 (m, 2H), 1.83 – 1.58 (m, 5H), 1.55 (s, 3H), 1.45 (s, 3H), 1.40 (s, 3H), 1.34 (s, 3H), 1.26 – 0.76 (m, 6H).

^{13}C NMR (101 MHz, Chloroform-*d*) δ 165.75, 154.30 (dd, *J* = 292.6, 287.6 Hz), 139.35 (dd, *J* = 5.0, 3.5 Hz), 129.95, 128.60, 128.30 (dd, *J* = 3.5, 3.5 Hz), 109.27, 108.94, 101.78, 90.97 (dd, *J* = 22.9, 11.7 Hz), 70.90, 70.69, 70.21, 65.60, 61.46, 35.97 (dd, *J* = 2.4, 2.4 Hz), 34.92, 32.93, 26.64, 26.43, 26.13, 25.97, 25.67, 24.12.

^{19}F NMR (376 MHz, Chloroform-*d*) (**3ta**) δ -88.79 (d, *J* = 38.8 Hz), -89.50 (d, *J* = 38.2 Hz).

^{19}F NMR (376 MHz, Chloroform-*d*) (**3ta'**) δ -69.59 (d, *J* = 9.1 Hz).

HRMS (APCI) calcd for $C_{28}H_{37}O_7F_2^+$ [(M+H)$^+$] 523.25019, found 523.24908.

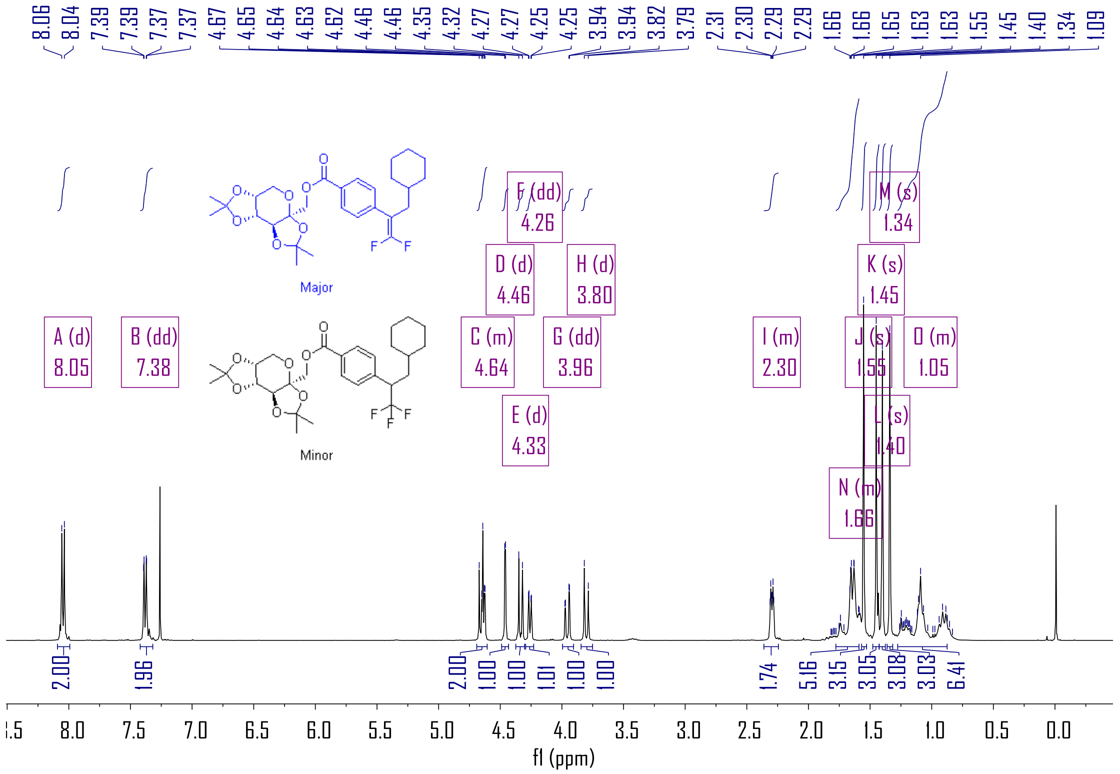

^{1}H NMR spectra for **3ta** & **3ta’**.

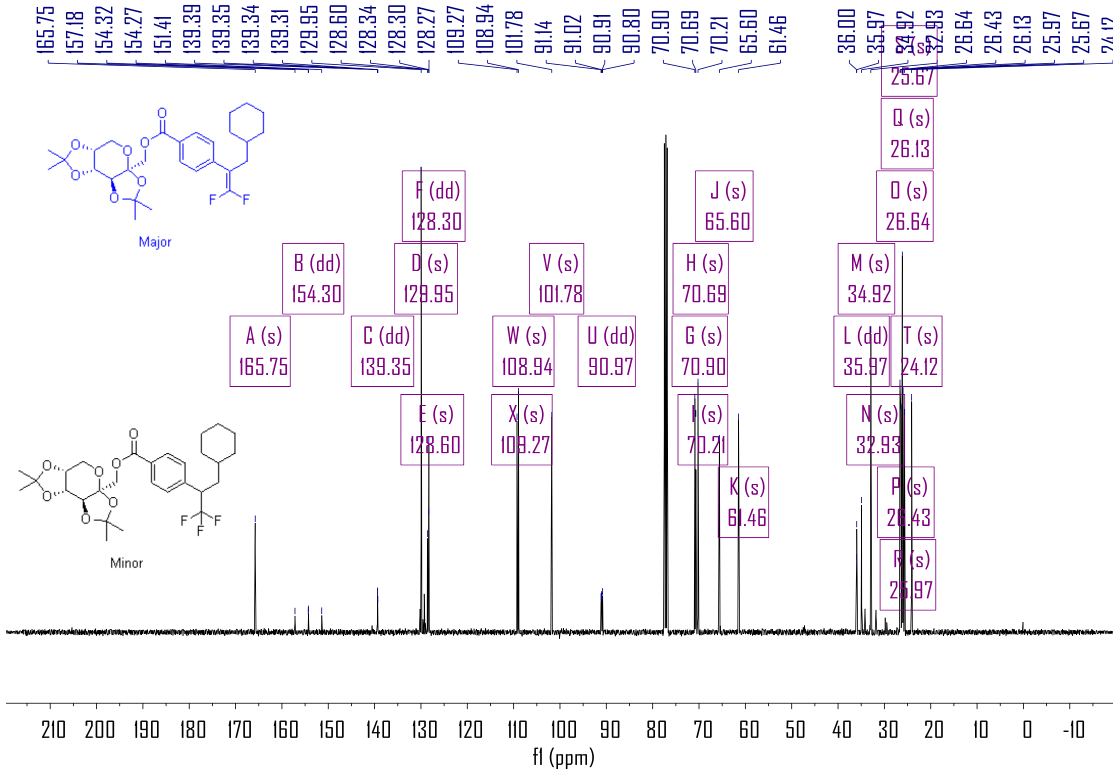

^{13}C NMR spectra for **3ta** & **3ta’**.

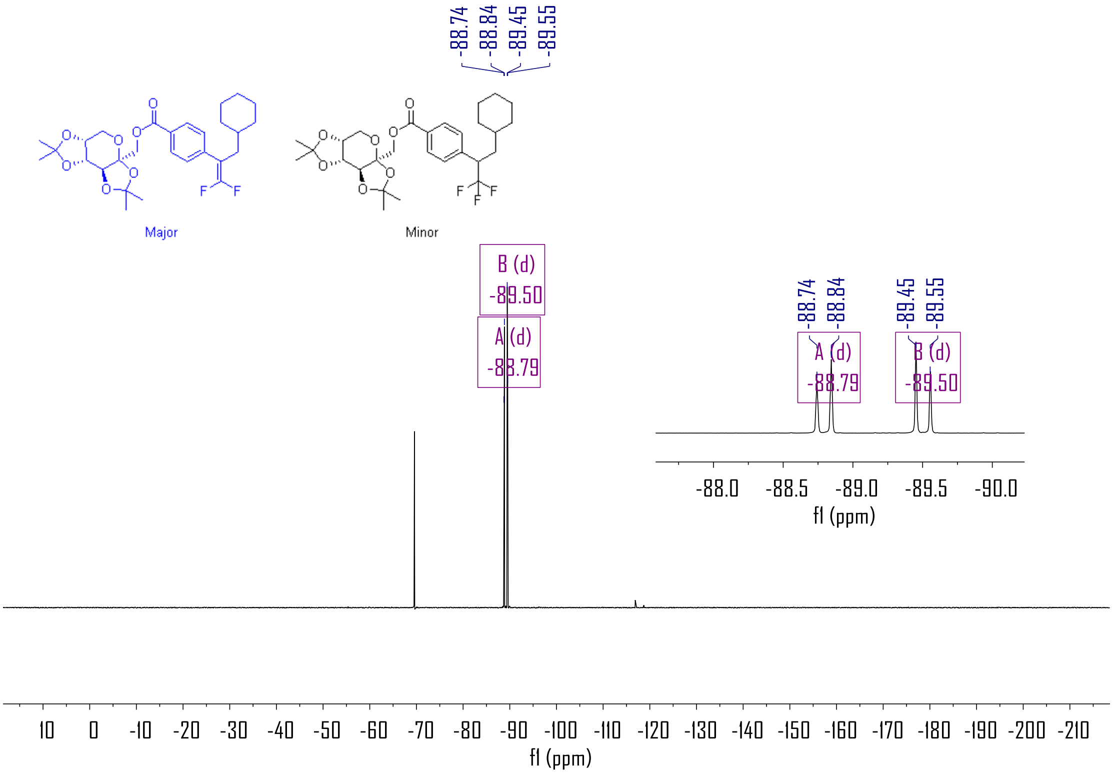

^{19}F NMR spectra for **3ta**.

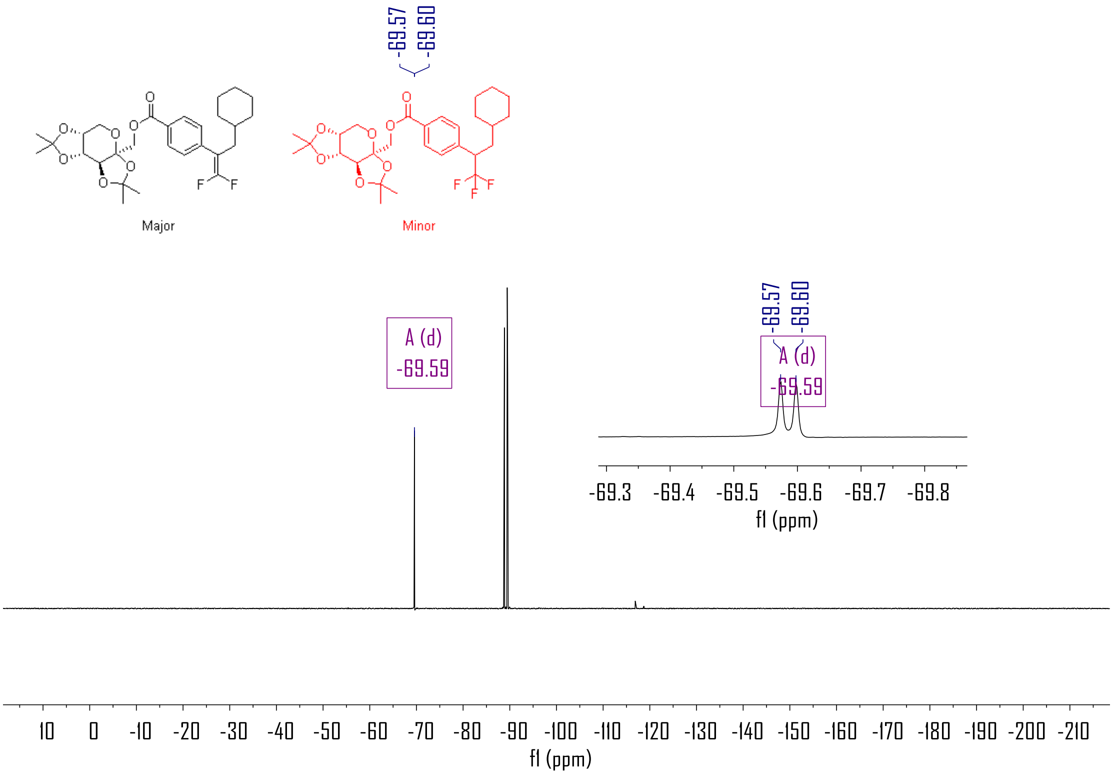

^{19}F NMR spectra for **3ta’**.

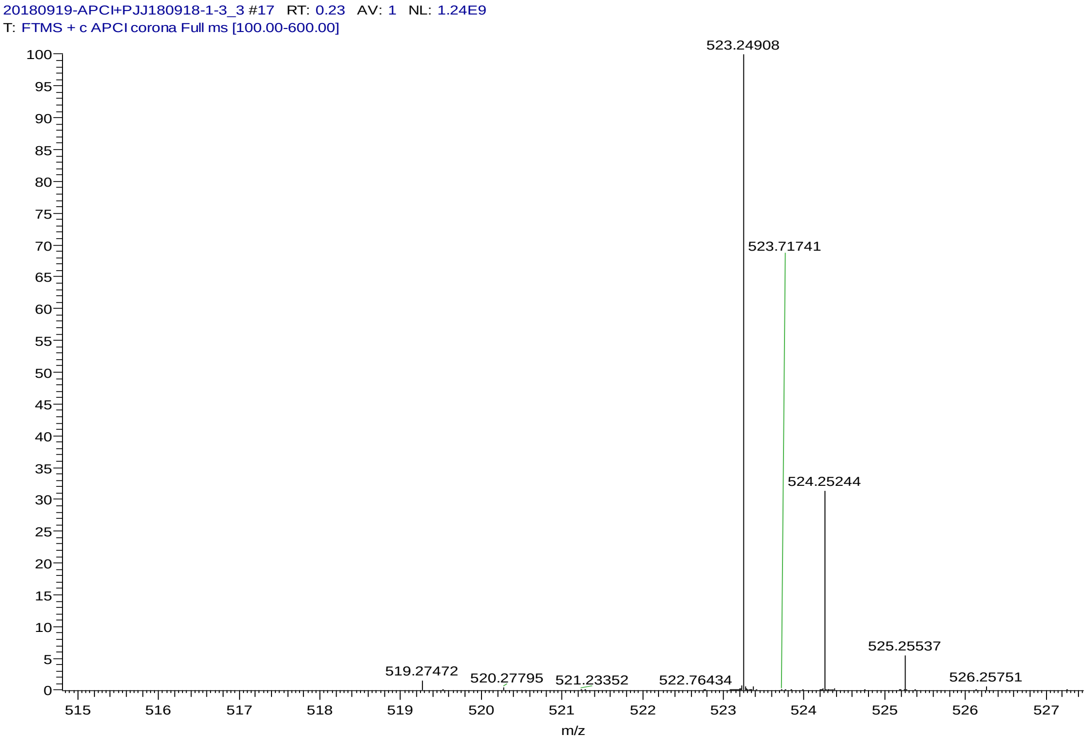

HRMS spectra for **3ta**.

9. General Procedure for Examples Described in Table 5

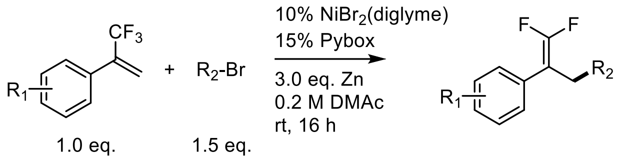

General procedure for examples described in Table 5.

$NiBr_2$(diglyme) (0.02 mmol, 10 mol%, 7.1 mg), Pybox (0.03 mmol, 15 mol%, 6.5 mg) and Zn (0.6 mmol, 3.0 equiv., 39 mg) were added to a Schlenk tube equipped with a stir bar. The Schlenk tube was evacuated and filled with argon (three cycles). To these solids, 1 mL DMAc (0.2 M) was added under argon atmosphere. Then, trifluoromethyl alkene (0.2 mmol, 1.0 equiv.) and alkyl bromides (0.3 mmol, 1.5 equiv.) were added and stirred at room temperature (~20 ℃) for 16 hours. The mixture was purified by column chromatography to afford the desired product.

10. Examples Described in Table 5

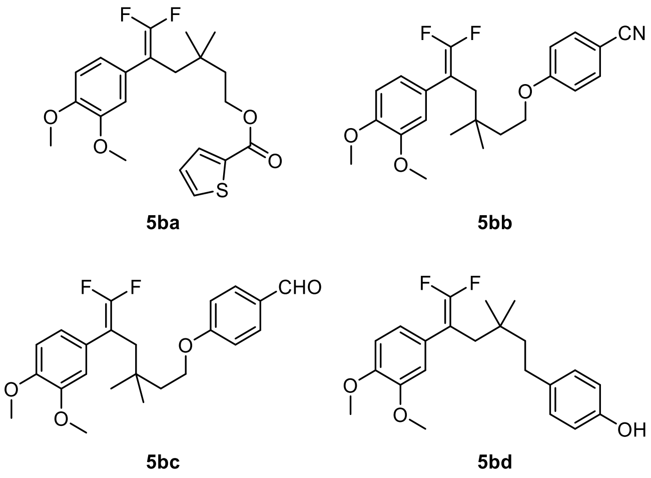

List of substrates in Table 5.

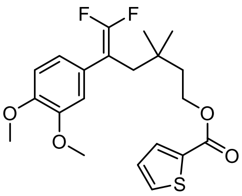

5-(3,4-dimethoxyphenyl)-6,6-difluoro-3,3-dimethylhex-5-en-1-yl thiophene-2-carboxylate (**5ba**)

Following general procedure, **1b** and **4a** were used. The product was isolated by column chromatography as colorless oil (71.3 mg, 0.174 mmol, 87%).

Selectivity (desired C-F cleavage product : addition by-product) > 50:1.

R_f (petroleum ether : ethyl acetate = 10:1) = 0.30.

^{1}H NMR (400 MHz, Chloroform-*d*) δ 7.74 (dt, *J* = 3.7, 1.0 Hz, 1H), 7.58 – 7.46 (m, 1H), 7.13 – 7.03 (m, 1H), 6.92 – 6.77 (m, 3H), 4.26 (t, *J* = 7.1 Hz, 2H), 3.86 (s, 3H), 3.83 (s, 3H), 2.38 (s, 2H), 1.62 (t, *J* = 7.1 Hz, 2H), 0.86 (s, 6H).

^{13}C NMR (101 MHz, Chloroform-*d*) δ 162.21, 154.43 (dd, *J* = 289.5, 287.6 Hz), 148.79, 148.21, 134.00, 133.29, 132.28, 127.75, 127.68 (dd, *J* = 4.4, 2.6 Hz), 120.98 (dd, *J* = 2.5, 2.5 Hz), 111.78 (dd, *J* = 2.6, 2.6 Hz), 111.05, 90.25 (dd, *J* = 21.4, 13.8 Hz), 62.27, 55.99, 55.80, 40.17, 40.07, 34.66 (dd, *J* = 2.4, 2.4 Hz), 27.34.

^{19}F NMR (376 MHz, Chloroform-*d*) δ -89.89 (d, *J* = 41.9 Hz), -91.74 (d, *J* = 42.0 Hz).

HRMS (APCI) calcd for $C_{21}H_{25}O_4F_2S^+$ [$(M+H)^+$] 411.14361, found 411.14279.

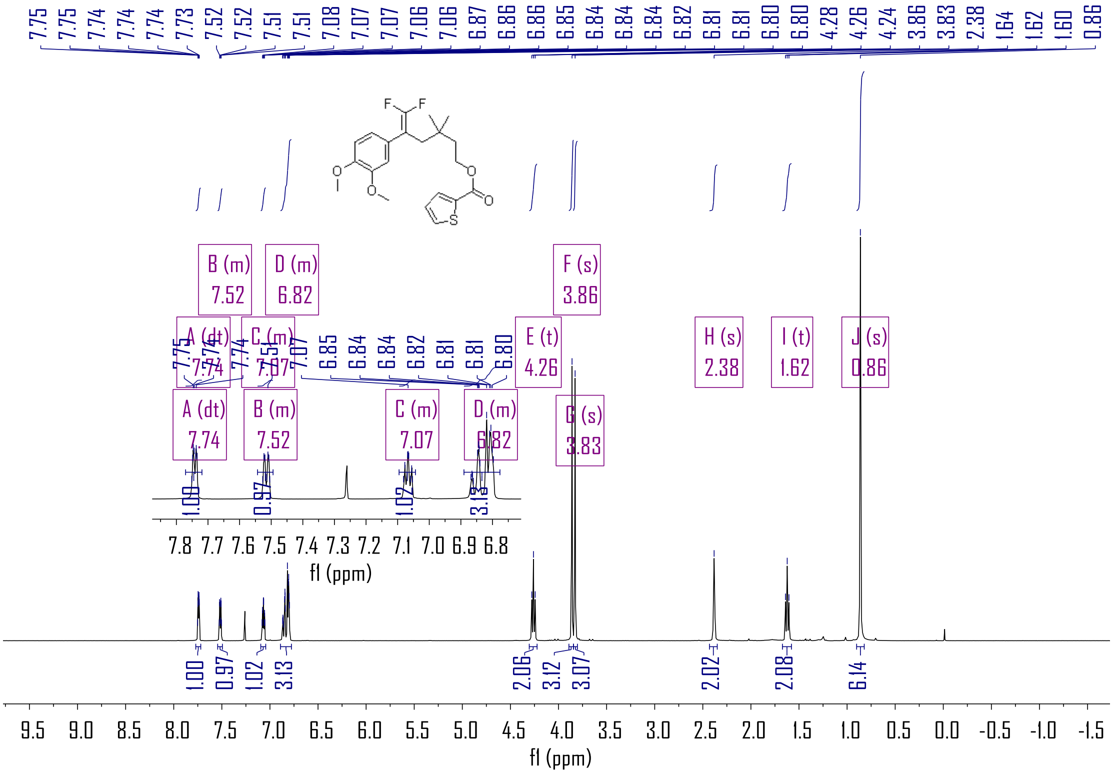

^{1}H NMR spectra for **5ba**.

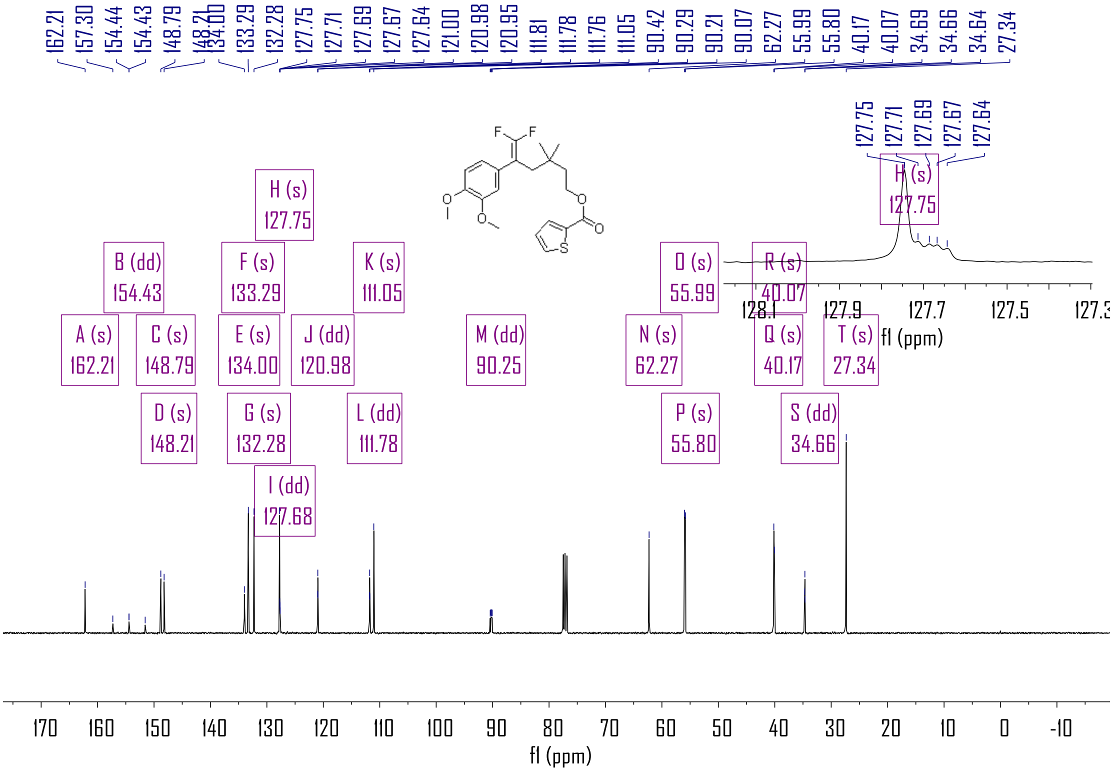

^{13}C NMR spectra for **5ba**.

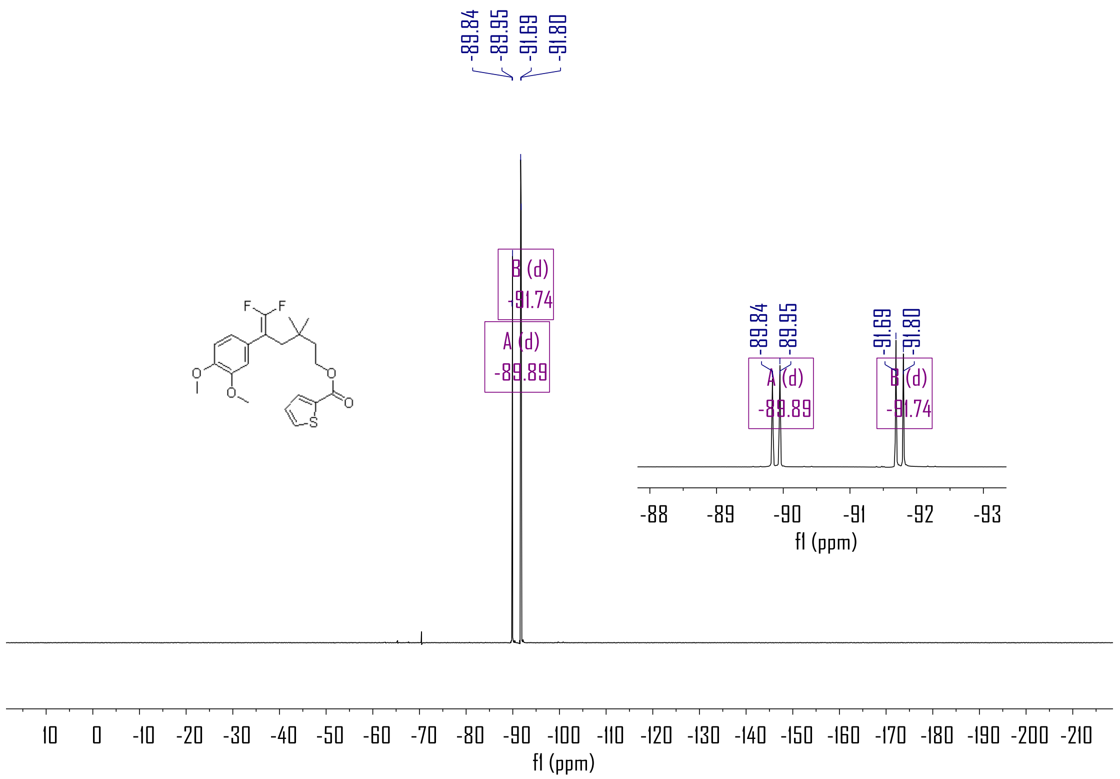

^{19}F NMR spectra for **5ba**.

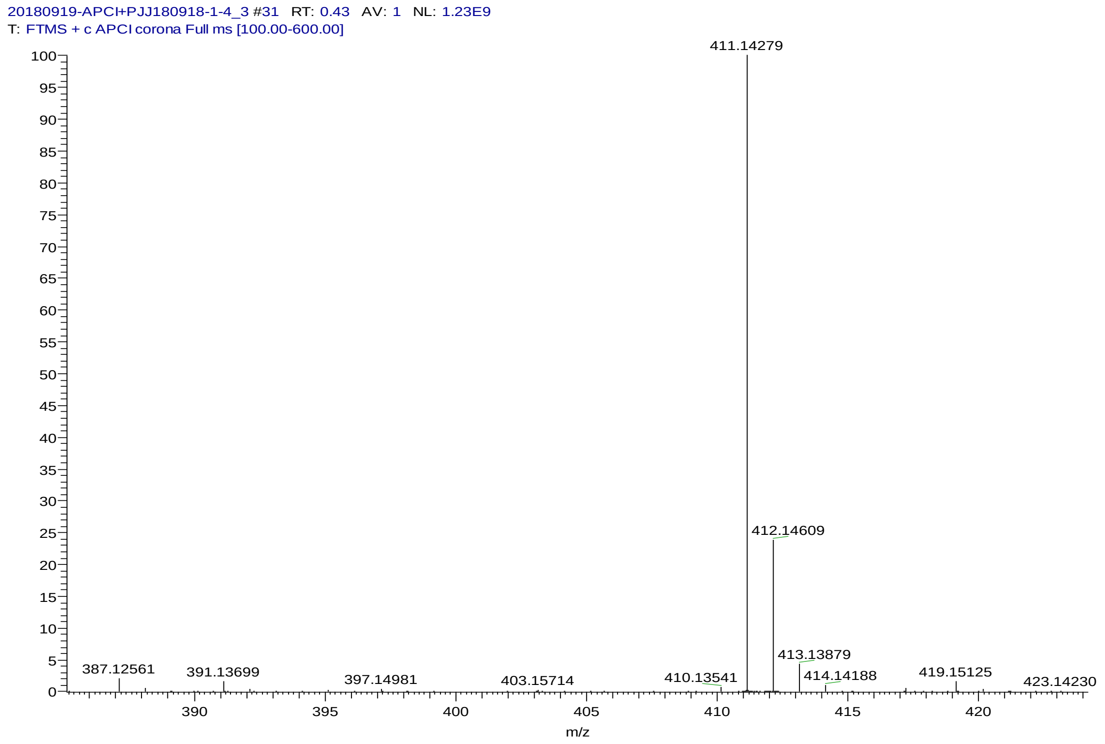

HRMS spectra for **5ba**.

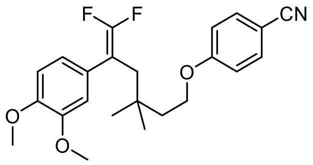

4-((5-(3,4-dimethoxyphenyl)-6,6-difluoro-3,3-dimethylhex-5-en-1-yl)oxy)benzonitrile (**5bb**)

Following general procedure, **1b** and **4b** were used. The product was isolated by column chromatography as colorless oil (73.0 mg, 0.182 mmol, 91%).

Selectivity (desired C-F cleavage product : addition by-product) > 50:1.

R_f (petroleum ether : ethyl acetate = 10:1) = 0.30.

^{1}H NMR (400 MHz, Chloroform-*d*) δ 7.52 (d, *J* = 8.8 Hz, 2H), 6.99 – 6.74 (m, 5H), 3.93 (t, *J* = 7.0 Hz, 2H), 3.85 (s, 3H), 3.83 (s, 3H), 2.38 (s, 2H), 1.67 (t, *J* = 7.0 Hz, 2H), 0.87 (s, 6H).

^{13}C NMR (101 MHz, Chloroform-*d*) δ 162.17, 154.40 (dd, *J* = 289.5, 287.8 Hz), 148.77, 148.22, 133.90, 127.60 (dd, *J* = 2.6, 2.6 Hz), 120.95, 119.26, 115.15, 111.80 (dd, *J* = 3.1, 2.9 Hz), 111.02, 103.68, 90.17 (dd, *J* = 21.3, 13.9 Hz), 65.27, 55.98, 55.80, 40.27, 40.24, 34.60 (dd, *J* = 2.6, 2.6 Hz), 27.43.

^{19}F NMR (376 MHz, Chloroform-*d*) δ -89.83 (d, *J* = 41.8 Hz), -91.61 (d, *J* = 41.9 Hz).

HRMS (APCI) calcd for $C_{23}H_{26}O_3NF_2^+$ [(M+H)$^+$] 402.18753, found 402.18683.

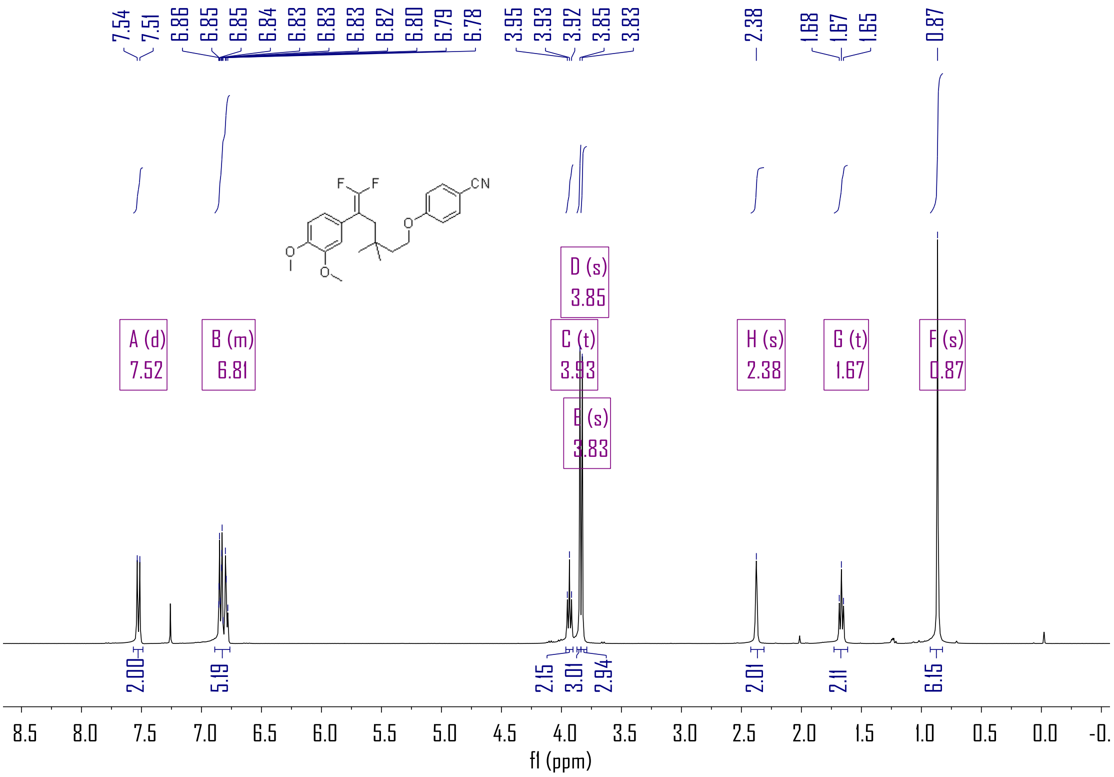

^{1}H NMR spectra for **5bb**.

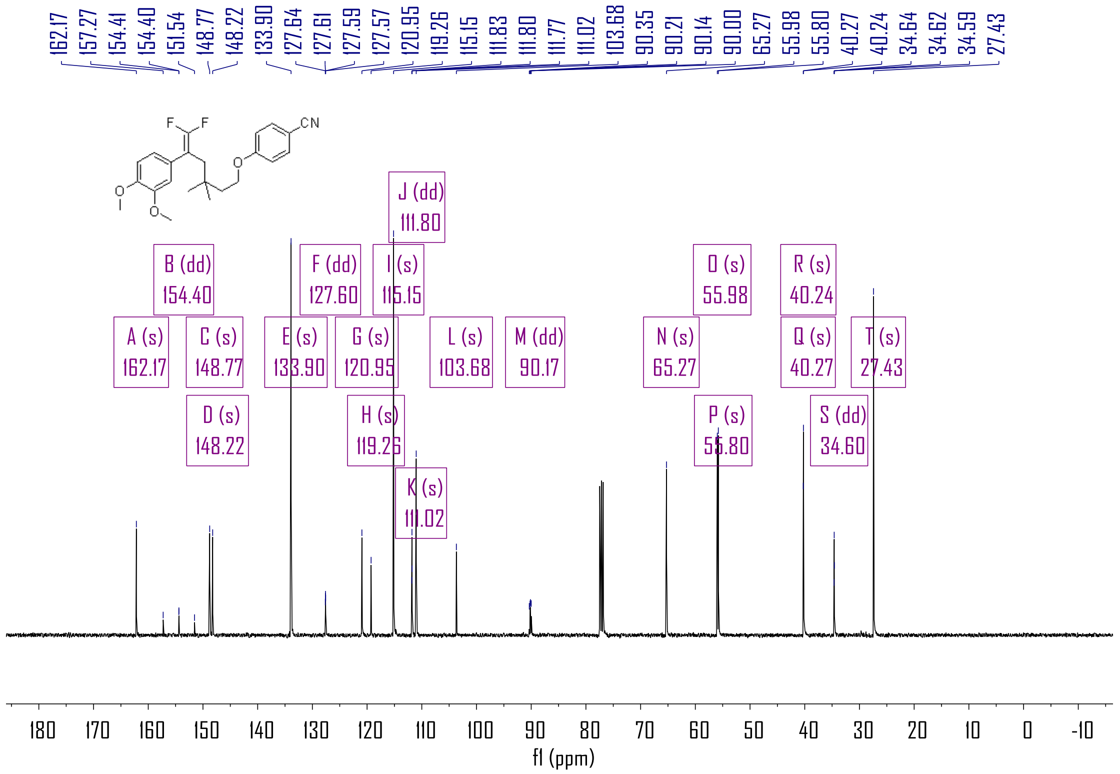

^{13}C NMR spectra for **5bb**.

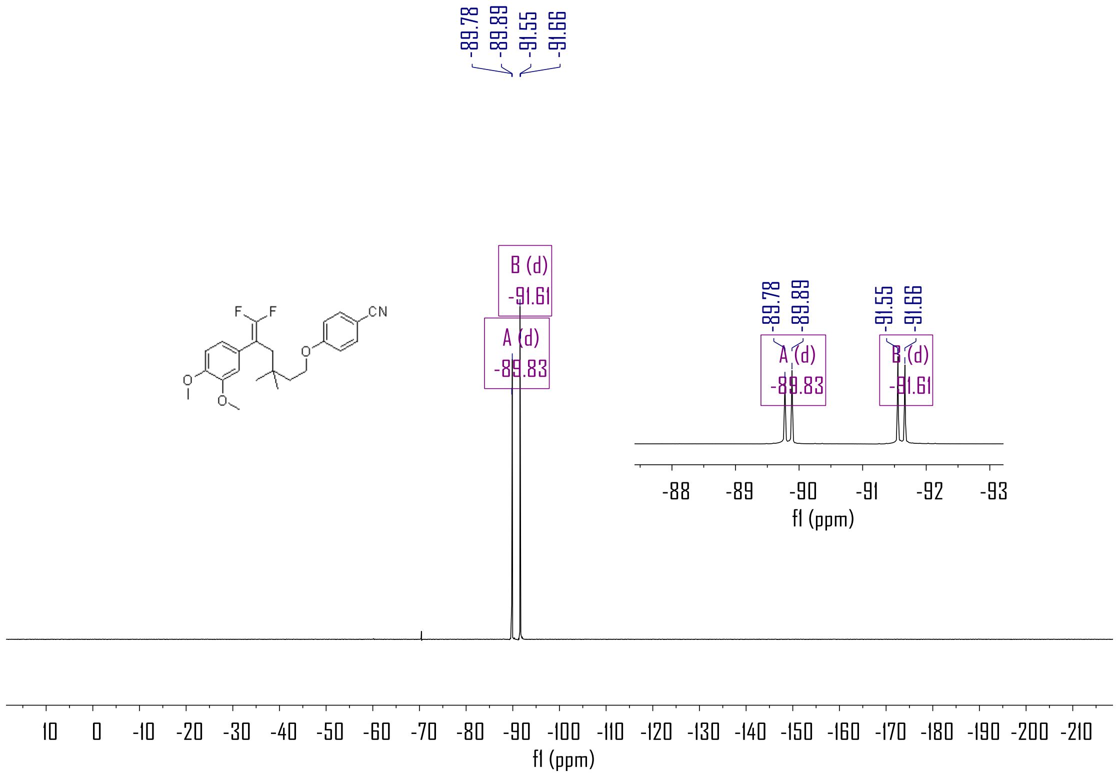

^{19}F NMR spectra for **5bb**.

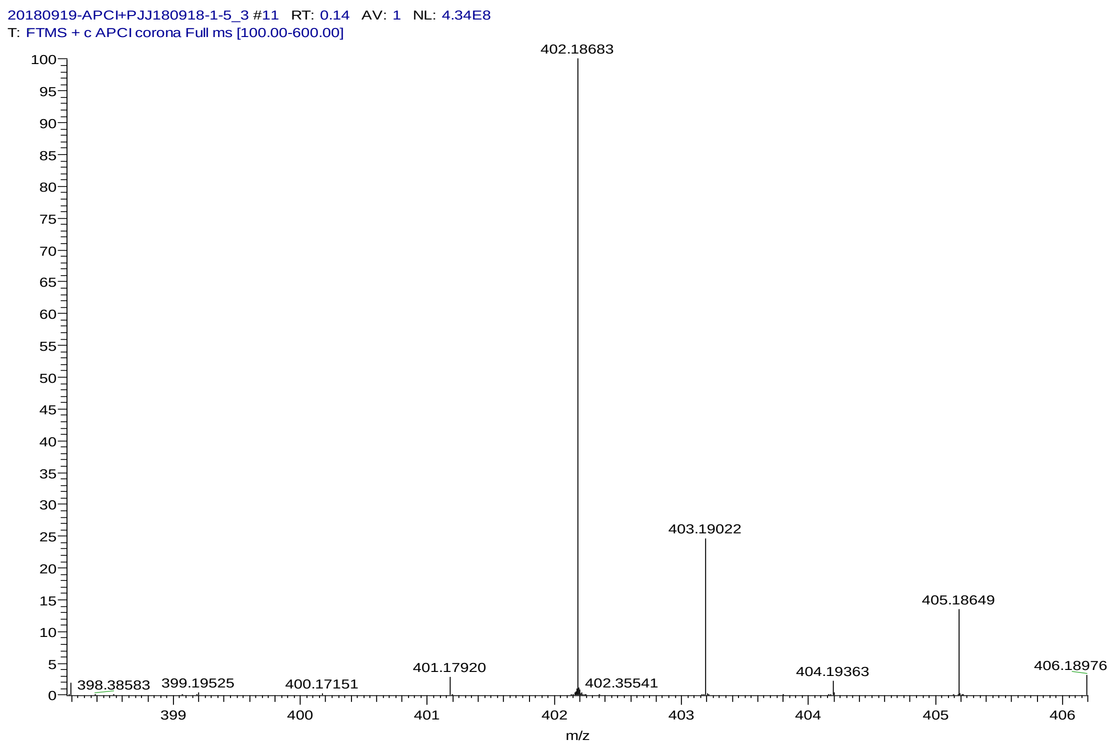

HRMS spectra for **5bb**.

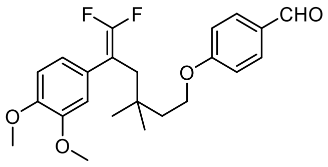

4-((5-(3,4-dimethoxyphenyl)-6,6-difluoro-3,3-dimethylhex-5-en-1-yl)oxy)benzaldehyde (**5bc**)

Following general procedure, **1b** and **4c** were used. The product was isolated by column chromatography as colorless oil (72.6 mg, 0.180 mmol, 90%).

Selectivity (desired C-F cleavage product : addition by-product) > 50:1.

R_f (petroleum ether : ethyl acetate = 10:1) = 0.27.

^{1}H NMR (400 MHz, Chloroform-*d*) δ 9.85 (s, 1H), 7.79 (d, *J* = 8.8 Hz, 2H), 6.90 (d, *J* = 8.7 Hz, 2H), 6.87 – 6.73 (m, 3H), 3.98 (t, *J* = 7.1 Hz, 2H), 3.85 (s, 3H), 3.82 (s, 3H), 2.39 (s, 2H), 1.69 (t, *J* = 7.0 Hz, 2H), 0.88 (s, 6H).

^{13}C NMR (101 MHz, Chloroform-*d*) δ 190.77, 163.98, 154.43 (dd, *J* = 289.5, 288.1 Hz), 148.79, 148.24, 131.95, 129.82, 127.65 (dd, *J* = 4.7, 2.5 Hz), 120.98 (dd, *J* = 2.8, 2.8 Hz), 114.74, 111.81 (dd, *J* = 2.7, 2.7 Hz), 111.04, 90.23 (dd, *J* = 21.4, 13.8 Hz), 65.28, 55.99, 55.81, 40.37, 40.31, 34.66 (dd, *J* = 2.4, 2.4 Hz), 27.46.

^{19}F NMR (376 MHz, Chloroform-*d*) δ -89.84 (d, *J* = 41.9 Hz), -91.64 (d, *J* = 41.9 Hz).

HRMS (APCI) calcd for $C_{23}H_{27}O_4F_2^+$ [(M+H)$^+$] 405.18719, found 405.18692.

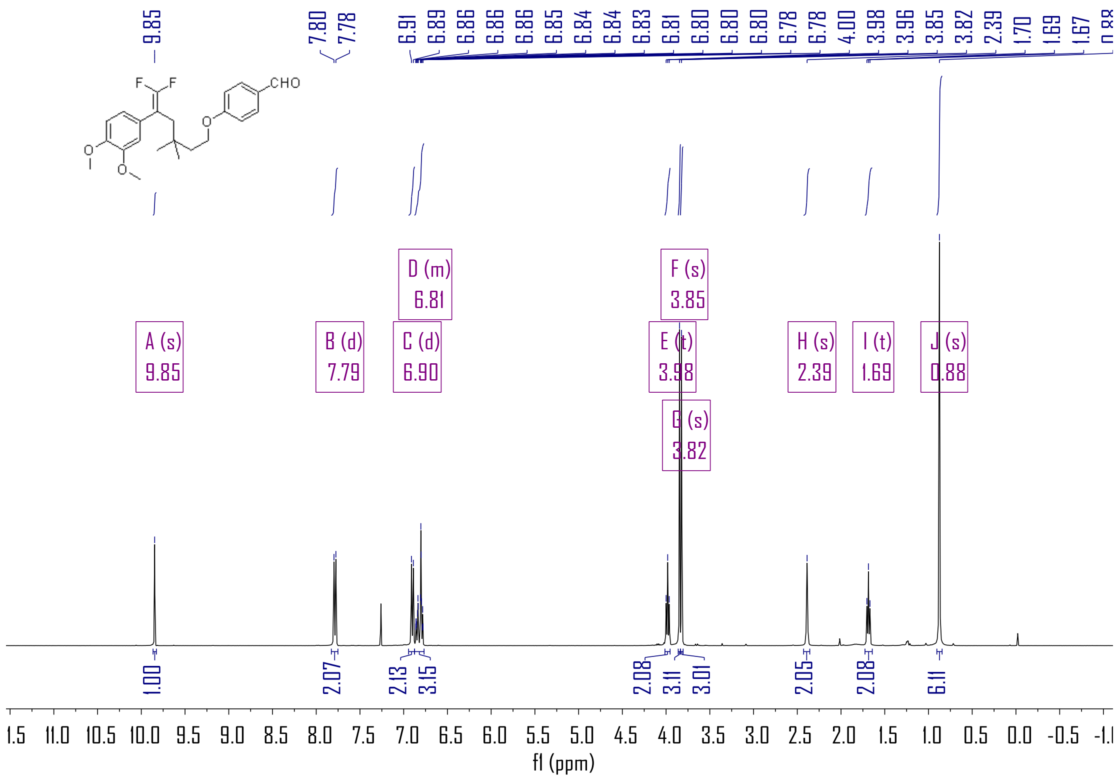

^{1}H NMR spectra for **5bc**.

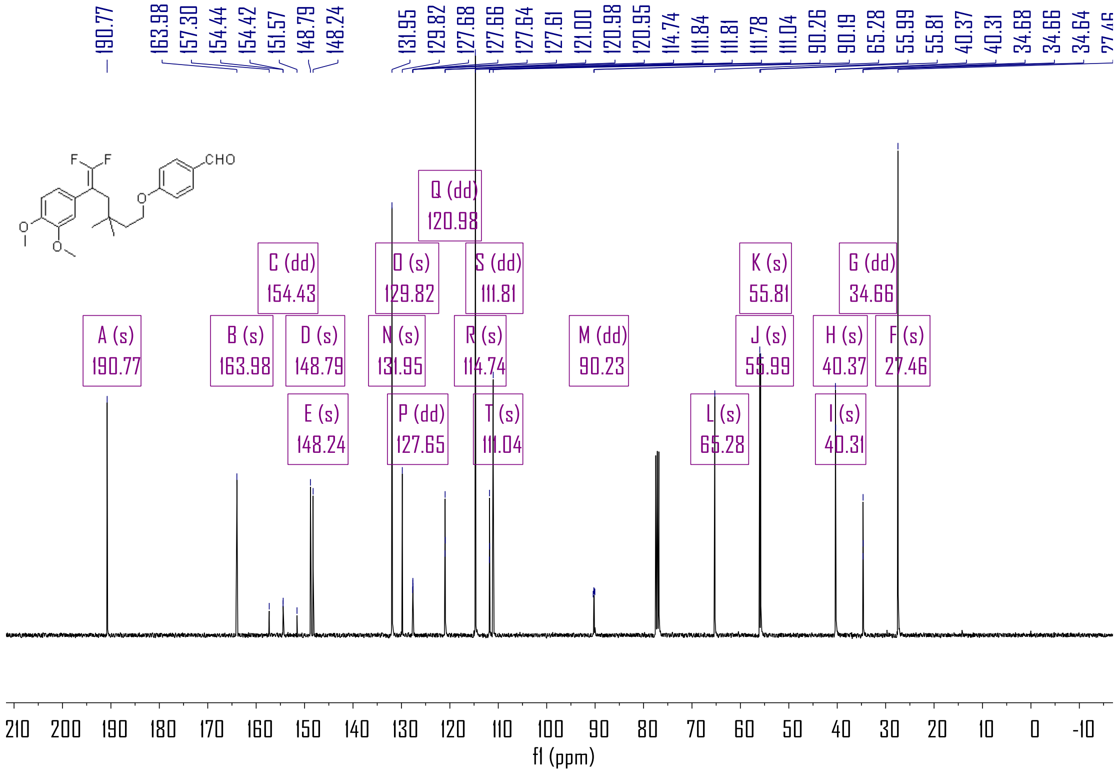

^{13}C NMR spectra for **5bc**.

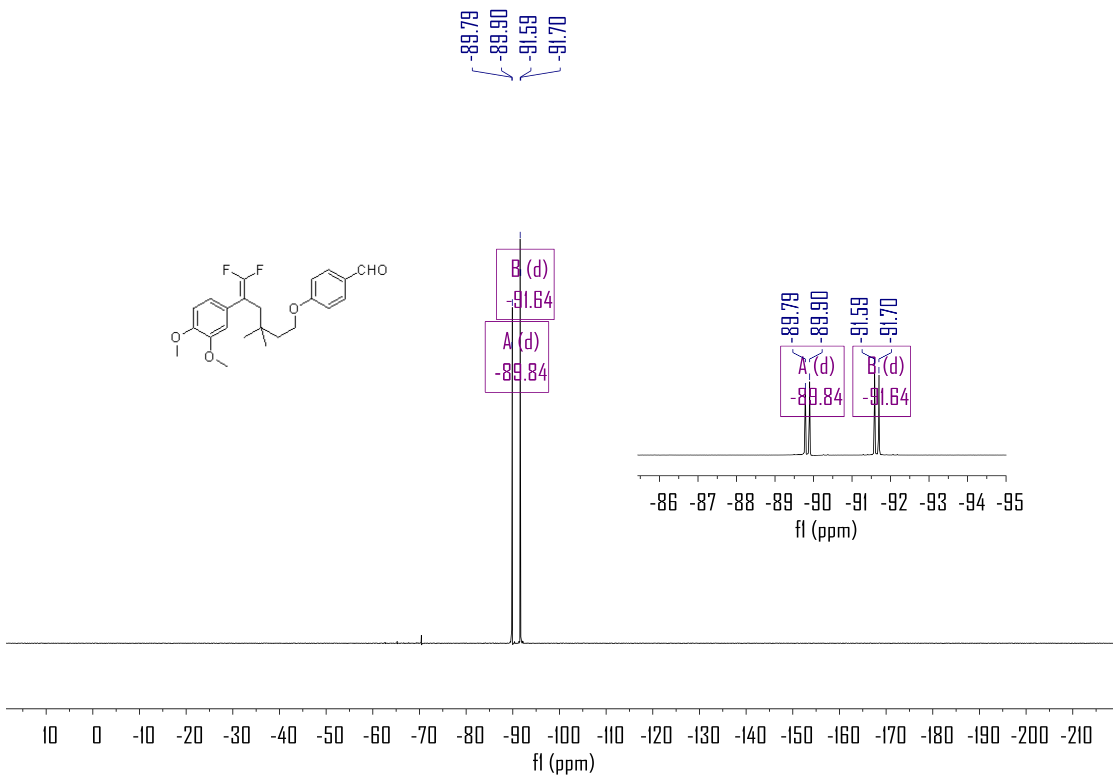

^{19}F NMR spectra for **5bc**.

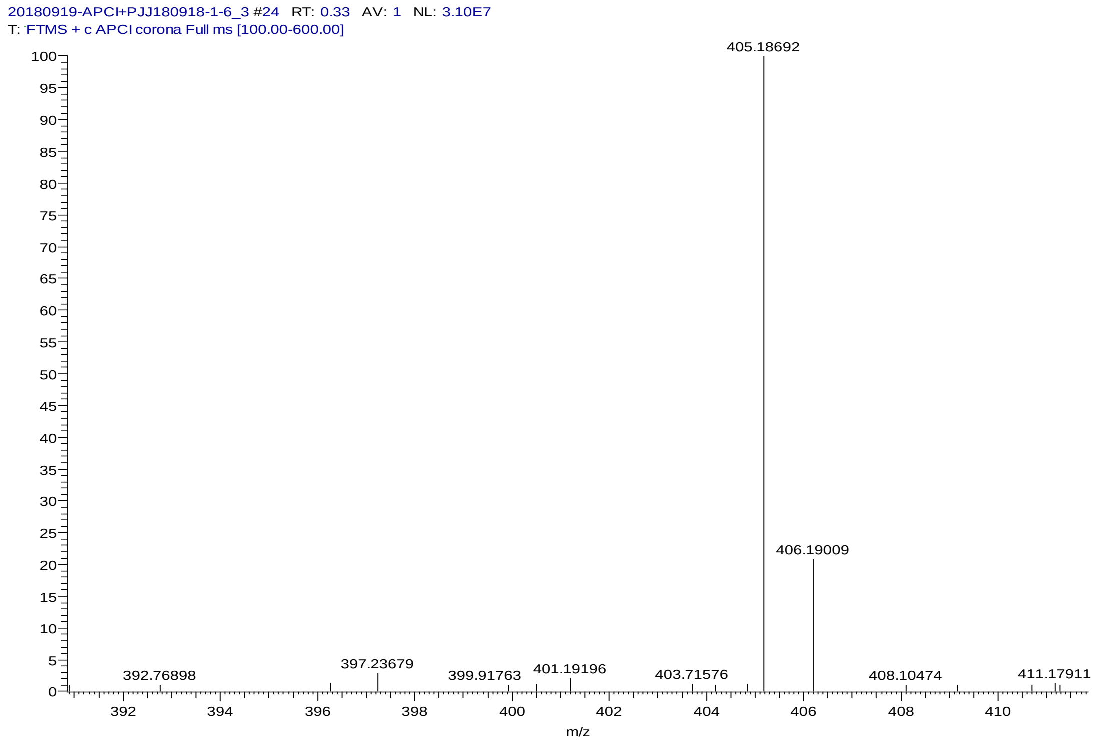

HRMS spectra for **5bc**.

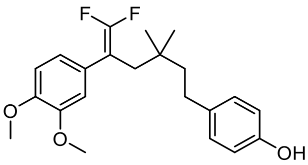

4-(5-(3,4-dimethoxyphenyl)-6,6-difluoro-3,3-dimethylhex-5-en-1-yl)phenol (**5bd**)

Following general procedure, **1b** and **4d** were used. The product was isolated by column chromatography as yellow oil (71.3 mg, 0.189 mmol, 95%).

<u>Selectivity (desired C-F cleavage product : addition by-product)</u> > 50:1.

<u>R_f (petroleum ether : ethyl acetate = 5:1)</u> = 0.31.

<u>^{1}H NMR (400 MHz, Chloroform-*d*)</u> δ 6.93 – 6.82 (m, 5H), 6.78 – 6.69 (m, 2H), 5.73 (brs, 1H), 3.87 (s, 3H), 3.87 (s, 3H), 2.50 – 2.33 (m, 4H), 1.55 – 1.37 (m, 2H), 0.87 (s, 6H).

<u>^{13}C NMR (101 MHz, Chloroform-*d*)</u> δ 154.41 (dd, *J* = 288.1, 288.1 Hz), 153.73, 148.72, 148.13, 134.95, 129.24, 128.22 (dd, *J* = 2.0, 2.0 Hz), 121.23, 115.20, 112.11, 111.20, 90.58 (dd, *J* = 21.5, 13.5 Hz), 56.03, 55.90, 44.66, 39.27, 35.39, 29.74, 27.47.

<u>^{19}F NMR (376 MHz, Chloroform-*d*)</u> δ -90.01 (d, *J* = 42.5 Hz), -91.99 (d, *J* = 42.5 Hz).

<u>HRMS (APCI)</u> calcd for $C_{22}H_{27}O_3F_2^+$ [(M+H)$^+$] 377.19228, found 377.19159.

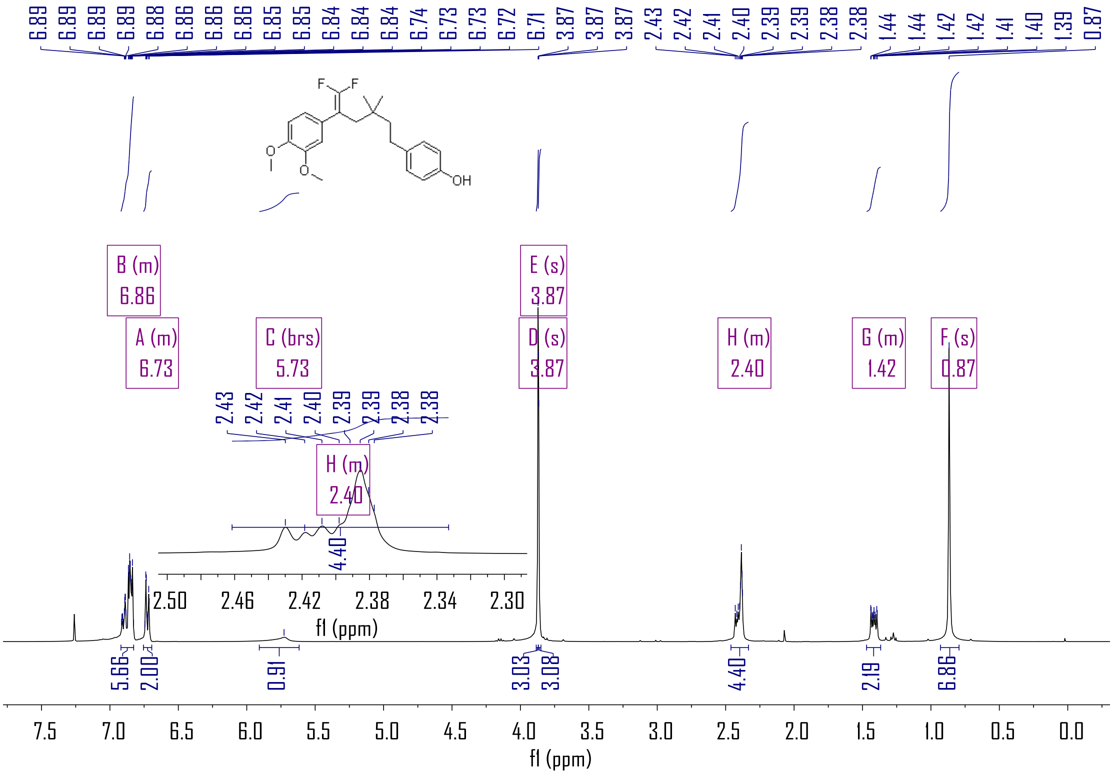

^{1}H NMR spectra for **5bd**.

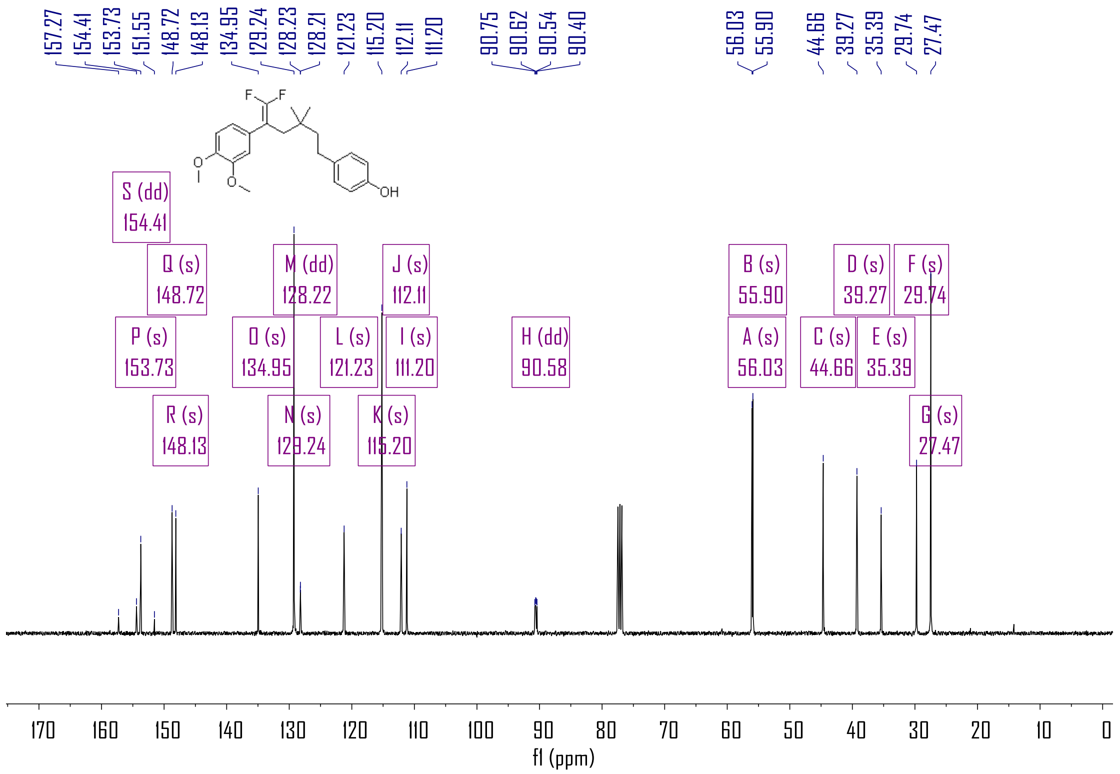

^{13}C NMR spectra for **5bd**.

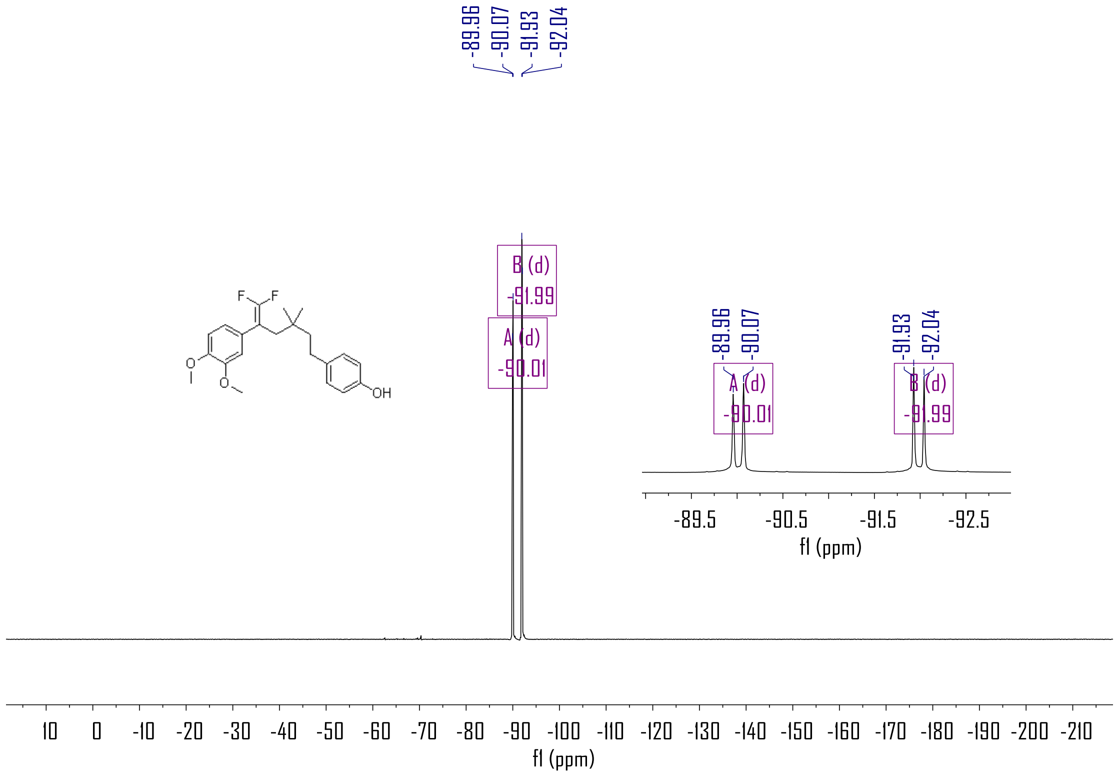

^{19}F NMR spectra for **5bd**.

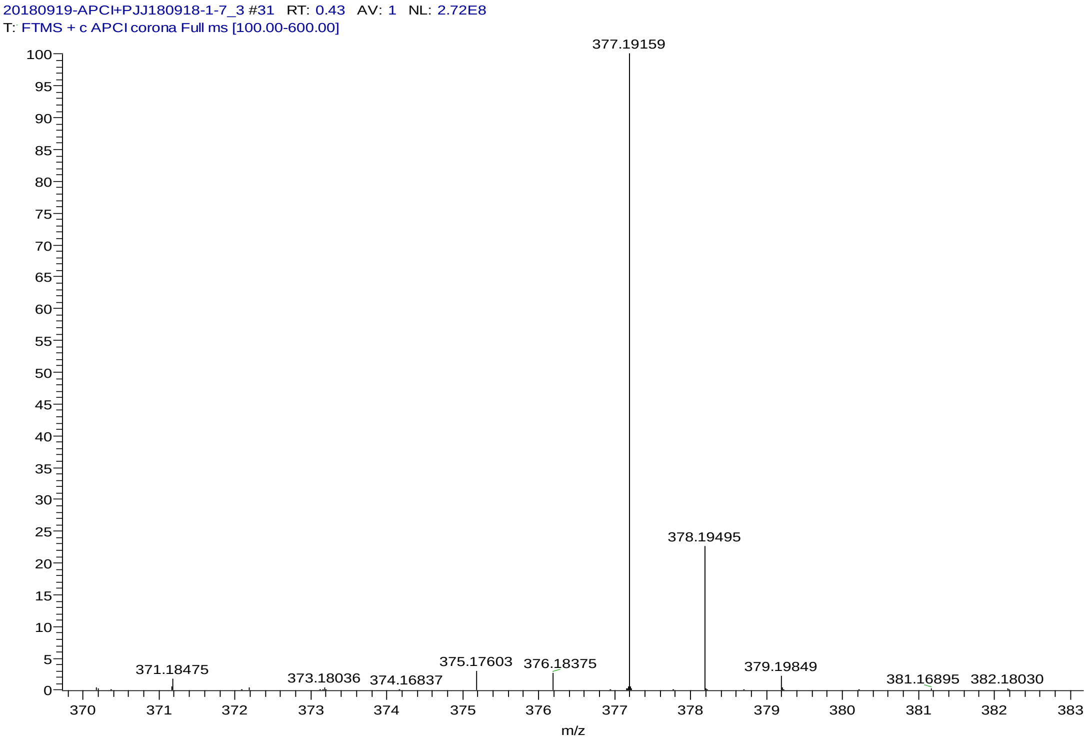

HRMS spectra for **5bd**.

11. General Procedure for Examples Described in Scheme 1

For the synthesis of **5be**, **5bf**, **5ag**, & **5ah**:

NiBr$_2$(diglyme) (0.02 mmol, 10 mol%, 7.1 mg), Pybox (0.03 mmol, 15 mol%, 6.5 mg) and Zn (0.6 mmol, 3.0 equiv., 39 mg) were added to a Schlenk tube equipped with a stir bar. The Schlenk tube was evacuated and filled with argon (three cycles). To these solids, 1 mL DMAc (0.2 M) was added under argon atmosphere. Then, trifluoromethyl alkene (0.2 mmol, 1.0 equiv.) and alkyl bromides (0.3 mmol, 1.5 equiv.) were added and stirred at room temperature (~20 °C) for 16 hours. The mixture was purified by column chromatography to afford the desired product.

For the synthesis of **3uba**, & **3ubb**:

NiBr$_2$(diglyme) (0.02 mmol, 10 mol%, 7.1 mg), Pybox (0.03 mmol, 15 mol%, 6.5 mg) and Zn (0.6 mmol, 3.0 equiv., 39 mg) were added to a Schlenk tube equipped with a stir bar. The Schlenk tube was evacuated and filled with argon (three cycles). To these solids, 1 mL DMSO (0.2 M) was added under argon atmosphere. Then, **1u** (0.2 mmol, 1.0 equiv.) and **2b** (0.3 mmol, 1.5 equiv.) were added and stirred at room temperature (~20 °C) for 16 hours. The mixture was purified by column chromatography to afford the desired product.

12. Examples Described in Scheme 1

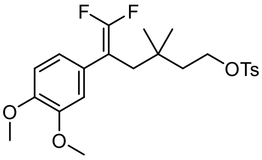

5be

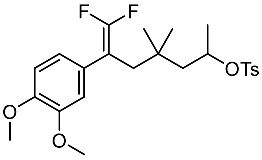

5bf

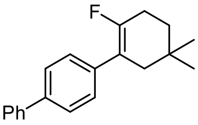

5ag

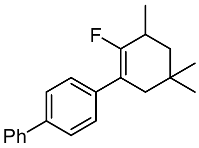

5ah

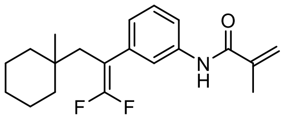

3uba

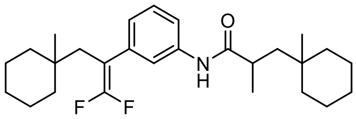

3ubb

List of substrates in Scheme 1.

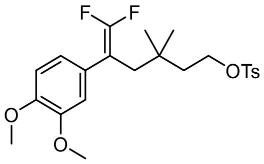

5-(3,4-dimethoxyphenyl)-6,6-difluoro-3,3-dimethylhex-5-en-1-yl 4-methylbenzenesulfonate (**5be**)

Following general procedure, **1b** and **4e** were used. The product was isolated by column chromatography as colorless oil (83.6 mg, 0.184 mmol, 92%).

Selectivity (desired C-F cleavage product : addition by-product) > 50:1.

R_f (petroleum ether : ethyl acetate = 5:1) = 0.33.

^{1}H NMR (400 MHz, Chloroform-*d*) δ 7.73 (d, *J* = 8.3 Hz, 2H), 7.32 (d, *J* = 8.3 Hz, 2H), 6.89 – 6.70 (m, 3H), 3.99 (t, *J* = 7.1 Hz, 2H), 3.85 (s, 3H), 3.85 (s, 3H), 2.42 (s, 3H), 2.27 (s, 2H), 1.52 (t, *J* = 7.1 Hz, 2H), 0.74 (s, 6H).

^{13}C NMR (101 MHz, Chloroform-*d*) δ 154.34 (dd, *J* = 289.4, 288.0 Hz), 148.79, 148.23, 144.76, 133.11, 129.84, 127.80, 127.42 (dd, *J* = 2.6, 2.6 Hz), 120.90 (dd, *J* = 2.8, 2.8 Hz), 111.67 (dd, *J* = 2.9, 2.9 Hz), 111.08, 90.01 (dd, *J* = 21.3, 13.9 Hz), 67.73, 55.98, 55.82, 40.07, 40.04, 34.48 (dd, *J* = 2.7, 2.7 Hz), 27.16, 21.60.

^{19}F NMR (376 MHz, Chloroform-*d*) δ -89.82 (d, *J* = 41.7 Hz), -91.61 (d, *J* = 41.7 Hz).

HRMS (APCI) calcd for $C_{23}H_{29}O_5F_2S^+$ [(M+H)$^+$] 455.16983, found 455.16873.

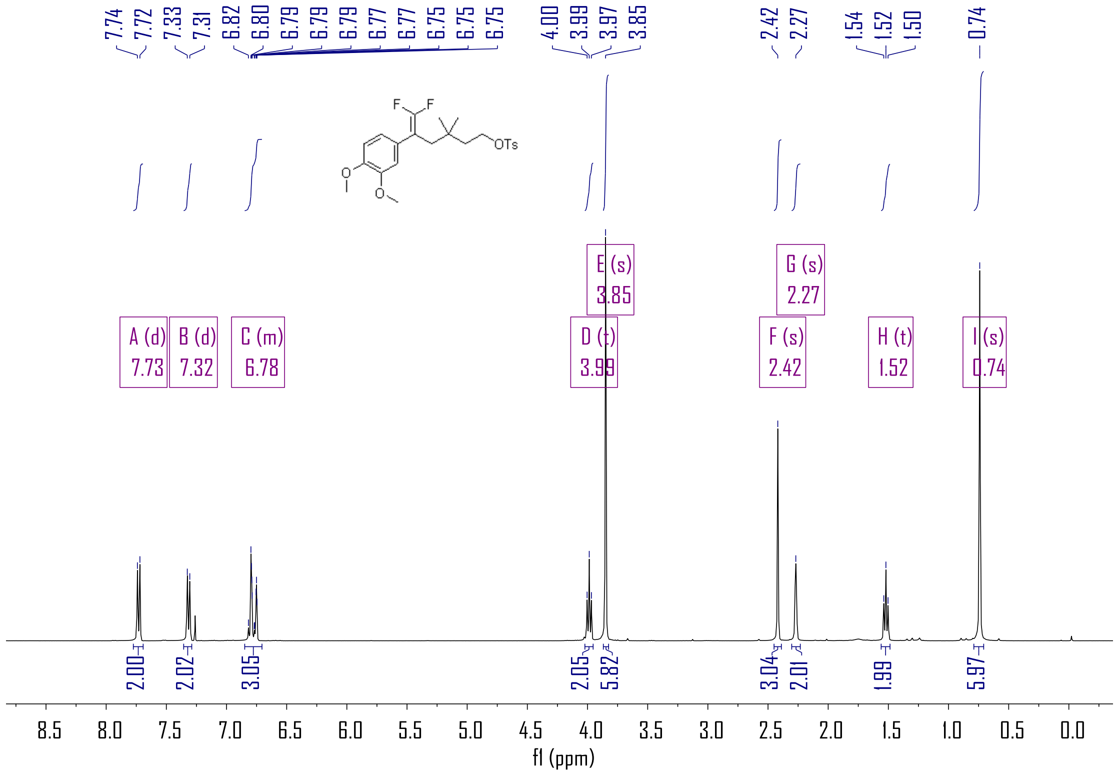

^{1}H NMR spectra for **5be**.

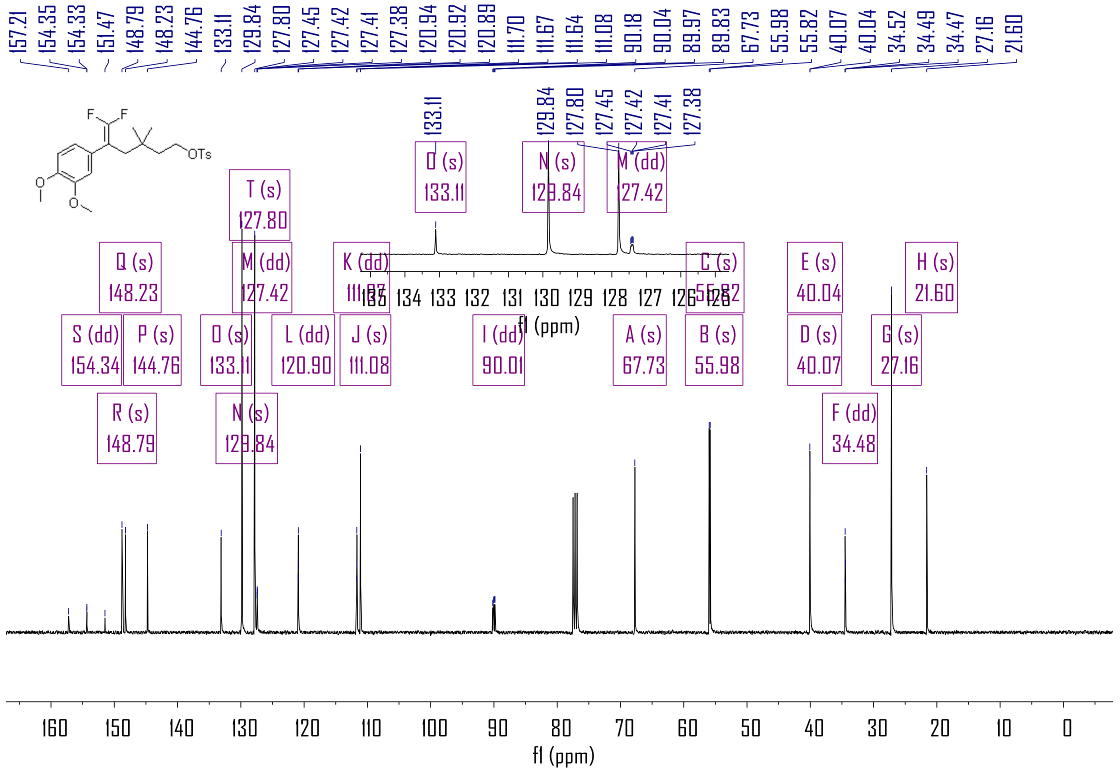

^{13}C NMR spectra for **5be**.

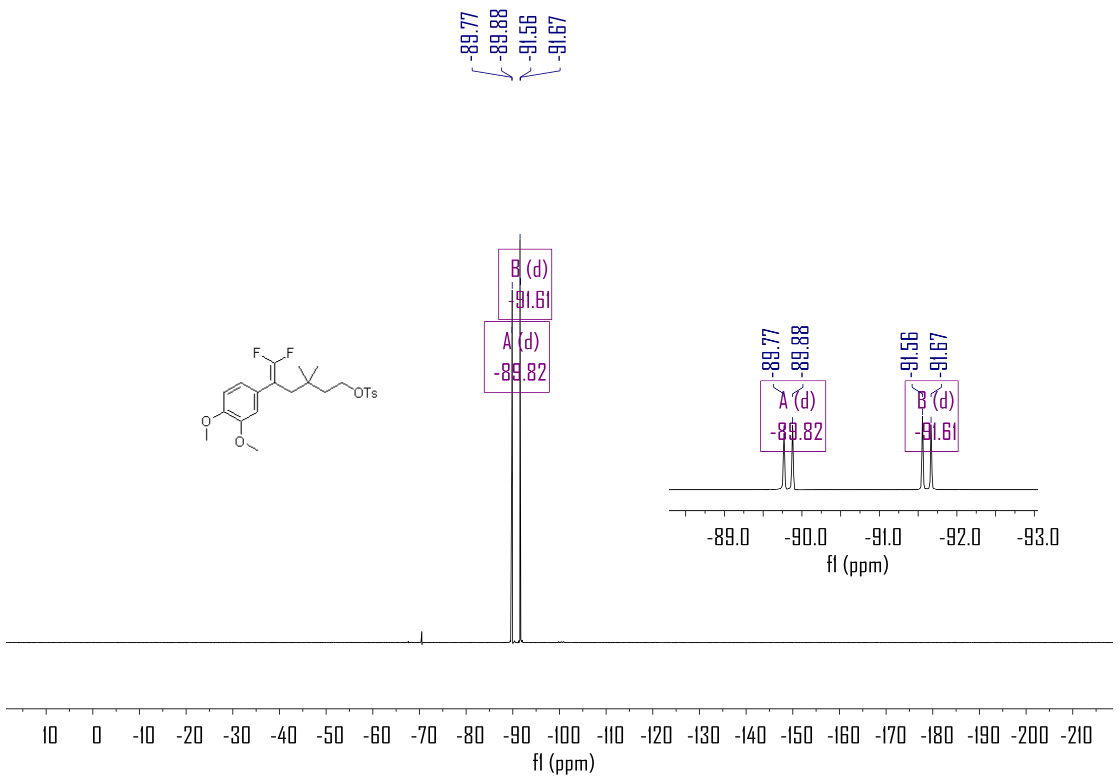

^{19}F NMR spectra for **5be**.

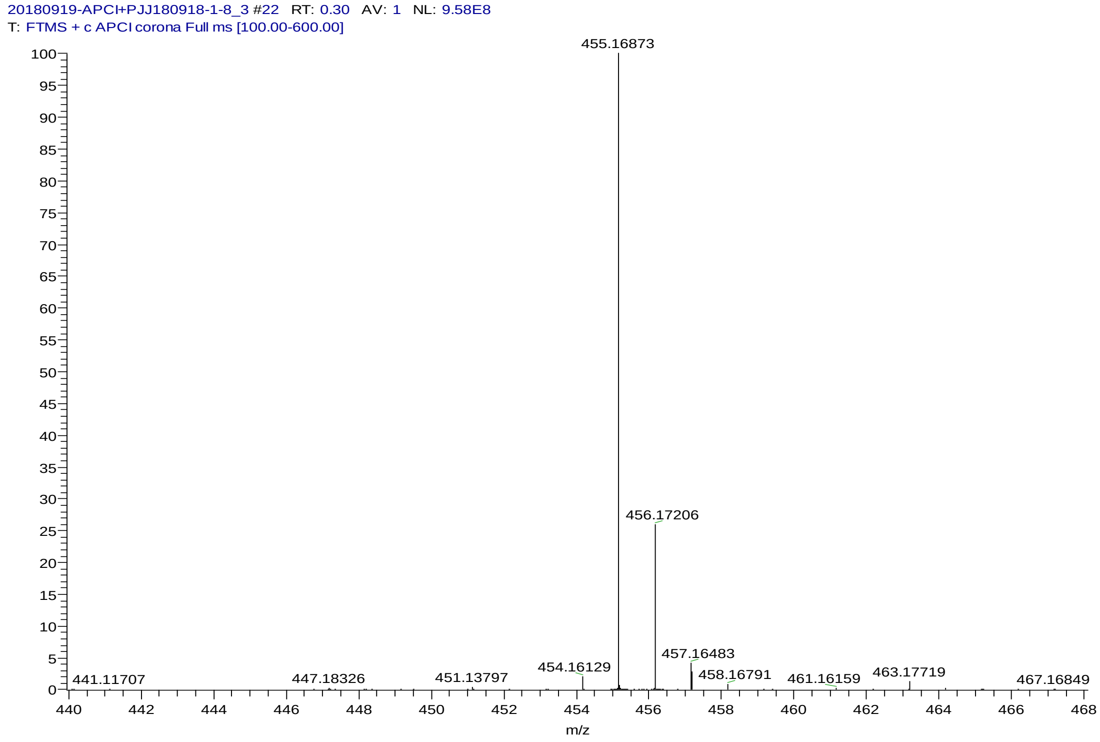

HRMS spectra for **5be**.

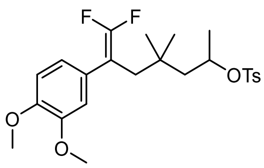

6-(3,4-dimethoxyphenyl)-7,7-difluoro-4,4-dimethylhept-6-en-2-yl 4-methylbenzenesulfonate (**5bf**)

Following general procedure, **1b** and **4f** were used. The product was isolated by column chromatography as colorless oil (72.1 mg, 0.154 mmol, 77%).

<u>Selectivity (desired C-F cleavage product : addition by-product)</u> > 50:1.

<u>R_f (petroleum ether : ethyl acetate = 5:1)</u> = 0.45.

<u>^{1}H NMR (400 MHz, Chloroform-*d*)</u> δ 7.74 (d, *J* = 8.4 Hz, 2H), 7.30 (d, *J* = 8.1 Hz, 2H), 6.88 – 6.77 (m, 3H), 4.83 – 4.71 (m, 1H), 3.88 (s, 3H), 3.86 (s, 3H), 2.41 (s, 3H), 2.37 (t, *J* = 2.7 Hz, 1H), 2.32 – 2.23 (m, 1H), 1.61 (dd, *J* = 15.2, 8.1 Hz, 1H), 1.29 (dd, *J* = 15.1, 3.3 Hz, 1H), 1.03 (d, *J* = 6.2 Hz, 3H), 0.80 (s, 3H), 0.75 (s, 3H).

<u>^{13}C NMR (101 MHz, Chloroform-*d*)</u> δ 154.40 (dd, *J* = 289.4, 288.0 Hz), 148.80, 148.15, 144.54, 135.06, 129.77, 127.68 (dd, *J* = 2.6, 2.6 Hz), 127.61, 121.03 (dd, *J* = 2.8, 2.8 Hz), 111.70 (dd, *J* = 2.9, 2.9 Hz), 110.99, 90.16 (dd, *J* = 21.4, 13.6 Hz), 78.11, 56.03, 55.89, 48.78, 39.77, 34.86 (dd, *J* = 2.6, 2.6 Hz), 27.51, 27.34, 22.83, 21.66.

<u>^{19}F NMR (376 MHz, Chloroform-*d*)</u> δ -89.80 (d, *J* = 41.6 Hz), -91.84 (d, *J* = 41.6 Hz).

<u>HRMS (APCI)</u> calcd for $C_{24}H_{31}O_5F_2S^+$ [(M+H)$^+$] 469.18548, found 469.18506.

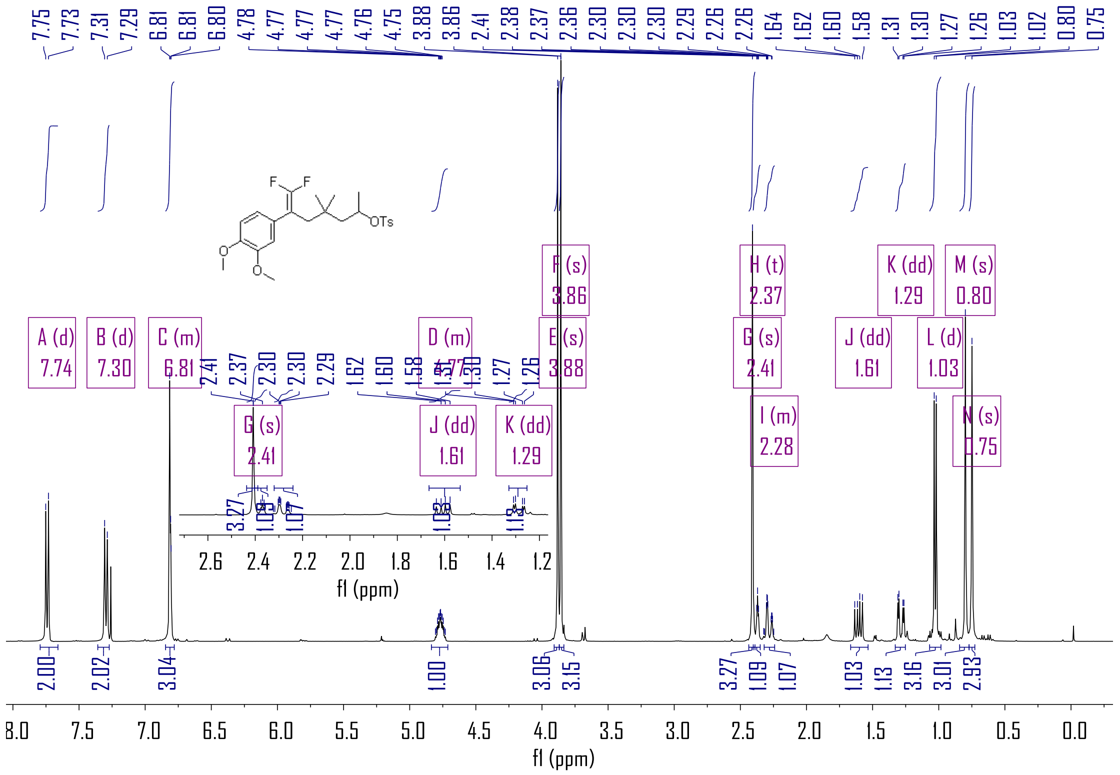

^{1}H NMR spectra for **5bf**.

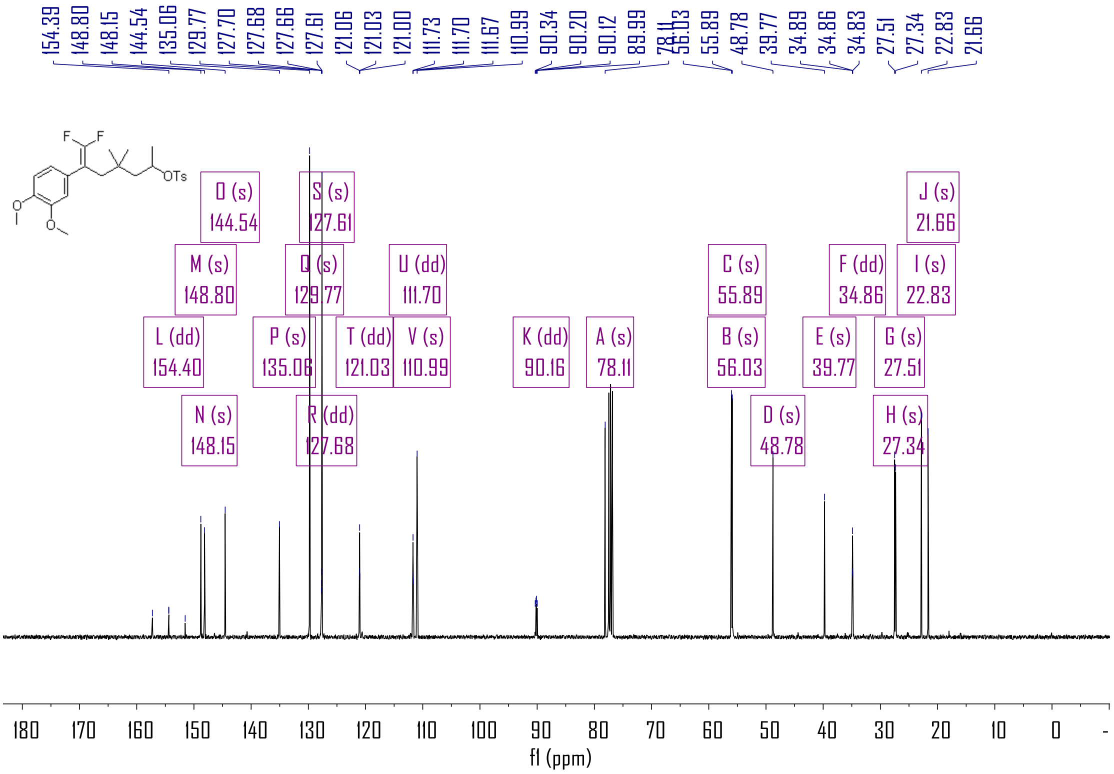

^{13}C NMR spectra for **5bf**.

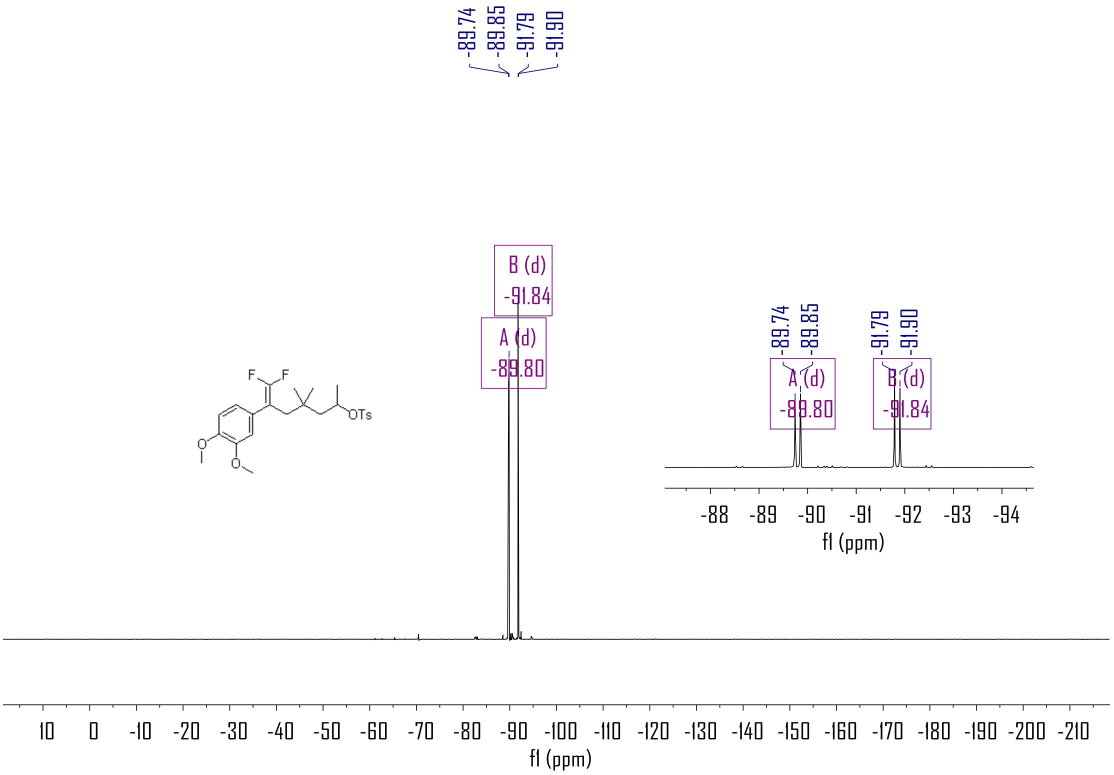

^{19}F NMR spectra for **5bf**.

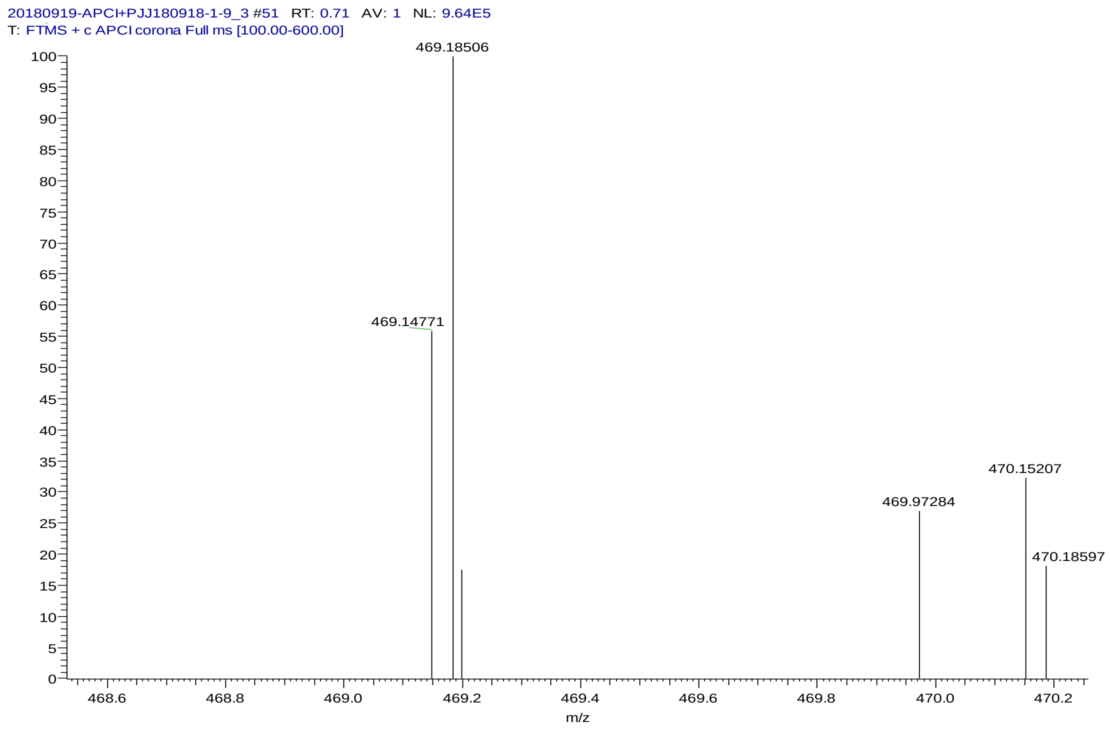

HRMS spectra for **5bf**.

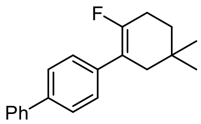

6-fluoro-3,3-dimethyl-2,3,4,5-tetrahydro-1,1':4',1''-terphenyl (**5ag**)

Following general procedure, **1a** and **4g** were used. The product was isolated by column chromatography as colorless oil (44.2 mg, 0.158 mmol, 79%).

R_f (petroleum ether) = 0.70.

^{1}H NMR (400 MHz, Chloroform-*d*) δ 7.66 – 7.58 (m, 4H), 7.54 – 7.43 (m, 4H), 7.39 – 7.34 (m, 1H), 2.51 – 2.37 (m, 2H), 2.32 – 2.22 (m, 2H), 1.71 – 1.55 (m, 2H), 1.08 (s, 6H).

^{13}C NMR (101 MHz, Chloroform-*d*) δ 155.07 (d, *J* = 258.6 Hz), 141.06, 139.47, 136.80, 128.87, 128.21 (d, *J* = 4.5 Hz), 127.29, 127.14, 126.88, 111.73 (d, *J* = 7.1 Hz), 42.21 (d, *J* = 4.5 Hz), 35.44 (d, *J* = 9.6 Hz), 29.76 (d, *J* = 1.4 Hz), 27.95, 24.44 (d, *J* = 24.3 Hz).

^{19}F NMR (376 MHz, Chloroform-*d*) δ -106.68.

HRMS (EI) calcd for $C_{20}H_{21}F^+$ [M^+] 280.16218, found 280.16217.

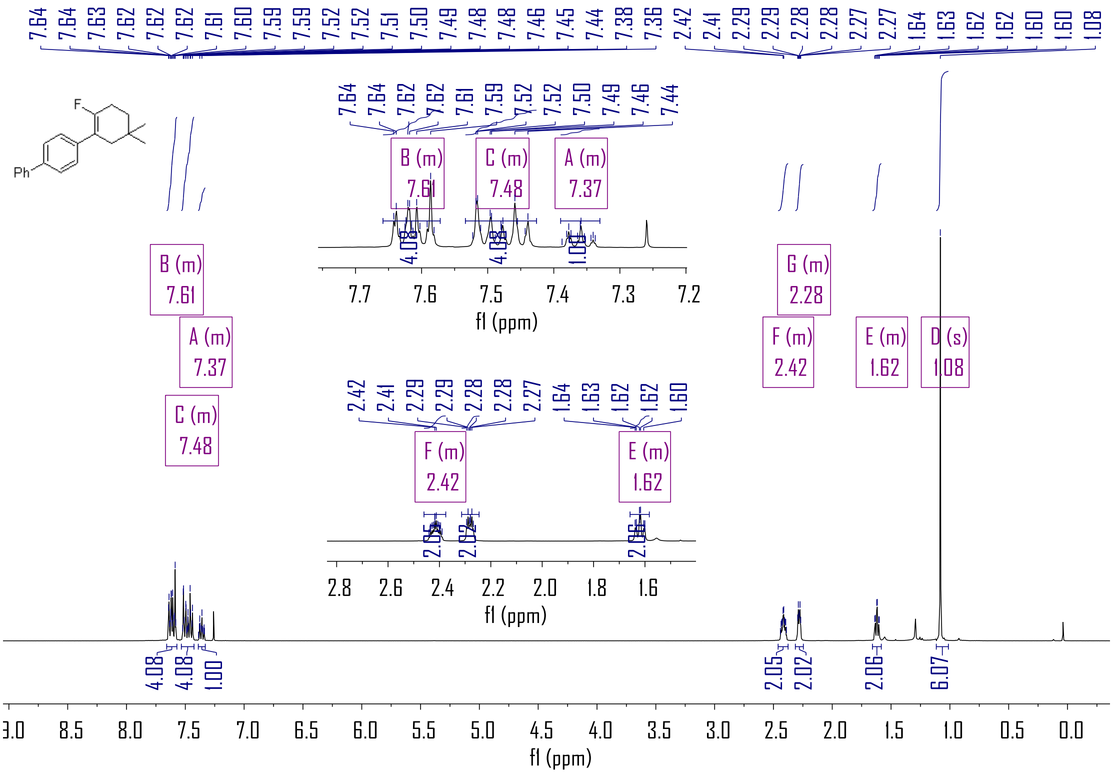

^{1}H NMR spectra for **5ag**.

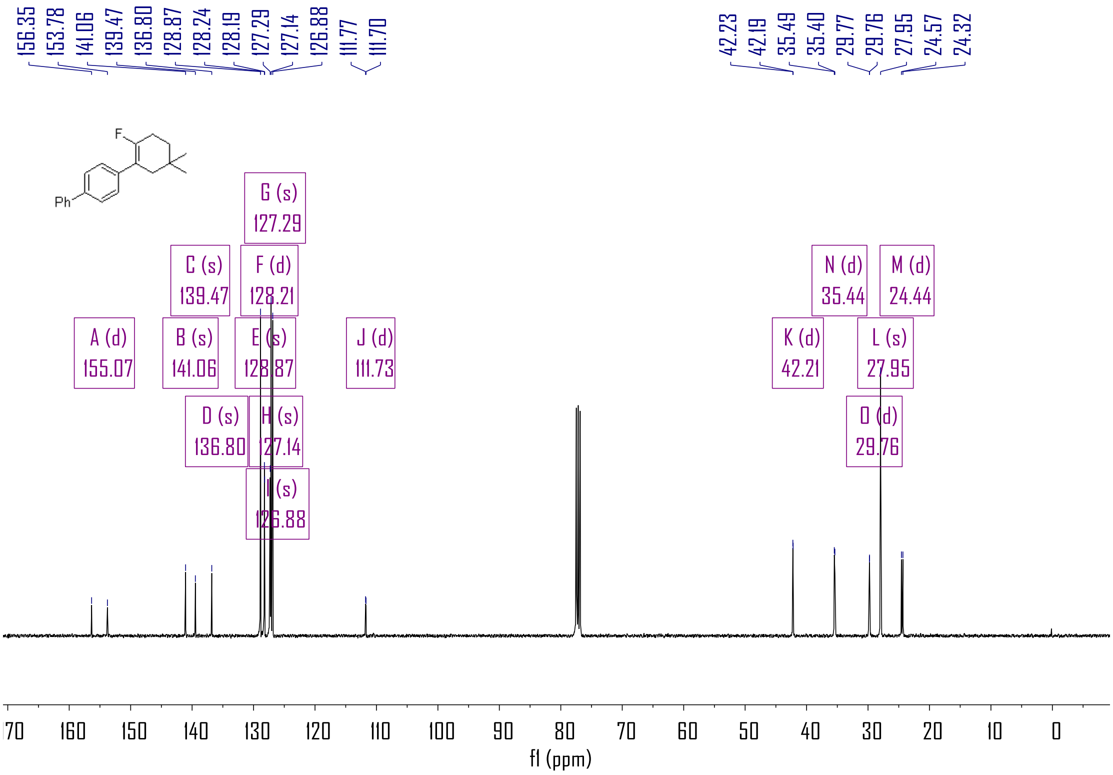

^{13}C NMR spectra for **5ag**.

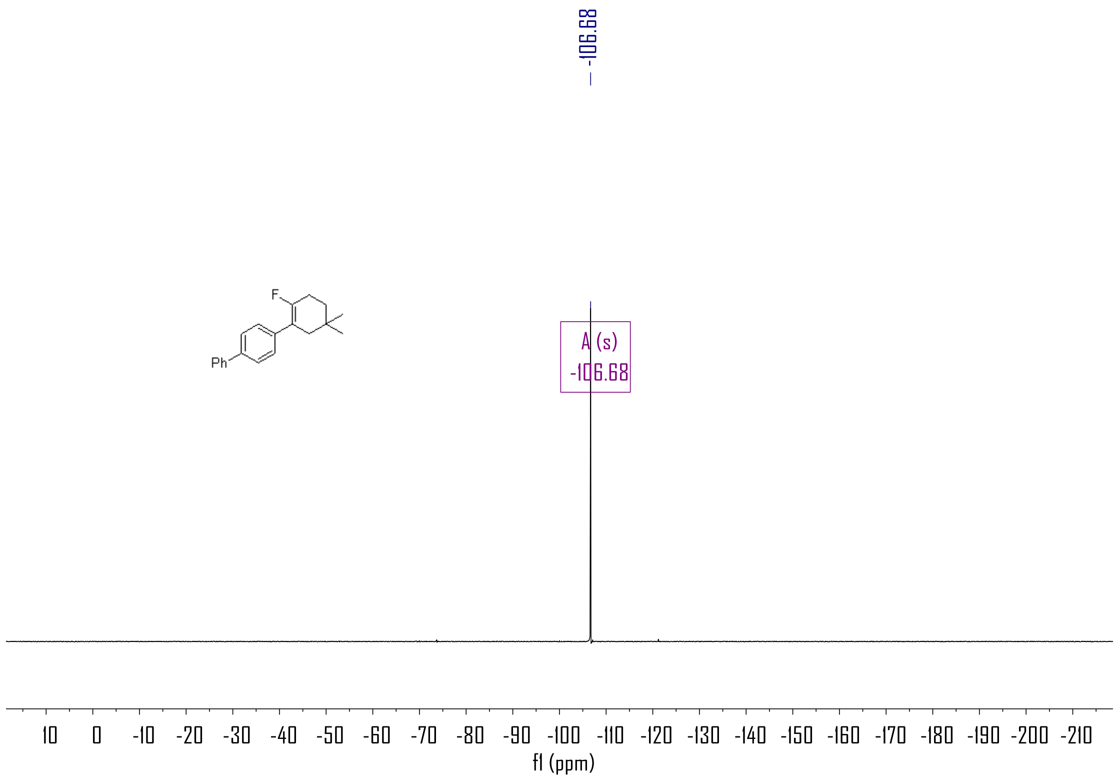

^{19}F NMR spectra for **5ag**.

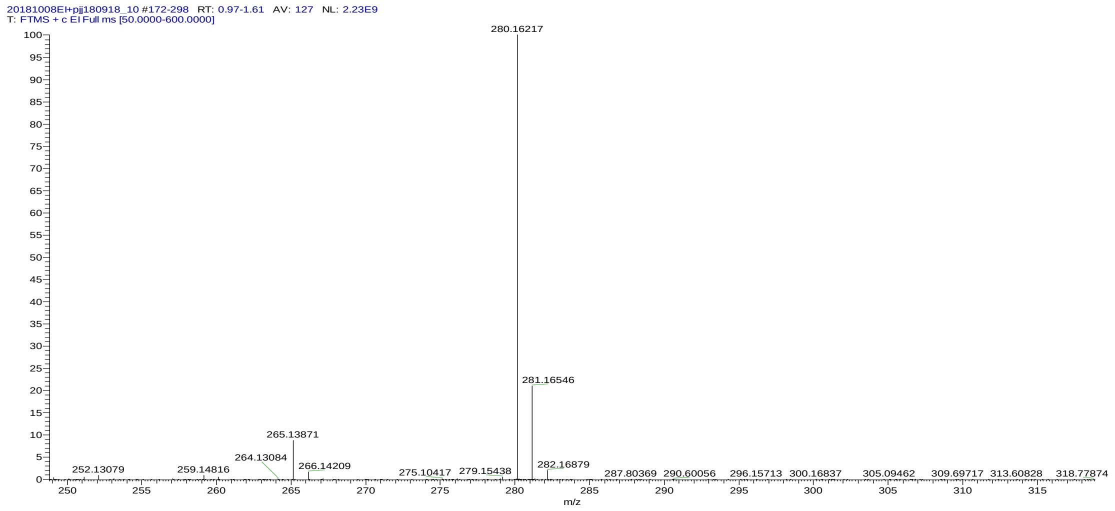

HRMS spectra for **5ag**.

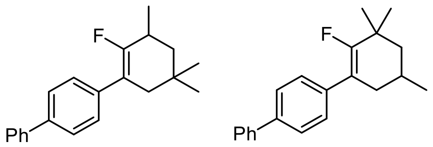

5ah **5ah'**

6-fluoro-3,3,5-trimethyl-2,3,4,5-tetrahydro-1,1':4',1''-terphenyl (**5ah**)

6-fluoro-3,5,5-trimethyl-2,3,4,5-tetrahydro-1,1':4',1''-terphenyl (**5ah'**)

Following general procedure, **1a** and **4h** were used. The products were isolated by

column chromatography as colorless oil (47.6 mg, 0.162 mmol, 81%). The yield of **5ah** was 75%, the yield of **5ah'** was 6%, which was determined by ^{19}F NMR.

R$_f$ (petroleum ether) = 0.70.

^{1}H NMR (400 MHz, Chloroform-*d*) δ 7.68 – 7.56 (m, 4H), 7.54 – 7.42 (m, 4H), 7.41 – 7.31 (m, 1H), 2.69 (ddtq, *J* = 10.6, 6.3, 4.0, 2.0 Hz, 1H), 2.46 (ddd, *J* = 16.1, 6.3, 3.8 Hz, 1H), 2.13 (dddd, *J* = 16.1, 4.6, 2.5, 1.7 Hz, 1H), 1.68 (dtd, *J* = 12.9, 6.0, 2.6 Hz, 1H), 1.37 (dd, *J* = 13.0, 10.8 Hz, 1H), 1.21 (dd, *J* = 6.8, 0.8 Hz, 3H), 1.08 (s, 3H), 1.08 (s, 3H).

^{13}C NMR (101 MHz, Chloroform-*d*) δ 158.05 (d, *J* = 260.8 Hz), 141.08, 139.48, 136.98, 128.87, 128.37 (d, *J* = 4.6 Hz), 127.29, 127.15, 126.85 , 111.72 (d, *J* = 8.5 Hz), 45.65 (d, *J* = 7.2 Hz), 42.89 (d, *J* = 4.3 Hz), 31.31 (d, *J* = 1.8 Hz), 30.18 (d, *J* = 1.7 Hz), 29.79 (d, *J* = 23.0 Hz), 25.23, 17.68 (d, *J* = 4.1 Hz).

^{19}F NMR (376 MHz, Chloroform-*d*) (**5ah**) δ -116.93 (d, *J* = 6.1 Hz).

^{19}F NMR (376 MHz, Chloroform-*d*) (**5ah'**) δ -123.14 (q, *J* = 5.8 Hz).

HRMS (EI) calcd for $C_{21}H_{23}F^+$ [M$^+$] 294.17783, found 294.17745.

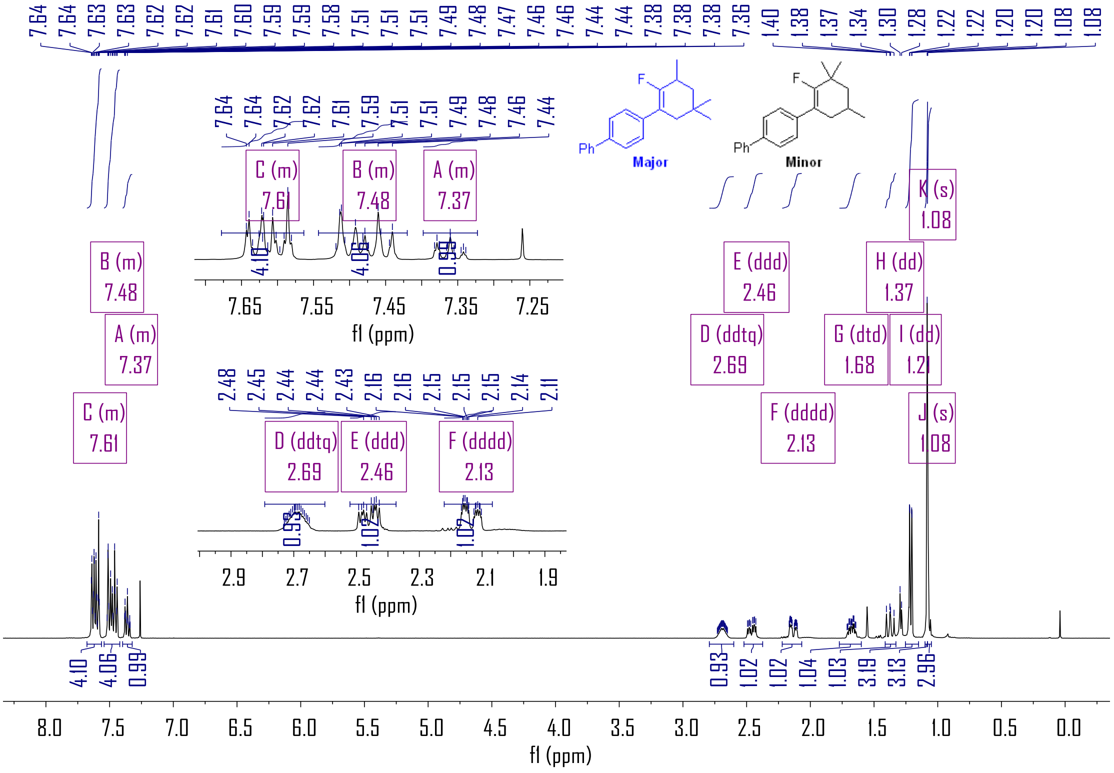

^{1}H NMR spectra for **5ah** & **5ah'**.

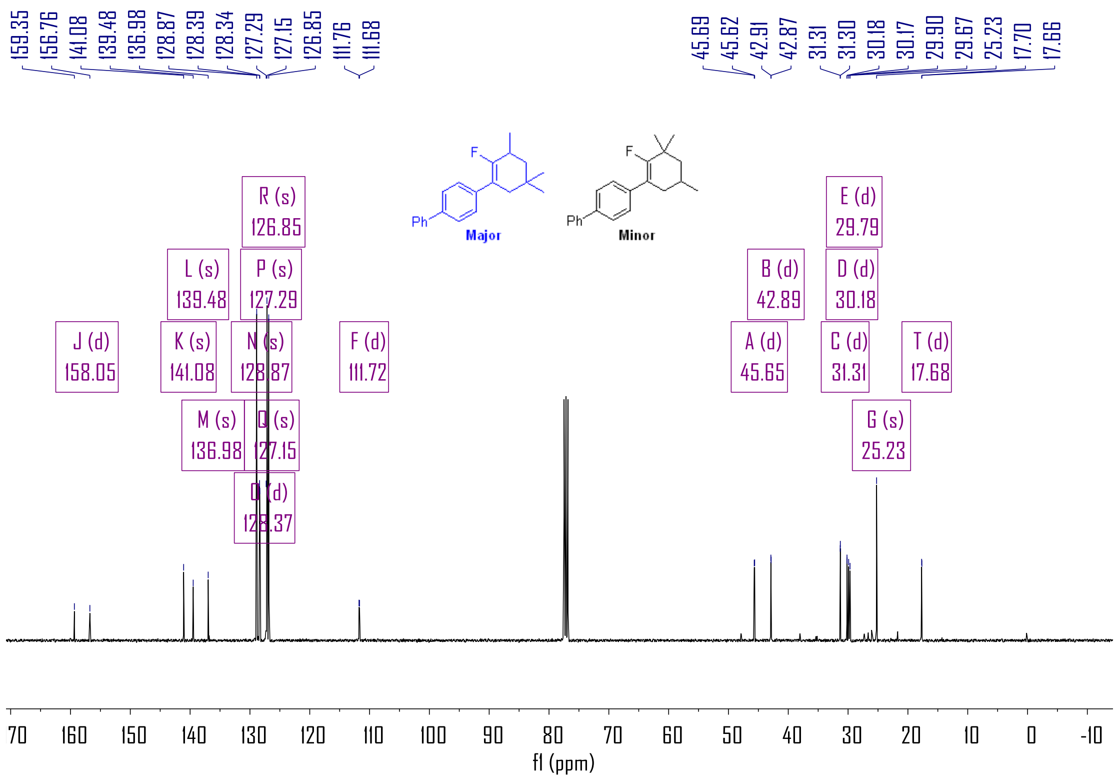

^{13}C NMR spectra for **5ah** & **5ah'**.

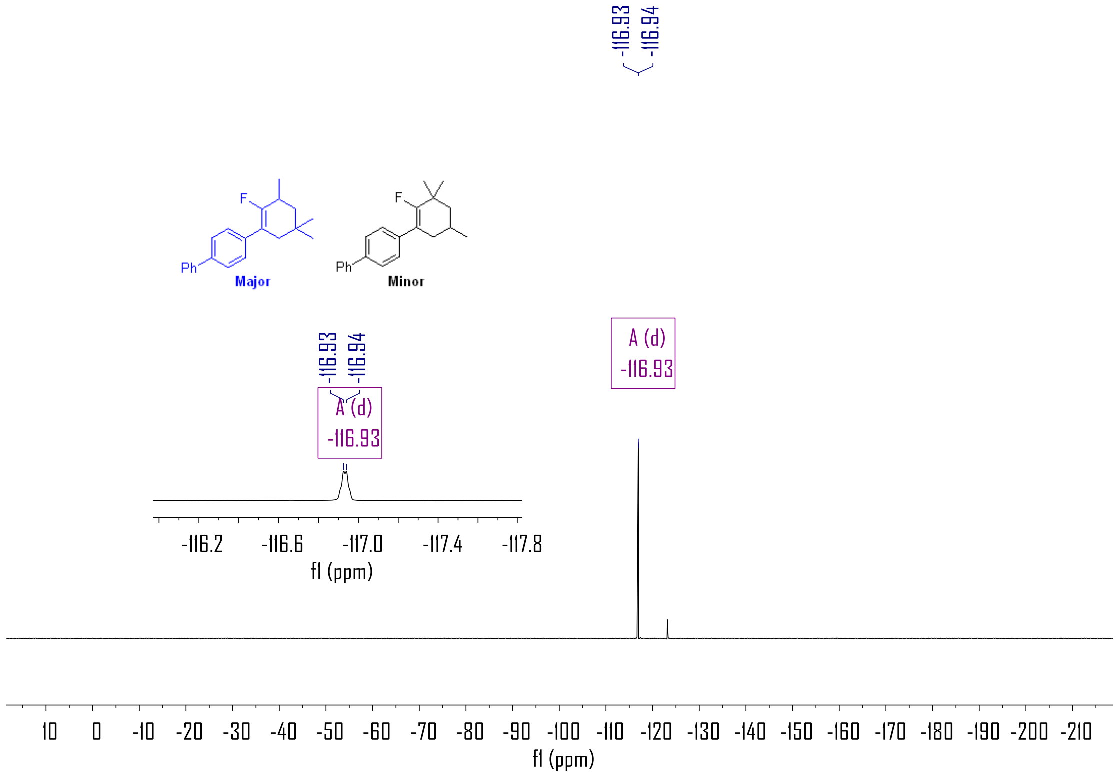

^{19}F NMR spectra for **5ah**.

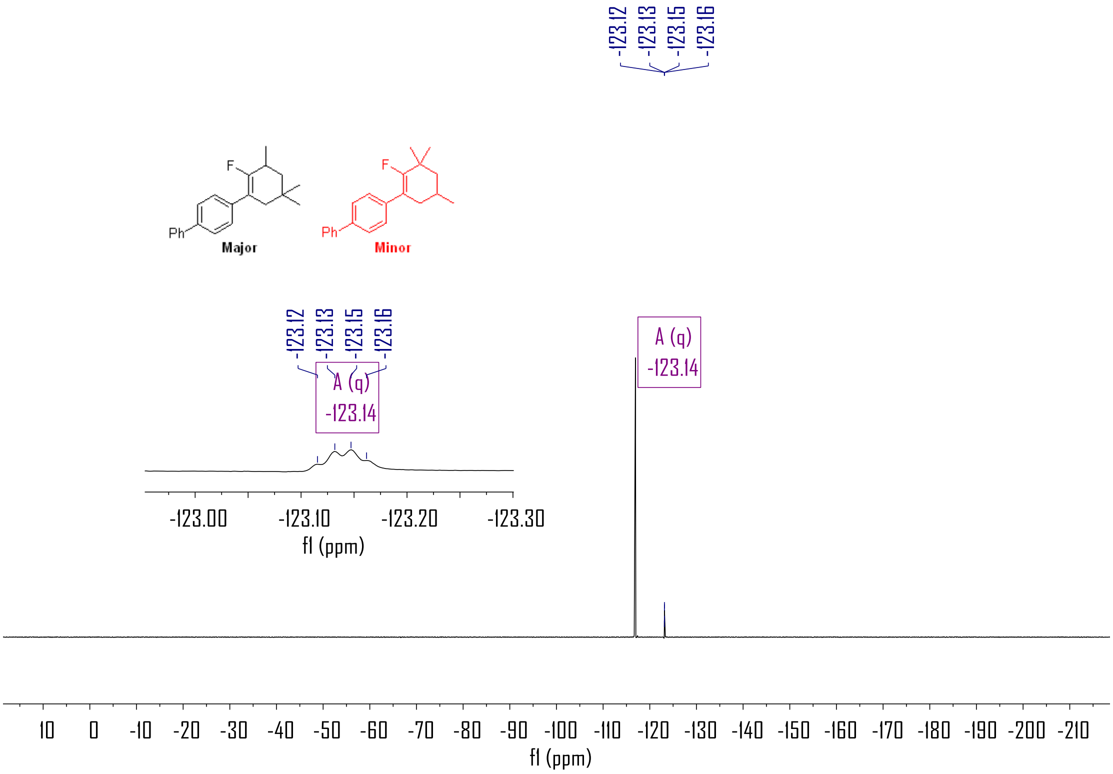

^{19}F NMR spectra for **5ah'**.

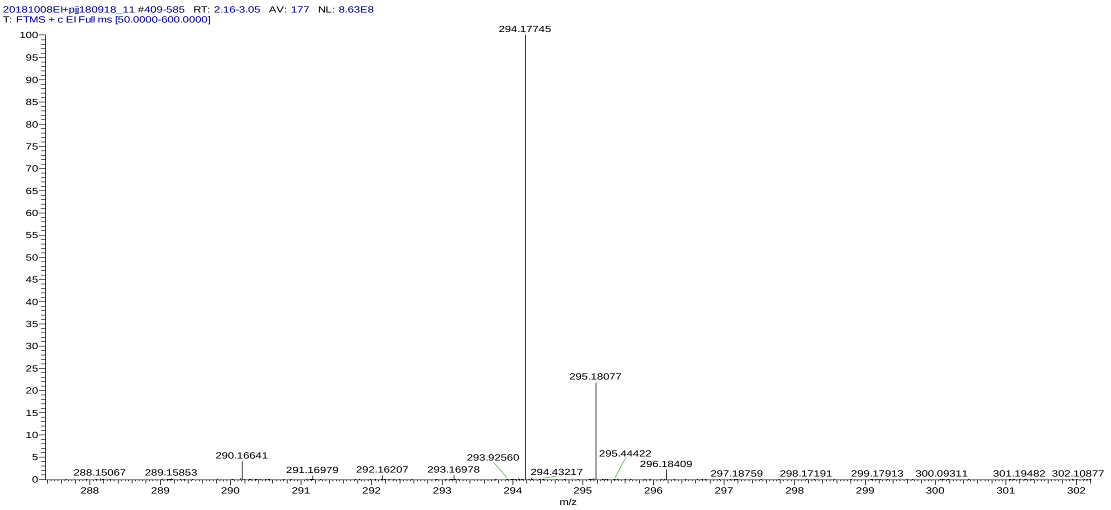

HRMS spectra for **5ah**.

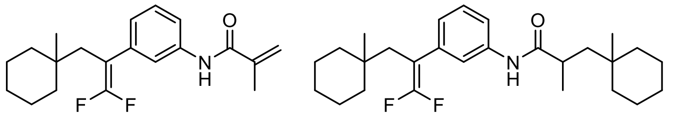

3uba **3ubb**

Following general procedure, **1u** and **2b** were used. The product **3uba** was isolated by column chromatography as white solid (40.2 mg, 0.120 mmol, 60%), the product **3ubb** was isolated by column chromatography as colorless oil (12.6 mg, 0.029 mmol, 14%).

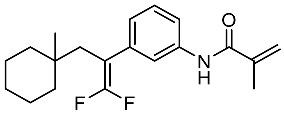

N-(3-(1,1-difluoro-3-(1-methylcyclohexyl)prop-1-en-2-yl)phenyl)methacrylamide (**3uba**)

Selectivity (desired C-F cleavage product : addition by-product) > 50:1.

R_f (petroleum ether : ethyl acetate = 5:1) = 0.40.

^{1}H NMR (400 MHz, Chloroform-*d*) δ 7.63 (brs, 1H), 7.55 (q, *J* = 1.7 Hz, 1H), 7.47 (ddd, *J* = 8.1, 2.3, 1.0 Hz, 1H), 7.29 – 7.24 (m, 1H), 7.07 (dq, *J* = 7.8, 1.4 Hz, 1H), 5.80 (s, 1H), 5.46 (dd, *J* = 1.7, 0.7 Hz, 1H), 2.36 – 2.31 (m, 2H), 2.08 – 2.04 (m, 3H), 1.48 – 1.12 (m, 10H), 0.76 (s, 3H).

^{13}C NMR (101 MHz, Chloroform-*d*) δ 166.79, 154.45 (dd, *J* = 290.3, 287.5 Hz), 140.92, 137.91, 136.89 (dd, *J* = 4.9, 2.6 Hz), 128.91, 124.68 (dd, *J* = 2.8, 2.8 Hz), 120.16, 120.07, 118.74, 90.49 (dd, *J* = 22.1, 12.7 Hz), 40.46, 38.04, 35.30, 26.36, 24.63, 22.07, 18.85.

^{19}F NMR (376 MHz, Chloroform-*d*) δ -89.04 (d, *J* = 40.1 Hz), -91.44 (d, *J* = 40.2 Hz).

HRMS (ESI) calcd for $C_{20}H_{26}ONF_2^+$ [(M+H)$^+$] 334.19770, found 334.19733.

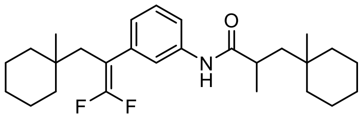

N-(3-(1,1-difluoro-3-(1-methylcyclohexyl)prop-1-en-2-yl)phenyl)-2-methyl-3-(1-methylcyclohexyl)propanamide (**3ubb**)

Selectivity (desired C-F cleavage product : addition by-product) > 50:1.

R_f (petroleum ether : ethyl acetate = 5:1) = 0.55.

^{1}H NMR (400 MHz, Chloroform-*d*) δ 7.50 – 7.46 (m, 1H), 7.46 – 7.41 (m, 1H), 7.29 – 7.23 (m, 2H), 7.11 – 7.01 (m, 1H), 2.51 – 2.38 (m, 1H), 2.35 – 2.29 (m, 2H), 2.07 – 1.95 (m, 1H), 1.55 – 1.10 (m, 24H), 0.90 (s, 3H), 0.75 (s, 3H).

^{13}C NMR (101 MHz, Chloroform-d) δ 176.02, 154.47 (dd, *J* = 289.8, 287.3 Hz), 138.26, 136.85 (dd, *J* = 4.8, 2.6 Hz), 128.90, 124.45, 119.86, 118.57, 90.52 (dd, *J* = 22.1, 12.8 Hz), 46.74, 40.38, 38.38, 38.31, 38.09, 38.06, 37.92, 35.31 (dd, *J* = 2.5, 2.5 Hz), 33.33, 26.50, 26.40, 24.72, 22.10, 21.44.

^{19}F NMR (376 MHz, Chloroform-*d*) δ -89.05 (d, *J* = 40.1 Hz), -91.42 (d, *J* = 40.3 Hz).

HRMS (ESI) calcd for $C_{27}H_{39}ONF_2Na^+$ [(M+Na)$^+$] 454.28919, found 454.28870.

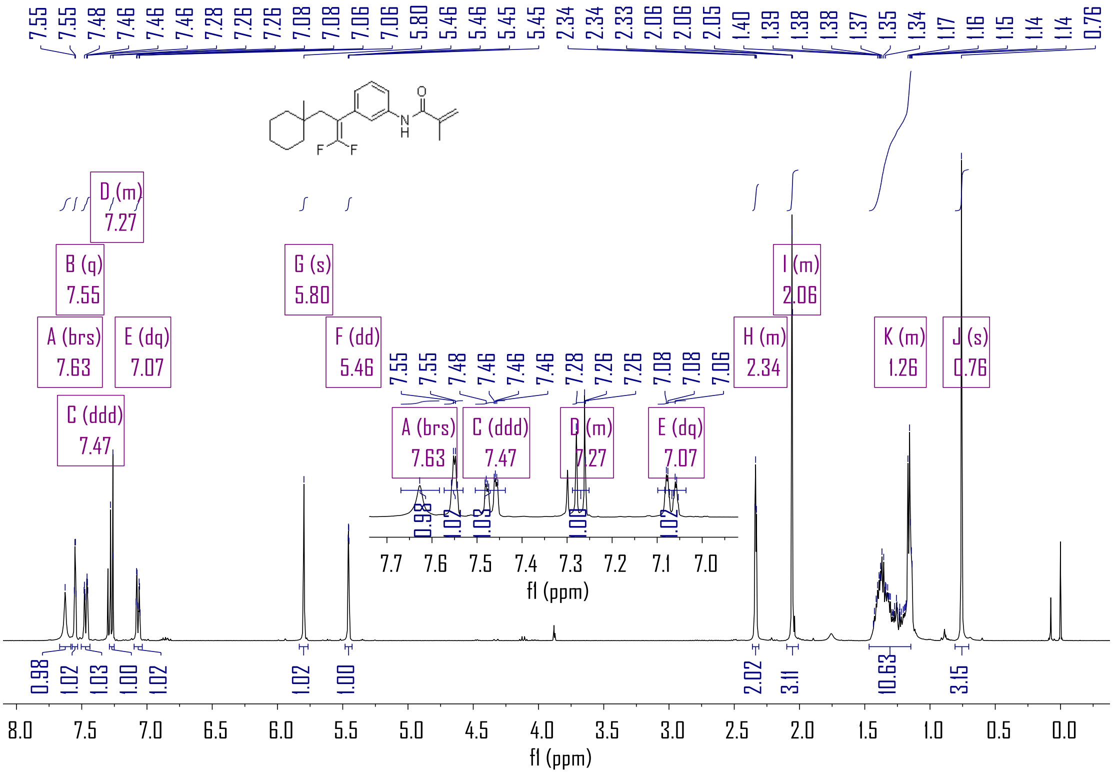

^{1}H NMR spectra for **3uba**.

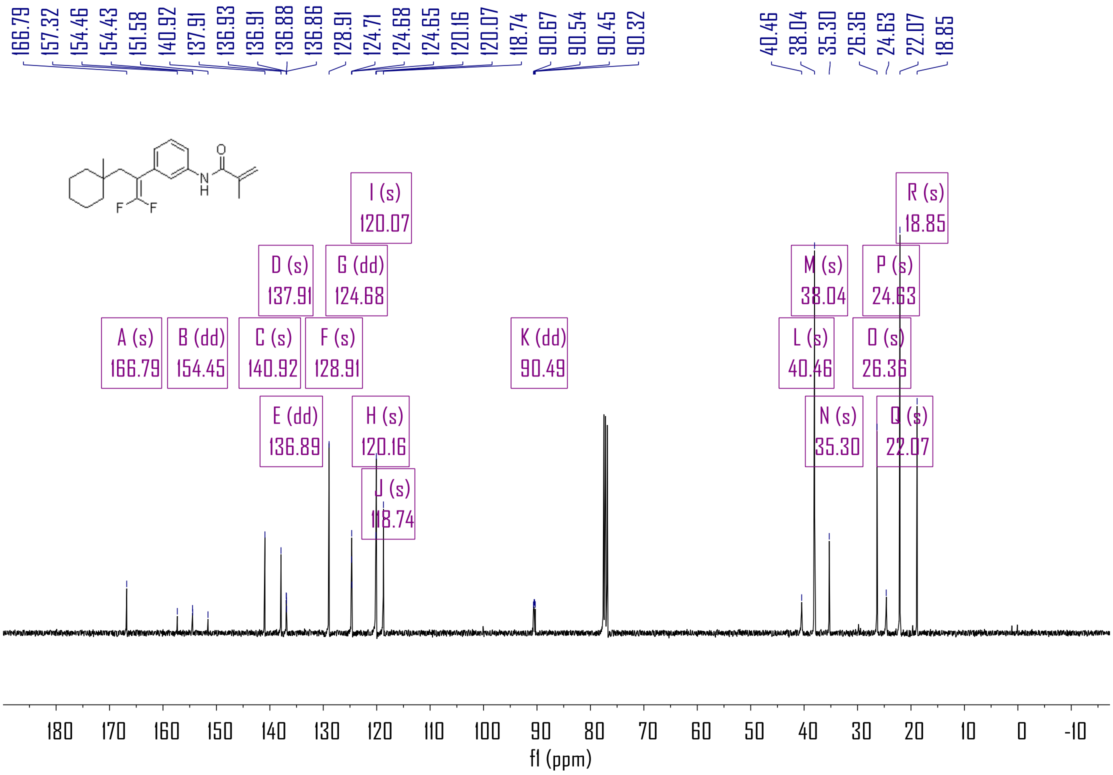

^{13}C NMR spectra for **3uba**.

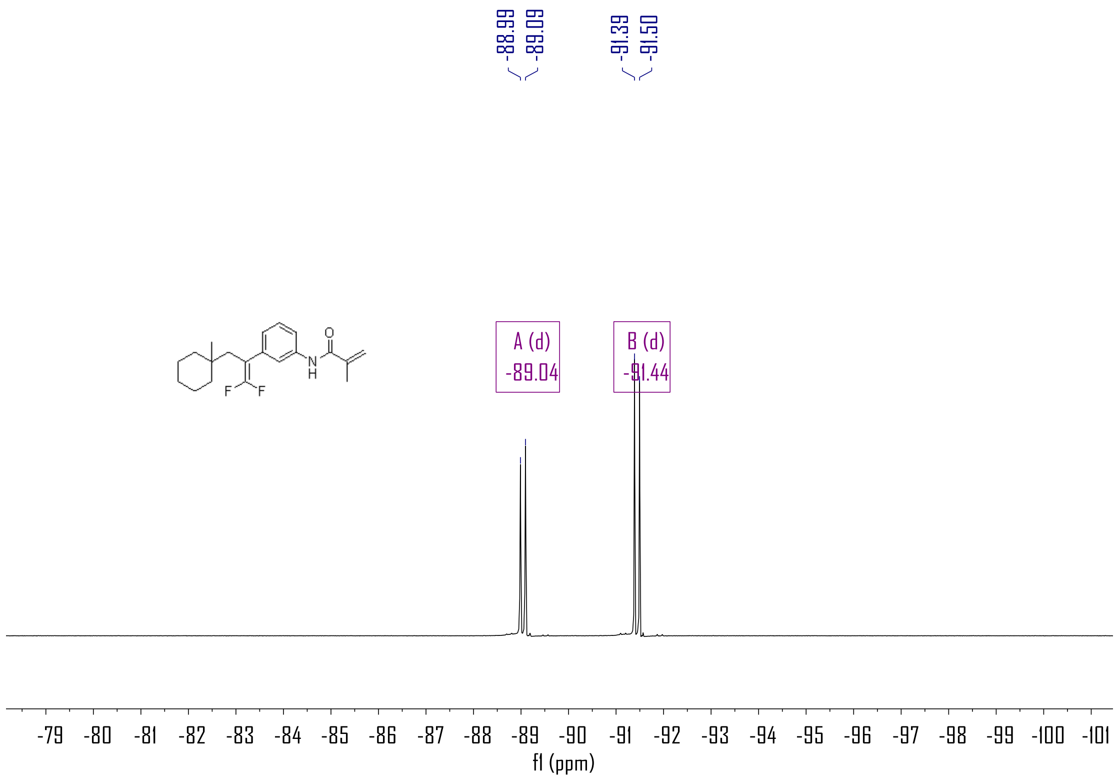

^{19}F NMR spectra for **3uba**.

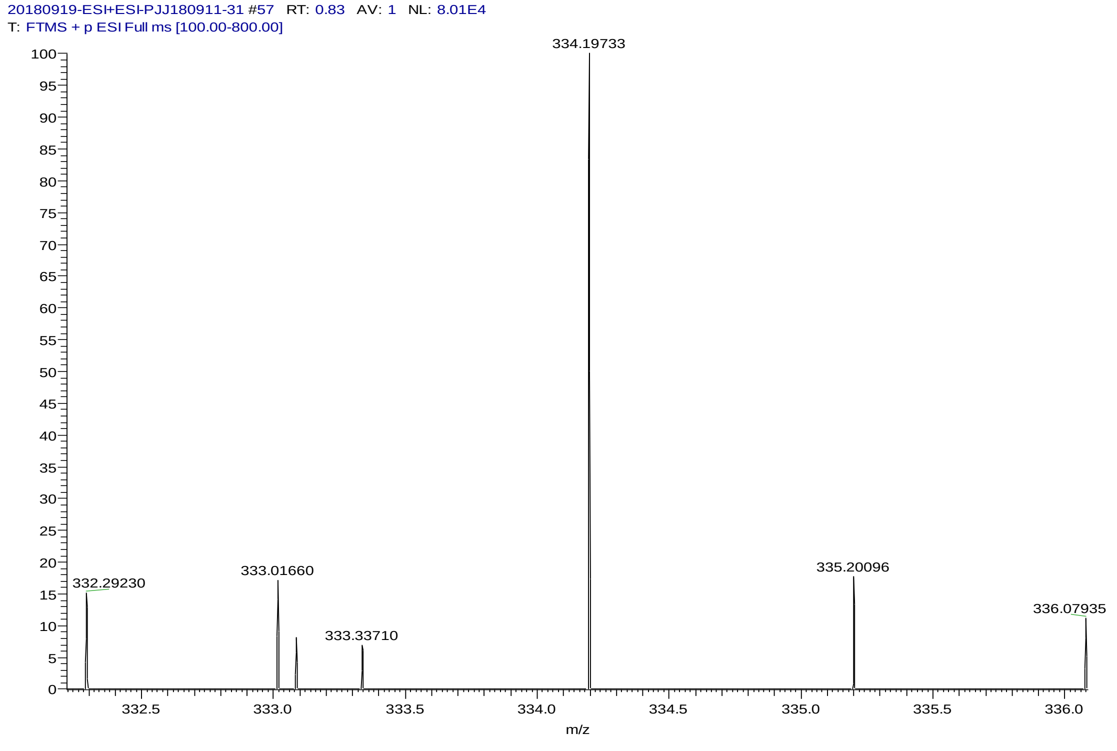

HRMS spectra for **3uba**.

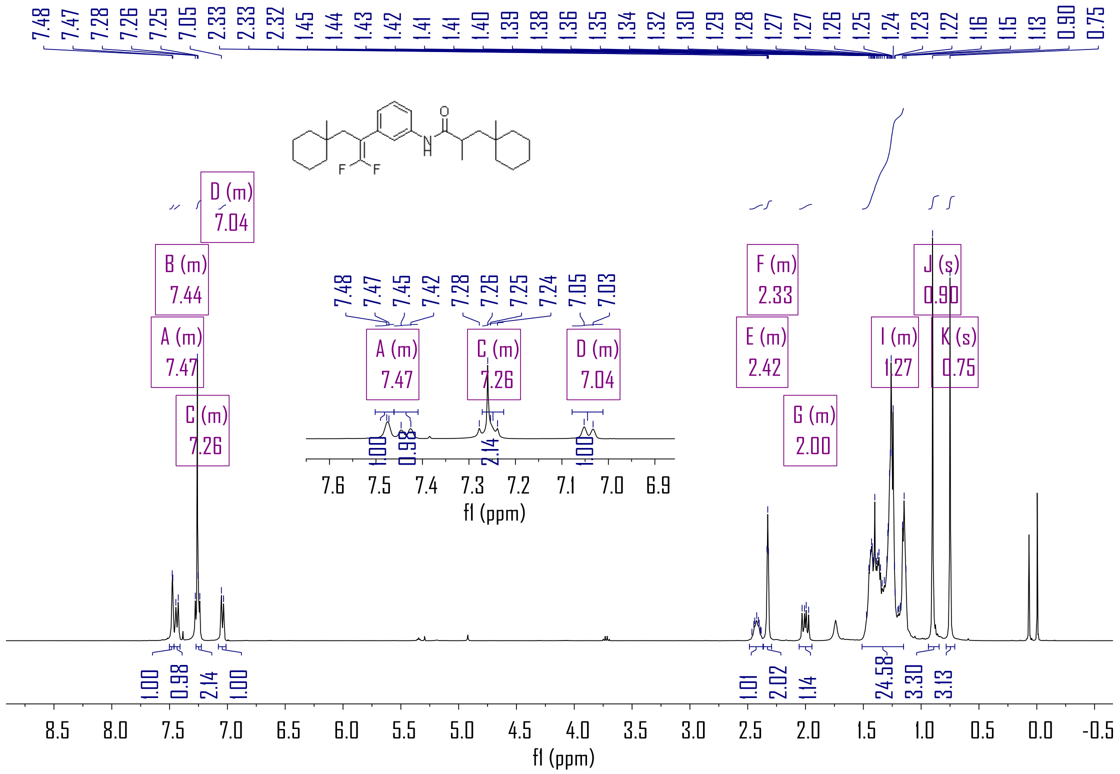

^{1}H NMR spectra for **3ubb**.

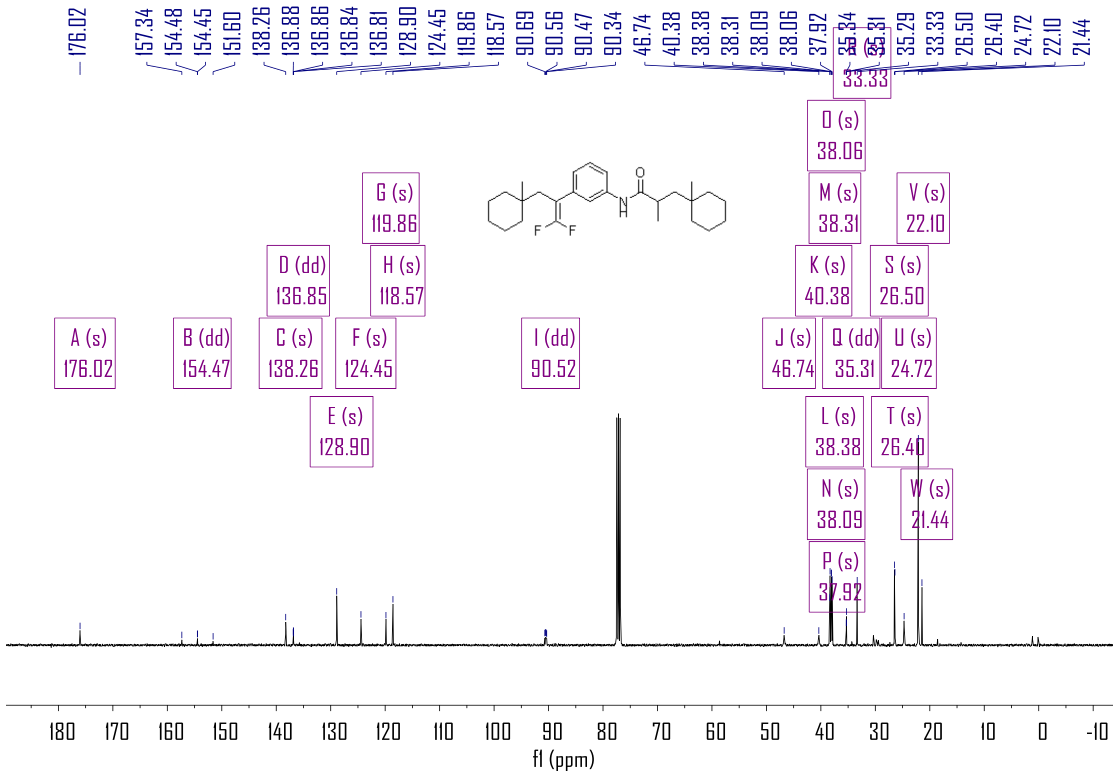

^{13}C NMR spectra for **3ubb**.

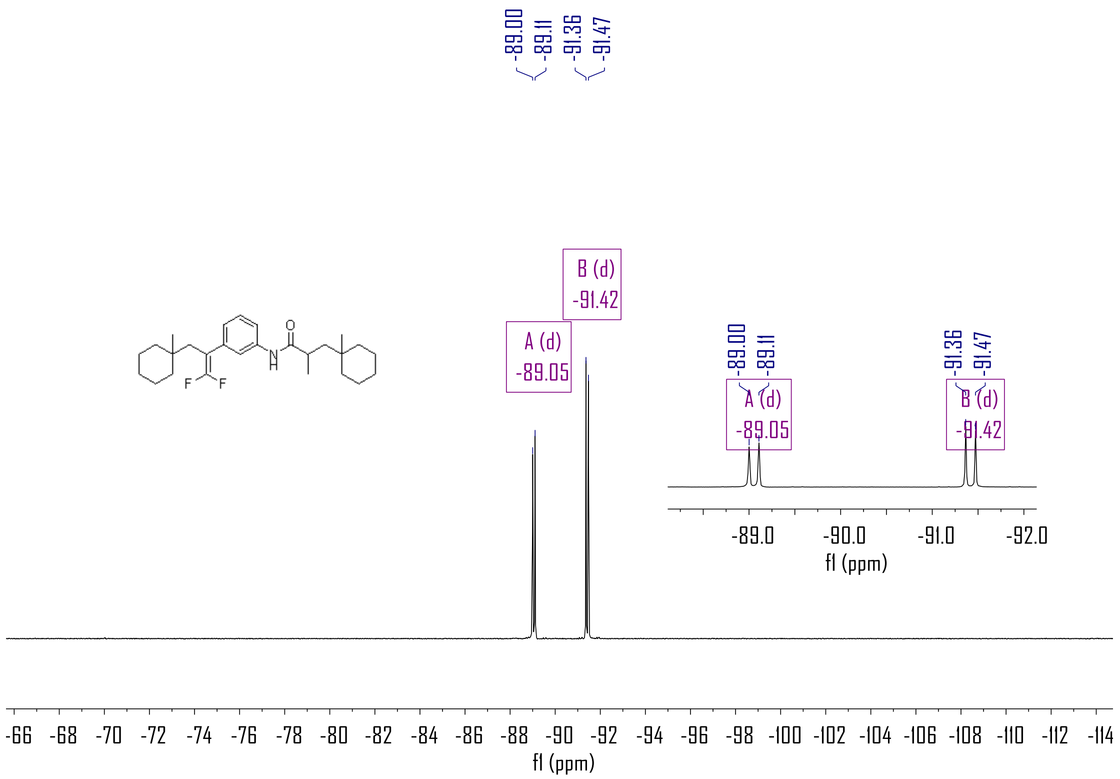

^{19}F NMR spectra for **3ubb**.

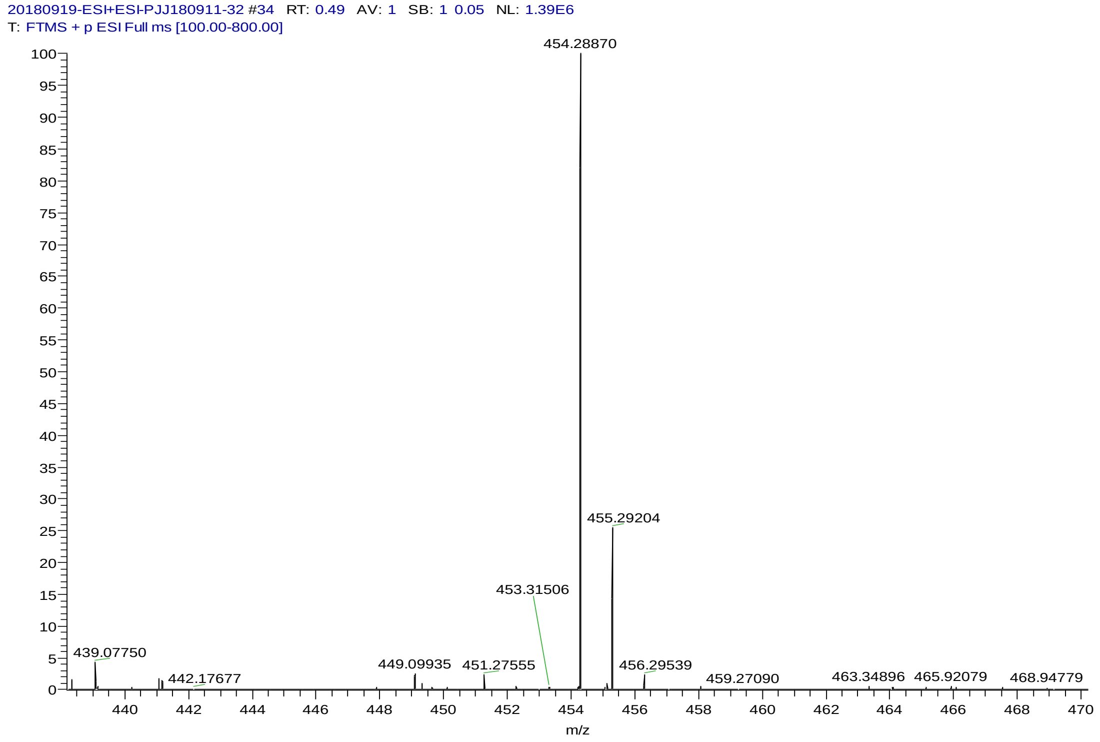

HRMS spectra for **3ubb**.

13. Examples Described in Scheme 2

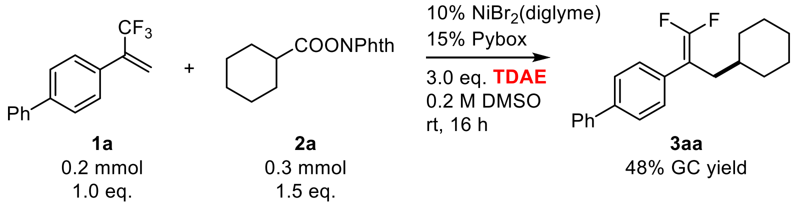

Model reaction with TDAE.

NiBr$_2$(diglyme) (0.02 mmol, 10 mol%, 7.1 mg) and Pybox (0.03 mmol, 15 mol%, 6.5 mg) were added to a Schlenk tube equipped with a stir bar. The Schlenk tube was evacuated and filled with argon (three cycles). To these solids, 1 mL DMSO was added under argon atmosphere. Then, **1a** (0.2 mmol, 1.0 equiv.), **2a** (0.3 mmol, 1.5 equiv.) and 1,1,2,2-tetrakis(dimethylamino)ethylene (0.6 mmol, 3.0 equiv.) were added and stirred at room temperature (~20 °C) for 16 hours. The yield was determined by GC with triphenylmethane as internal standard, 48%.

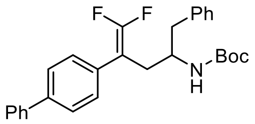

tert-butyl (4-([1,1'-biphenyl]-4-yl)-5,5-difluoro-1-phenylpent-4-en-2-yl)carbamate (**3ar**)

Following general procedure, **1a** and **2r** were used. The product was isolated by column chromatography as colorless oil (76.4 mg, 0.170 mmol, 85%).

HPLC analysis: The *ee* value was determined to be 0% by HPLC analysis on a Chiralpak AD-H column, λ = 254 nm, *n*-hexane/*i*-PrOH (90:10), flow rate = 1.0 mL/min; t_R = 6.0 min, 7.1 min.

Selectivity (desired C-F cleavage product : addition by-product) > 50:1.

R_f (petroleum ether : ethyl acetate = 20:1) = 0.17.

^{1}H NMR (400 MHz, Chloroform-*d*) δ 7.61 – 7.51 (m, 4H), 7.47 – 7.40 (m, 2H), 7.37 – 7.29 (m, 3H), 7.28 – 7.17 (m, 3H), 7.10 (d, *J* = 7.4 Hz, 2H), 4.68 – 4.29 (m, 1H), 3.97 – 3.72 (m, 1H), 2.77 (d, *J* = 6.6 Hz, 2H), 2.68 – 2.46 (m, 2H), 1.40 – 1.18 (m, 9H).

^{13}C NMR (101 MHz, Chloroform-*d*) δ 155.27, 154.47 (dd, *J* = 291.2, 287.6 Hz), 140.64, 140.32, 137.91, 132.10, 129.37, 128.89, 128.75, 128.50, 127.49, 127.30, 127.11, 126.54, 89.82 (dd, *J* = 20.3, 15.1 Hz), 80.03, 79.26, 51.75, 50.52, 41.94, 40.88, 33.24, 32.81, 28.40, 28.13.

^{19}F NMR (376 MHz, Chloroform-*d*) δ -89.69 (d, J = 40.6 Hz), -89.99 (d, J = 40.7 Hz).

HRMS (ESI) calcd for $C_{28}H_{29}O_2NF_2Na^+$ [$(M+Na)^+$] 472.20586, found 472.20561.

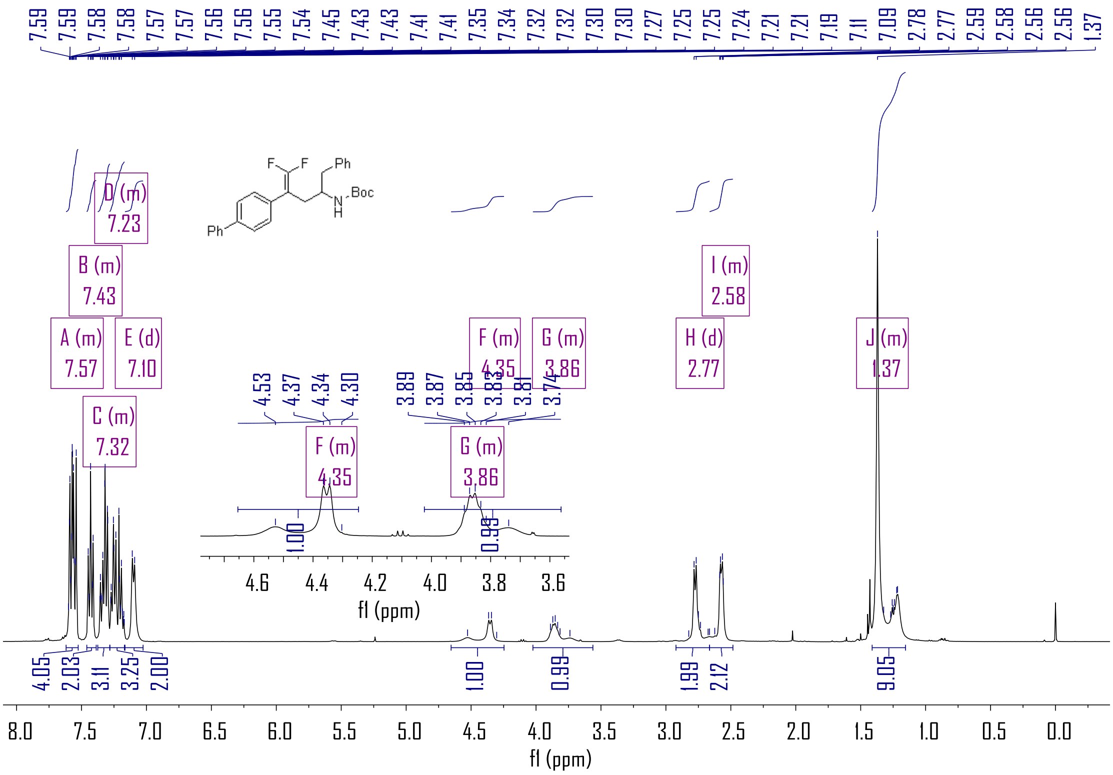

^{1}H NMR spectra for **3ar**.

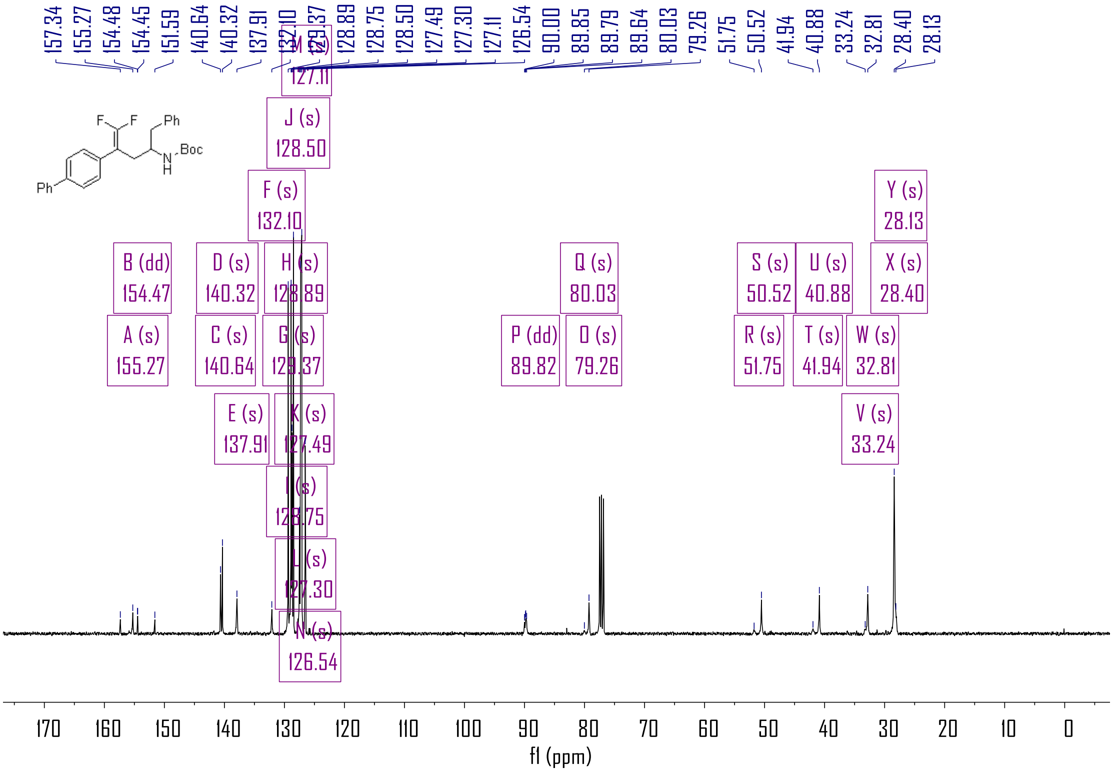

^{13}C NMR spectra for **3ar**.

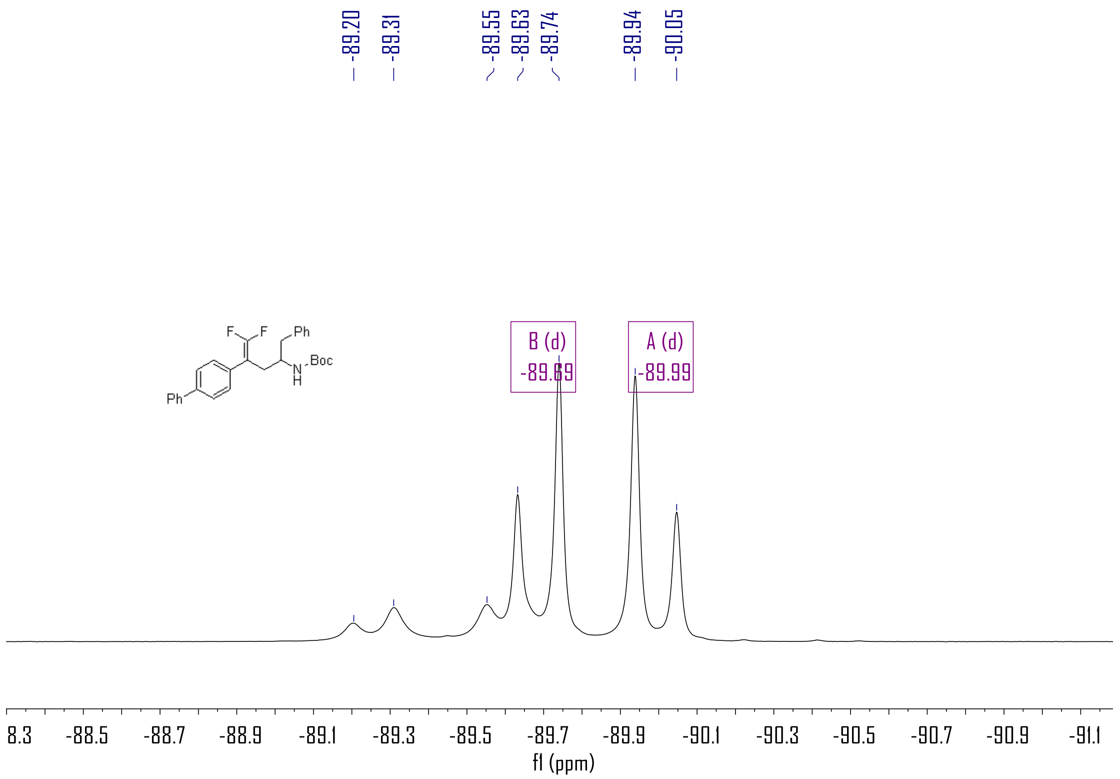

^{19}F NMR spectra for **3ar**.

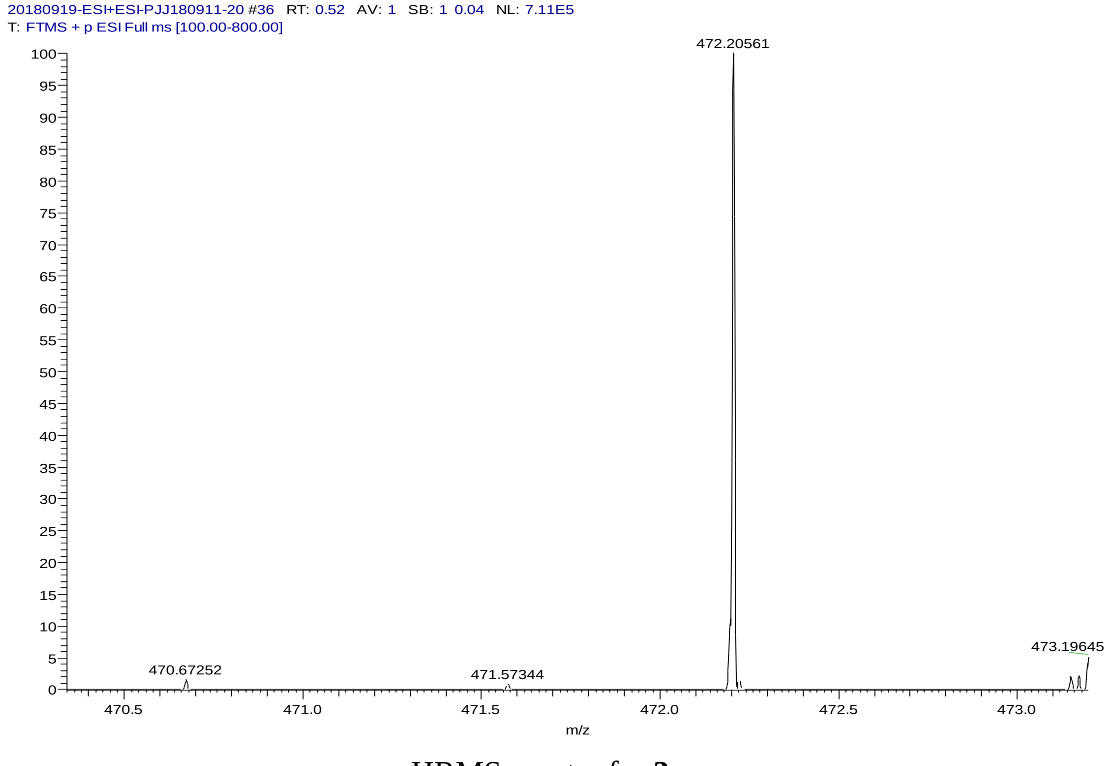

HRMS spectra for **3ar**.

14. References

[1] Zhu, Y.-Y.; Cui, C.; Li, N.; Wang, B.-W.; Wang, Z.-M.; Gao, S. *Eur. J. Inorg. Chem.* **2013**, *2013*, 3101.

[2] Liu, Y.; Zhou, Y.; Zhao, Y.; Qu, J. *Org. Lett.* **2017**, *19*, 946.

[3] Wang, X.; Xu, Y.; Deng, Y.; Zhou, Y.; Feng, J.; Ji, G.; Zhang, Y.; Wang, J. *Chem. Eur. J.* **2014**, *20*, 961.

[4] Li, Y.; Zhao, B.; Dai, K.; Tu, D.-H.; Wang, B.; Wang, Y.-Y.; Liu, Z.-T.; Liu, Z.-W.; Lu, J. *Tetrahedron* **2016**, *72*, 5684.

[5] Lu, X.; Yi, J.; Zhang, Z.-Q.; Dai, J.-J.; Liu, J.-H.; Xiao, B.; Fu, Y.; Liu, L. *Chem. Eur. J.* **2014**, *20*, 15339.

[6] Lu, X.; Xiao, B.; Zhang, Z.; Gong, T.; Su, W.; Yi, J.; Fu, Y.; Liu, L. *Nat. Commun.* **2016**, *7*, 11129.

[7] Lu, X.; Xiao, B.; Liu, L.; Fu, Y. *Chem. Eur. J.* **2016**, *22*, 11161.

[8] Zhao, W.; Wurz, R. P.; Peters, J. C.; Fu, G. C. *J. Am. Chem. Soc.* **2017**, *139*, 12153.

[9] Pratsch, G.; Lackner, G. L.; Overman, L. E. *J. Org. Chem.* **2015**, *80*, 6025.

[10] Xu, X.; Sun, J.; Lin, Y.; Cheng, J.; Li, P.; Yan, Y.; Shuai, Q.; Xie, Y. *Org. Biomol. Chem.* **2017**, *15*, 9875.

[11] Li, H.; Breen, C. P.; Seo, H.; Jamison, T. F.; Fang, Y.-Q.; Bio, M. M. *Org. Lett.* **2018**, *20*, 1338.

[12] Yu, L.; Tang, M.-L.; Si, C.-M.; Meng, Z.; Liang, Y.; Han, J.; Sun, X. *Org. Lett.* **2018**, *20*, 4579.

[13] Cheng, W.-M.; Shang, R.; Fu, M.-C.; Fu, Y. *Chem. Eur. J.* **2017**, *23*, 2537.

[14] Schwarz, J.; König, B. *Green Chem.* **2016**, *18*, 4743.

[15] Lu, X.; Wang, Y.; Zhang, B.; Pi, J.-J.; Wang, X.-X.; Gong, T.-J.; Xiao, B.; Fu, Y. *J. Am. Chem. Soc.* **2017**, *139*, 12632.

[16] Chen, H.; Jia, X.; Yu, Y.; Qian, Q.; Gong, H. *Angew. Chem. Int. Ed.* **2017**, *56*, 13103.

[17] Bartleson, J. D.; Burk, R. E.; Lankelma, H. P. *J. Am. Chem. Soc.* **1946**, *68*, 2513.
